# Atezolizumab in early triple-negative breast cancer: the randomized phase 3 NSABP B-59/GeparDouze trial

**DOI:** 10.1038/s41591-026-04565-6

**Published:** 2026-09-01

**Authors:** Sibylle Loibl, Gong Tang, Valentina Nekljudova, Priya Rastogi, Sivaramakrishna Rachakonda, Mattea Reinisch, Joshua Acosta, Andreas Schneeweiss, Christie Hilton, Sabine Seiler, Rohit Bhargava, Thomas Karn, Patricia Cortazar, Fernando Moreno, Jay Andersen, Stephani Christensen, Peter Klare, Sujatha Murali, Serafín Morales, Jens Huober, Jean-François Boileau, Álvaro Rodríguez-Lescure, Dominique Boudreau, Peter J. Polewski, Julia Teply-Szymanski, João Mouta, Eleftherios P. Mamounas, Carsten Denkert, Norman Wolmark, Charles E. Geyer

**Affiliations:** 1 GBG c/o GBG Forschungs GmbH, Neu-Isenburg, Germany; 2 University Cancer Center (UCT), Goethe University Frankfurt, Frankfurt, Germany; 3 University Cancer Center (UCT) Frankfurt-Marburg, Frankfurt, Germany; 4 NRG Oncology Statistical and Data Management Center; Department of Biostatistics and Health Data Science, University of Pittsburgh, Pittsburgh, PA USA; 5 NSABP Foundation, Inc., Pittsburgh, PA USA; 6 University of Pittsburgh School of Medicine and UPMC Hillman Cancer Center, Pittsburgh, PA USA; 7 Interdisciplinary Breast Unit, Kliniken Essen-Mitte, Essen, Germany; 8 Breast Unit, University Hospital, Mannheim, Germany; 9 Allegheny Health Network, Pittsburgh, PA USA; 10 National Center for Tumor Diseases (NCT); Heidelberg University Hospital and German Cancer Research Center, Heidelberg, Germany; 11 Sana Klinikum Offenbach, Offenbach, Germany; 12 Magee-Womens Hospital; Department of Pathology, University of Pittsburgh, Pittsburgh, PA USA; 13 Department of Gynecology and Obstetrics, University of Frankfurt, Frankfurt, Germany; 14 Genentech Inc., San Francisco, CA USA; 15 Department of Medical Oncology, Hospital Universitario Clínico San Carlos, Madrid, Spain; 16 GEICAM Spanish Breast Cancer Group, Madrid, Spain; 17 Compass Oncology, Sarah Cannon Research Institute, Portland, OR USA; 18 Comprehensive Cancer Centers of Nevada, Henderson, NV USA; 19 MediOnko-Institut GbR Berlin, Berlin, Germany; 20 Southern CA Permanente Medical Group, San Diego, CA USA; 21 Arnau de Vilanova University Hospital; Research Group of Cancer Biomarkers, Lleida Institute for Biomedical Research Dr. Pifarré Foundation, Lleida, Spain; 22 Breast Centre, Cantonal Hospital St Gallen, HOCH, St. Gallen, Switzerland; 23 University of Ulm, Ulm, Germany; 24 Segal Cancer Centre, Jewish General Hospital, McGill University, Montreal, Quebec Canada; 25 Hospital General Universitario de Elche, Elche, Spain; 26 Hôpital du Saint-Sacrement, Quebec, Quebec Canada; 27 Advocate Aurora Health, Milwaukee, WI USA; 28 Institute of Pathology, Marburg University and University Hospital Marburg (UKGM), Marburg, Germany; 29 Global Product Development Medical Affairs Oncology, Roche Farmacêutica Química, Lda., Amadora, Portugal; 30 AdventHealth Cancer Institute, Orlando, FL USA

**Keywords:** Breast cancer, Cancer therapy, Biomarkers

## Abstract

Triple-negative breast cancer (TNBC) is an aggressive subtype with an activated tumor immune microenvironment. The multicenter, multinational, double-blinded NSABP B-59/GeparDouze trial evaluated the addition of atezolizumab (atezo) (773 patients randomized) or placebo (777 patients) to sequential taxane–carboplatin–anthracycline-based neoadjuvant chemotherapy in stage II–III TNBC. The addition of atezo did not significantly improve the primary endpoint of event-free survival (EFS) (HR, 0.80 (95% CI, 0.062–1.03); stratified log-rank *P* = 0.083, 4-year EFS rates difference 3.3%). The HR for overall survival was 0.86 (95% CI, 0.62–1.19), with a 4-year benefit of 0.7%. Prespecified subgroup analyses suggested heterogeneity in EFS, with benefit of atezo in patients presenting with clinical lymph node involvement (*P*_interaction_ = 0.039). Treatment-emergent adverse events with grades ≥3 were reported in 75.3% (atezo) versus 73.4% (placebo), and immune-related adverse events were reported in 27.6% (atezo) versus 11.4% (placebo). A total of 196 (25.5%) patients discontinued atezo and 143 (18.8%) patients discontinued placebo in the neoadjuvant phase. In an exploratory mRNA-based subset analysis, patients with basal-like immune-activated tumors may have benefited from atezo. TNBC subtyping to identify basal-like immune-activated tumors and quantification of tumor-infiltrating lymphocytes to identify tumors with high tumor-infiltrating lymphocyte counts could be a promising strategy to identify patients who benefit from the addition of immune checkpoint inhibitors to neoadjuvant chemotherapy. ClinicalTrials.gov registration: NCT03281954.

## Abstract (web summary)

The phase 3 NSABP B-59/GeparDouze trial, in which patients with early-stage triple-negative breast cancer received neoadjuvant chemotherapy with or without atezolizumab, did not meet its primary endpoint of event-free survival, and comprehensive translational analyses show tumor microenvironment-dependent determinants of response, with basal-like immune-activated tumors potentially responding to atezolizumab and chemotherapy.

## Main

Triple-negative breast cancer (TNBC) is an aggressive and relatively immunogenic subtype with potential susceptibility to immune checkpoint inhibitors (ICIs)^1,2^. Several neoadjuvant studies in early TNBC have shown a moderately increased pathological complete response (pCR) rate of neoadjuvant chemotherapy (NACT) with ICIs^3,4^. In addition to pCR improvement, KEYNOTE-522 showed a statistically significant improvement in event-free survival (EFS) and overall survival (OS), irrespective of programmed death-ligand 1 (PD-L1) status, with the addition of anti-PD-1 antibody pembrolizumab to chemotherapy^5,6^.

Atezolizumab, an anti-PD-L1 antibody, significantly improved progression-free survival when added to nab-paclitaxel for the first-line treatment of metastatic, PD-L1-positive TNBC^7^. It also improved the pCR rate of early-stage TNBC with a trend for improved long-term outcomes when added to taxane–anthracycline-based NACT^8^.

**Extended Data Fig. 1:**
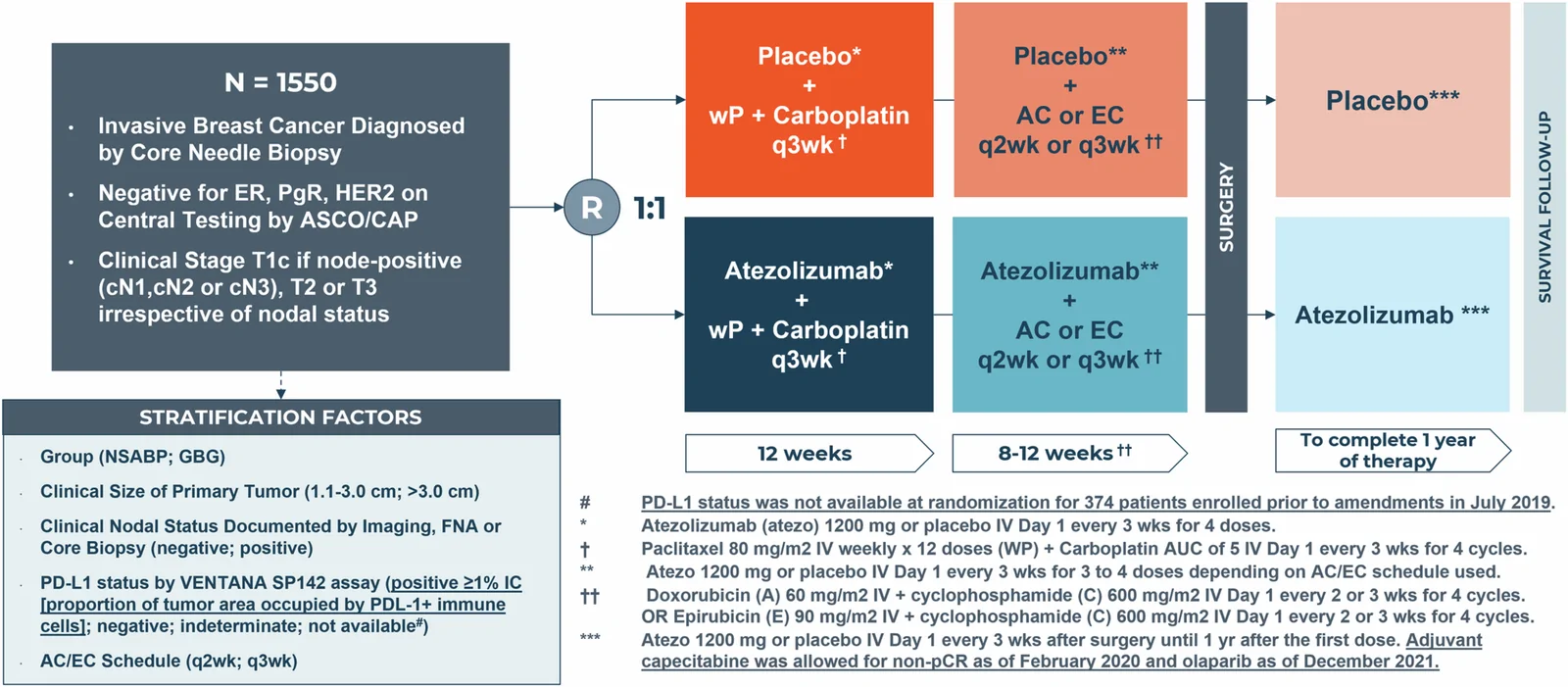
**Study design**.

NSABP B-59/GeparDouze (NCT03281954) was an international, double-blind, placebo-controlled, academic phase 3 clinical trial which investigated whether the addition of atezolizumab to neoadjuvant taxane–carboplatin–anthracycline-based chemotherapy would improve outcomes for patients with stages II–III centrally confirmed TNBC. The study was developed as a contemporary to the global KEYNOTE-522 trial. The study design is shown in Extended Data Fig. 1.

In this Article, we present the primary analysis (4-year EFS results) and secondary analyses (pCR, distant disease-free survival (DDFS), OS, safety) of the NSABP B-59/GeparDouze trial along with exploratory translational investigations focusing on the prognostic and predictive potential of tumor-infiltrating lymphocytes (TILs). In a prospectively planned sub-study, mRNA analysis and TNBC molecular subtyping of primary tumors were integrated. An exploratory analysis of the outcomes by mRNA-based TNBC subtypes was implemented and compared with the TIL results.

## Results

### Patients and therapy

Between 29 December 2017 and 28 May 2021, 2,188 patients were screened and 1,550 patients with centrally confirmed TNBC were enrolled across 353 sites in 4 countries (USA, Germany, Canada and Spain), with 773 patients randomly assigned to receive neoadjuvant atezolizumab 1,200 mg IV and 777 patients to receive placebo IV every 3 weeks concurrently with NACT. Chemotherapy was composed of paclitaxel 80 mg m^−2^ IV weekly × 12 doses with carboplatin area under the concentration-time curve (AUC) of 5 IV on day 1 and every 3 weeks for 4 cycles, followed by doxorubicin (anthracyclin, A) 60 mg m^−2^ IV or epirubicin (E) 90 mg m^−2^ IV with cyclophosphamide (C) 600 mg m^−2^ IV on day 1 and every 2 weeks or 3 weeks per investigator discretion for 4 cycles (AC and EC, respectively). Adjuvant atezolizumab or placebo was administered to complete 1 year of study therapy.

**Fig. 1:**
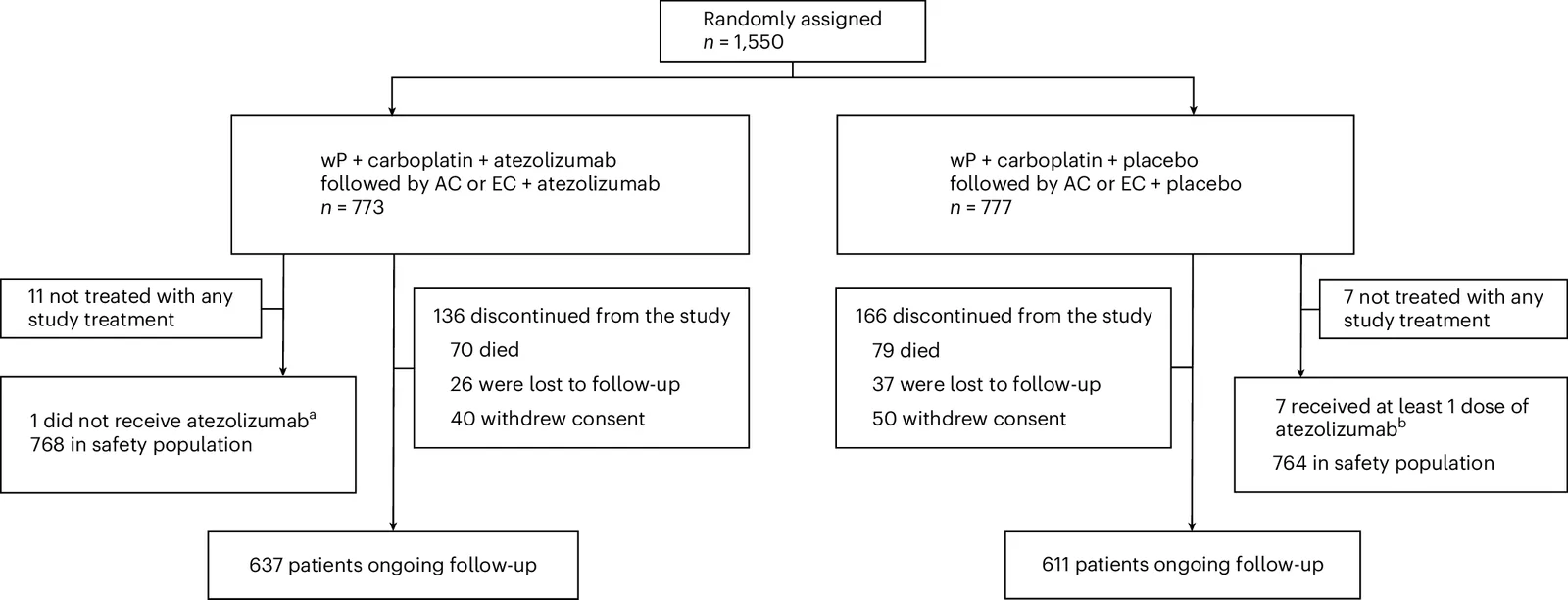
Consort diagram. Disposition of patients in the NSABP B-59/GeparDouze trial. ^a^Analyzed as receiving placebo for the safety analysis. ^b^Analyzed as receiving atezolizumab for the safety analysis.

The treatment assignment and analysis populations are shown in Fig. 1. The baseline patient and tumor characteristics were well balanced (Table 1). The stratification factors were distributed as follows: 978 patients were enrolled in Germany and Spain and 572 in the USA and Canada. A total of 649 (41.9%) patients were node positive, 640 (41.3%) had tumors larger than 3 cm, 697 (45%) had a centrally positive PD-L1 status and 984 (63.5%) received AC or EC every 2 weeks.

**Table 1:** Baseline characteristics: demographic and baseline disease characteristics

Parameter	CT/atezolizumab (*n* = 773)	CT/placebo (*n* = 777)	Overall (*n* = 1,550)
**Age (years)**			
Median	49.0	49.0	49.0
Range	22, 79	23, 77	22, 79
**Sex**			
Female	773 (100%)	776 (99.9%)	1,549 (99.9%)
Male	0 (0%)	1 (0.1%)	1 (0.1%)
**Group**			
GBG	488 (63.1%)	490 (63.1%)	978 (63.1%)
NSABP	285 (36.9%)	287 (36.9%)	572 (36.9%)
**Nodal status**			
Negative (cN0)	445 (57.6%)	456 (58.7%)	901 (58.1%)
Positive (cN1, cN2, cN3)	328 (42.4%)	321 (41.3%)	649 (41.9%)
**AC and EC schedule**			
Every 2 weeks (q2w)	489 (63.3%)	495 (63.7%)	984 (63.5%)
Every 3 weeks (q3w)	284 (36.7%)	282 (36.3%)	566 (36.5%)
**Clinical size of the primary tumor (cm)**			
1.1–3.0	453 (58.6%)	457 (58.8%)	910 (58.7%)
>3.0	320 (41.4%)	320 (41.2%)	640 (41.3%)
**Tumor stage**			
I^a^	4 (0.5%)	4 (0.5%)	8 (0.5%)
IIa	428 (55.4%)	442 (56.9%)	870 (56.1%)
IIb	233 (30.1%)	238 (30.6%)	471 (30.4%)
IIIa	72 (9.3%)	67 (8.6%)	139 (9.0%)
IIIb	33 (4.3%)	25 (3.2%)	58 (3.7%)
Unknown or missing	3 (0.4%)	1 (0.1%)	4 (0.3%)
**PD-L1 status**^b^			
Negative or indeterminate	437 (55.2%)	426 (54.8%)	853 (55.0%)
Positive	346 (44.8%)	351 (45.2%)	697 (45.0%)
**Histological tumor type**			
Invasive ductal or invasive carcinoma of no special type	729 (94.3%)	740 (95.2%)	1469 (94.8%)
Invasive lobular carcinoma or mixed lobular carcinoma	9 (1.2%)	10 (1.3%)	19 (1.2%)
Other	35 (4.5%)	27 (3.5%)	62 (4.0%)
**Tumor grade**			
1	7 (0.9%)	9 (1.2%)	16 (1.0%)
2	140 (18.1%)	135 (17.4%)	275 (17.7%)
3	626 (81.0%)	631 (81.2%)	1257 (81.1%)
Unknown or missing	0 (0%)	2 (0.3%)	2 (0.1%)
**Stromal TIL category on baseline specimen** (%)			
<10%	180 (23.3%)	170 (21.8%)	350 (22.6%)
10–29%	300 (38.8%)	310 (39.9%)	610 (39.3%)
≥30%	288 (37.3%)	295 (38.0%)	583 (37.6%)
Missing	5 (0.6%)	2 (0.3%)	7 (0.5%)
**Germline pathogenic variant status**			
*BRCA1* pathogenic variant	67 (8.7%)	62 (8.0%)	129 (8.3%)
*BRCA2* pathogenic variant	22 (2.8%)	16 (2.1%)	38 (2.5%)
Wild type for *BRCA1* and *BRCA2*	394 (51.0%)	425 (54.7%)	819 (52.8%)
Missing *BRCA* germline testing status	294 (38%)	275 (35.4%)	569 (36.7%)

^a^Exclusion according to protocol.

^b^Including assessed retrospectively (for patients recruited before amendment introducing stratification by PD-L1). CT, chemotherapy.

Following random assignment, 18 patients never started neoadjuvant therapy. Surgery was performed in 1,495 patients (96.5%). Adjuvant study therapy was initiated in 1,191 patients (572 of 773 with atezolizumab (74.0%) and 619 of 777 with placebo (79.7%); Supplementary Table 1). An amendment allowing adjuvant capecitabine to be administered with the study medication for patients with a non-pCR status became active as of 20 May 2020 after 673 patients had been enrolled and 468 patients reached surgery. Before the amendment, 54 of 191 (28.3%) patients with a non-pCR status received capecitabine but were required to discontinue study medication. After the amendment, surgery was scheduled in 1,082 patients, and 245 of 427 (54.7%) patients with a non-pCR status received capecitabine. An amendment allowing olaparib for patients with a non-pCR status and pathogenic germline variants in *BRCA1* or *BRCA2* became effective as of 12 May 2021, and 9 patients subsequently received olaparib (hence discontinuing atezolizumab and placebo per protocol).

### Efficacy

**Fig. 2:**
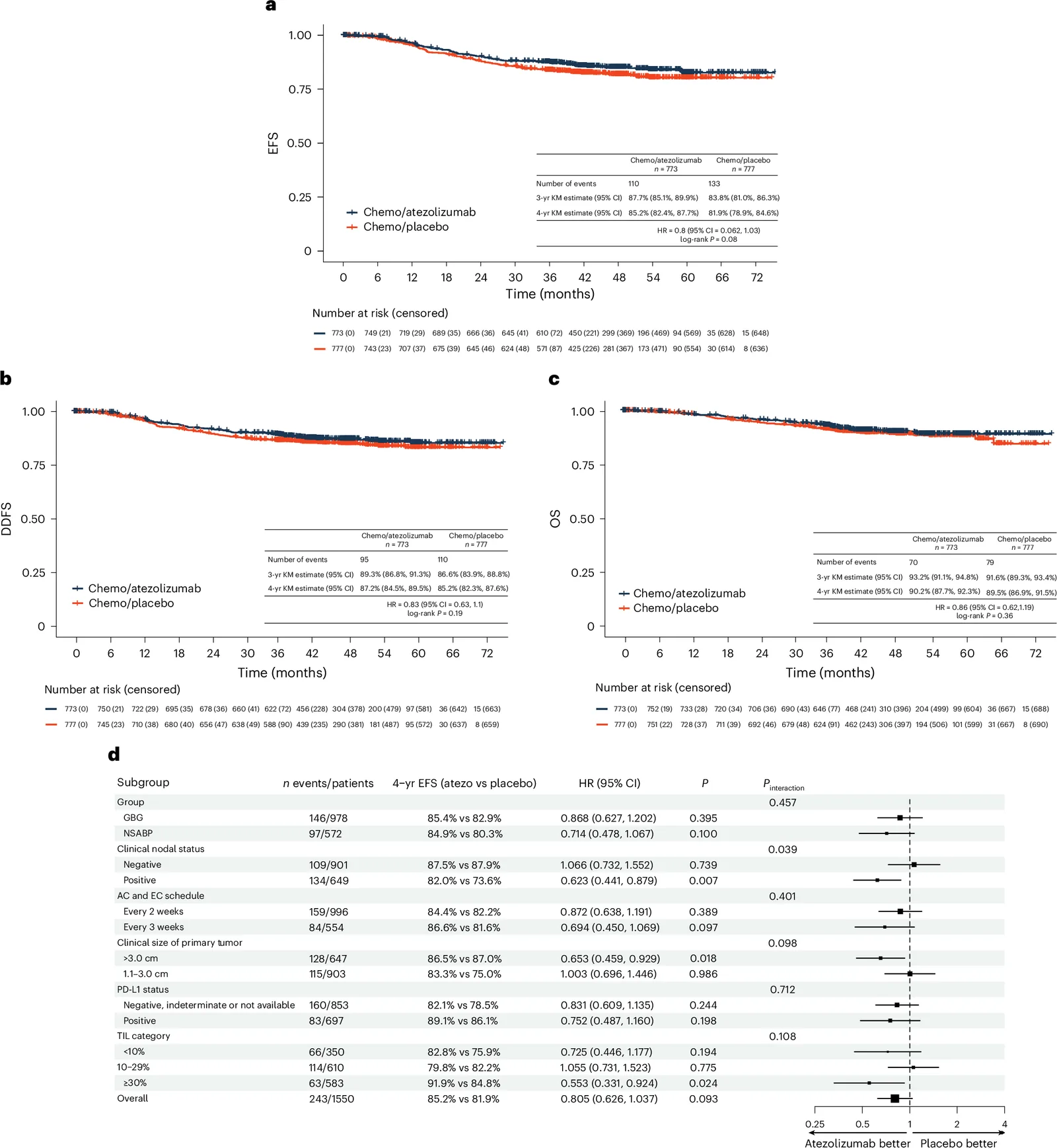
Time-to-event endpoints. **a**–**c**, Kaplan–Meier (KM) plots of EFS (**a**), DDFS (**b**) and OS (**c**) in the overall ITT population per treatment arm. **d**, Forest plot of EFS in stratified and further prespecified subgroups for all patients. Squares are point estimates for the hazard ratio of atezolizumab versus placebo (the size of the square represents the group size); a horizontal line passing through each square represents the 95% CI. x axis is under log scale. The *P* values are from the unstratified two-sided Wald test from the Cox proportional hazard model; two-sided test for interaction of the Wald test from a Cox regression with an interaction term. Atezo, atezolizumab; Chemo, chemotherapy; DDFS, distant disease-free survival; ITT, intent-to treat; OS, overall survival.

At the data cutoff of 16 September 2024, the median duration of follow-up was 46.9 months (interquartile range, 41.0–54.8 months; 90 patients withdrew consent, and 63 patients were lost to follow-up; all other patients were followed until the end of the study) with 243 patients (15.7%) having had an EFS event (110 in the atezolizumab arm and 133 in the placebo arm). The 4-year EFS rate was 85.2% with atezolizumab and 81.9% with placebo; the stratified hazard ratio (HR) was 0.800 (95% confidence interval (CI), 0.621–1.030); stratified log-rank test *P* = 0.083 (Fig. 2a), which did not meet the prespecified boundary for statistical significance. However, prespecified subset analyses suggested heterogeneity among subgroups, with higher-risk patients deriving possible benefit from atezolizumab. Patients with clinical nodal involvement had an HR of 0.623 (95% CI, 0.441–0.879; *P* = 0.007) for atezolizumab relative to placebo while those with negative nodal involvement had an HR of 1.066 (95% CI, 0.732–1.552; *P* = 0.739) with *P*_interaction_ = 0.039. For other prespecified subgroups, the HR differences were similar but interaction tests did not reach statistical significance. For example, patients with tumors larger than 3 cm had an HR of 0.653 (*P* = 0.018) while those with smaller tumors had an HR of 1.003 (*P* = 0.986) with *P*_interaction_ = 0.098. Those with TILs ≥30% had an HR of 0.553 (*P* = 0.024), those with 10–29% TILs had an HR of 1.055 (*P* = 0.775) and those with <10% TILs had an HR of 0.725 (*P* = 0.194) with *P*_interaction_ = 0.108 (Fig. 2d). The PD-L1 status as analyzed in this trial was not significant (Fig. 2d).

Most EFS events were distant recurrences (*n* = 135; 56%), 77 with placebo and 58 with atezolizumab. Of those 135, 53 (39%) of first distant recurrences involved the central nervous system (CNS) (24 with placebo and 29 with atezolizumab). One patient in the placebo arm had progression during neoadjuvant therapy not amenable for surgery. There were no major differences between the two treatment arms regarding the other EFS events (Supplementary Table 2).

**Extended Data Fig. 2:**
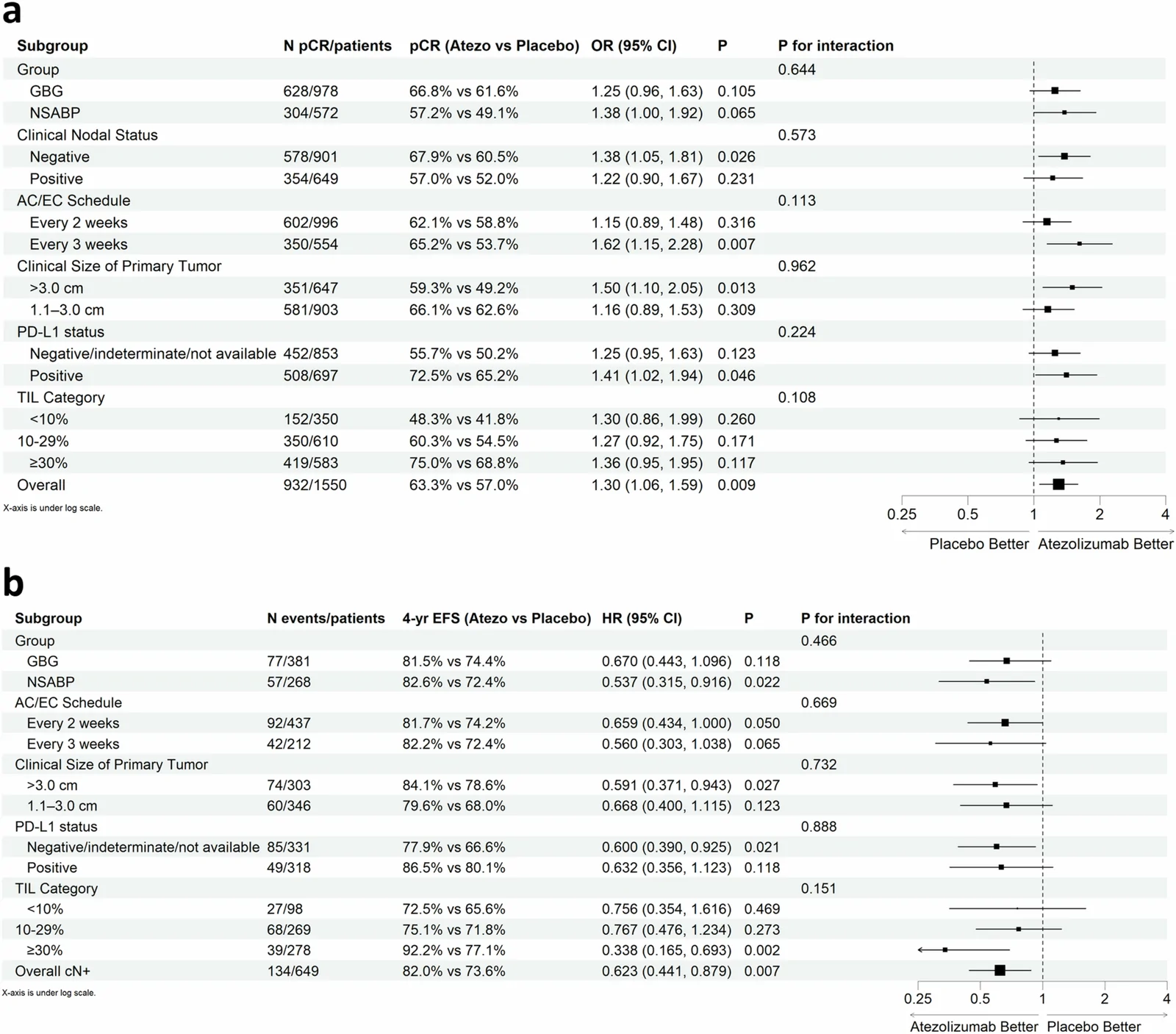
Forest plot of pCR in for all patients and EFS for nodal positive population. **a**: Forest plot of pCR in for all patients. **b**: Forest plot of EFS in stratified and further pre-specified subgroups for nodal positive population. Squares are point estimates for odds ratio atezolizumab vs placebo (the size of the square represents the group size); a horizontal line passes through each square, representing the 95% confidence interval. **a**: *P*-value is from the unstratified 2-sided Wald test from the logistic regression; 2-sided test for interaction is Breslow-Day except for the TILs where Wald test from a logistic regression with an interaction term was used. **b**: *P*-value is from the unstratified 2-sided Wald test from the Cox proportional hazard model; 2-sided test for interaction the Wald test from a Cox regression with an interaction term.

Overall, 63.3% of patients in the atezolizumab arm and 57.0% of patients in the placebo arm had a pCR (secondary endpoint) with an absolute difference of 6.3% (*P* = 0.009). Patients with pCR had a significantly better outcome than those without. Patients with pCR had a 4-year EFS of 93.0% with atezolizumab and 91.4% with placebo. The patients with non-pCR had a 4-year EFS of 71.3% with atezolizumab and 71.1% with placebo. Subset analyses of pCR rates by arm, stratification variables and TILs are presented in Extended Data Fig. 2a. There were higher pCR rates with atezolizumab in node-positive patients (unstratified *P* = 0.026), those treated every 3 weeks with AC or EC (unstratified *P* = 0.007), those with larger tumors (unstratified *P* = 0.013) and patients with PD-L1-positive tumors (unstratified *P* = 0.046). However, interaction testing by treatment arm was negative for all groups.

Capecitabine as adjuvant therapy for patients with a non-pCR status after NACT and atezolizumab or placebo was allowed after an amendment. Overall, about half of the patients with a non-pCR status received capecitabine (atezolizumab 46.8% and placebo 49.7%). There was no apparent treatment effect of atezolizumab in either the cohort of non-pCR patients receiving capecitabine or the cohort not receiving capecitabine (post hoc; Supplementary Fig. 1). Because of the limited number of patients receiving olaparib, no analysis was carried out to explore the potential impact of olaparib.

A total of 205 patients experienced a DDFS event (95 in the atezolizumab arm and 110 in the placebo arm). The difference in the 4-year DDFS rate, 87.2% with atezolizumab and 85.2% with placebo, was not statistically significant (HR, 0.834 (95% CI, 0.633–1.098); *P* = 0.193) (Fig. 2b).

There were 70 deaths reported in the atezolizumab arm and 79 in the placebo arm (HR, 0.86 (95% CI, 0.62–1.19)). The 4-year OS was similar in the two treatment arms, 90.2% and 89.5%, respectively (Fig. 2c).

### Safety and drug delivery

The safety overview is presented in Table 2. Treatment-emergent adverse event (TEAE) grades ≥3 were reported in 75.3% of patients receiving atezolizumab versus 73.4% receiving placebo. Serious adverse events were reported in 35.2% and 30.2%, respectively. Five patients died during treatment, two patients in the atezolizumab arm (non-neutropenic sepsis and severe hyponatremia) and three in the placebo arm (sudden death, pneumonitis and unknown).

**Table 2:** Adverse events: selected TEAEs by highest grade in at least 5% of patients by arms as treated

TEAE (%)	Atezolizumab (*n* = 768)					Placebo (*n* = 764)				
	Grade 1	Grade 2	Grade 3	Grade 4	Total	Grade 1	Grade 2	Grade 3	Grade 4	Total
Overall^a^	1.6	23.2	48.4	26.6	100	1.2	25.1	50	23	99.7
Fatigue	41.4	40.2	5.5	0	87.1	42	39.7	6	0	87.7
Nausea	48.7	21.2	1.8	0	71.7	47	25	2.1	0	74.1
Peripheral sensory neuropathy	45.7	16.9	2.6	0.1	65.3	47.1	17.7	2.9	0.1	67.8
Skin reaction	40	11.6	1	0	52.6	36.1	10.6	0.5	0	47.2
Infections and infestations	16.8	24.5	9.6	0.5	51.4	19.5	22.8	6.2	0.5	49
Anemia	6.8	21	17.7	0.4	45.9	6.7	20	17	0.8	44.5
Arthralgia	33.2	11.2	0.7	0	45.1	33.6	10.1	0.4	0	44.1
Stomatitis	28.8	12.5	1.6	0	42.9	30	11	0.4	0	41.4
Headache	29.9	9.8	0.9	0	40.6	32.3	9.3	1.3	0	42.9
Diarrhea	28.8	8.9	2.7	0.1	40.5	31.5	8	2.2	0	41.7
Neutropenia	1.6	5.6	16.3	10.9	34.4	2	4.6	16.4	8.8	31.8
Dyspnea	24.5	7	1.7	0.1	33.3	20.7	8.2	1.2	0.1	30.2
Myalgia	23	6.8	0.7	0	30.5	29.1	6.8	0.8	0	36.7
Epistaxis	28.6	0.3	0	0	28.9	27	0.8	0	0	27.8
Decreased appetite	16.3	7.7	0.5	0	24.5	16.6	6.2	0.4	0	23.2
Vertigo	18.5	3.8	0.7	0	23	19.6	4.8	0.7	0	25.1
Vomiting	15.2	6.1	1.3	0	22.6	20.8	5.4	1.4	0	27.6
ALT/AST	9.8	4.9	5.5	0.5	20.7	11.4	5.9	3.7	0.1	21.1
Insomnia	14.3	5.6	0	0	19.9	17.8	4.2	0.1	0	22.1
Cardiac disorders	12.9	3.4	1.6	0.3	18.2	10.5	3.8	0.7	0	15
Thrombocytopenia	7.6	3.9	2.5	1.7	15.7	4.6	3.4	2.6	0.9	11.5
Dyspepsia	9.8	5.1	0	0	14.9	9.3	5	0.3	0	14.6
Hypertension	6.4	5.3	2	0	13.7	6.4	4.3	2.4	0	13.1
Febrile neutropenia	0.1	0	8.1	1.8	10	0	0	7.2	1.7	8.9
Hyperglycemia	3.6	1.8	3	0.4	8.8	4.8	1.3	1.7	0.4	8.2
Embolism	1.2	3.5	1.7	0	6.4	0.7	4.1	2	0.3	7.1
Anxiety	3.3	3	0	0	6.3	6.4	3.5	0.1	0	10
Gastroesophageal reflux disease	2.1	2.7	0	0	4.8	3.4	3.5	0	0	6.9

^a^In addition, 5 patients died during treatment; 2 patients (0.3%) died in the atezolizumab arm (non-neutropenic sepsis and severe hyponatremia), and 3 patients (0.4%) died in the placebo arm (sudden death, pneumonitis and unknown).

Immune-related adverse events were reported in 27.6% of patients receiving atezolizumab and in 11.4% of patients receiving placebo. Events by treatment arm are summarized in Supplementary Table 3. The most common immune-related adverse events in patients receiving atezolizumab were hypothyroidism, hyperthyroidism and infusion-related reactions.

Complete delivery of chemotherapy was defined as the administration of ≥10 doses of weekly paclitaxel, 4 doses of carboplatin and 4 doses of AC or EC, and was achieved in 606 of 773 (78.4%) patients in the atezolizumab arm and in 632 of 777 (81.3%) patients in the placebo arm (Supplementary Fig. 2). All planned doses of atezolizumab were administered to 461 of 773 (59.6%) patients randomized to the atezolizumab arm, and all doses of placebo were administered to 528 of 777 (68.0%) patients randomized to the placebo arm. During the neoadjuvant phase, 196 (25.5%) patients discontinued atezolizumab and 143 (18.8%) patients discontinued placebo. In the adjuvant phase, an additional 116 (15.0%) and 106 (13.6%) discontinued atezolizumab and placebo early, respectively.

TEAEs leading to therapy discontinuation occurred in 163 (21.2%) patients receiving atezolizumab and 81 (10.6%) patients receiving placebo. In patients receiving atezolizumab, TEAEs led to early discontinuation of paclitaxel in 93 (12.1%), carboplatin in 65 (8.5%) and AC or EC in 40 (5.2%) patients. The corresponding early discontinuation of chemotherapy due to TEAEs in patients receiving placebo was paclitaxel in 87 (11.4%), carboplatin in 56 (7.3%) and AC or EC in 27 (3.5%), respectively.

### Subgroup analyses for TILs and nodal status

**Fig. 3:**
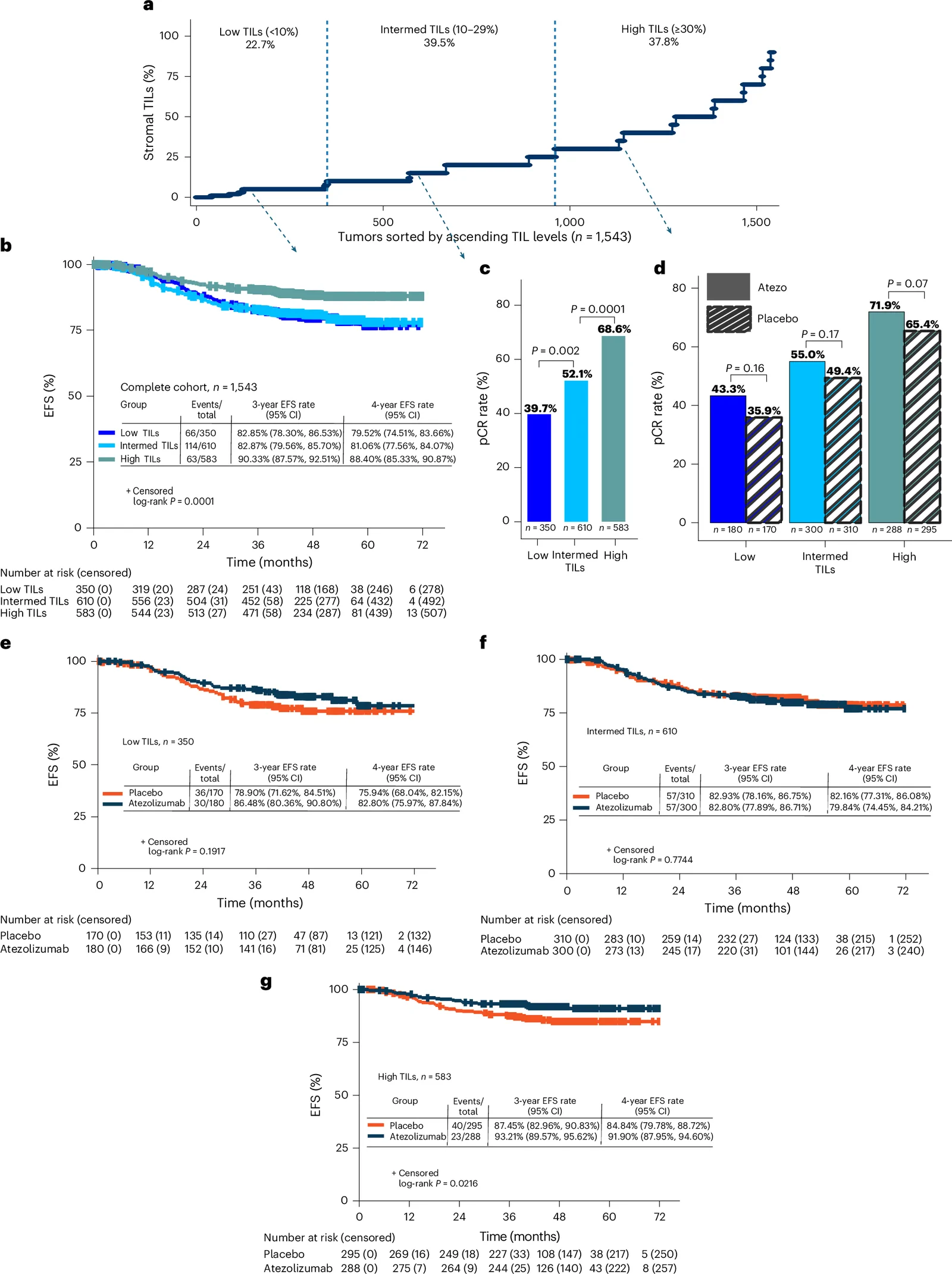
Evaluation of TILs in the complete clinical trial cohort (*n* = 1,543). **a**, Distribution of TILs and definition of three predefined groups (low TILs: <10%, 22.7% of the cohort; intermediate TILs: 10–29%, 39.5% of the cohort; high TILs: ≥30%, 37.8% of the cohort). **b**, Significantly improved prognosis of patients with high-TIL tumors in survival analysis (*P* = 0.001, log-rank test). **c**, Increased pCR rate in patients with high TILs. **d**, pCR stratified by treatment arm and TILs. **e**–**g**, Kaplan–Meier analysis of treatment effect in the three groups with low TILs (**e**), intermediate TILs (**f**) and high TILs (**g**). A significant benefit of atezolizumab therapy is observed only in patients with high TILs. The bar plots (**c** and **d**) show the proportion of patients achieving pCR in each TIL subgroup, with sample sizes indicated below the bars. Statistical significance was assessed using two‑sided Fisher’s exact tests, and *P* values are shown in the panel. intermed, intermediate.

Prespecified central evaluation of TILs was performed in 1,543 patients (99.5% of the complete cohort of 1,550 patients). Of these patients, 350 (22.7%) had low TILs (<10%), 610 (39.5%) had intermediate TILs (defined as 10–29%) and 583 (37.8%) had high TILs (defined as ≥30%; Fig. 3a); the median was 20% in both arms (range 0–90%), and the mean (s.d.) was 25% (20.4) in the placebo arm and 24.4% (19.7) with atezolizumab. TILs correlated with PD-L1 expression (Supplementary Table 4). In the complete cohort, patients with high TILs had a significantly improved EFS (*P* = 0.0001), but there was no difference in EFS between the intermediate- and the low-TIL groups (Fig. 3b). Patients with high-TIL tumors also had an increased pCR rate (68.6% for patients with high TILs, compared with 52.1% (intermediate TILs) and 39.7% (low TILs); *n* = 1,543; *P* = 0.0001 and *P* = 0.0002; Fig. 3c). However, stratification by treatment did not result in significant atezolizumab-related differences for the pCR rate within the TIL groups (Fig. 3d). No significant EFS differences were observed in the Kaplan–Meier plots in the low- and intermediate-TIL subgroups (Fig. 3e,f), but an improvement in EFS with atezolizumab was suggested in patients with tumors with high TILs (*n* = 583, *P* = 0.0216; Fig. 3g).

As part of the exploratory analyses, subgroups defined by TILs and nodal status were further evaluated. The benefit from atezolizumab appeared to be most pronounced in the node-positive patients with high TILs (≥30%), with 77.1% of patients in the placebo arm and 92.2% in the atezolizumab arm remaining event-free at 3 years, corresponding to an absolute benefit of 15.1% (HR, 0.34 (95% CI, 0.16–0.69), *P* = 0.002) (Extended Data Fig. 2b).

### Molecular heterogeneity of TNBC subtypes—comparison with TILs

**Fig. 4:**
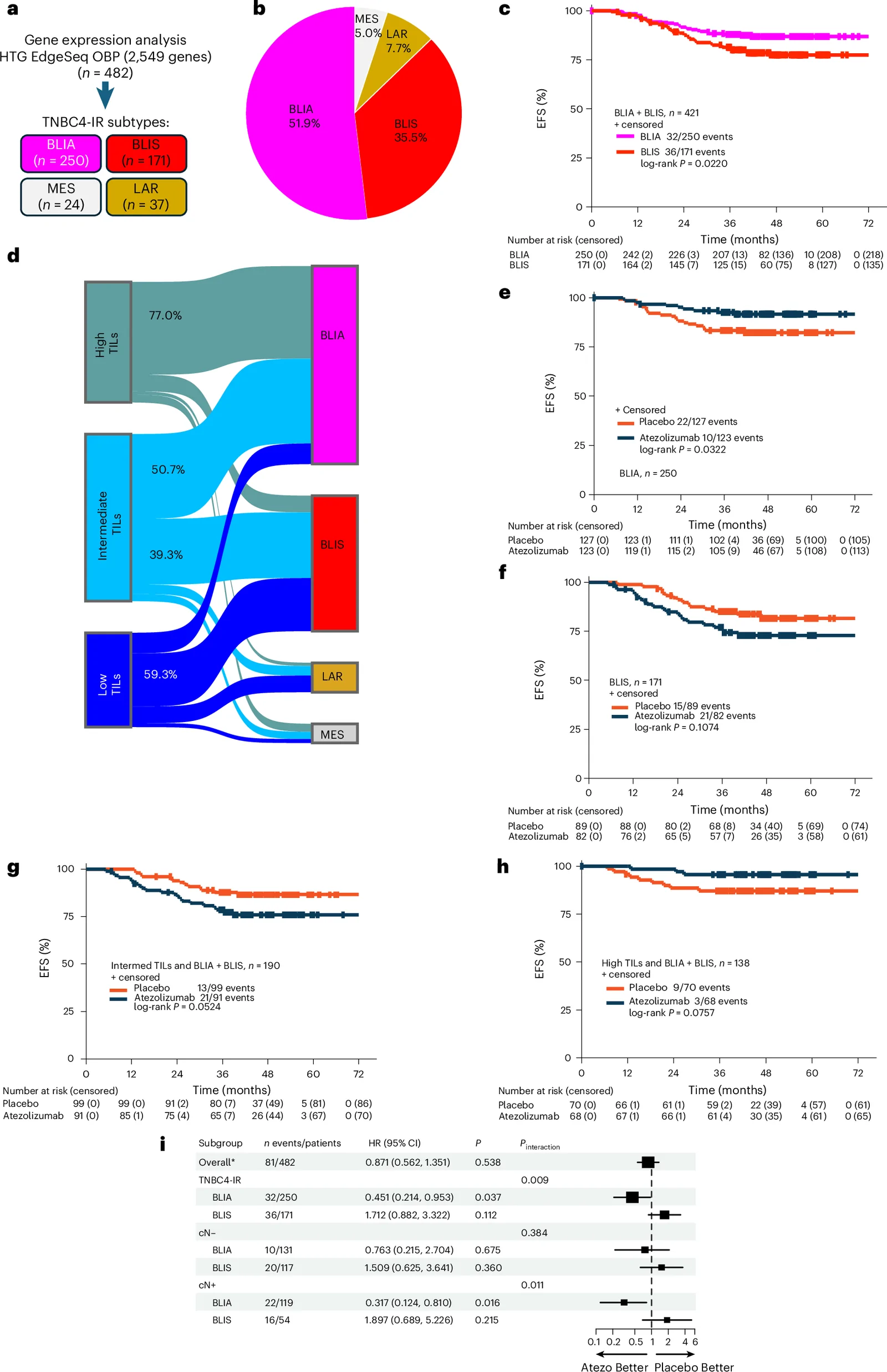
Molecular subtyping of TNBC in the biomarker cohort of 482 patients with the modified TNBC4-IR subtyping. **a**, A total of 482 tumor samples were analyzed and evaluated using the modified TNBC4-IR classification, resulting in four defined molecular subtypes: BLIA, BLIS, LAR and MES. **b**, Distribution of the four TNBC subtypes in the biomarker cohort; the majority of patients had a tumor with either a BLIA or a BLIS subtype. **c**, Survival analysis shows a significantly improved prognosis of patients with immune-activated BLIA tumors (*P* = 0.02, log-rank test). **d**, Comparison of TIL subgroups and TNBC4-IR subtypes. Tumors with high TILs are predominantly BLIA tumors, while tumors with intermediate TILs have a heterogenous subtype distribution. **e**,**f**, Survival analysis of BLIA and BLIS tumors in the two therapy arms. An improved prognosis with atezolizumab is observed in the subgroup of patients with BLIA tumors (**e**, *P* = 0.03), while no significant treatment effect is observed in the BLIS subgroup (**f**). **g**,**h**, Survival analysis focused on the combined BLIA and BLIS groups, stratified by TIL levels and therapy arm. For patients with intermediate TILs, there is no benefit from atezolizumab (**g**), the benefit is observed only in the high-TIL group (**h**). **i**, Forest plot of EFS in the BLIA and BLIS subtypes; squares are point estimates for hazard ratio atezolizumab versus the placebo (the size of the square represents the group size); a horizontal line passing through each square represents the 95% CI.

Patients recruited in Germany and Spain were included in a sub-study that included the evaluation of gene expression of 2,549 genes in their diagnostic core biopsies using the HTG oncology biomarker panel (OBP; HTG Molecular Diagnostics). To explore the potential benefit of atezolizumab in different TIL subgroups, we performed exploratory post hoc mRNA-based investigations in 494 pre-therapy samples from patients enrolled in the sub-study. A total of 12 samples could not be successfully measured, resulting in a cohort of 482 samples for biomarker evaluation (Fig. 4a). The characteristics and outcomes of this biomarker cohort were not substantially different from those of the overall study cohort (Supplementary Table 5). The hypothesis addressed by the translational study was that different TNBC subtypes would respond differently to neoadjuvant ICI. In addition, we assessed predefined immune-, proliferation- and stroma-related gene sets that were originally defined in the GeparNuevo trial^9^ and applied these signatures to this translational cohort. We then compared their distribution and relative proportions across TNBC subtypes in the present analysis. We also evaluated the endpoints of pCR and EFS as well as genes and gene sets that were differentially regulated.

The original four-subtype TNBC (TNBC4) subtypes previously described^10^ were based on an 80-gene signature that had been identified in a cohort of 198 TNBC tumors using the Affymetrix U133 gene expression array. This gene signature has been subsequently extended to a larger set of differentially expressed genes^11^. On the basis of this extended gene set, we developed a modified approach for TNBC subtyping in clinical immunotherapy response trials (TNBC4-IR, a modified version of the four-subtype TNBC classification for clinical immunotherapy trials); details are described in the Methods section. This modified TNBC4-IR subtyping identified four molecularly defined subtypes: basal-like immune-activated (BLIA), basal-like immunosuppressed (BLIS), luminal androgen receptor (LAR) and mesenchymal (MES; Fig. 4a). The individual names of these four subtypes are identical to the original Burstein subtypes, but underlying gene signatures have been adapted for use in gene expression datasets from immunotherapy studies. As shown in Fig. 4b, the TNBC4-IR classification identified two main subtypes: BLIA (51.9%) and BLIS (35.5%), as well as two small groups: LAR (7.7%) and MES (5.0%). In the complete cohort, there was significantly improved EFS for patients with BLIA tumors compared with patients with BLIS tumors (*P* = 0.02, *n* = 421; Fig. 4c).

**Extended Data Fig. 3:**
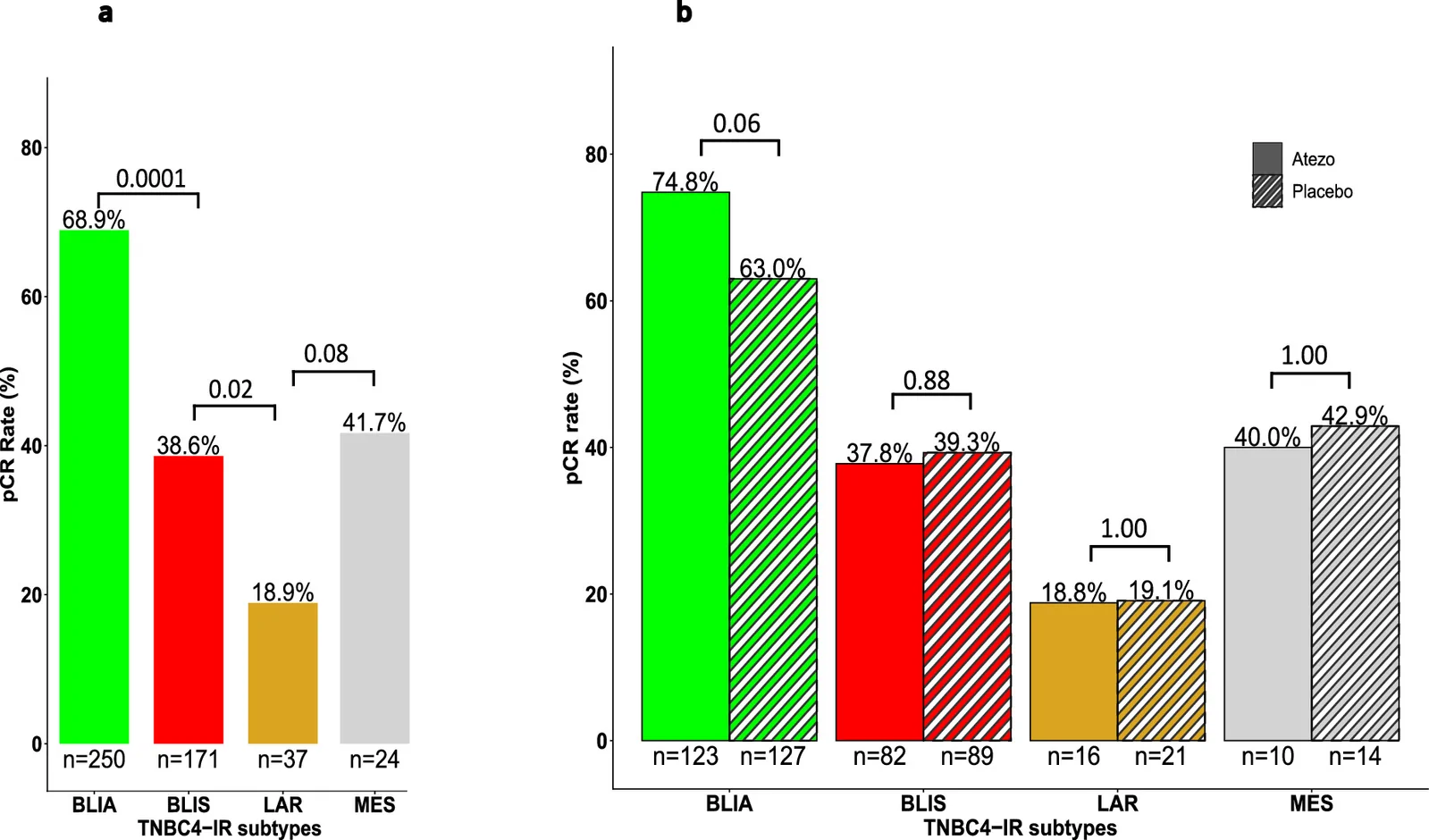
Evaluation of pCR rate in TNBC4-IR subtypes. **a**: Significant differences in pCR rate between the individual subtypes, with increased pCR rates for BLIA tumors. **b**: Comparison of pCR rate in TNBC4-IR subtypes with or without Atezolizumab. A numerical non-significant increase is observed in the Atezolizumab arm for BLIA tumors, no differences are observed for the other subtypes. The bar plots show the proportion of patients with pCR in each TNBC4-IR subtype, with sample sizes indicated below the bars. Statistical significance was assessed using two‑sided Fisher’s exact tests, and *P*‑values are shown in the panel.

**Extended Data Fig. 4:**
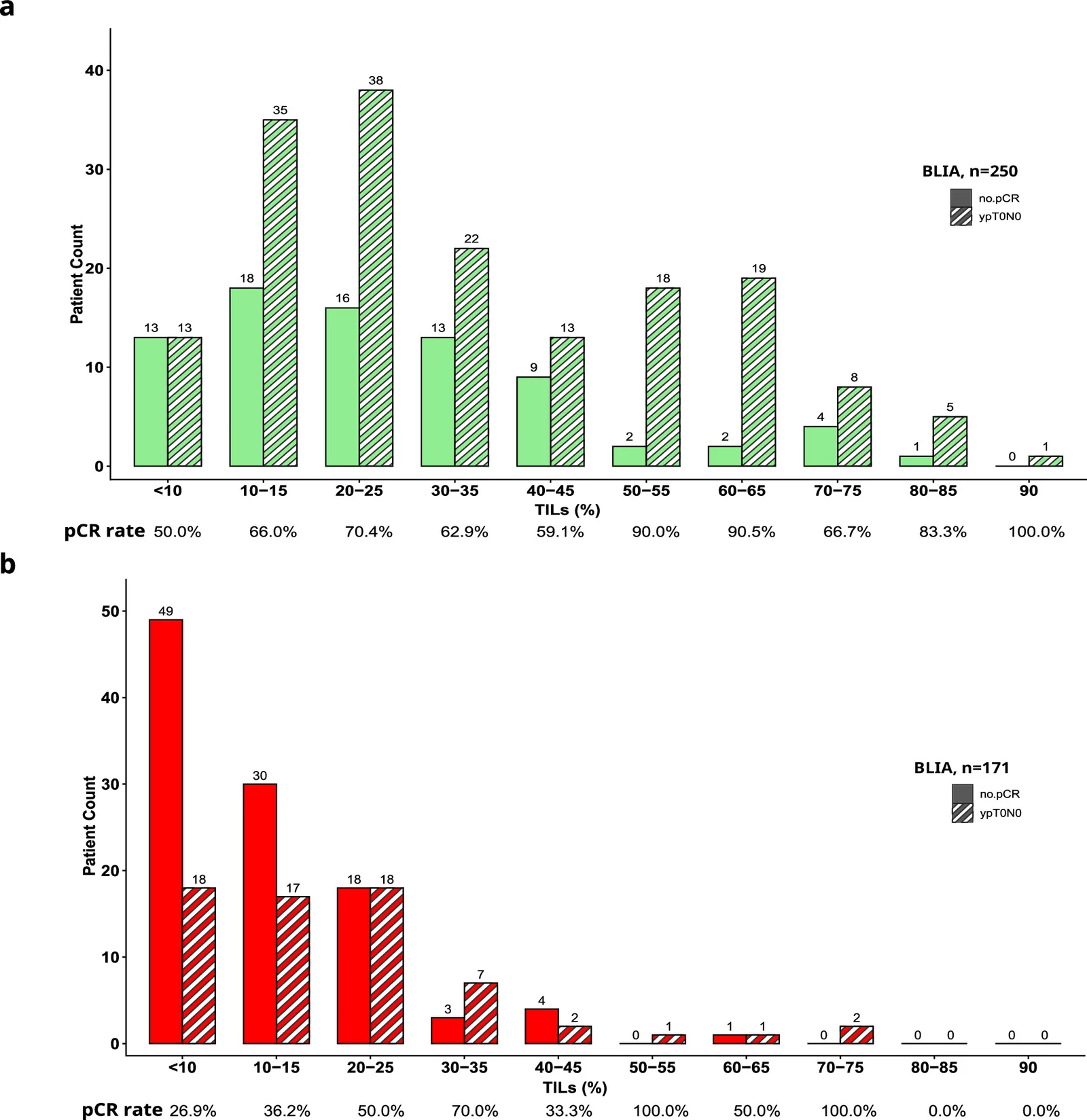
Clustered bar plot showing the distribution of patients in HTG cohort for TNBC4 subtypes. **a**: Clustered bar plot showing the distribution of patients in HTG cohort for TNBC4-IR subtype BLIA with and without pCR based on TIL groups. Bars represent patient counts for pCR and no-pCR subgroups stratified by TILs. The pCR rate of each subgroup is shown below the x-axis. **b**: Clustered bar plot showing the distribution of patients in HTG cohort for TNBC4 subtype BLIS with and without pCR based on TILs. Bars represent patient counts for pCR and no-pCR subgroups stratified by TILs. The pCR rate of each subgroup is shown below the x-axis.

Patients with BLIA tumors had a pCR rate of 68.9%, while patients with BLIS tumors had a pCR rate of 38.6% (*P* = 0.0001; Extended Data Fig. 3a). In the BLIA cohort, a significantly improved EFS was observed in patients receiving atezolizumab compared with placebo (log-rank *P* = 0.03, *n* = 250; Fig. 4e), while no significant difference was observed for patients with TNBC4-IR BLIS tumors (log-rank *P* = 0.11, *n* = 171; Fig. 4f). Only the BLIA subtype showed a numerically increased pCR rate with atezolizumab (74.8% versus 63.0% in the placebo arm, *P* = 0.06; Extended Data Fig. 3b). An overview of the pCR rates in BLIA and BLIS tumors with different TIL levels is given in Extended Data Fig. 4.

For a better understanding of the heterogeneity of the subgroup of tumors with intermediate TILs, we evaluated the distribution of TNBC4-IR subtypes in the different TIL-related subgroups. As shown in Fig. 4d, the intermediate-TIL subgroup consisted of a heterogenous mixture of BLIA (50.7%) and BLIS tumors (39.3%). By contrast, 77.0% of the tumors with high TILs had a BLIA subtype. Tumors with low TILs had a non-BLIA subtype in 78.2% of patients (56.3% BLIS, 17.7% LAR and 4.2% MES). Taking BLIA and BLIS tumors together, we observed a positive treatment effect for atezolizumab in patients with high TILs, but not for patients with intermediate TIL levels, in which atezolizumab seems to be outperformed by placebo (Fig. 4g–i).

**Extended Data Fig. 5:**
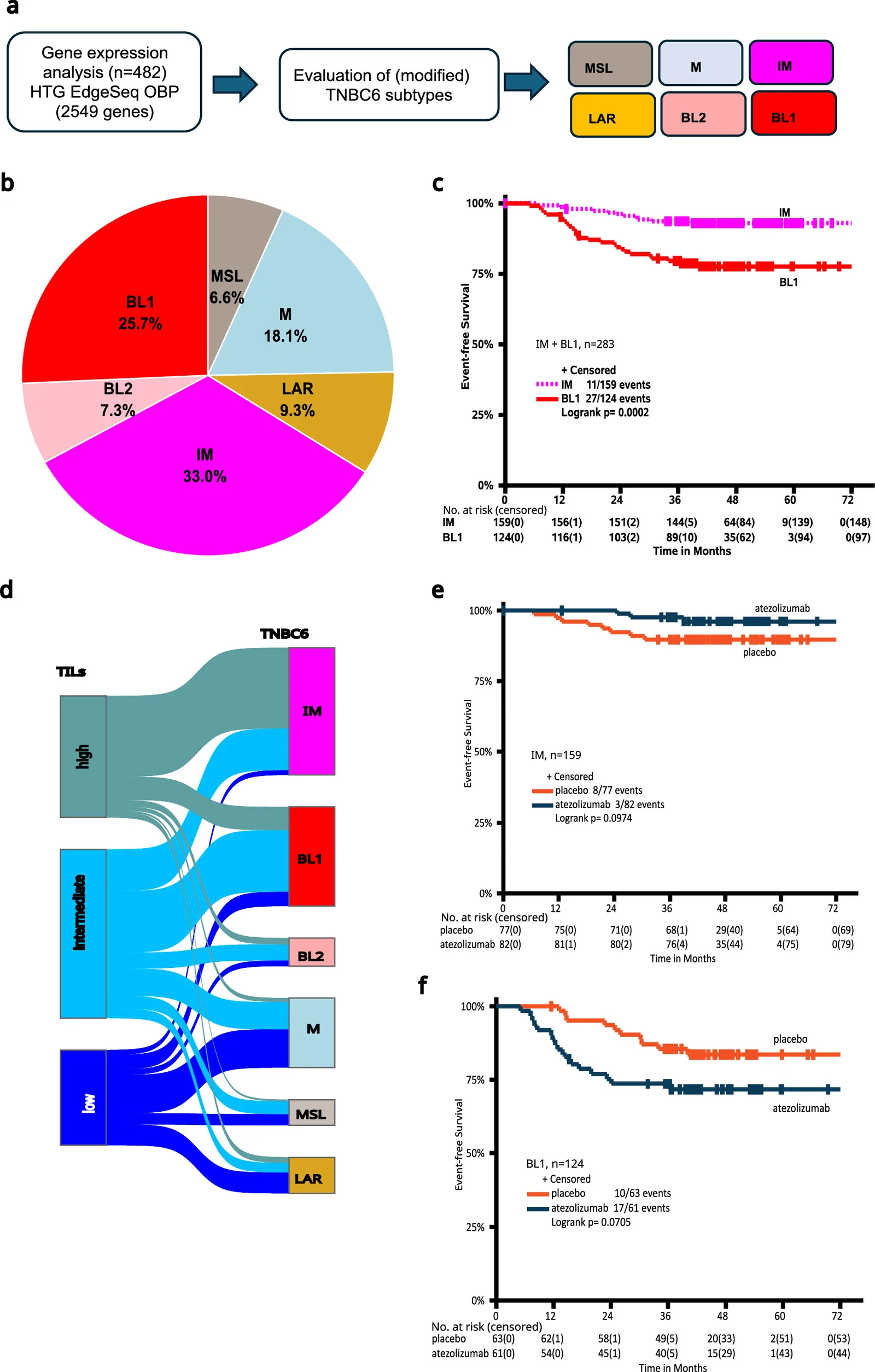
Molecular subtyping of TNBC in 482 patients with the TNBC-6 subtyping approach. **a**: A total of 482 tumor samples were analyzed and evaluated using the TNBC6 classification, resulting in 6 defined molecular subtypes IM, BL1, BL2, MES and M. **b**: Distribution of the 6 TNBC subtypes in the biomarker cohort, the main groups were the IM, the BL1 and the M subtype. **c**: Survival analysis shows a significantly improved prognosis of patients with immune-activated IM tumors compared to BL1 tumors (p = 0.0002, log rank test). **d**: Comparison of TIL subgroups and TNBC6 subtypes, Tumors with high TILs are predominantly IM tumors, while tumors with intermediate TILs have a heterogenous subtype distribution. e,f: Survival analysis of IM and BL1 tumors in the two therapy arms.

**Extended Data Fig. 6:**
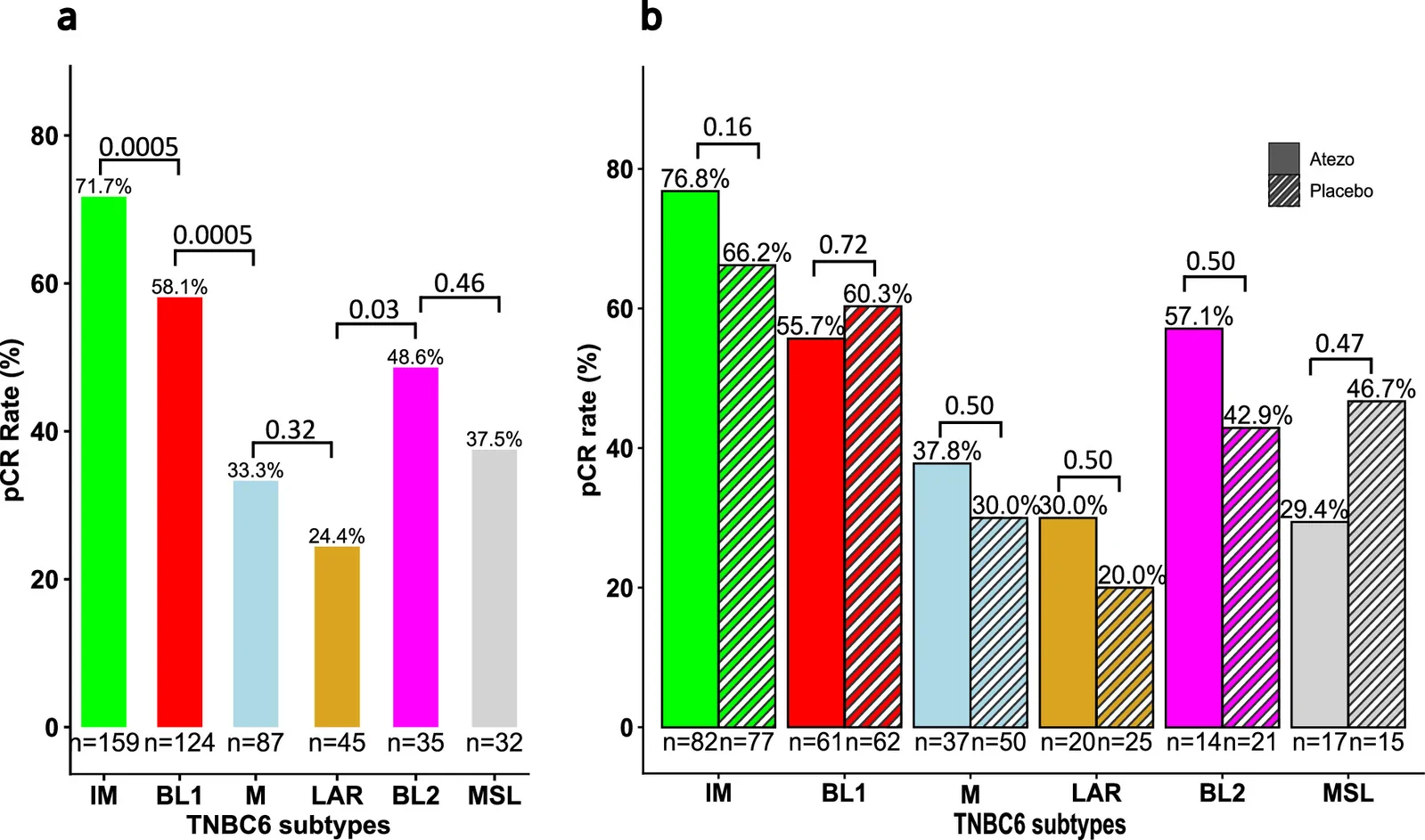
Subgroup analysis for TNBC6 for pCR. **a**: Significant differences in pCR rate between the individual TNBC6 subtypes, with increased pCR rates for IM and BL1 tumors. **b**: Comparison of pCR rate in TNBC6 subtypes with or without atezolizumab. The bar plots show the proportion of patients achieving pCR in each TNBC6 subtype, with sample sizes indicated below the bars. Statistical significance was assessed using two‑sided Fisher’s exact tests, and *P*‑values are shown in the panel.

The main focus of the biomarker investigation is the TNBC4-IR subtyping. As an additional validation, we extended our analysis to the TNBC6 subtyping, originally described in a previous study^12^. This analysis also showed differences of TNBC subtypes in the overall pCR rate and EFS as well as a heterogenous distribution of TNBC6 subtypes in tumors with intermediate TILs (Extended Data Figs. 5 and 6).

### Heterogeneity of the tumor microenvironment on the gene expression level

**Extended Data Fig. 7:**
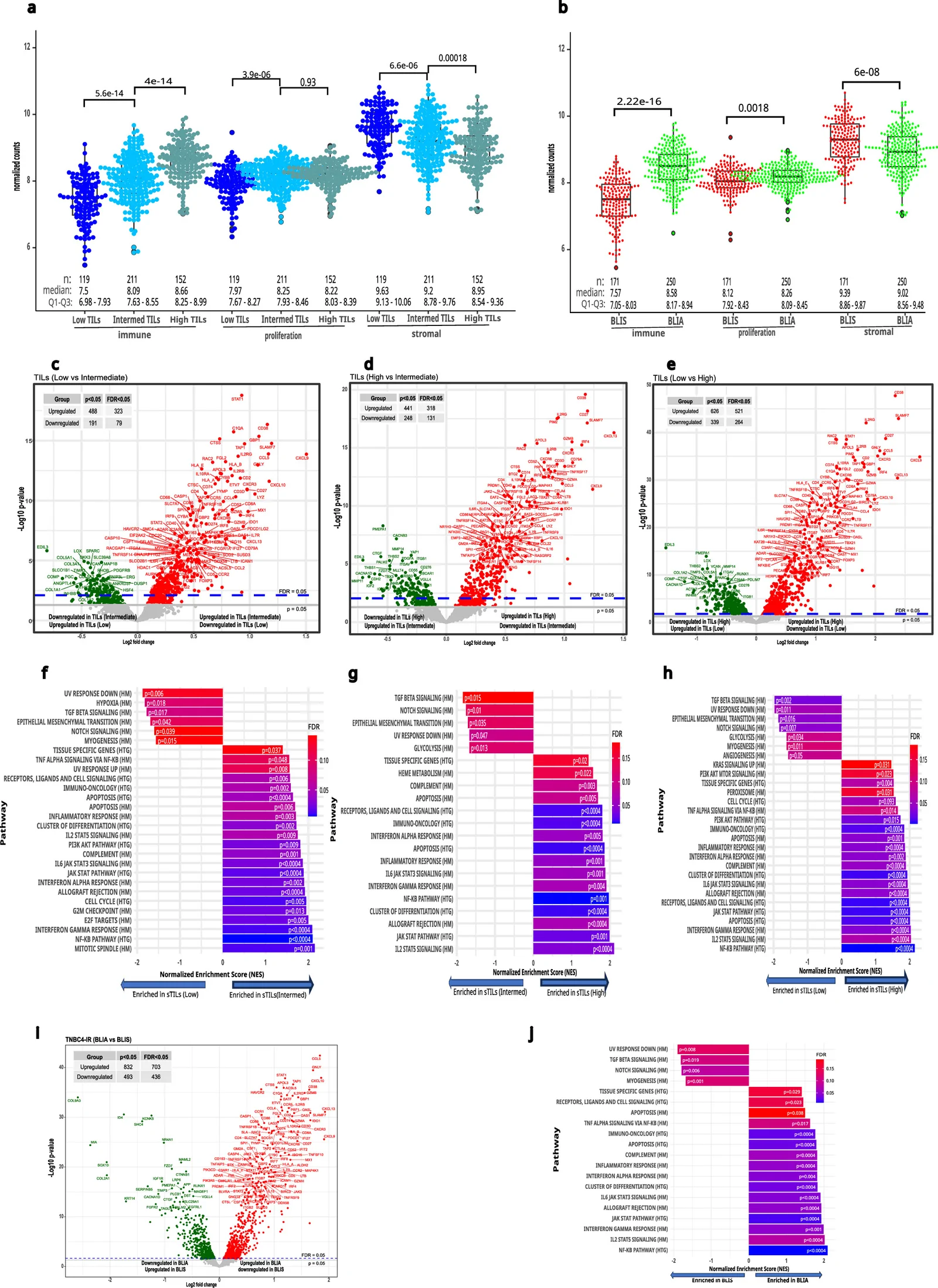
Gene expression analysis in TIL-related subgroups as well as BLIA and BLIS molecular subtypes. **a**: Evaluation of three predefined signatures generated in the GeparNuevo cohort for immune, proliferation and stromal genes in the three different TIL groups (low, intermediate, high). An increased immune gene expression and a reduced stromal gene expression is observed with increased TILs, while the proliferation-related genes show only minor differences. **b**: Evaluation of the GeparNuevo signatures in BLIA and BLIS tumors show a similar pattern as the three TIL groups. The box plots (a, b) show the median, first and third quartiles, and the minimum and maximum values within 1.5x the IQR. Statistical significance was determined using a two-sided Wilcoxon rank-sum test and the exact *P*-values are displayed directly in the figure panel. **c–e**: Differential gene expression in the three TIL groups (c: low vs. intermediate TILs; d: intermediate vs. high TILs, e: low vsl high TILs). **f–h**: Gene set enrichment analysis (GSEA) in the three TIL groups (f: low vs. intermediate TILs; g: intermediate vs high TILs, h: low vs. high TILs). **i, j**: Comparison of BLIA and BLIS tumors based in differential gene expression (i) and GSEA (j). The p-values for the volcano plots (c-e, i) were obtained using limma moderated t-tests, adjusted using the Benjamini–Hochberg false discovery rate (FDR).

In the previous translational program of the neoadjuvant GeparNuevo (G9) trial, we had identified three G9 signatures relevant for response to neoadjuvant immunochemotherapy^9^. As shown in Extended Data Fig. 7a,b, in the NSABP B-59/GeparDouze biomarker cohort, these gene signatures were differentially expressed across the three TIL subgroups as well as the TNBC4-IR subtypes. BLIA tumors as well as tumors with high TILs had increased levels of the G9 immune signature and reduced levels of the G9-stromal signature. For the G9 proliferation signature, only minor differences were observed between the different TNBC subtypes and TIL-related subgroups.

We performed an analysis of differential gene expression and a gene set enrichment analysis (GSEA) in different TIL subgroups as well as the BLIA and BLIS subgroups. This analysis showed a significant enrichment of immune-related pathways and genes in tumors with high TILs (Extended Data Fig. 7c–h). By contrast, stromal gene expression, including the transforming growth factor (TGF)-beta pathway, was significantly higher in tumors with low TILs. There were also considerable differences in gene expression in the different TNBC subtypes, suggesting relevant differences in cellular interactions in the tumor microenvironment. The BLIA and BLIS subtypes showed inverse expression patterns of immune genes, which are overexpressed in the BLIA subtype and reduced in the BLIS subtype (Extended Data Fig. 7i). In parallel, stromal biomarkers including the TGF-beta pathway were increased in the BLIS subtype and decreased in the BLIA subtype (Extended Data Fig. 7j). This suggests that the balance between immune activation and stromal organization in the tumor microenvironment is relevant for the response to immunotherapy.

**Extended Data Fig. 8:**
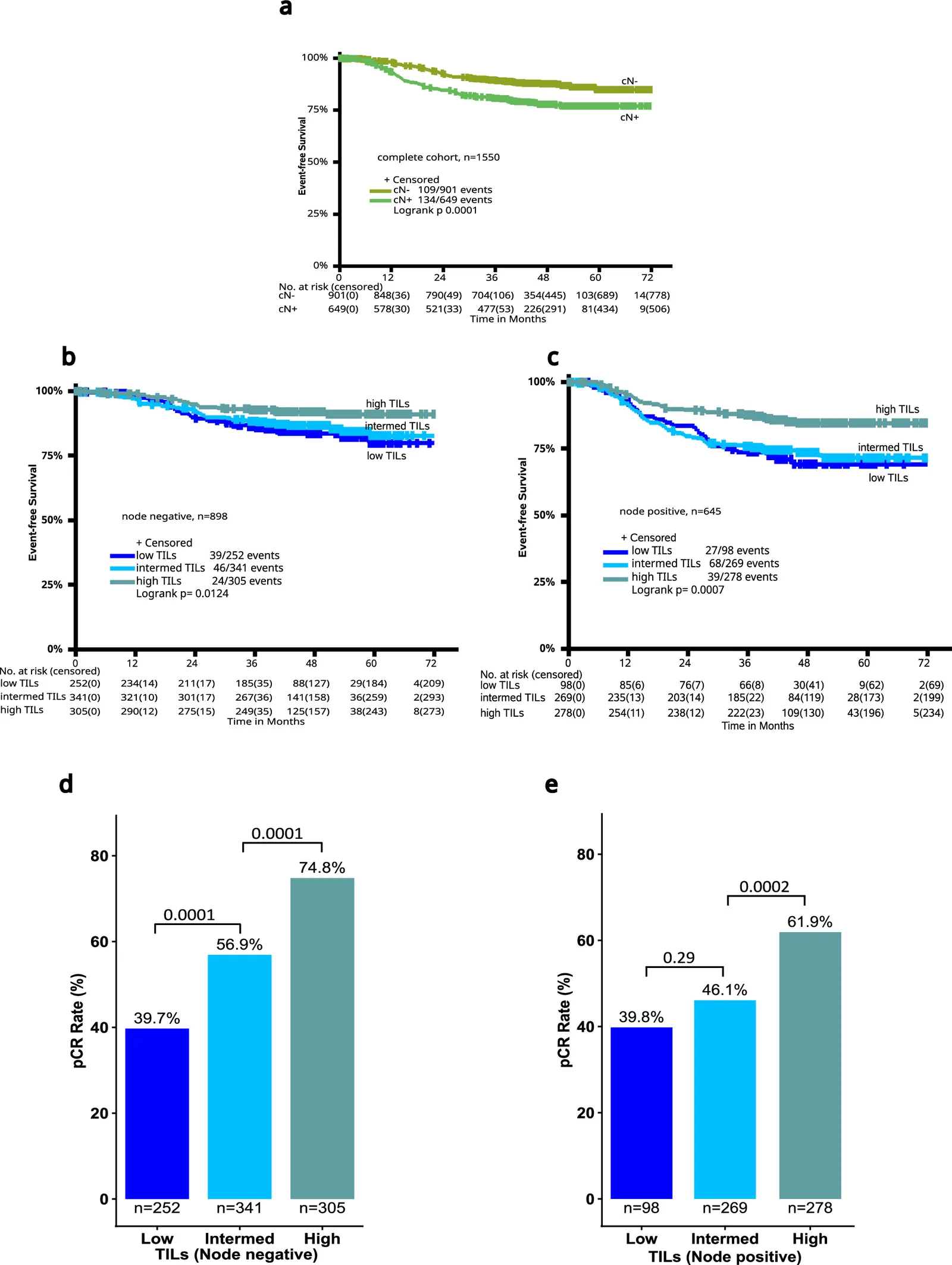
Evaluation of TILs in nodal-positive and nodal-negative disease. **a**: Kaplan-Meier analysis for nodal status with reduced EFS in nodal-positive tumors. **b**: TILs are significant for EFS in nodal-negative tumors. **c**: TILs are significant for EFS in nodal-positive tumors. **d-e**: Bar plots showing the proportion of node negative or node positive patients achieving pCR in each TILs subgroup, with sample sizes indicated below the bars. Statistical significance was assessed using two‑sided Fisher’s exact tests, and *P*‑values are shown in the panel. The plots indicates an increased pCR rate in node negative or node positive patients with high TILs.

In an additional exploratory subgroup analysis, we evaluated TILs as well as TNBC4-IR subtypes in patient subgroups stratified by nodal status, based on the clinical observation that high TILs and positive nodal status determine subgroups with a significant atezolizumab effect. The effect of TILs on EFS and pCR was comparable among nodal-positive and nodal-negative tumors (Extended Data Fig. 8).

**Extended Data Fig. 9:**
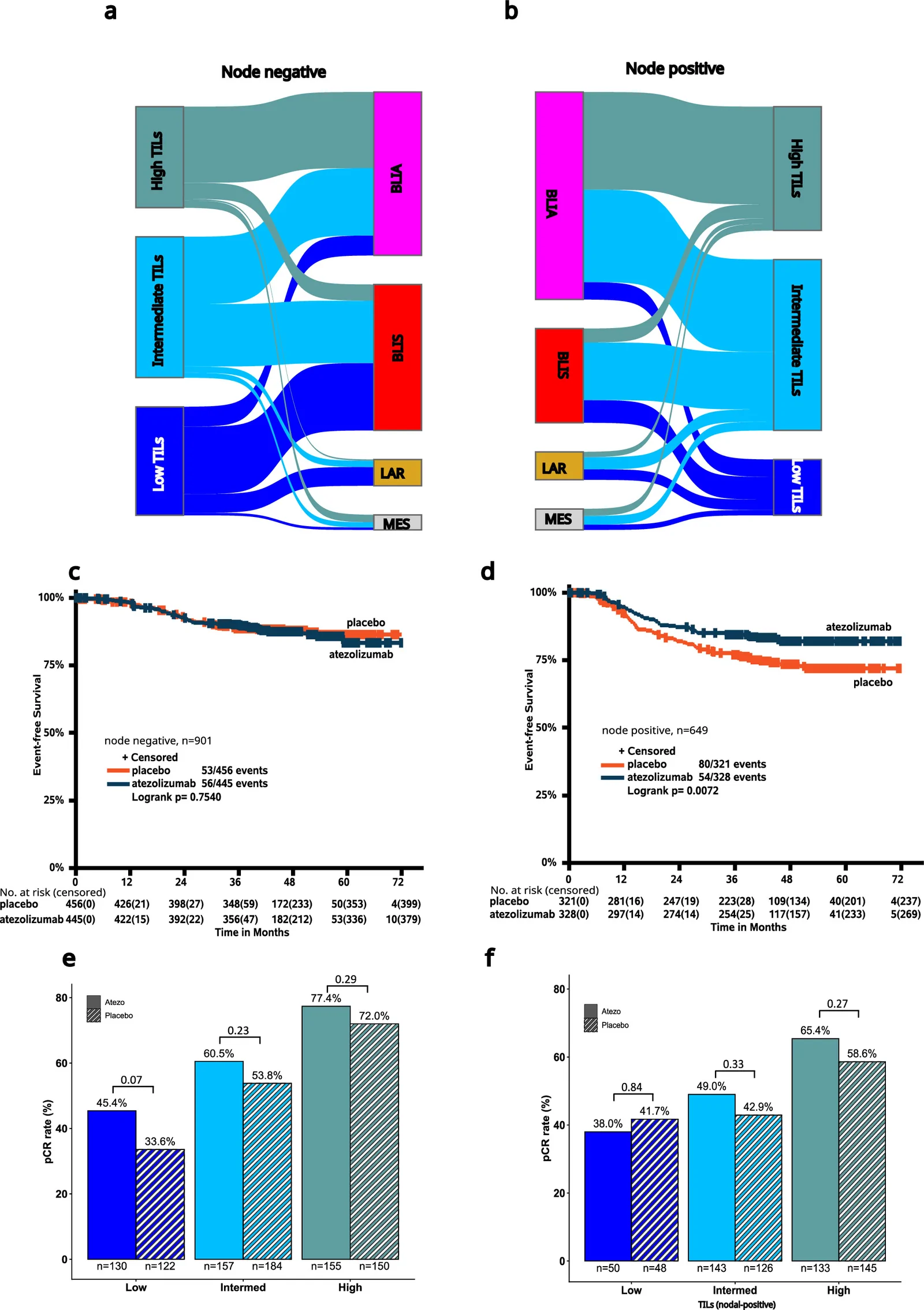
Comparison of nodal-positive and nodal-negative tumors. **a,b**: Comparison of TIL subgroups and TNBC4-IR subtypes in nodal-positive (A) and nodal-negative (B) tumors. Nodal-positive tumors have more high TILs and BLIA subtype compared to nodal-negative tumors. **c**: No effect of atezolizumab on EFS in nodal-negative tumors (complete cohort). **d**: significant effect of Atezolizumab on EFS in nodal-positive tumors (complete cohort). **e,f**: pCR stratified by treatment arm and TILs in nodal-negative (E) and nodal-positive (F) tumors (complete cohort). No significant treatment effect was observed in either subgroup.

**Extended Data Fig. 10:**
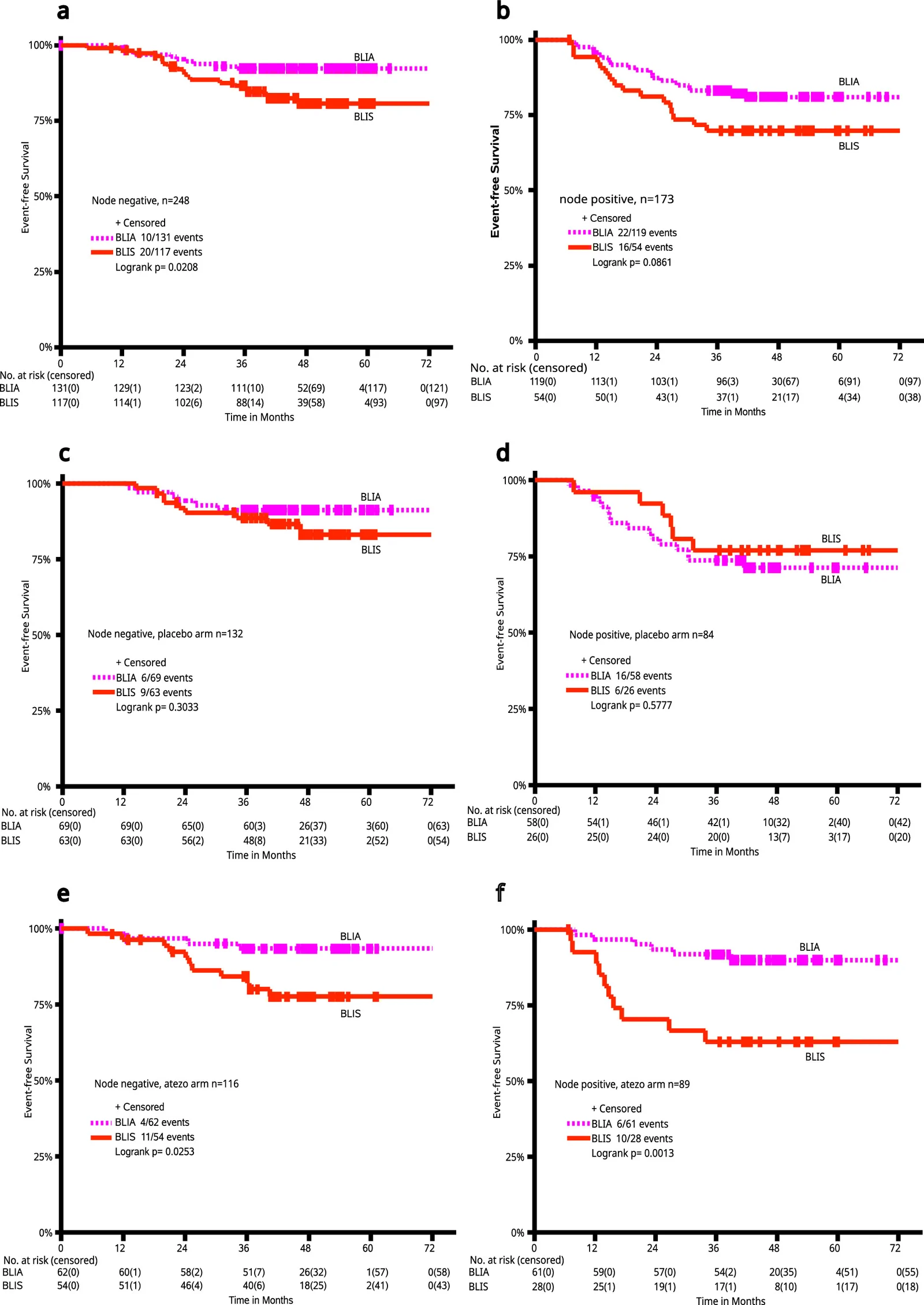
Comparison of BLIA and BLIS tumors in nodal-positive and nodal-negative tumors stratified by therapy arm. **a**: Significant differences in EFS for BLIA vs. BLIS tumors in nodal-negative disease **b**: Non-significant differences in EFS for BLIA vs. BLIS tumors in nodal-positive disease. **c**: No difference in EFS for BLIA vs. BLIS tumors in nodal-negative disease in the placebo arm. **d**: No difference in EFS for BLIA vs. BLIS tumors in nodal-positive disease in the placebo arm. **e**: Significant difference in EFS for BLIA vs. BLIS tumors in nodal-negative disease in the atezolizumab arm. **f**: Highly significant difference in EFS for BLIA vs. BLIS tumors in nodal-positive disease in the atezolizumab arm.

Interestingly, the distribution of TILs as well as TNBC4-IR subtypes was different in the nodal-positive and the nodal-negative subsets of patients in the biomarker cohort. As shown in Extended Data Fig. 9, in the nodal-positive subgroup, there were more tumors with high TILs (35.3% versus 28.8%) and more tumors with intermediate TILs (48.8% versus 40.2%) than in the node-negative group. By contrast, in the node-positive subgroup, only 15.9% of patients had low TILs, compared with 31% in the nodal-negative subset. In the nodal-positive subgroup, 59.2% of patients had a BLIA subtype, compared with only 46.6% in the nodal-negative subgroup. Therefore, the higher rate of immune-activated tumors in the nodal-positive subgroup could explain the increased effect of atezolizumab in this subgroup. The KM analysis of different subtypes in nodal-positive and negative tumors is shown in Extended Data Fig. 10.

## Discussion

The NSABP B-59/GeparDouze study investigated the addition of atezolizumab to a standard taxane–carboplatin–anthracycline-based NACT regimen. At the definitive analysis of the primary endpoint, the 4-year EFS was 85.2% with atezolizumab and 81.9% with placebo (stratified HR, 0.800 (95% CI, 0.621–1.030); stratified log-rank test *P* = 0.083), which did not meet the prespecified boundary for statistical significance and claiming superiority. Exploratory prespecified subgroup analyses of EFS showed heterogeneity across subgroups, suggesting potential benefit of atezolizumab in subgroups such as those with nodal involvement at presentation, those with primary tumors >3 cm and those with tumor TILs ≥30%. Exploratory mRNA-based subtyping also suggested an atezolizumab effect in patients whose tumors were TIL enriched and of the BLIA subtype, while patients whose tumors had intermediate TIL levels showed heterogeneity of the tumor immune microenvironment with no suggestion of atezolizumab benefit.

The result of the secondary endpoint of DDFS was similar, with a 4-year DDFS rate of 87.2% with atezolizumab and 85.2% with placebo (HR, 0.834 (95% CI, 0.633–1.098)). OS analysis was immature, but the 4-year OS was favorable in both arms, 90.2% versus 89.5%, respectively (HR, 0.86 (95% CI, 0.62–1.19)).

The absolute increase in the secondary endpoint of the pCR rate of 6.3% (57% with placebo to 63.3% with atezolizumab) is in line with previously reported data^3–5,8,13^. Notably, the pCR rate in the NACT-placebo group is one of the highest reported without ICI therapy and patients who experience pCR have a favorable outcome regardless of ICI therapy.

In contrast to KEYNOTE-522, our study did not show a significant improvement in EFS and OS by adding a PD-L1 inhibitor to an optimal NACT regimen. There are important differences between the two trials. Firstly, the ICI investigated in both trials is different—the PD-1 inhibitor pembrolizumab was used in KEYNOTE-522 while the PD-L1 inhibitor atezolizumab was used in this trial. Secondly, two-thirds of the patients in this trial received dose-dense EC or AC, which was not allowed in KEYNOTE-522, and 50% of the patients with non-pCR in the NSABP B-59/GeparDouze trial received capecitabine as adjuvant therapy. Finally, the overall risk profile accrued to the respective trials appeared to be lower in the NSABP B-59/GeparDouze study. For instance, clinically node-positive patients comprised 51% of the KEYNOTE-522 population compared with 42% in this study. Non-pCR patients receiving placebo and no capecitabine in NSABP B-59/GeparDouze had a 3-year EFS rate of 70.2% compared with 56.8% in KEYNOTE-522. In general, a lower-risk population has a lower event rate, and therefore, it is difficult to detect the same benefit on a statistically significant level.

The description of TILs as a marker for increased pCR rates after neoadjuvant therapy and as a prognostic marker for survival outcome has been validated in many previous studies^14–19^. The NSABP B-59/GeparDouze trial incorporated prospective central evaluation of TILs in randomized patients by trained pathologists at the central pathology sites for the study groups NSABP and GBG following the standardized recommendations of the International TILs Working Group^18^, to evaluate the specific role of TILs as a potential marker of immunotherapy benefit^20^. Our study confirms the prognostic role of TILs as well as a marker for an increased pCR rate in tumors with higher TILs. Exploratory analysis also suggested that patients’ high-TIL tumors (defined as ≥30%) may have had a favorable effect on EFS with atezolizumab compared with placebo. This suggests that high levels of TILs might be able to identify a subgroup of patients with increased benefit from ICI in early TNBC. Our parallel gene expression analysis shows that tumors with high TIL levels consist predominantly of the BLIA subtype, with an increased expression of many immune-related genes.

In contrast to this high-TIL group, the potential clinical utility of TIL assessment is currently limited in tumors with intermediate TILs. Our gene expression analysis shows considerable heterogeneity in this subgroup, with a mixture of BLIS and BLIA subtypes. Consequently, no difference between the atezolizumab arm and the placebo arm was observed in this subgroup with intermediate TILs. Therefore, it might be useful to use other approaches, in particular gene expression-based subtyping, for patients with tumors with intermediate TILs. Interestingly, this biomarker approach would be very similar to the situation for proliferation markers and gene expression testing in luminal breast cancer: on the basis of the recommendation of the International Ki67 in Breast Cancer Working Group, Ki-67 values of 30% or more or of 5% or less could be used for chemotherapy-related therapy decisions, while the intermediate range of Ki-67 between 5% and 30% would require additional gene expression analysis^21^.

In summary, integration of quantifying TILs with RNA-based subtyping to identify the immune-activated BLIA subtype and the high-TIL group identified cohorts with apparent improved outcomes with atezolizumab, suggesting that a pretherapeutic immunologically ‘hot’ microenvironment might be an important predictor of benefit.

It should also be emphasized that, in addition to the observed differences in benefit of atezolizumab, there were large differences in neoadjuvant response and survival in the different molecular subtypes of TNBC, observed with both the TNBC4-IR and the TNBC6 approaches in a remarkably similar pattern. This suggests that it might be time to remove TNBC as a ‘title of convenience’^22^ from breast cancer classification systems and to replace it with a standardized biological classification based on gene expression profiling. This might be essential for the next generation of clinical trials addressing the molecular diversity of the tumor microenvironment in the former TNBC subtype. Our gene expression analysis shows a consistent activation of pathways related to stromal reorganization such as the TGF-beta pathway in tumors with low TILs as well as in the BLIS subtype. The therapeutic interaction with this pathway might provide options to improve response to immunotherapy in this critical subtype.

In our exploratory analysis of node-positive and node-negative tumors, we observed higher TIL levels and more immune-activated tumors (BLIA subtype) in the node-positive subgroup. This observation could explain the apparent differential effect of atezolizumab by nodal status in NSABP B-59/GeparDouze. While there are relevant statistical limitations for these exploratory subgroup comparisons, our observations raise the interesting immunological hypothesis whether the interaction between tumor cells and immune cells in the lymph node tissue might be relevant for immunotherapy response as an additional systemic activation of the immune system.

Nodal status could reflect differences in antigen exposure or priming in the tumor-draining lymph nodes. In a node-positive tumor, the tumor cells have direct contact with the systemic immune system, and there might be additional immune cells entering the tumor microenvironment that have been stimulated against the tumor cells in the lymph node. Therefore, in the node-positive situation, the immune system has more options to replenish exhausted immune cells in the tumor microenvironment, resulting in a more activated immune system and a higher response rate to atezolizumab. This could be particularly relevant in subgroups with heterogenous immune status, such as the intermediate-TIL group, which might include tumors with an exhausted immune system. In this exhausted milieu, TILs may be present, but are not functional against the tumor cells, and might not be activated by ICI treatment. It would be of interest to investigate this hypothesis in additional cohorts and prospective translational and preclinical investigations.

There are limitations relevant to this translational analysis: we have only been able to analyze the gene expression in a subcohort of 482 patients, and even in this comparably large subset, there are smaller subgroups that limit the statistical analyses and the interpretation. We therefore focused our analyses on the most predominant subtypes. Due to the use of the HTG platform for mRNA analysis, we also had to adjust the approaches for determination of the subtypes to the 2,549 genes that are included in the HTG OBP panel. It would be very interesting to evaluate the TNBC4-IR subtypes in additional clinical trial cohorts, including trials with different types of immune-checkpoint inhibitors.

In conclusion, the NSABP B-59/GeparDouze study did not show a statistically significant improvement in EFS with atezolizumab, although a significant but small increase in pCR was shown. Exploratory studies suggested potential benefit from atezolizumab in patients with the immune-activated BLIA subtype and in those with high TILs and nodal-positive disease. These findings highlight the potential role of an activated tumor immune microenvironment in predicting a therapeutic benefit with atezolizumab and potentially other ICIs in TNBC.

## Methods

### Ethical approval and consent

The trial complied with good clinical practice guidelines and the Declaration of Helsinki. The study protocol and amendments were approved by the competent authorities and independent ethics committees, which are listed in Supplementary Information 1. An independent data monitoring committee oversaw the study. The patients provided written informed consent. There was no compensation for participants.

### Trial design

NSABP B-59/GeparDouze was a multicenter, multinational, prospectively randomized, double-blind placebo-controlled phase 3 trial (NCT03281954) which investigated whether the addition of atezolizumab to NACT followed by adjuvant atezolizumab to complete 1 year of therapy improved EFS in patients with early centrally confirmed TNBC designed by the NSABP, GBG and Investigational Drug Development Institute (IDDI) and funded by grants from Genentech/Roche to the NSABP Foundation. The academic study was led by NSABP and GBG in collaboration with the Spanish Breast Cancer Group (Grupo Español de Investigación es Cáncer de Mama (GEICAM)), US Oncology Research (USOR) and Translational Research in Oncology (TRIO). The study design is shown in Extended Data Fig. 1. The study protocol, a summary of clinically important protocol amendments, and the statistical plan are provided as Supplementary Information 2–4. No further follow-up phase is planned for the study. This academic-led trial was designed, conducted and analyzed by NSABP and GBG in collaboration with IDDI and Roche/Genentech. GEICAM, TRIO and USOR supported recruitment.

### Patients

Eligible patients had clinical stage II–III early TNBC, defined as estrogen receptor (ER) and progesterone receptor (PgR) status below 1% and negative HER2 status. Central testing for HER2, ER and PgR was required to confirm TNBC. For patients with clinical regional lymph node involvement—defined as cN1 with cytological or histological confirmation, or cN2–N3 with or without a biopsy—the primary breast tumor could be clinically staged as T1c, T2 or T3. For patients with clinically negative nodes, the primary tumor had to be T2 or T3.

Bilateral or multicentric HER2-negative breast cancers were permitted if the highest-risk tumor was ER negative and PgR negative and met stage eligibility criteria. Patients were also required to have a left ventricular ejection fraction >55% and no important cardiac history. Patients were excluded if they had a history of ipsilateral invasive BC or ipsilateral ductal carcinoma in situ, evidence of metastatic disease, prior treatment for the currently diagnosed BC before randomization, or if they had received immunotherapy previously.

Patients were eligible for the study regardless of sex or gender, and sex was determined based on self-report at the time of enrollment. As only one male patient was included in the study, no sex- or gender-based analyses were performed, and study results are reported for the overall cohort. Data were not disaggregated for privacy reasons.

### Randomization and treatment

The study was designed as a double-blind trial; neither the patient nor the investigator knew which treatment had been assigned. At the time of protocol activation, patients were stratified by region (North America versus Europe), tumor size (1.1–3.0 cm versus >3.0 cm), AC or EC schedule (q2w versus q3w) and clinical nodal status (positive versus negative) and then centrally randomized 1:1 using permuted block randomization to receive atezolizumab or placebo (1,200 mg IV every 3 weeks).

PD-L1 status (positive, negative, indeterminate or not available) was added as a 5th stratification factor by amendment number 1 (16 July 2019) after 374 patients had been randomized. PD-L1 status assessment was done centrally using the antibody Ventana PD-L1 (SP142); the immune cell (IC) score was reported as a percentage according to the Ventana evaluation protocol. A PD-L1 positive status was defined as ≥1% staining of tumor-infiltrating immune cells as a percentage of the tumor area using the SP142 Ventana assay (Ventana Medical Systems).

The TIL analysis was performed based on the standardized recommendations of the international TIL working group^18^. The analysis was performed by trained pathologists at the two central pathology sites for NSABP and GBG. The predefined statistical analysis plan included the TIL cutoff of 10% as well as the cutoff of 30%, based on a large analysis of TILs in TNBC^23^.

All patients were to receive weekly paclitaxel 80 mg m^−2^ for 12 doses plus carboplatin AUC of 5 every 3 weeks for 4 doses followed by either doxorubicin 60 mg m^−2^ or epirubicin 90 mg m^−2^ with cyclophosphamide 600 mg m^−2^ (AC or EC) every 2–3 weeks for 4 doses (per investigator’s discretion). After surgery (performed within 6 weeks of the last CT dose), the pathologic response status was assessed by the site pathologists, with an additional centrally blinded evaluation of histopathology reports. Patients who did not discontinue atezolizumab or placebo owing to toxicity during neoadjuvant therapy resumed the original randomized investigational therapy assigned until 1 year after the initial dose of atezolizumab or placebo. Radiotherapy, if indicated, was co-administered with atezolizumab or placebo.

Post-neoadjuvant systemic therapy other than atezolizumab or placebo was not allowed at the initiation of the trial, but results of the CREATE-X^24^ and OlympiA^25^ trials, subsequently changing the standard treatment landscape, required amendments during the accrual phase of the trial to allow administration of adjuvant capecitabine with atezolizumab or placebo for patients with non-pCR status at surgery. Olaparib was allowed for patients with non-pCR status and germline pathogenic variants in *BRCA1/2* but required discontinuation of atezolizumab or placebo.

### Endpoints

The primary endpoint was EFS, defined in accordance with NeoSTEEP^26^ as time from randomization to the occurrence of any of the following events: disease progression on protocol therapy resulting in the initiation of non-protocol cancer therapy or inoperability, local invasive recurrence following mastectomy, local invasive recurrence in the ipsilateral breast after lumpectomy, regional recurrence, distant recurrence, contralateral invasive breast cancer, a second non-breast primary malignancy or death from any cause. EFS provides earlier and clinically relevant assessment of treatment efficacy and is an established endpoint that has supported regulatory decision-making in the neoadjuvant setting.

Secondary endpoints included pCR in the breast and lymph nodes evaluated after surgery (ypT0/Tis ypN0) defined as the absence of any invasive residual tumor in the resected breast specimen and lymph nodes following completion of neoadjuvant therapy; OS, defined as the time from randomization to death due to any reason; DDFS, defined as time from randomization until distant recurrence, death from breast cancer, death from other causes or occurrence of a second primary invasive non-breast cancer; and disease-free survival, defined as the time from first breast surgery to disease recurrence or death from any cause.

Safety by toxicity grades was defined by the National Cancer Institute Common Terminology Criteria for Adverse Events version 4.0. Cardiac safety was evaluated in the first 60 patients randomized and who initiated AC or EC in the cardiac safety lead-in cohort. The evaluation was based on the frequency of elevated troponin-T levels and changes in left ventricular ejection fraction, to identify and further assess potential cardiac toxicity associated with the combination of anthracycline and atezolizumab. Brain metastases-free survival was an exploratory endpoint.

### Study assessments

EFS was evaluated by the investigator in accordance with national guidelines. pCR was assessed by a local pathologist following completion of NACT and confirmed by central assessment of histopathology reports. All adverse events, including SAEs, were monitored throughout the trial and for 30 days after the discontinuation of study treatment. Adverse events of special interest were defined by a protocol-specified list of terms and were classified as adverse events of special interest only if they met the corresponding predefined severity grading criteria. These events were monitored—regardless of their relationship to the study drug—from the first dose through follow-up, until study closure.

### Translational biomarker program

#### Gene expression analysis using the HTG OBP panel

Gene expression was profiled in 494 FFPE tumor tissues using the HTG OBP (HTG Molecular Diagnostics) that measures the expression of 2,549 genes associated with tumor biology. For quality control of HTG measurements, the mean of four negative and four positive internal controls was calculated for each sample. The measurement was repeated for a sample if the mean of its positive controls was below two standard deviations (s.d.s) of the grand mean across all samples or if the mean of its negative controls was above two s.d.s from the grand mean. As additional quality control, samples with less than 1.5 million total counts were excluded from further analysis. The quality-controlled data were normalized using the counts per million (CPM) method and log_2_ transformation. Finally, to improve reproducibility, a modification of the CPM method was applied by introducing a lower bound of 3 on CPM values as reported previously^9^. All subsequent translational analyses involving gene expression and TNBC subtypes were conducted post hoc and are therefore considered exploratory.

### Molecular subtyping of TNBC

#### TNBC6 subtyping

We developed a TNBC6 subtype classification algorithm using the gene signatures previously defined^12,27^. The algorithm was adapted to the HTG OBP, which provided coverage for 1,070 genes overlapping with the published TNBC6 signatures. Our algorithm classified all samples into one of the six TNBC subtypes (BL1, BL2, IM, M, MSL and LAR), and care was taken to avoid assigning samples to an ‘unstable’ category.

Transcriptomic data of these 1,070 overlapping genes from 180 TNBC samples in The Cancer Genome Atlas (TCGA), each with previously reported TNBC6 subtype assignments^27^, were used as the reference set. Gene expression values were log_2_ transformed and standardized using *z*-transformation (mean = 0, s.d. = 1) to ensure comparability across samples. The resulting *z*-scored matrix was used to derive subtype-specific centroids. For each TNBC6 subtype, a centroid was calculated as the mean expression profile of all samples belonging to that subtype in the TCGA dataset. These centroids represent the ‘reference signatures’ against which test samples were compared.

For classification, test samples were *z*-transformed and compared against the TCGA-derived centroids. Correlations between each sample and all centroids were computed using Spearman’s rank correlation coefficient. Each sample was then assigned to the subtype whose centroid showed the strongest correlation with its expression profile ensuring robust classification.

#### TNBC4-IR subtyping

Subtype-specific gene sets were curated from a previous study^11^. Of the 719 upregulated differentially expressed genes originally reported, 217 were represented on the HTG OBP and used in TNBC4-IR subtyping. These curated gene sets served as the reference gene sets for classification. Gene expression data were log_2_ normalized before analysis, without additional scaling or centering. Single-sample gene set variation analysis was performed using the GSVA (v2.2.0) R package with default settings, including normalization^28^. Enrichment scores were calculated for each of the four subtype gene sets (BLIA, BLIS, MES and LAR), and each sample was assigned to the subtype with the highest enrichment score, taking care to avoid the ‘unclassified’ category. The workflow was implemented in R (v4.5.1) with consistent preprocessing and GSVA parameters to ensure reproducibility. Sankey plots were generated using the ggsankey package (v 0.0.99999) for TNBC subtype data for the TIL categories.

### Differential expression and GSEA

The normalized counts were analyzed for differential gene expression using LIMMA (v3.50.3) in R, and volcano plots were drawn for genes that were statistically significant (false discovery rate (FDR) < 0.05). Briefly, for differential gene expression analysis, the normalized data were batch corrected and incorporated into the linear model fitting process using the lmFit function. Contrast matrices were applied to define the comparisons of interest, and the data were analyzed to identify differentially expressed genes. The eBayes function was used to compute *t*-statistics, log odds and Benjamini–Hochberg FDR-adjusted *P* values. All analyses were performed using default parameters unless otherwise specified.

GSEA was performed using the phenotype-based method, testing predefined gene sets for enrichment across chosen group comparisons^29^. For GSEA, the 2,549 genes of the HTG OBP panel were mapped to 25 gene groups and pathways predefined by HTG (HTG gene sets), as well as to the established 50 hallmark gene sets of the Human Molecular Signatures Database (v2023.2) representing well-defined biological states and processes. GSEA was performed in a phenotype-based approach using normalized expression data for 2,549 genes. The analysis used 2,500 permutations, a weighted enrichment statistic and the Signal2Noise metric, with all other parameters set to default. The key metrics of GSEA enriched scores and normalized enrichment scores were scored using default conditions with few modifications (maximum size to exclude larger sets = 750, minimum size to exclude smaller sets = 10) to include all the gene sets for the analysis. The threshold for significant enrichment was defined as a *P* value of <0.05 and an FDR of <0.25. The statistically significant gene sets were shown as dot plots generated in R. The GSEA was carried out using Microsoft Windows-based software v.4.3.2.

### Statistical analysis

Efficacy was evaluated in the intention-to-treat population, which included all patients who had undergone randomization. Safety was evaluated in the as-treated population, which included all patients who had undergone randomization and received at least one dose of the trial drug. The original sample size of 760 patients per arm (total 1,520 patients) was calculated for the log-rank test to provide 80% power to detect a hazard ratio of 0.70 at an overall alpha level of 0.045 with 269 EFS events. Following adjustment based on actual accrual and the decision to eliminate pCR as a co-primary endpoint, the study was redesigned to detect an HR of 0.7 for EFS between the atezolizumab arm and the placebo arm with 252 EFS confirmed events maintaining 80% power at an overall 2-sided alpha level of 0.05. However, due to consistently low monthly event rates in 2024, an amendment of the statistical analysis plan, blinded to treatment allocation, was undertaken to allow definitive analysis to proceed once at least 242 EFS events were confirmed. The resultant power loss of about 2% was considered acceptable as an alternative to waiting for 6 months based on the persistent low monthly event rate to reach 252 events. To preserve the overall level of alpha of 0.05 following the prespecified interim analysis at 196 events, which did not cross the efficacy boundary for superiority of atezolizumab, the nominal level of alpha for the final analysis of the primary endpoint calculated according to the O’Brian–Fleming boundary was 0.0444.

For time-to-event endpoints, the Kaplan–Meyer product-limit method was used to present the endpoints graphically and to report 4-year rates with 95% CI. The primary comparison of EFS (and secondary time-to-event endpoints) between the two treatment arms was analyzed using the stratified log-rank test incorporating the stratification factors. To avoid over-stratification, one stratification factor PD-L1 was dropped according to the predefined strategy (if at least one stratum has fewer than four EFS events, the stratification factor that contains the level with the smallest number of patients had to be removed). The Cox proportional hazards model was used to report the HR with 95% CI between treatment arms, for the whole study population, and (without stratification) in the subgroups. No adjustment for multiple comparisons was performed for the subgroup analyses, including prespecified subgroup analyses, so all subgroup analyses for differential benefit of atezolizumab are (prespecified) exploratory and for the purpose of hypothesis generation. Binary endpoints (pCR) are reported as rates with 95% CI and compared between treatment arms using the Cochran–Mantel–Haenszel test stratified by the same factors as for the primary endpoint. An unstratified continuity-corrected chi-square test was used to compare pCR between arms in prespecified subgroups. Logistic regression was used to calculate odds ratios and 95% CIs; the Breslow–Day test for interaction was used to assess heterogeneity between subgroups; for the TILs in three groups, a logistic regression with an interaction term was used.

Exploratory post hoc analysis of EFS by study treatment with and without capecitabine across the entire accrual and treatment period was conducted in 557 non-pCR patients with a landmark of 6 months to avoid guarantee-time bias. Analyses were also conducted in the subsets of patients entered before and after the amendment, which allowed concurrent administration of capecitabine and study medication.

### Reporting summary

Further information on research design is available in the Nature Portfolio Reporting Summary linked to this article.

## Online content

Any methods, additional references, Nature Portfolio reporting summaries, source data, extended data, supplementary information, acknowledgements, peer review information; details of author contributions and competing interests; and statements of data and code availability are available at https://doi.org/10.1038/s41591-026-04565-6.

## Supplementary information

Supplementary Information Supplementary Tables 1–5, Supplementary Figs. 1 and 2, and Supplementary Information 1–4.

Reporting Summary

## Data Availability

The dataset contains multiple indirect identifiers and sensitive information; therefore, access to these data is controlled and will be shared upon request. Individual participant data that underlie the results reported in this article, after final analysis and publication of all secondary efficacy endpoints, will be shared with researchers who provide translational research proposals. Proposals should be approved by the NSABP B-59/GeparDouze Translational Committee. The study protocol and statistical report may be shared, if necessary, for the project. Proposal forms should be requested from trafo@gbg.de; once the application has been approved and a data transfer agreement has been signed, researchers will be given access to the data within 4 weeks.
