# Supplementary material for: Atezolizumab in early triple-negative breast cancer: the randomized phase 3 NSABP B-59/GeparDouze trial
Source: Nat Med. 2026 Sep 1;32(10):3801–11. doi: 10.1038/s41591-026-04565-6 (PMC13668648; doi:10.1038/s41591-026-04565-6)
Supplement: Supplementary file 1 [file 41591_2026_4565_MOESM1_ESM.pdf]

nature medicine

Supplementary information https://doi.org/10.1038/s41591-026-04565-6

Atezolizumab in early triple-negative breast cancer: the randomized phase 3 NSABP B-59/ GeparDouze trial

In the format provided by the authors and unedited

Supplementary Tables

Supplementary Table 1: Median duration/number of doses before and after surgery among the safety population.

Treatment	**Atezolizumab (n=768)**	**Placebo (n=764)**
Paclitaxel		
Median weeks (range)	12.0 (3-19)	12.3 (1-18)
Median no. doses (range)	4.0 (1-5)	12.0 (1-13)
Carboplatin		
Median weeks (range)	12.0 (3-19)	12.0 (3-17)
Median no. doses (range)	4.0 (1-5)	4.0 (1-5)
AC/EC		
N started AC/EC	737	739
Median weeks (range)	8.6 (2-18)	9.0 (2-17)
Median no. doses (range)	4.0 (1-4)	4.0 (1-4)
Atezolizumab/Placebo		
N started atezolizumab/placebo	768	762
Median weeks (range)	46.0 (3-58)	47.0 (3-57)
Median no. doses (range)	14.0 (1-18)	15.0 (1-18)
Neoadjuvant treatment		
Median weeks (range)	24 (3-35)	24 (3-36)
Median no. doses (range)	7 (1-11)	7 (1-10)
Adjuvant treatment		
N started adjuvant atezolizumab/placebo	572	619
Median weeks (range)	23.6 (3-35)	24 (3-36)
Median no. doses (range)	7 (1-11)	8 (1-12)

Supplementary Table 2: Summary of the EFS events

	**Chemo/Atezolizumab (n=773)**	**Chemo/Placebo (n=777)**
Patients with Event	110 (14.2%)	133 (17.1%)
Type of First Event:		
Neoadjuvant Progression	0 (0%)	1 (0.1%)
Locoregional Recurrence*	31 (4.0%)	37 (4.8%)
Distant Recurrence	58 (7.5%)	77 (9.9%)
Distant Recurrence Involving CNS	29 (3.8%)	24 (3.1%)
Distant Recurrence Not Involving CNS	29 (3.8%)	53 (6.8%)
Contralateral Invasive Breast Cancer	5 (0.6%)	3 (0.4%)
Second Non-breast Primary Cancer	10 (1.3%)	7 (0.9%)
Death at first EFS event	6 (0.8%)	8 (1.0%)

*Of them, 8 (1%) in each arm experienced distant recurrence within 8 weeks (none involved CNS).

Supplementary Table 3: Summary of the Immune-related AEs

	Chemo/Atezolizumab (n=768)	Chemo/Placebo (n=764)	Total (n=1532)
Overall	212 (27.6%)	87 (11.4%)	299 (19.5%)
Hypothyroidism	89 (11.6%)	17 (2.2%)	106 (6.9%)
Hyperthyroidism	52 (6.8%)	19 (2.5%)	71 (4.6%)
Infusion-related reaction	11 (1.4%)	5 (0.7%)	16 (1.0%)
Hyperglycemia	13 (1.7%)	9 (1.2%)	22 (1.4%)
Colitis	13 (1.7%)	4 (0.5%)	17 (1.1%)
Pneumonitis	10 (1.3%)	4 (0.5%)	14 (0.9%)
Adrenal Insufficiency	9 (1.2%)	4 (0.5%)	13 (0.8%)
Nephritis	11 (1.4%)	1 (0.1%)	12 (0.8%)
Hypophysitis	9 (1.2%)	1 (0.1%)	10 (0.7%)
Pancreatitis	5 (0.7%)	1 (0.1%)	6 (0.4%)
Rash maculo-papular	5 (0.7%)	0 (0.0%)	5 (0.3%)
Cytokine release syndrome	4 (0.5%)	0 (0.0%)	4 (0.3%)
Cardiac failure	3 (0.4%)	1 (0.1%)	4 (0.3%)
Left ventricular dysfunction	1 (0.1%)	0 (0.0%)	1 (0.1%)
Autoimmune hemolytic anemia	2 (0.3%)	0 (0.0%)	2 (0.1%)
Myocarditis	1 (0.1%)	0 (0.0%)	1 (0.1%)

Supplementary Table 4: PD-L1 vs stromal TILs

Parameter	*PD-L1 negative*	*PD-L1 positive*	*Overall*	*p-value*
TILs <10%	325 (38.4)	25 (3.6)	350 (22.7)	<.001
TILs 10-29%	361 (42.7)	249 (35.7)	610 (39.5)	
TILs ≥30%	160 (18.9)	423 (60.7)	583 (37.8)	
missing	7	0	7	

Supplementary Table 5: Baseline Characteristics: Demographic and Baseline Disease Characteristics in patients with and without HTG measurement

Parameter	Pts with HTG measurement (n=482)	Pts without HTG measurement (n=1068)
**Age (years)**		
Median	50.0	49.0
Range	23, 79	22, 79
**Nodal Status**		
Negative (cN0)	281 (58.3%)	620 (58.1%)
Positive (cN1, cN2, cN3)	201 (41.7%)	448 (41.9%)
**AC/EC Schedule**		
Every 2 weeks (q2w)	289 (60.0%)	707 (66.2%)
Every 3 Weeks (q3w)	193 (40.0%)	361 (33.8%)
**Clinical Size of the Primary Tumor**		
1.1-3.0 cm	313 (64.9%)	590 (55.2%)
> 3.0 cm	169 (35.1%)	478 (44.8%)
**PD-L1 Status**		
Negative/Indeterminate/Not available	300 (62.2%)	553 (51.8%)
Positive	182 (37.8%)	515 (48.2%)
**Histological Tumor Type**		
Invasive Ductal/Invasive Carcinoma of No Special Type (NST)	461 (95.6%)	1008 (94.4%)
Invasive Lobular Carcinoma or Mixed Lobular Carcinoma	8 (1.7%)	11 (1.0%)
Other	13 (2.7%)	49 (4.6%)
**Tumor Grade**		
1	3 (0.6%)	13 (1.2%)
2	85 (17.6%)	190 (17.8%)
3	394 (81.7%)	863 (80.8%)
Unknown/Missing	0 (0%)	2 (0.2%)
**Stromal TILs Category on Baseline Specimen** (%)		
<10%	119 (24.7%)	231 (21.6%)
10-29%	211 (43.8%)	399 (37.3%)
≥30%	152 (31.5%)	431 (40.4%)
Unknown/Missing	0 (0%)	7 (0.7%)
**Germline Pathogenic Variant Status**		
*BRCA*1 Pathogenic Variant	49 (10.2%)	80 (7.5%)
*BRCA*2 Pathogenic Variant	15 (3.1%)	23 (2.2%)
Wild Type for *BRCA*1 and *BRCA*2	273 (56.6%)	546 (51.1%)
Missing *BRCA* Germline Testing Status	149 (30.9%)	420 (39.3%)

Supplementary Figures

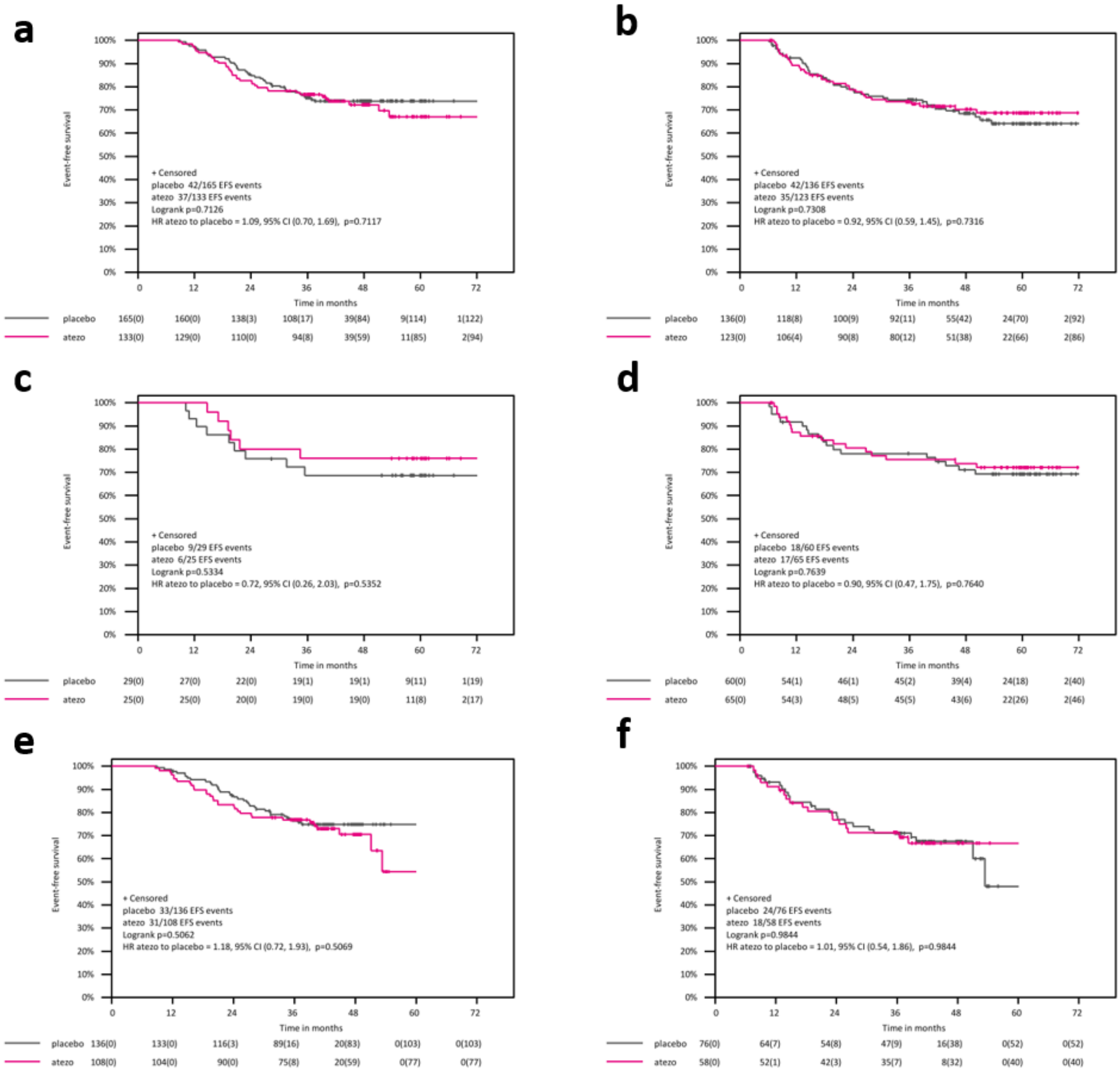

Supplementary Fig. 1: Kaplan-Meier plots of EFS with (a, c and e) and without (b, d and f) capecitabine treatment in non-pCR patients per treatment arm, overall (a and b) and separated for the patient who had surgery before amendment implementation (c and d) vs after amendment implementation (e and f). Data were analysed post hoc with landmark of 6 months to avoid guarantee-time bias, 557 non-pCR patients were included; p-values are from a two-sided log-rank test

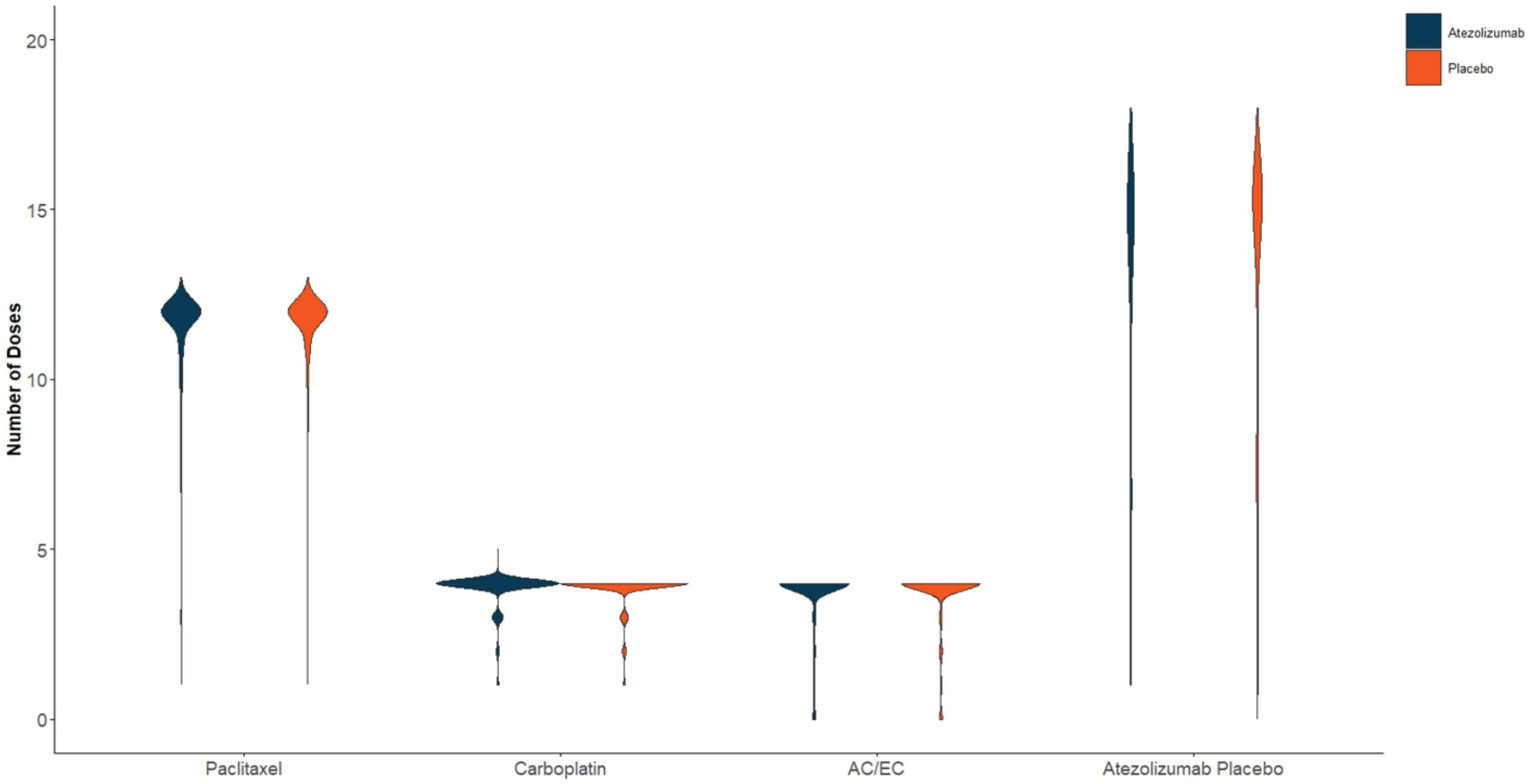

Supplementary Fig. 2: Drug delivery by treatment arm

Supplemental Information

Supplemental Information 1: List of ethic committees for NSABP B-59/GeparDOUZE

Supplemental Information 2: Study protocol, redacted

Supplemental Information 3: Summary of clinically important protocol amendments

Supplemental Information 4: Statistical plan, redacted

Supplemental Information 1: List of Ethic committees in NSABP B-59/GeparDouze

**Ethics committees Germany**
Ethikkommission der LÄK Baden-Württemberg, Liebknechtstr. 33, 70565 Stuttgart
Ethik-Kommission der Albert-Ludwigs-Universität Freiburg, EngelBerger Str. 21, 79106 Freiburg
Ethikkommission der Medizinischen Fakultät Heidelberg, Alte Glockengießerei 11/1, 69115 Heidelberg
Medizinische Ethik-Kommission II Medizinischen Fakultät Mannheim, Theodor-Kutzer-Ufer 1-3, 68167 Mannheim
Ethik-Kommission an der Medizinischen Fakultät der Eberhard-Karls-Universität und am Universitätsklinikum Tübingen, Gartenstr. 47, 72074 Tübingen
Ethik-Kommission der Universität Ulm, Oberberghof 7 Barbara Mez-Starck-Haus, 89081 Ulm
Bayerische Landesärztekammer, Ethik-Kommission, Mühlbaurstr. 16, 81677 München
Ethik-Kommission der Medizinischen Fakultät der Universität Erlangen-Nürnberg, Krankenhausstr. 12, 91054 Erlangen
Ethik-Kommission der Universität Würzburg, Josef-Schneider-Str. 4, 97080 Würzburg
Ethikkommission des Landes Berlin, Landesamt für Gesundheit und Soziales Berlin, Turmstr. 21, 10559 Berlin
Ethikkommission der Landesärztekammer Brandenburg, Dreifertstr. 12, 03044 Cottbus
Ethik-Kommission der Ärztekammer Hamburg, Weidestraße 122 b, 22083 Hamburg
**Ethikkommission der Landesärztekammer Hessen, Hanauer Landstraße 152, 60314 Frankfurt (federführend)**
Ethik-Kommission des Fachbereichs Medizin der Johann Wolfgang Goethe-Universität, Theodor-Stern-Kai 7, 60590 Frankfurt/ Main
Ernst-Moritz-Arndt-Universität Greifswald, Ethikkommission an der Medizinischen Fakultät Institut für Pharmakologie, Felix-Hausdorff-Str. 3, 17475 Greifswald
Ethikkommission an der Medizinischen Fakultät der Universität Rostock, St.-Georg-Str. 108, 18055 Rostock
Ethikkommission der Ärztekammer Niedersachsen, Karl-Wiechert-Allee 18-22, 30625 Hannover
Medizinischen Ethikkommission, Fakultät VI Medizin und Gesundheitswissenschaften, Carl von Ossietzky Universität Oldenburg, Carl von Ossietzky Str. 9-11, 26111 Oldenburg
Ethikkommission der Ärztekammer Nordrhein, Tersteegenstr. 9, 40474 Düsseldorf
Ethikkommission der Ärztekammer Westfalen-Lippe und der Westfälischen Wilhelms-Universität Münster, Gartenstr. 210 – 214, 48147 Münster
Ethik-Kommission an der Med. Fakultät der RWTH Aachen am Universitätsklinikum Aachen, Pauwelsstr. 30, 52074 Aachen
Ethikkommission der medizinischen Fakultät der HHU Düsseldorf, Moorenstraße 5, 40225 Düsseldorf
Ethik-Kommission der Medizinischen Fakultät der Universität Duisburg-Essen, Robert-Koch-Str. 9-11, 45147 Essen
Ethik-Kommission der Universität Witten/Herdecke, Alfred-Herrhausen-Str. 50, 58448 Witten
Ethik-Kommission bei der Landesärztekammer Rheinland-Pfalz, Deutschhausplatz 3, 55116 Mainz
Ärztekammer des Saarlandes Ethik-Kommission, Faktoreistr. 4, 66111 Saarbrücken
Sächsische Landesärztekammer Ethikkommission, Schützenhöhe 16, 1099 Dresden
Ethik-Kommission der Medizinischen Fakultät der TU Carl Gustav Carus, Fetscherstr. 74, 01307 Dresden
Ethik-Kommission des Landes Sachsen-Anhalt, Kühnauerstr. 70, 06846 Dessau-Roßlau
Ethik-Kommission der Medizinischen Fakultät der Christian-Albrechts-Universität zu Kiel, Schwanenweg 20, 24105 Kiel
Ethik-Kommission der Landesärztekammer Thüringen, Im Semmicht 33, 07751 Jena
**Ethics Committee for translational research**
Ethik-Kommission des Fachbereichs Medizin; Philipps-Universität Marburg, Baldingerstraße, 35043 Marburg, Germany
**Ethics committee Spain**

CEIM Parc Taulí<br>Parc Taulí 1, Edifici Santa Fe, Ala izquierda, 2a planta<br>08208 Sabadell (Barcelona) Spain
**Institutional Review Boards US**
Advarra IRB
Arrowhead Regional Medical Center IRB
Avera Health IRB
Decatur Memorial Hospital IRB
Greater Baltimore Medical Center IRB
Henry Ford Health System IRB
Holy Cross Hospital of Silver Spring IRB
Houston Methodist Hospital Research Institute IRB
Kaiser Foundation Southern California Region IRB
Kaiser Permanente Northwest IRB
Michigan State University Biomedical and Health IRB
Mount Sinai Medical Center IRB
Ochsner Clinic Foundation IRB
Ohio State University IRB
Orlando Regional Healthcare System, Inc. IRB
University of Louisville IRB
University of Missouri Health Sciences IRB
University of Pittsburgh IRB
US Oncology Research, Inc. IRB
Vassar Brothers Medical Center IRB
WCG IRB
Wellmont Health System IRB
Women & Infants Hospital of Rhode Island IRB
**Research Ethics Boards Canada**
CHU de Quebec-Universite Laval REB
CHUM: Centre Hospitalier de l'Universite de Montréal - Hotel-Dieu REB
CIUSSS West-Central Montreal REB - Jewish General
CIUSS de l'est-de-l'île-de-Montréal REB
McGill University Health Centre REB

NSABP PROTOCOL B-59/GBG 96-GeparDouze

(ClinicalTrials.gov NCT03281954)

A Randomized, Double-Blind, Phase III Clinical Trial of Neoadjuvant Chemotherapy with Atezolizumab or Placebo in Patients with Triple-Negative Breast Cancer Followed by Adjuvant Continuation of Atezolizumab or Placebo

NSABP Foundation, Inc.
[redacted]

TELEPHONE: 1-800-270-[redacted]
E-MAIL: [redacted]

FOR CLINICAL QUESTIONS: [redacted]

KEY STUDY PERSONNEL

NSABP Foundation Chair:	[redacted]
NSABP Foundation Breast Committee Chair:	[redacted]
German Breast Group Chair:	[redacted]
NSABP Protocol Chair:	[redacted]
German Breast Group Protocol Chair:	[redacted]
Protocol Officer:	[redacted]
Protocol Statisticians:	[redacted]
Protocol Pathologists:	[redacted]
Protocol Immuno-Oncologist	[redacted]
Co-ordinating Investigator Germany (according to the German drug law AMG)	[redacted]

Protocol NSABP B-59/GBG 96-GeparDouze IND #136073 (atezolizumab), sponsored by NSABP Foundation, Inc.

EudraCT No.: 2017-002771-25

This study will be conducted in compliance with the protocol, ICH-GCP, and any applicable regulatory requirements.

Version Date: December 20, 2022

PROTOCOL REVISION RECORD

Original Version: August 16, 2017

Amendment #1: October 2, 2017

NSABP B-59/GBG 96-GeparDouze Protocol was amended prior to Protocol Activation.

Amendment #2: **July 17, 2019**

Cover Page
Information Resources
Glossary of Abbreviations and Acronyms
Protocol Synopsis
Section 2.0: 2.0, 2.1, 2.2, 2.3, 2.4, 2.4.1, 2.4.2, 2.4.3, 2.5, 2.7, 2.8
Section:3.0: 3.4
Section 4.0: 4.0, 4.1.1, 4.1.5, 4.1.14, 4.1.15, 4.1.17, 4.1.19, 4.2.15
Section 5.0: Table 1, Table 2, Table 3
Section 6.0: 6.1 (Table 4), 6.2, 6.3, 6.3.1, 6.3.2, 6.3.4, 6.4, 6.5
Section 7.0: 7.0, 7.1 (Table 5), 7.2.1, 7.3.1, 7.5, 7.6, 7.8
Section 8.0: 8.1, 8.3, 8.6, 8.16 (Table 16), 8.18 (Table 18), 8.19 (Table 19), 8.20.2 (Table 20), 8.21 (Table 22), 8.22.2
Section 9.0: 9.1, 9.4, 9.7
Section 10.0: 10.0, 10.4.3, 10.5, 10.7.2
Section 11.0: 11.2
Section 13.0: 13.1, 13.2, 13.2.2, 13.3, 13.5
Section 14.0: 14.1
Section 15.0: 15.4, 15.6, 15.7.1, 15.7.4
Section 17.0: 17.1, 17.5
Section 18.0: 18.1, 18.7
Section 20.0
Appendices C and D

Amendment #3 **February 7, 2020**

Cover Page
Information Resources
Glossary of Abbreviations and Acronyms
Section 1.0
Section 2.0: 2.8
Section 4.0: 4.1.15, 4.1.16
Section 5.0: Table 2
Section 7.0: 7.1 (Table 5), 7.2.1, 7.2.2, 7.7

Section 8.0: 8.3, 8.7, 8.9 (Table 9), 8.11 (Table 11), 8.14 (Table 14), 8.18 (Table 18), 8.19 (Table 19)
Section 9.0: 9.4
Section 10.0: 10.5, 10.8
Section 15.0: 15.6
Section 17.0: 17.1, 17.2
Section 19.0
Appendices C and D

Amendment #4 **April 21, 2020**

Cover Page
Glossary of Abbreviations and Acronyms
Section 5.0: Table 1
Section 7.0: Table 5
Section 8.0: 8.3, 8.9, 8.10, 8.11, 8.12 (Table 11), 8.15 (Table 14), 8.17, 8.20, 8.22.3
Section 9.0: 9.1, 9.4

Amendment #5 **December 22, 2021**

Cover Page
Information Resources
Protocol Synopsis
Section 2.0: 2.2, 2.3, 2.4.1, 2.4.3, 2.8
Section 3.0: 3.1, 3.2, 3.3, 3.4
Section 5.0: Table 2
Section 7.0: 7.7
Section 8.0: 8.7, 8.9 (Table 8), 8.11 (Table 10), 8.12 (Table 11), 8.14 (Table 13), 8.15 (Table 14), 8.16 (Table 15), 8.17 (Table 16), 8.19 (Table 18), 8.20, 8.21 (Table 20), 8.22.3 (Table 21)
Section 9.0: 9.1, 9.9
Section 10.0: 10.0, 10.4.3, 10.5, 10.7.1, 10.7.2, 10.7.3
Section 11.0: 11.2
Section 12.0: 12.1
Section 13.0: 13.5
Section 15.0: 15.2, 15.3, 15.4, 15.7, 15.8.1, 15.8.2, 15.8.3, 15.8.4, 15.8.7
Section 18.0: 18.7
Section 20.0

Amendment #6 **March 3, 2022**

Cover Page
Section 3.0: 3.2.4
Section 8.0: 8.9 (Table 8), 8.12 (Table 11), 8.14 (Table 13), 8.16 (Table 15), 8.19 (Table 18), 8.20, 8.21 (Table 20), 8.22.3 (Table 21)
Section 10.0: 10.5
Section 20.0

Amendment #7 **December 20, 2022**

Cover Page

Information Resources

Section 1.0: Exploratory aims and endpoints, Statistical considerations

Figure 1

Section 3.0: 3.3.3, 3.3.5

Section 8.0: 8.7, 8.9 (Table 8), 8.11 (Table 10), 8.12 (Table 11), 8.17 (Table 16), 8.22.3, 8.22.4 (Table 21)

Section 15.0: 15.5, 15.8.1, 15.8.4

Section 20.0

TABLE OF CONTENTS

Information Resources 9
Glossary of Abbreviations and Acronyms 11

1.0 PROTOCOL SYNOPSIS 14

2.0 BACKGROUND 28
2.1 Atezolizumab as monotherapy in metastatic triple-negative breast cancer 29
2.2 Safety data for atezolizumab with taxanes (with or without carboplatin) (Atezolizumab IB 2018) 30
2.3 Efficacy of atezolizumab with nab-paclitaxel in metastatic triple-negative breast cancer (IMpassion130) 32
2.4 Carboplatin as component of neoadjuvant chemotherapy in triple-negative breast cancer 33
2.5 Rationale for the chemotherapy regimen 37
2.6 Rationale for atezolizumab dose and schedule 38
2.7 Overview of proposed trial 38
2.8 Allowance of capecitabine in patients with non-pCR or olaparib in patients with *gBRCA1* or *gBRCA2* mutations with non-pCR 39
2.9 Study delivery model 40

3.0 STUDY AIMS AND ENDPOINTS 41
3.1 Primary aim and endpoint 41
3.2 Secondary aims and endpoints 41
3.3 Exploratory aims and endpoints 42
3.4 Correlative science aims and endpoints 43

4.0 PATIENT ELIGIBILITY AND INELIGIBILITY 45
4.1 Conditions for patient eligibility 45
4.2 Conditions for patient ineligibility 47

5.0 REQUIREMENTS FOR STUDY ENTRY AND DURING TREATMENT AND FOLLOW-UP 51

6.0 PATHOLOGY AND CORRELATIVE SCIENCE STUDIES 57
6.1 Overview of requirements 57
6.2 Use of specimens 59
6.3 Tumor and plasma specimen submissions 59
6.4 Central testing for PD-L1 status 59
6.5 Rationale for correlative science studies 59

7.0 STUDY TREATMENT 61
7.1 Study regimen 61
7.2 Dose determinations 63
7.3 Supportive therapy 64
7.4 Concomitant therapy precautions 64
7.5 Surgery 65
7.6 Postoperative radiation therapy 65
7.7 Prohibited therapies 65
7.8 Participation in other clinical trials 65

8.0 TREATMENT MANAGEMENT 66
8.1 General instructions 66
8.2 Management of anemia 66

8.3 Management of protocol therapy with documented COVID-19 infection 66
8.4 Treatment management for carboplatin and paclitaxel 67
8.5 Dose modifications for carboplatin and paclitaxel 67
8.6 Dose modifications for AC/EC 70
8.7 Management of atezolizumab/placebo specific adverse events and potential unblinding 72
8.8 Treatment management for atezolizumab/placebo 74
8.9 Pulmonary events 74
8.10 Hepatic events 75
8.11 Gastrointestinal events 76
8.12 Endocrine events 77
8.13 Ocular events 80
8.14 Infusion-related reaction and cytokine-release syndrome 80
8.15 Management of pancreatic events 84
8.16 Dermatologic events 85
8.17 Neurological disorders 86
8.18 Immune-mediated meningoencephalitis 88
8.19 Management and Evaluation of Immune-Mediated Renal Events 88
8.20 Immune-mediated myositis 89
8.21 Hemophagocytic lymphohistiocytosis and macrophage activation syndrome 91
8.22 Immune-mediated cardiac toxicity 92
8.23 Management of atezolizumab/placebo during an asymptomatic decrease in LVEF 96
8.24 Liver dysfunction (Hy's Law) 97

9.0 DRUG INFORMATION 98
9.1 Chemotherapy 98
9.2 Atezolizumab/placebo description and supply 98
9.3 Atezolizumab 99
9.4 Preparation and administration 99
9.5 Procurement of atezolizumab/placebo 99
9.6 Transfer of atezolizumab/placebo 100
9.7 Destruction of atezolizumab/placebo 100
9.8 Drug accountability 100
9.9 Procedures for unblinding 100

10.0 ADVERSE EVENT REPORTING 102
10.1 Double-blind study drug 102
10.2 Definition of an AE 102
10.3 Definition of an SAE 103
10.4 Events requiring expedited reporting 103
10.5 Pregnancy 106
10.6 Grading the severity of the AE 106
10.7 Expedited reporting instructions 106
10.8 Time period and frequency for routine reporting of AEs 107

11.0 ASSESSMENT OF EFFECT 108
11.1 Evaluation of pCR 108
11.2 Disease progression on neoadjuvant therapy 108

12.0 DIAGNOSIS OF BREAST CANCER RECURRENCE 109
12.1 General instructions 109
12.2 Local recurrence 109
12.3 Regional recurrence 109
12.4 Distant recurrence 109

12.5 Contralateral breast cancer ... 110
12.6 Second primary cancer ... 110

13.0 PATIENT ENTRY PROCEDURES ... 111
13.1 Patient consent form ... 111
13.2 Study entry ... 111
13.3 Treatment assignment ... 111
13.4 Regulatory requirements for Canadian institutions ... 111
13.5 Requirements for discontinuation of study therapy ... 112
13.6 Patient-initiated discontinuation of study therapy ... 112
13.7 Patient-initiated withdrawal from the study ... 112

14.0 DATA HANDLING AND RECORDKEEPING ... 113
14.1 Instruction for completion of NSABP B-59/GBG 96-GeparDouze forms and materials ... 113

15.0 STATISTICAL CONSIDERATIONS ... 114
15.1 Study design ... 114
15.2 Primary aim and endpoint ... 114
15.3 Secondary endpoints ... 114
15.4 Exploratory endpoints ... 114
15.5 Stratification and randomization ... 115
15.6 Patient cohorts used for analysis ... 115
15.7 Sample size determination and protocol duration for the primary endpoint of EFS ... 115
15.8 Statistical analysis plans ... 116
15.9 Data Safety Monitoring Board (DSMB) ... 119

16.0 PREMATURE TERMINATION OF THE STUDY ... 120

17.0 ETHICAL AND LEGAL CONDUCT OF THE STUDY ... 121
17.1 Study conduct ... 121
17.2 Patient information and consent ... 121
17.3 Record retention ... 122
17.4 Confidential follow-up ... 122
17.5 Study agreements ... 122

18.0 STUDY MANAGEMENT ... 123
18.1 Pre-study activities ... 123
18.2 Training of study site personnel ... 123
18.3 Monitoring of the study ... 123
18.4 Auditing and inspection ... 123
18.5 Source data ... 123
18.6 Archiving of study documents ... 123
18.7 Data management ... 123

19.0 PUBLICATION INFORMATION ... 125

20.0 REFERENCES ... 126

Appendix A Assessment of Performance Status and Activities of Daily Living ... 131
Appendix B Comprehensive List of Autoimmune Diseases and Immune Deficiencies ... 132
Appendix C Country-Specific Surgery Guidelines ... 133
Appendix D Country-Specific Suggested Procedure For Evaluation of Surgical Specimens ... 138

Figure 1. NSABP B-59/GBG 96-GeparDouze Schema 27

Table 1. Tests, exams, and other requirements prior to randomization 51
Table 2. Tests, exams, and other requirements during therapy and through end of study for Arm 1 and Arm 2 53
Table 3. Additional studies required in cardiac safety lead-in study (first 60 patients to begin AC/EC) 56
Table 4. Summary of tumor and blood sample submission requirements 57
Table 5. Arm 1 and Arm 2 treatment regimen 61
Table 6. Dose levels for paclitaxel and carboplatin (Arm 1 and Arm 2) 67
Table 7. Dose levels for AC/EC (Arm 1 and Arm 2) 71
Table 8. Management guidelines for pulmonary events, including pneumonitis, related to atezolizumab/placebo 75
Table 9. Management guidelines for hepatic events related to atezolizumab/placebo 76
Table 10. Management guidelines for gastrointestinal events (diarrhea or colitis) related to atezolizumab/placebo 77
Table 11. Management guidelines for endocrine events related to atezolizumab/placebo 78
Table 12. Management guidelines for ocular events related to atezolizumab/placebo 80
Table 13. Management guidelines for infusion-related reactions and cytokine-release syndrome related to atezolizumab/placebo 82
Table 14. Management guidelines for pancreatic events, including pancreatitis, related to atezolizumab/placebo 84
Table 15. Management for dermatologic events related to atezolizumab/placebo 85
Table 16. Treatment management guidelines for neurological disorders related to atezolizumab/placebo 87
Table 17. Management guidelines for immune-mediated meningoencephalitis related to atezolizumab/placebo 88
Table 18. Management and evaluation of immune-mediated renal events (potential immune-mediated nephritis) 89
Table 19. Management and evaluation of immune-mediated myositis 90
Table 20. Management and evaluation of hemophagocytic lymphohistiocytosis and macrophage activation syndrome 92
Table 21. Treatment modifications and instructions for symptomatic cardiac toxicities including myocarditis related to atezolizumab/placebo 94
Table 22. CTCAE grade 2 other cardiac disorders 96
Table 23. Resumption of atezolizumab/placebo following surgery based on LVEF assessment after surgery 96

INFORMATION RESOURCES

NSABP Department of Site and Study Management	GBG
[redacted]<br>**Phone: 1-800-270-[redacted]**<br>**E-mail: [redacted]**	GBG Forschungs GmbH<br>[redacted]<br>**Phone: +49-6102-7480-[redacted]**<br>**E-mail: [redacted]**

	NSABP	GBG Sites
**For questions regarding:**<br>• IRB review & informed consent<br>• Submission of IRB approval<br>• SAEs	Department of Site and Study Management (DSSM)<br>Phone: 1-800-270-[redacted]<br>E-mail: [redacted]	Project Management<br>[redacted]<br>Phone: +49-6102-7480-[redacted]<br>E-mail: [redacted]
**For questions regarding:**<br>• Eligibility<br>• Treatment regimen<br>• Dose modifications/ delays<br>• Other clinical aspects of the trial	Clinical Coordinating Department (CCD)<br>Phone: 1-800-477-[redacted]<br>E-mail: [redacted]	Project Management<br>[redacted]<br>Phone: +49-6102-7480-[redacted]<br>E-mail: [redacted]
**For questions regarding data management**	Department of Site and Study Management (DSSM)<br>Phone: 1-800-270-[redacted]<br>E-mail: [redacted]	E-mail: [redacted]
**Questions regarding atezolizumab/placebo supply**	Department of Site and Study Management (DSSM)<br>Phone: 1-800-270-[redacted]<br>E-mail: [redacted]	E-mail: [redacted]
**For questions regarding SAE reporting**	[redacted]<br>Phone: 651-917-[redacted]<br>[redacted]	E-mail: [redacted]

Information resources continued on next page.

INFORMATION RESOURCES (continued)

	**NSABP**	**GBG Sites**
**Request for unblinding (including 24-hour emergency unblinding)**	IDDI Help Desk<br>Phone: 1-866-767-[redacted] (toll free in the US/Canada)	Project Management<br>[redacted]<br>Phone: +49-6102-7480-[redacted]<br>E-mail: [redacted]
**Questions about submission of tumor samples for central testing and correlative science studies**	NSABP Division of Pathology<br>[redacted]<br>Phone: 412-697-[redacted]<br>E-mail: [redacted]<br>*Refer to NSABP B-59/GBG 96-GeparDouze Pathology and Correlative Science Instructions*	Translational Research<br>[redacted]<br>Phone: +49-6102-7480-[redacted]
**Monitoring Coordination**		[redacted]<br>Phone: +49 (0) 6102 / 7480-[redacted]<br>E-mail: [redacted]

GLOSSARY OF ABBREVIATIONS AND ACRONYMS

AACR	American Association for Cancer Research
AC	doxorubicin (A) + cyclophosphamide (C)
AE	adverse event
AESI	adverse events of special interest
AJCC	American Joint Committee on Cancer
ALT	alanine aminotransferase
ANC	absolute neutrophil count
ASCO	American Society of Clinical Oncology
AST	aspartate aminotransferase
AUC	area under the curve
BP	blood pressure
BRCA	breast cancer susceptibility gene
°C	degree Celsius
CALGB	Cancer and Leukemia Group B
CBC	complete blood count
CCD	Clinical Coordinating Department
cCR	clinical complete response
CHF	chronic heart failure
CI	confidence interval
CRS	cytokine-release syndrome
CSSA	Clinical Site Study Agreement
CT	computed tomography
CTCAE v4.0	Common Terminology Criteria for Adverse Events Version 4.0
ctDNA	circulating tumor deoxyribonucleic acid
CTEP	Cancer Therapy Evaluation Program
DCIS	ductal carcinoma in situ
DD	dose dense
DDFS	distant disease-free survival
DFS	disease-free survival
DNA	deoxyribonucleic acid
DSMB	Data Safety Monitoring Board
DSSM	Department of Site and Study Management
EC	epirubicin (E) + cyclophosphamide (C)
ECG	electrocardiogram
ECOG	Eastern Cooperative Oncology Group
EDC	electronic data capture
eCRF	electronic case report form
EFS	event-free survival
ER	estrogen receptor
°F	degree Fahrenheit
FDA	Food and Drug Administration
FNA	fine needle aspiration

GLOSSARY OF ABBREVIATIONS AND ACRONYMS (continued)

GBG	German Breast Group
GCP	Good Clinical Practice
G-CSF	granulocyte colony stimulating factor
GGT	gamma-glutamyl transferase
GM-CSF	granulocyte macrophage-colony stimulating factor
GMP	Good Manufacturing Practice
H&P	history and physical
HER	human epidermal growth factor receptor
HIV	human immunodeficiency virus
HR	hazard ratio
IB	Investigator's Brochure
ICF	informed consent form
ICH	International Conference on Harmonization of Technical Requirements for Registration of Pharmaceuticals for Human Use
IDDI	International Drug Development Institute
iDMC	independent Data Safety Monitoring Committee
IEFS	invasive event-free survival
IHC	immunohistochemistry
IND	investigational new drug
INR	international normalized ratio
IRB	Institutional Review Board
IRR	infusion-related reaction
ITT	intent-to-treat
IV	intravenous
LFT	liver function test
LVEF	left ventricular ejection fraction
MDS	myelodysplastic syndrome
mg	milligram
MRI	magnetic resonance imaging
MUGA	MUltiGAted Aquisition
NaCl	sodium chloride
NCI	National Cancer Institute
NSABP	NSABP Foundation, Inc.
ORR	objective response rate
OS	overall survival
PD-1	programmed death 1
PD-L1	programmed death-ligand 1
pCR	pathologic complete response
PET	positron emission tomography
PFS	progression-free survival
PR	progesterone receptor
REB	Research Ethics Board

GLOSSARY OF ABBREVIATIONS AND ACRONYMS (continued)

RFI	recurrence-free interval
RNA	ribonucleic acid
RT	radiation therapy
SABCS	San Antonio Breast Cancer Symposium
SAE	serious adverse event
TIA	transient ischemic attacks
TILs	tumor-infiltrating lymphocytes
TNBC	triple-negative breast cancer
TSH	thyroid-stimulating hormone
ULN	upper limit of normal
WBC	white blood cell
WHO	World Health Organization
WP	weekly paclitaxel

1.0 PROTOCOL SYNOPSIS

**Study Title**	**A Randomized, Double-Blind, Phase III Clinical Trial of Neoadjuvant Chemotherapy with Atezolizumab or Placebo in Patients with Triple-Negative Breast Cancer Followed by Adjuvant Continuation of Atezolizumab or Placebo**
**Study Code**	**NSABP B-59/GBG 96**
**EudraCT Number**<br>**FDA IND Number**	**2017-002771-25**<br>**136073**
**Study delivery model and sponsor**	This is a collaborative study being conducted by NSABP Foundation, Inc. in partnership with the German Breast Group (GBG), and supported by funding [redacted]<br>Two versions of a single protocol have been written to cover this collaborative neoadjuvant breast cancer study. One protocol version covers all patients recruited by GBG and the other protocol version covers patients recruited by NSABP Foundation, Inc. The two versions of the protocol are identical in terms of study objectives and scientific content and only differ in logistical content appropriate for the country(ies) they cover and exploratory studies of the microbiome and ovarian function.
**Development phase**	NSABP B-59/GBG 96-GeparDouze is a prospective, randomized, double-blind, Phase III clinical trial.
**Study overview**	In this clinical trial of neoadjuvant and adjuvant administration of atezolizumab/placebo in patients with high risk triple-negative breast cancer, the potential incremental efficacy and safety of neoadjuvant administration of atezolizumab/placebo with a sequential regimen of weekly paclitaxel with every-3-week carboplatin followed immediately by neoadjuvant administration of atezolizumab/placebo with AC/EC will be evaluated. Patients will then undergo surgery (should be performed within 6 weeks after last chemotherapy application). Following recovery from surgery, patients will initiate approximately 6 months of adjuvant therapy with atezolizumab/placebo and receive the same investigational agent they received pre-operatively. Administration of radiation therapy will be based on local standards at the discretion of patients and investigators, but if administered, atezolizumab/placebo will be administered concurrently. Adjuvant atezolizumab/placebo may be delayed until after completion of radiation therapy per investigator discretion. Patients with residual invasive cancer at the time of surgery may receive capecitabine concurrently with atezolizumab/placebo in the adjuvant setting per investigator discretion and local guidelines. Patients with germline *BRCA1* or *BRCA2* mutations with residual invasive cancer at the time of surgery may receive olaparib in the adjuvant setting per investigator discretion and local guidelines. Patients receiving olaparib must discontinue atezolizumab/placebo.<br>In order to proactively identify and further assess any cardiac toxicity that may occur with the combination of anthracyclines and atezolizumab, this study includes a cardiac safety lead-in for the first 60 patients who initiate AC/EC.<br>Research core biopsy of breast primary at baseline is a study requirement for all patients. A research biopsy at 1-4 days prior to the second dose of

	atezolizumab/placebo is a study requirement for the first 500 patients who agree to an optional research biopsy. One to three representative blocks of residual primary tumor containing the maximum amount of tumor and node with the largest focus of metastasis is required from the definitive breast surgery if gross residual disease is ≥ 1.0 cm. If gross residual disease is < 1.0 cm, tissue should be submitted, if possible. Plasma samples for ctDNA analysis will be collected at multiple times during the study. Accrual for this study will be 1,520 randomized patients. A total of 1550 patients were accrued in 42 months. Follow-up of an additional 22 months after completion of accrual (total time from first patient in to EFS analysis of 64 months) would be sufficient to obtain 252 EFS events.
**Study regimen**	• 12 weeks Paclitaxel 80 mg/m2 IV weekly + Carboplatin (AUC 5) IV every 3 weeks + Atezolizumab 1200 mg or placebo IV Day 1 every 3 weeks for 4 doses followed by<br>• 4 x Doxorubicin 60 mg/m2 IV + Cyclophosphamide 600 mg/m2 IV OR 4 x Epirubicin 90 mg/m2 IV + Cyclophosphamide 600 mg/m2 IV Day 1 every 2 or 3 weeks + Atezolizumab 1200 mg /placebo IV every 3 weeks<br>• After Surgery: Atezolizumab 1200 mg or placebo IV Day 1 every 3 weeks until 52 weeks after the first dose
**Primary aim and endpoint**	Event-free survival (EFS)<br>*Aim:* To determine whether the addition of atezolizumab to chemotherapy (weekly paclitaxel plus carboplatin followed by AC or EC) followed by adjuvant atezolizumab improves event-free survival (EFS) in patients with triple-negative breast cancer.<br>*Endpoint:* EFS is defined as time from randomization until event: EFS events are progression on protocol therapy resulting in administration of non-protocol cancer therapy or inoperability, local invasive recurrence following mastectomy, local invasive recurrence in the ipsilateral breast following lumpectomy, regional recurrence, distant recurrence, contralateral invasive breast cancer, second non-breast primary cancer (excluding squamous or basal cell carcinoma of the skin), or death from any cause prior to recurrence or second primary cancer.
**Secondary aims and endpoints**	• Overall survival (OS)<br>*Aim:* To determine whether the addition of atezolizumab to chemotherapy (weekly paclitaxel plus carboplatin followed by AC or EC) followed by adjuvant atezolizumab improves overall survival (OS) in patients with triple-negative breast cancer.<br>*Endpoint:* Defined as time from randomization until death from any cause.<br>• Pathologic complete response in the breast and lymph nodes (ypT0/Tis ypN0)<br>*Aim:* To determine whether the addition of atezolizumab to chemotherapy (weekly paclitaxel plus carboplatin followed by AC or EC) improves pathologic complete response in the breast and post-therapy lymph nodes evaluated histologically (pCR breast and nodes) in patients with triple-negative breast cancer.

	Endpoint: Defined as the absence of any invasive component in the resected breast specimen and all resected lymph nodes following completion of neoadjuvant therapy (ypT0/Tis ypN0).<br>• Distant disease-free survival (DDFS)<br>*Aim:* To determine whether the addition of atezolizumab to chemotherapy (weekly paclitaxel plus carboplatin followed by AC or EC) followed by adjuvant atezolizumab improves distant disease-free survival (DDFS) in patients with triple-negative breast cancer.<br>*Endpoint:* Defined as time from randomization until distant recurrence, death from breast cancer, death from other causes, and second primary invasive cancer (non-breast).<br>• Disease-free survival (DFS)<br>*Aim:* To determine whether the addition of atezolizumab to chemotherapy (weekly paclitaxel plus carboplatin followed by AC or EC) followed by adjuvant atezolizumab improves disease-free survival (DFS) in patients with triple-negative breast cancer.<br>*Endpoint:* Defined as time from the first breast surgical procedure (operation) to the first occurrence of disease recurrence or death from any cause. Events defining DFS are ipsilateral invasive breast tumor recurrence, ipsilateral local-regional invasive breast cancer recurrence, distant recurrence, contralateral invasive breast cancer, ipsilateral or contralateral DCIS, second primary non-breast invasive cancer (with the exception of non-melanoma skin cancers and non-breast in situ carcinoma of any site) and death attributable to any cause including breast cancer, non-breast cancer, or unknown cause.<br>• Toxicity<br>*Aim:* To evaluate toxicity associated with study therapy added to chemotherapy and radiation therapy.<br>*Aim:* To evaluate immune-adverse events of special interest<br>*Endpoint:* Frequency and severity of adverse events graded according to the NCI Common Terminology Criteria for Adverse Events version 4.0 (CTCAE v4.0).<br>• Cardiac safety lead-in study<br>*Aim:* To assess for potential augmentation of anthracycline-related cardiac toxicity with co-administration of study therapy.<br>*Endpoints:* Troponin-T levels 1) prior to initial dose of AC/EC following completion of carboplatin/paclitaxel co-administered with atezolizumab/placebo and after administration of the 1st and 3rd cycles of AC/EC prior to administration of atezolizumab/placebo.<br>LVEF levels at 1) baseline prior to initiation of carboplatin/paclitaxel and atezolizumab/placebo, 2) before 3rd cycle of AC/EC, and 3) after surgery.
**Exploratory aims and endpoints**	• Pathologic complete response in the breast (ypT0/Tis)<br>*Aim:* To determine whether the addition of atezolizumab to chemotherapy (weekly paclitaxel plus carboplatin followed by AC or EC) improves

	pathologic complete response in the breast (pCR breast) in patients with triple-negative breast cancer.<br>*Endpoint:* Defined as the absence of any invasive component in the resected breast specimen (nodal material not considered).<br>• Pathologic complete response in the breast and lymph nodes (ypT0 ypN0)<br>*Aim:* To determine whether the addition of atezolizumab to chemotherapy (weekly paclitaxel plus carboplatin followed by AC or EC) improves pathologic complete response in the breast and post-therapy lymph nodes evaluated histologically (pCR breast and nodes) in patients with triple-negative breast cancer.<br>*Endpoint:* Defined as the absence of any invasive component or DCIS in the resected breast specimen and all resected lymph nodes following completion of neoadjuvant therapy.<br>• Positive nodal status conversion rate<br>*Aim:* To determine whether the addition of atezolizumab to chemotherapy (weekly paclitaxel plus carboplatin followed by AC or EC) improves conversion rate from clinically node-positive to pathologically node-negative.<br>*Endpoint:* Percentage of patients clinically node-positive that convert to pathologically node-negative following completion of neoadjuvant chemotherapy.<br>• Recurrence-free interval (RFI)<br>*Aim:* To determine whether the addition of atezolizumab to chemotherapy (weekly paclitaxel plus carboplatin followed by AC or EC) followed by adjuvant atezolizumab improves recurrence-free interval (RFI) in patients with triple-negative breast cancer.<br>*Endpoint:* Defined as time from randomization until invasive local, regional, or distant recurrence, or death from breast cancer (censored for death from other causes).<br>• Brain metastases free survival<br>*Aim:* To determine whether the addition of atezolizumab to chemotherapy (weekly paclitaxel plus carboplatin followed by AC or EC) followed by adjuvant atezolizumab improves brain metastases free survival in patients with triple-negative breast cancer.<br>*Endpoint:* Defined as time from randomization until documentation of first event brain metastasis or death from any cause. Patients who develop locoregional or distant recurrence outside of the central nervous system as first event will continue to be followed for subsequent development of brain metastases.<br>• Pathologic complete response in the breast and lymph nodes (ypT0/Tis ypN0) in patients with deleterious germline *BRCA* mutation status<br>*Aim:* To determine whether the addition of atezolizumab to chemotherapy (weekly paclitaxel plus carboplatin followed by AC or EC) improves pathologic complete response in the breast and post-therapy lymph nodes evaluated histologically (pCR breast and nodes [ypT0/Tis ypN0]) in patients with deleterious germline *BRCA* mutation status.

	• Event-free survival (EFS) in patients with deleterious germline *BRCA* mutation status<br>*Aim:* To determine whether the addition of atezolizumab to chemotherapy (weekly paclitaxel plus carboplatin followed by AC or EC) followed by adjuvant atezolizumab improves event-free survival (EFS) in patients with deleterious germline *BRCA* mutation status.
**Correlative science aims and endpoints**	• *Aim:* To evaluate baseline expression of PD-L1 and expression of PD-L1 following initial dose of atezolizumab with paclitaxel plus carboplatin as predictor for pCR of breast and nodes (ypT0/Tis ypN0) following neoadjuvant administration of atezolizumab with chemotherapy.<br>*Endpoint:* pCR of breast and nodes (ypT0/Tis ypN0)<br>• *Aim:* To evaluate baseline expression of PD-L1 and expression of PD-L1 following initial dose of atezolizumab with paclitaxel plus carboplatin as predictor for EFS following neoadjuvant administration of atezolizumab with chemotherapy followed by adjuvant administration of atezolizumab<br>*Endpoint:* EFS<br>• *Aim:* To evaluate baseline percentages of TILs and percentage of TILs following initial dose of atezolizumab with paclitaxel plus carboplatin as predictor for pCR of breast and nodes following neoadjuvant administration of atezolizumab with chemotherapy<br>*Endpoint:* pCR of breast and nodes (ypT0/Tis ypN0)<br>• *Aim:* To evaluate baseline percentages of TILs and percentage of TILs following initial dose of atezolizumab with paclitaxel plus carboplatin as predictor for EFS following neoadjuvant administration of atezolizumab with chemotherapy followed by adjuvant administration of atezolizumab<br>*Endpoint:* EFS<br>• *Aim:* To evaluate percentages of TILs in patients with residual breast cancer at time of surgery following neoadjuvant administration of atezolizumab with chemotherapy followed by adjuvant administration of atezolizumab as predictor for EFS.<br>*Endpoint:* EFS<br>• *Aim:* To use baseline and on therapy specimens, such as plasma ctDNA, to explore potential new biomarkers of responses and resistance to neoadjuvant administration of atezolizumab with chemotherapy followed by adjuvant administration of atezolizumab.
**Patient eligibility**	Investigators should consider each of these factors when selecting patients for this trial. Investigators should also consider all other relevant factors (medical and non-medical), as well as the risks and benefits of the study therapy, when deciding if a patient is an appropriate candidate for this trial.<br>• Study therapy should begin within 2 weeks after randomization and will continue for 1 year total of study therapy (inclusive of neoadjuvant and adjuvant therapy). Discussion should include patient availability and the timing of study therapy.<br>• ***Submission of samples from the breast surgery if there is residual tumor measuring ≥ 1 cm is required for all patients*** (see Section 6.1). The local

	country and pathology department policies regarding release of blocks must be considered when screening patients.<br>• *BRCA* mutation testing ***is not required*** for entry in the NSABP B-59/GBG 96-GeparDouze study; however, *BRCA* mutation status (positive; negative, unknown) will be collected at entry. If *BRCA* mutation testing has not been performed, record the *BRCA* mutation status as "Unknown." If *BRCA* mutation status becomes available subsequent to entry, the *BRCA* mutation status should be updated.<br>**Conditions for patient eligibility**<br>1. The patient must have consented to participate and, prior to beginning specific study procedures, must have signed and dated an appropriate IRB-approved consent form that conforms to federal and institutional guidelines for study treatment and for submission of tumor samples from a research biopsy as required by NSABP B-59/GBG 96-GeparDouze for baseline correlative science studies (see Section 6.0).<br>2. The diagnosis of invasive adenocarcinoma of the breast must have been made by core needle biopsy.<br>3. Local testing on the diagnostic core must have determined the tumor to be ER-negative, PgR-negative, and HER2-negative by current ASCO/CAP guidelines. (If local testing has determined a tumor to be HER2 equivocal or to have a borderline ER/PgR status (% IHC staining < 10% for both) and other eligibility criteria are met, material may be submitted for central testing to determine eligibility.)<br>4. Central testing for ER, PgR, and HER2 will be performed, and the tumor must be determined to be ER-negative, PgR-negative, and HER2-negative by current ASCO/CAP Guidelines Recommendations.<br>5. The tumor specimen used for central ER, PgR, and HER2 testing must also be used for central testing of PD-L1 status using the Ventana PD-L1 (SP142) assay kit. Patients will be eligible irrespective of PD-L1 testing result including PD-L1 indeterminate. Patients will be classified as positive, negative, or indeterminate for stratification purposes.<br>6. Patients must be ≥ 18 years old.<br>7. Patient may be female or male.<br>8. The ECOG performance status must be 0-1 (see Appendix A).<br>9. The primary tumor can be clinical stage T2 or T3, if clinically node negative according to AJCC 7th Edition. If the regional lymph nodes are cN1 and cytologically or histologically positive or cN2–N3 with or without a biopsy, the primary breast tumor can be clinically T1c, T2, or T3.<br>10. Ipsilateral axillary lymph nodes must be evaluated by imaging (ultrasound, and/or MRI) within 42 days prior to study entry. If suspicious or abnormal, FNA or core biopsy is recommended. Findings of these evaluations will be used to define the nodal status prior to study entry according to the following criteria:<br>*Nodal status – negative*<br>- Imaging of the axilla is negative;

- Imaging is suspicious or abnormal but the FNA or core biopsy of the questionable node(s) on imaging is negative;

Nodal status – positive

- FNA or core biopsy of the node(s) is cytologically or histologically suspicious or positive.
- Imaging is suspicious or abnormal but FNA or core biopsy was not performed.

11. Patients with synchronous bilateral or multicentric HER2-negative breast cancer are eligible as long as the highest risk tumor is ER-negative and PgR-negative and meets stage eligibility criteria. All of the other invasive tumors must also be HER2-negative by ASCO/CAP Guidelines based on local testing. Central testing to confirm TNBC status is only required for the highest risk tumor.
12. Blood counts performed within 28 days prior to randomization must meet the following criteria:
    - ANC must be ≥ 1500/mm^3;
    - platelet count must be ≥ 100,000/mm^3; and
    - hemoglobin must be ≥ 10 g/dL.
13. The following criteria for evidence of adequate hepatic function performed within 28 days prior to randomization must be met:
    - total bilirubin must be ≤ ULN for the lab unless the patient has a bilirubin elevation > ULN to 1.5 x ULN due to Gilbert's disease or similar syndrome involving slow conjugation of bilirubin; *and*
    - alkaline phosphatase must be ≤ 2.5 x ULN for the lab; *and*
    - AST and ALT must be ≤ 1.5 x ULN for the lab.
14. Patients with AST or ALT or alkaline phosphatase > ULN are eligible for inclusion in the study if liver imaging (CT, MRI, abdominal ultrasound, PET-CT, or PET scan) performed within 28 days prior to randomization does not demonstrate metastatic disease and the requirements in criterion 13 are met.
15. Patients with alkaline phosphatase that is > ULN but ≤ 2.5 x ULN or with unexplained bone pain are eligible for inclusion in the study if bone imaging (bone scan, PET-CT scan, or PET scan) supported by additional studies when indicated (CT, x-ray, MRI) performed within 42 days prior to randomization does not demonstrate metastatic disease.
16. Patients with N2 or N3 nodal disease or T3 primary disease must undergo liver imaging within 28 days prior to randomization and bone imaging (as described in criteria 14 and 15) within 42 days prior to randomization, irrespective of baseline lab results, and studies must not demonstrate metastatic disease. Chest imaging with chest x-ray PA and Lateral, CT of the chest, or PET-CT must also be performed.
17. Creatinine clearance ≥ 50 mL/min (see Section 7.2.1 for instructions regarding calculation of creatinine clearance) performed within 28 days prior to randomization.

	18. PT/INR ≤ ULN within 28 days prior to randomization. For laboratories that do not report an ULN for the INR assay, use ≤ 1.2 as the value for the ULN. Patients receiving therapeutic anti-coagulants are not eligible.<br>19. A serum TSH and AM (morning) cortisol performed within 28 days prior to randomization to obtain a baseline value. Patients with abnormal TSH or AM cortisol baseline levels should be further evaluated and managed per institutional standards. Asymptomatic patients who require initiation or adjustment of medication or are followed without initiating treatment based on endocrinologist's recommendations are eligible.<br>20. LVEF assessment must be performed within 42 days prior to randomization. (LVEF assessment performed by echocardiogram is preferred; however, MUGA scan may be substituted based on institutional preferences.) ***The LVEF must be ≥ 55% regardless of the cardiac imaging facility's lower limit of normal.***<br>21. For women of childbearing potential: agreement to remain abstinent (refrain from heterosexual intercourse) or use contraceptive methods that result in a failure rate of < 1% per year during the treatment period and for at least 5 months after the last dose of atezolizumab/placebo or 12 months after the last dose of chemotherapy.<br>A woman is considered to be of childbearing potential if she is not postmenopausal, has not reached a postmenopausal state (≥ 12 continuous months of amenorrhea with no identified cause other than menopause), and has not undergone surgical sterilization (removal of ovaries and/or uterus).<br>Examples of contraceptive methods with a failure rate of < 1% per year include: bilateral tubal ligation; male partner sterilization; intrauterine devices.<br>The reliability of sexual abstinence should be evaluated in relation to the duration of the clinical study and the preferred and usual lifestyle of the patient. Periodic abstinence (e.g., calendar, ovulation, symptothermal, or postovulation methods) and withdrawal are not acceptable methods of contraception.<br>22. Patient must be willing and able to comply with scheduled visits, treatment plans, laboratory tests, and other study procedures.
**Patient ineligibility**	1. Excisional biopsy or lumpectomy performed prior to study entry.<br>2. FNA alone to diagnose the breast cancer.<br>3. Surgical axillary staging procedure prior to randomization. Exception: FNA or core biopsy of an axillary node is permitted for any patient. A pre-neoadjuvant therapy sentinel lymph node biopsy for patients with clinically negative axillary nodes is prohibited.<br>4. Definitive clinical or radiologic evidence of metastatic disease.<br>5. Previous history of contralateral invasive breast cancer. (Patients with synchronous and/or previous contralateral DCIS or LCIS are eligible.)<br>6. Previous history of ipsilateral invasive breast cancer or ipsilateral DCIS. (Patients with synchronous or previous ipsilateral LCIS are eligible.)

	7. History of non-breast malignancies (except for in situ cancers treated only by local excision and basal cell and squamous cell carcinomas of the skin) within 5 years prior to study entry.<br>8. Treatment including radiation therapy, chemotherapy, or targeted therapy, for the currently diagnosed breast cancer prior to randomization.<br>9. Previous therapy with anthracyclines or taxanes for any malignancy.<br>10. Cardiac disease (history of and/or active disease) that would preclude the use of the drugs included in the treatment regimens. This includes but is not confined to:<br>*Active cardiac disease:*<br>- angina pectoris that requires the use of anti-anginal medication;<br>- ventricular arrhythmias except for benign premature ventricular contractions;<br>- supraventricular and nodal arrhythmias requiring a pacemaker or not controlled with medication;<br>- conduction abnormality requiring a pacemaker;<br>- valvular disease with documented compromise in cardiac function; or<br>- symptomatic pericarditis.<br>*History of cardiac disease:*<br>- myocardial infarction documented by elevated cardiac enzymes or persistent regional wall abnormalities on assessment of left ventricular function within 6 months prior to randomization;<br>- history of documented CHF; or<br>- documented cardiomyopathy.<br>11. Uncontrolled hypertension defined as sustained systolic BP > 150 mmHg or diastolic BP > 90 mmHg. (Patients with initial BP elevations are eligible if initiation or adjustment of BP medication lowers pressure to meet entry criteria.) Patients requiring ≥ 3 BP medications are not eligible.<br>12. History of severe allergic, anaphylactic, or other hypersensitivity reactions to chimeric or humanized antibodies or fusion proteins.<br>13. Known hypersensitivity to biopharmaceuticals produced in Chinese hamster ovary cells.<br>14. Known allergy or hypersensitivity to the components of the atezolizumab formulation.<br>15. Known allergy or hypersensitivity to the components of the doxorubicin, epirubicin, cyclophosphamide, carboplatin, or paclitaxel formulations.<br>16. Known allergy or hypersensitivity to liposomal or pegylated G-CSF formulations.<br>17. Active or history of autoimmune disease or immune deficiency, including but not limited to myasthenia gravis, myositis, autoimmune hepatitis, systemic lupus erythematosus, rheumatoid arthritis, inflammatory bowel disease, antiphospholipid syndrome, Wegener granulomatosis, Sjögren syndrome, Guillain-Barré syndrome, or multiple sclerosis (see Appendix B) with the following exceptions:

	- Patients with a history of autoimmune-related hypothyroidism on a stable dose of thyroid replacement hormone may be eligible for this study.<br>- Patients with controlled Type 1 diabetes mellitus on a stable dose of insulin regimen may be eligible for this study.<br>- Patients with eczema, psoriasis, lichen simplex chronicus, or vitiligo with dermatologic manifestations only (e.g., patients with psoriatic arthritis are excluded) are permitted provided all of following conditions are met:<br>• Rash must cover < 10% of body surface area.<br>• Disease is well controlled at baseline and requires only low-potency topical corticosteroids.<br>• No occurrence of acute exacerbations of the underlying condition requiring psoralen plus ultraviolet A radiation, methotrexate, retinoids, biologic agents, oral calcineurin inhibitors, or high-potency or oral corticosteroids within the previous 12 months.<br>18. History of idiopathic pulmonary fibrosis, organizing pneumonia (e.g., bronchiolitis obliterans), drug-induced pneumonitis, idiopathic pneumonitis, or evidence of active pneumonitis on screening chest CT scan.<br>19. Patients known to be HIV positive.<br>20. Active hepatitis B virus (HBV) infection, defined as having a positive hepatitis B surface antigen (HBsAg) test at screening. Patients with a past or resolved HBV infection, defined as having a negative HBsAg test and a positive total hepatitis B core antibody (HBcAb) test at screening, are eligible for the study if active HBV infection is ruled out on the basis of HBV DNA viral load per local guidelines.<br>21. Active hepatitis C virus (HCV) infection, defined as having a positive HCV antibody test at screening confirmed by a polymerase chain reaction (PCR) positive for HCV RNA.<br>22. Patients with clinically active tuberculosis.<br>23. Severe infection within 28 days prior to randomization, including but not limited to hospitalization for complications of infection, bacteremia, or severe pneumonia.<br>24. Prior allogeneic stem cell or solid organ transplantation.<br>25. Administration of a live, attenuated vaccine within 28 days prior to randomization or anticipation that such vaccine will be required during the study. Patients must agree not to receive live, attenuated influenza vaccine (e.g., FluMist®) within 28 days prior to randomization, during treatment or within 5 months following the last dose of atezolizumab/placebo.<br>26. Any other disease, metabolic dysfunction, physical examination finding, or clinical laboratory finding giving reasonable suspicion of a disease or condition that contraindicates the use of an investigational drug or that may affect the interpretation of the results or render the patient at high risk from treatment complications.

	27. Prior treatment with CD137 agonists or immune checkpoint-blockade therapies, including anti-CD40, anti-CTLA-4, anti-PD-1, and anti-PD-L1 therapeutic antibodies.<br>28. Treatment with systemic immunosuppressive medications (including but not limited to interferons, IL-2) within 28 days or 5 half-lives of the drug, whichever is longer, prior to randomization.<br>29. Treatment with systemic immunosuppressive medications (including but not limited to prednisone, cyclophosphamide, azathioprine, methotrexate, thalidomide, and anti−tumor necrosis [anti-TNF] factor agents) within 14 days prior to randomization or anticipation of need for systemic immunosuppressive medications during the study.<br>30. Nervous system disorder (paresthesias, peripheral motor neuropathy, or peripheral sensory neuropathy) ≥ Grade 2, per the CTCAE v4.0.<br>31. Symptomatic peripheral ischemia.<br>32. Pregnancy or lactation at the time of randomization or intention to become pregnant during the study. ***(Note: Negative serum pregnancy test must be obtained within 14 days prior to randomization)***.<br>33. Use of any investigational agent within 28 days prior to randomization.
**Statistical considerations**	**Stratification and randomization**<br>Randomization will be accomplished using stratified permuted blocks. Eligible triple-negative breast cancer patients will be randomized to receive either<br>paclitaxel/carboplatin/placebo followed by doxorubicin/cyclophosphamide/placebo or epirubicin/cyclophosphamide/placebo<br>or<br>paclitaxel/carboplatin/atezolizumab followed by doxorubicin/cyclophosphamide/atezolizumab or epirubicin/cyclophosphamide/atezolizumab.<br>The stratification factors for the study are:<br>1) Group (NSABP Foundation, Inc.; GBG)<br>2) Nodal status as determined by protocol-specified criteria (negative, positive)<br>3) AC/EC (every 2 weeks; every 3 weeks)<br>4) Clinical size of the primary tumor (1.1-3.0 cm; > 3.0 cm)<br>5) PD-L1 status (positive; negative, indeterminate, or not available*)<br>* PD-L1 status of patients were not available at randomization for patients enrolled prior to amendments in July 2019.<br>**Patient cohorts used for analysis**<br>• The intent-to-treat (ITT) population (defined as all randomized patients) will be used for analysis of the primary and secondary endpoints except toxicity. Patients will be analyzed according to their randomly assigned treatment regardless of treatment actually received.<br>• The safety population includes all patients who receive at least one dose of study therapy and will be used for the toxicity endpoint. Patients will be

	analyzed according to treatment actually received, thus any patient receiving at least one dose of atezolizumab/placebo will comprise the experimental group.<br><br>**Sample size determination and protocol duration for the primary endpoint of EFS**<br><br>The sample size for this trial is based on the requirements for testing of the EFS endpoint. The hazard rates used for determination were taken from our experience in NSABP B-40 trial. In NSABP B-40, the annual hazard rates were not constant and varied substantially over the first 4 to 5 years. Thus, instead of using a constant hazard rate across time, yearly-specific hazards rates were used. These rates were then reduced by 20% to account for an anticipated effect of adding carboplatin to the treatment regimen based on CALGB 40603 (Sikov SABCS 2015). To achieve monthly precision in estimates of accrual and study duration, the calculation of sample size was based on monthly hazard rates converted from each of the yearly hazard rates described above. The resulting monthly hazard rates are 0.00460 during year 1, 0.01020 during year 2, 0.00440 during year 3, 0.00186 during year 4 and 0.00200 for year 5 and all subsequent years.<br><br>The original sample size was based on having sufficient statistical power to detect a hazard ratio of 0.70 at α=0.045 assuming a lost-to-follow-up rate of 0.00083 per month. A sample size of 760 patients per arm for a total sample size of 1,520 patients followed to obtain 269 EFS events would provide a test of hypothesis with 80% statistical power at an overall alpha level of 0.045. After allowing for a ramp-up of accrual in the first eighteen months to 40 patients per month and a further ramp-up to 90 per month by accrual month 28, it is anticipated that the accrual rate would peak at 105 patients per month by accrual month 32. With this accrual pattern, it would take about 34 months to accrue 1,520 patients. Follow-up of an additional 30 months after completion of accrual (total study time of 64 months) would be sufficient to obtain 269 EFS events.<br><br>Accrual to NSABP B-59/GBG 96-GeparDouze began in December 2017 and was completed in May 2021 with a total of 1550 patients randomized. Based on actual accrual and the decision to eliminate pCR as a co-primary endpoint, we recalculated the power to detect a hazard ratio of 0.70 attributed to the addition of atezolizumab, assuming a lost-to-follow-up rate of 0.00083 per month, using the actual accrual pattern for the power calculation. With 1550 patients accrued in 42 months, an additional 22 months follow up will allow us to obtain 252 events under the assumptions stated above, which will provide 80% power to detect a HR of 0.7 between the atezolizumab and the placebo arm at an overall 2-sided alpha level of 0.05.
**Enrollment period**	It was anticipated that it would take about 34 months to accrue 1,520 patients. (Q-IV 2017 – Q-III 2020). The final enrollment period took 42 months to accrue 1,550 patients.
**Follow-up**	It was anticipated at the start of the study that follow-up of an additional 30 months after completion of accrual would be sufficient to obtain 269 EFS events (total study time of 64 months). With the final enrollment period and accrual information, follow-up of an additional 22 months after completion of accrual should be sufficient to obtain 252 EFS events (total time from first patient in to EFS analysis of 64 months).

Study design AC/EC q3 weekly

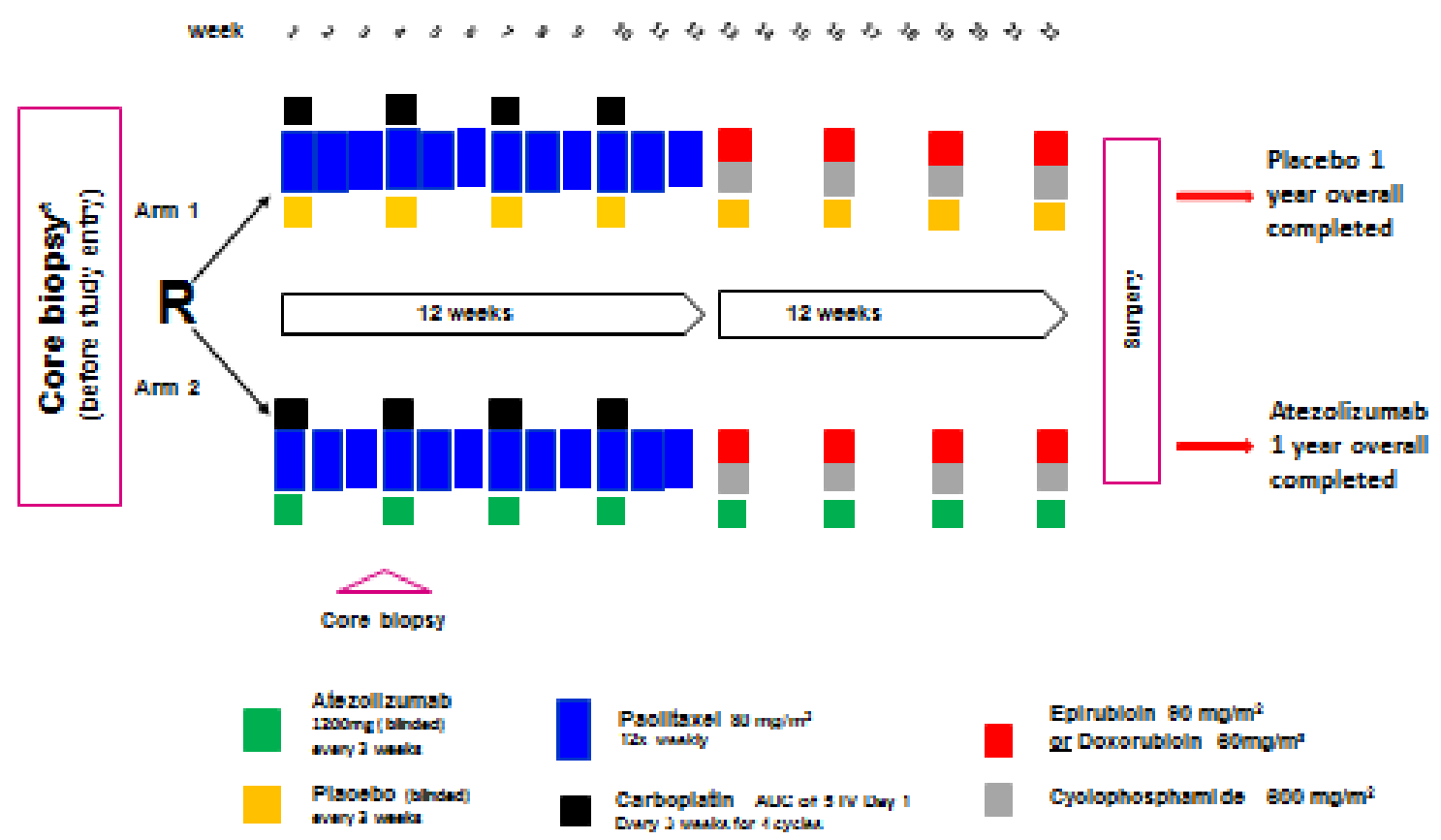

Study design AC/EC q2 weekly

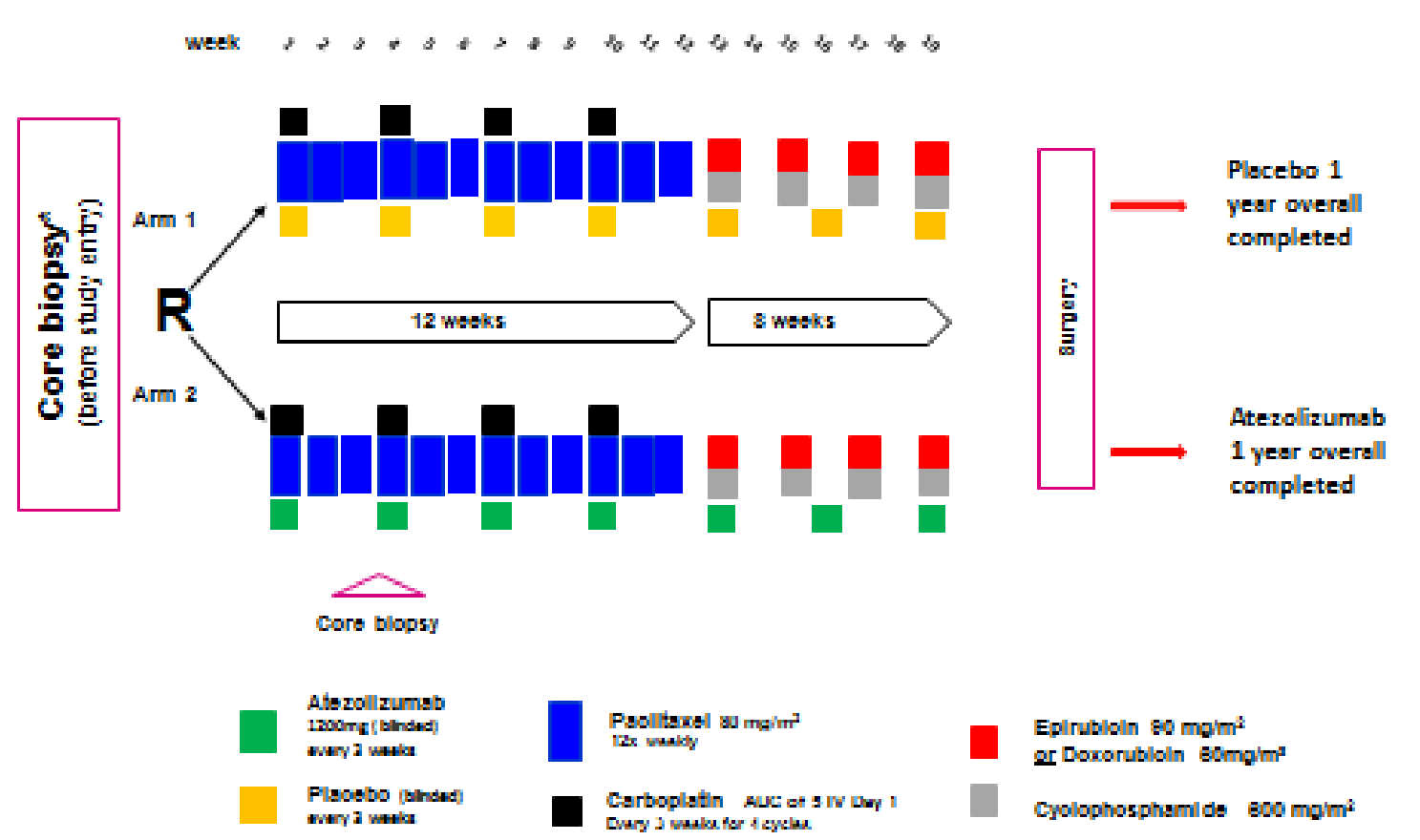

Figure 1.
NSABP B-59/GBG 96-GeparDouze Schema

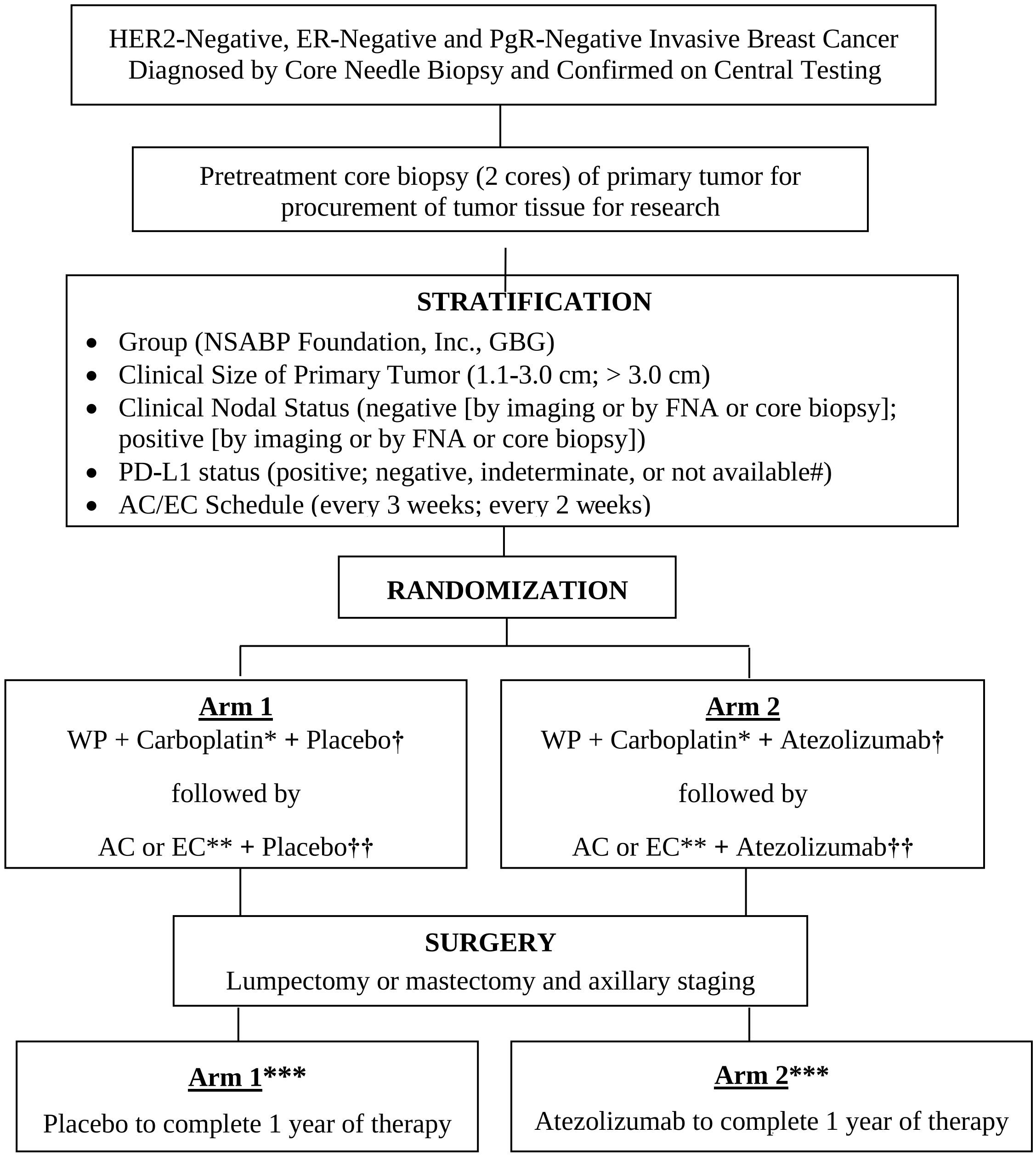

#	PD-L1 status of patients were not available at randomization for patients enrolled prior to amendments in July 2019.
*	Paclitaxel 80 mg/m^2 IV weekly x 12 doses (WP) + Carboplatin AUC of 5 IV Day 1 every 3 weeks for 4 cycles.
**	Doxorubicin (A) 60 mg/m^2 IV + cyclophosphamide (C) 600 mg/m^2 IV Day 1 every 2 or 3 weeks for 4 cycles **OR** Epirubicin (E) 90 mg/m^2 IV + cyclophosphamide (C) 600 mg/m^2 IV Day 1 every 2 or 3 weeks for 4 cycles. Choice of anthracycline and schedule per investigator discretion.
†	Atezolizumab 1200 mg or placebo IV Day 1 every 3 weeks for 4 doses.
††	Atezolizumab 1200 mg or placebo IV Day 1 every 3 weeks for 3 to 4 doses depending on AC/EC schedule used.
***	Atezolizumab 1200 mg or placebo IV Day 1 every 3 weeks after surgery until 1 year after the first dose.

2.0 BACKGROUND

Preoperative or neoadjuvant therapy, initially used for locally advanced breast cancers, has become more commonly employed for patients with operable disease, particularly those with larger primary tumors. By reducing the size of the primary tumor, preoperative therapy allows a higher rate of breast conserving surgery (Hortobagyi 1983, Hortobagyi 1988, Bonadonna 1990, Bonadonna 1998, Fisher 1997, Fisher 1998, Wolmark 2001, Bear 2003, Bear 2006, Rastogi 2008). The neoadjuvant setting also provides opportunities to obtain insights into breast cancer biology and accelerate evaluation of new therapies.

Use of neoadjuvant chemotherapy in operable breast cancer was evaluated in the National Surgical Adjuvant Breast and Bowel Project (NSABP) Protocol B-18 clinical trial in which 1,523 women with operable breast cancer received 4 cycles of AC (doxorubicin 60 mg/m^2, cyclophosphamide 600 mg/m^2) every 21 days either before or after definitive surgery (Fisher 1997, Fisher 1998, Wolmark 2001, Rastogi 2008). A pathologic complete response (pCR) in the breast was documented in 13% of patients, who had substantially improved outcomes relative to the group without pCR (disease-free survival [DFS] hazard ratio [HR]=0.47, $p < 0.0001$; overall survival [OS] HR=0.32, $p < 0.0001$). Pathologically negative nodes were identified in 58% of women receiving the neoadjuvant therapy compared with 42% among women undergoing initial surgery ($p < 0.0001$). A higher rate of breast conserving surgery was also observed in women receiving preoperative therapy (68% vs. 60%; p=0.001). There were no statistically significant differences in DFS (HR=0.93, p=0.27) and OS (HR=0.99, p=0.90) between the two groups.

The NSABP B-27 trial evaluated the incorporation of docetaxel in the neoadjuvant setting (Bear 2003, Bear 2006, Rastogi 2008). Women with operable breast cancer were randomized to receive either 1) preoperative AC followed by surgery, 2) preoperative AC followed by 4 cycles of preoperative docetaxel followed by surgery, or 3) preoperative AC followed by surgery followed by 4 cycles of postoperative docetaxel. Preoperative docetaxel following AC increased the pCR rate (13% to 26%, $p < 0.001$), and also, the proportion of women with negative nodes (51% to 58%, $p < 0.001$) relative to AC alone. While this group of women with pCR had a more favorable outcome (DFS HR=0.49, $p < 0.0001$ and OS HR=0.36, $p < 0.0001$), there were no statistically significant differences in DFS and OS demonstrated between the three treatment groups (the p across all arms = 0.76).

An international working group, in collaboration with the Food and Drug Administration (FDA), conducted a large meta-analysis of 11,955 patients treated in twelve randomized neoadjuvant chemotherapy trials with available long-term follow-up for DFS and OS. Improved outcomes in patients with pCR compared to those without were demonstrated (event-free survival HR=0.48, (95% CI, 0.43-0.54) and OS HR=0.36, (95% CI, 0.31-0.42). Moreover, the magnitude of the association between pCR and event free survival was greater in patients with aggressive breast cancer subtypes ([HR=0.49, 95% CI, 0.33-0.71 for hormone receptor +/HER2- and HR=0.58, 95% CI, 0.42-0.82 for hormone receptor +/HER2+] vs. [HR=0.24, 95% CI, 0.18-0.33 for TNBC and HR=0.25, 95% CI, 0.18-0.34 for hormone receptor-/HER2+]). However, correlation of improvement in long-term outcomes with increased pCR rates could not be established. This may have been due to the relatively low pCR rates reported in these early trials, the heterogeneity of the populations accrued, and lack of targeted therapy in the majority of patients (Cortazar 2014).

Triple-negative breast cancer (TNBC) is associated with worse outcomes compared to other subtypes of breast cancer. The risk of distant recurrence is substantially higher in the TNBC patient population (Dent 2007). The triple-negative phenotype is associated with relatively higher percentages of pCR following modern neoadjuvant chemotherapy, chemotherapy regimens

combining anthracyclines with cyclophosphamide and a taxane (approximately 30-40%), and patients with a pCR have a favorable prognosis. However, the majority of women with residual disease have a substantially higher risk of recurrence than women with other subtypes of breast cancer, so there remains a compelling need to identify additional therapies to increase the percentage of patients with pCR and improve long term outcomes (Cortazar 2014, Liedtke 2008, Carey 2007, Loibl 2017). A relatively mature avenue of research has been the incorporation of additional chemotherapeutic agents such as carboplatin. More recent approaches have been evaluating immune therapy with inhibitors of the PD-1/PD-L1 interaction in combination with chemotherapy.

One of these PD-1/PD-L1 inhibitors is atezolizumab, a humanized immunoglobulin (Ig) G1 monoclonal antibody that targets programmed death−ligand 1 (PD-L1) and inhibits the interaction between PD-L1 and its receptors, programmed death−1 (PD-1) and B7.1 (also known as CD80), both of which function as inhibitory receptors expressed on T cells. Therapeutic blockade of PD-L1 binding by atezolizumab has been shown to enhance the magnitude and quality of tumor-specific T-cell responses, resulting in improved anti-tumor activity (Fehrenbacher 2016, Rosenberg 2016). Atezolizumab has minimal binding to Fc receptors, thus eliminating detectable Fc-effector function and associated antibody-mediated clearance of activated effector T cells.

Atezolizumab shows anti-tumor activity in both nonclinical models and cancer patients and is being investigated as a potential therapy in a wide variety of malignancies. Atezolizumab is being studied as a single agent as well as in combination with chemotherapy, targeted therapy, and cancer immunotherapy.

Atezolizumab is approved in the United States for the treatment of locally advanced or metastatic urothelial carcinoma and metastatic non-small cell lung cancer (NSCLC) refractory to one prior treatment. Refer to the current Atezolizumab Investigator's Brochure for additional details on nonclinical and clinical studies.

2.1 Atezolizumab as monotherapy in metastatic triple-negative breast cancer

Atezolizumab (MPDL3280A) was initially evaluated in a cohort of 115 women with metastatic TNBC (Emens 2015, Schmid 2017). This included 63% of women initially selected for high PD-L1 expression levels (at least 5% of tumor-infiltrating immune cells were PD-L1 positive) and 33% of patients with TNBC regardless of PD-L1 expression. Four percent of patients had unknown PD-L1 expression levels. Efficacy was evaluated in 112 patients and safety was evaluated in 115 patients. PD-L1 expression was centrally evaluated on tumor-infiltrating immune cells based on an IHC assay. Atezolizumab was administered at 15 mg/kg, 20 mg/kg, or 1200 mg IV every 3 weeks. The objective response rate (ORR) was 10% (95% CI, 5-17) among the 112 evaluable patients. The one and two year overall survival rates for these responders were 100% and for non-responders, the overall survival rates were 33% and 11%, respectively. The median duration of response was 21 months (2.8 to 26.5+). Atezolizumab was generally well-tolerated. Treatment-related Grade 3 or 4 adverse events were reported in 11% of patients and adverse events leading to treatment discontinuation occurred in 3% of the cases. Two treatment related Grade 5 adverse events, pulmonary hypertension and death (unspecified), were reported in a hospitalized patient.

2.2 Safety data for atezolizumab with taxanes (with or without carboplatin) (Atezolizumab IB 2018)

As of 17 May 2018, approximately > 16,000 patients have been exposed to atezolizumab as monotherapy or in combination with chemotherapy and/or targeted therapy across a broad range of malignancies, including TNBC, HER2+ breast cancer, non-small cell lung cancer (NSCLC), urothelial bladder cancer, melanoma, renal cell carcinoma (RCC), gastric cancer, and colorectal cancer (CRC). Study GP28328 is an ongoing Phase Ib trial of atezolizumab in combination with bevacizumab and/or chemotherapy in patients with advanced solid tumors. As of the clinical cutoff date of 30 April 2017, there were 235 safety-evaluable patients enrolled across six treatment arms:

- Arm A: atezolizumab and bevacizumab in patients with multiple solid tumor types including CRC and RCC (n=90)
- Arm B: atezolizumab, bevacizumab, and mFOLFOX-6 in patients with metastatic solid tumors, metastatic CRC (n=24)
- Arm C: atezolizumab, carboplatin, and paclitaxel in patients with first-line (1L) NSCLC (n=25)
- Arm D: atezolizumab, carboplatin, and pemetrexed in patients with 1L NSCLC (n=25)
- Arm E: atezolizumab, carboplatin, and nab-paclitaxel in patients with 1L NSCLC (n=26)
- Arm F: atezolizumab and nab-paclitaxel in patients with 1L TNBC (n=33)

NSCLC Patients (Arms C, D, and E)

All 76 1L safety-evaluable patients with NSCLC (100%) in Arms C, D, and E reported an adverse event. The most commonly occurring AEs (≥ 25%) were fatigue (69.7%), nausea (59.2%), neutropenia (52.6%), diarrhea (44.7%), anemia (43.4%), constipation (39.5%), vomiting, decreased appetite, and alopecia (35.5% each), arthralgia (31.6%), cough and thrombocytopenia (28.9% each), and rash (27.6%). These AEs were consistent with the known toxicity profiles of atezolizumab and chemotherapy.

Sixty-four patients (84.2%) experienced a Grade 3-5 event, with the most frequently occurring (> 6.6% or ≥ 5 patients) being neutropenia (39.5%), anemia (22.4%), fatigue, (10.5%), dehydration, thrombocytopenia, and neutrophil count decreased (7.9% each), and diarrhea and febrile neutropenia (6.6% each). A third of the patients with NSCLC experienced atezolizumab-related Grade 3-5 AEs (29 patients [38.2%]). The most common of these AEs (≥ 6.6% or ≥ 5 patients) were fatigue (7.9%) and neutropenia (6.6%).

Two patients (2.6%) experienced a Grade 5 event, which occurred ≤ 30 days of discontinuing study treatment. These 2 events were pneumonia and systemic candida and were considered related to atezolizumab by the investigator.

Serious adverse events were observed in 47.4% of patients. Most of the events occurred in 1 patient each (1.3%), with the exception of dehydration (7.9%), pneumonia and neutropenia (6.6% each), febrile neutropenia (5.3%), pancreatitis (3.9%), and nausea, urinary tract infection, dyspnea, and fatigue (2.6% each). Serious adverse events that were assessed as related to atezolizumab occurred in 26.3% of the NSCLC patients, with the most common being pancreatitis, fatigue, febrile neutropenia, dehydration (all of which were reported in 2 patients [2.6%] each). Dyspnea, hypoxia, neutropenia, pancytopenia, thrombocytopenia, dehydration, and

alcoholism were the AEs (regardless of attribution) that led to discontinuation of atezolizumab in 7 of the NSCLC safety-evaluable patients (9.2%).

TNBC Patients (Arm F)

All 33 1L+ safety-evaluable patients with TNBC (100%) had an adverse event reported. The most commonly occurring AEs (≥ 30%) were fatigue (66.7%), diarrhea (51.5%), alopecia (42.4%), pyrexia and neutrophil count decreased (36.4% each), nausea, constipation, and cough (39.4% each), neutropenia (33.3%), and peripheral neuropathy (30.3%). These AEs were consistent with the known toxicity profiles of atezolizumab and nab-paclitaxel.

Over three-quarters of the patients with TNBC (84.8%) had a Grade 3-4 event. The Grade 3-4 events that occurred in > 1 patient (3%) were neutrophil count decreased (27.3%), neutropenia (18.2%), anemia and AST increased (9.1% each), and thrombocytopenia, diarrhea, pneumonia, sepsis, platelet count decreased, white blood cell count decreased, hyponatremia, and arthralgia (6.1% each). Twenty-four patients (72.7%) had a Grade 3-4 AE that was assessed by the investigator as related to atezolizumab. The Grade 3-4 atezolizumab-related events reported in more than 1 patient (3%) were neutrophil count decreased (3 patients [9.1%]), and pneumonia and thrombocytopenia (2 patients [6.1%]). There were no patients with TNBC who experienced a Grade 5 adverse event.

Seventeen TNBC patients (51.5%) experienced a serious adverse event; most events were single reports with the exception of 2 patients (6.1%), with Grade 3 sepsis, which was assessed as unrelated to atezolizumab. Seven patients (21.2%) had SAEs assessed as related to atezolizumab: pneumonia (2 patients; 6.1%), and diarrhea, pyrexia, pneumonia mycoplasmal, and anemia, each reported in one patient each. Two patients with TNBC discontinued atezolizumab due to an AE.

In summary, existing safety data for atezolizumab in combination with cytotoxic chemotherapy (including carboplatin + taxane) and/or bevacizumab (Study GP28328) in multiple tumor types, including 33 TNBC patients who received atezolizumab with nab-paclitaxel, suggest that atezolizumab can be safely combined with standard chemotherapy treatments. Atezolizumab in combination with cytotoxic chemotherapy has not been associated with significant additive severe (Grade 3 or higher) toxicities, and the AEs observed for atezolizumab in combination with chemotherapy are consistent with the known risks of each study treatment.

The randomized double-blind Phase 3 IMpassion130 (WO29522) study has completed enrollment for 900 TNBC patients (approximately 450 patients exposed to atezolizumab with nab-paclitaxel). The independent data monitoring committee (iDMC) regularly reviews unblinded safety data for this study and no new or significant safety concerns have been identified to date ([redacted]).

In addition, there is supportive safety data on the combination of atezolizumab with bevacizumab, paclitaxel, and carboplatin from the randomized, Phase III study IMPower150 (GO29436) in patients with 1L NSCLC. An iDMC meeting has reviewed safety data for approximately 400 patients total who received this combination and provided a positive recommendation to continue the study without any changes to study conduct ([redacted]).

The Impassion031 (WO39392) study in early TNBC evaluating atezolizumab in combination with neoadjuvant anthracycline/taxane-based chemotherapy, is a global Phase III, double-blind, randomized, placebo-controlled study designed to evaluate the efficacy and safety of neoadjuvant treatment with atezolizumab (840 mg) administered via intravenous (IV) infusion every 2 weeks

in combination with nab-paclitaxel (125 mg/m^2) administered via IV infusion every week for 12 weeks followed by atezolizumab (840 mg) administered every 2 weeks in combination with doxorubicin (60 mg/m^2) + cyclophosphamide (600 mg/m^2). To date, approximately 103 patients have been enrolled and 26 patients have received at least one dose of anthracycline + cyclophosphamide (with atezolizumab/placebo). An iDMC regularly evaluates unblinded safety data in this study ([redacted]).

[The following paragraph updating Impassion031 trial results was added in Amendment #5.]

Results of the trial were published in Lancet in October 2020, with 165 patients randomly assigned to receive atezolizumab plus chemotherapy and 168 to receive placebo plus chemotherapy. The study was designed with co-primary endpoints of pCR in the intent-to-treat (ITT) population and the PD-L1-positive population, with splitting of the alpha between the two co-primary endpoints. Pathological complete response (pCR) was documented in 58% (95% CI 50-65) of the ITT patient population in the atezolizumab plus chemotherapy group and 41% (95% CI 34-49) of the ITT patient population in the placebo plus chemotherapy group (rate difference 17%, 95% CI 6-27; one-sided p=0.0044 exceeding the significance boundary of 0.0184). In the PD-L1-positive subset, pCR was documented in 69% (95% CI 57-79) of patients in the atezolizumab plus chemotherapy group versus 49% (95% CI 38-61) of patients in the placebo plus chemotherapy group (rate difference 20%, 95% CI 4-35; one-sided p=0.021 exceeding the significance boundary 0.0184). Post-hoc exploratory analysis of pCR in the PD-L1 negative cohort was 48% in the atezolizumab plus chemotherapy group versus 34% in the placebo plus chemotherapy group (rate difference 13%, 95% CI 1-28). In the neoadjuvant phase, grade 3–4 adverse events were balanced between arms though treatment-related serious adverse events that occurred were increased with atezolizumab relative to placebo 23% versus 16% of patients. Immune-related adverse events were increased with atezolizumab but were infrequent with the exception of hypothyroidism which occurred in 7% versus 1% of patients (Mittendorf 2020).

Given the mechanism of action of atezolizumab, events associated with inflammation and/or immune-mediated AEs are closely monitored in the atezolizumab clinical program. The risk of potential overlapping toxicities (e.g., diarrhea, skin toxicity, pneumonitis/interstitial lung disease, cardiac toxicity) between atezolizumab and paclitaxel, carboplatin, and anthracycline + cyclophosphamide is thought to be minimal based on the mechanism of action of each product.

We do not anticipate an increased rate of cardiotoxicity with the combination of atezolizumab and AC following atezolizumab with carboplatin and paclitaxel, but will incorporate a safety lead-in study providing additional cardiac monitoring in the first 60 patients who initiate AC/EC, thus providing a mechanism to identify and further assess any cardiac toxicities that may occur with the combination of anthracyclines and atezolizumab.

2.3 Efficacy of atezolizumab with nab-paclitaxel in metastatic triple-negative breast cancer (IMpassion130)

IMpassion130 (WO29522) is a global, double-blind randomized Phase III clinical trial in which patients with untreated metastatic triple-negative breast cancer were randomly assigned in a 1:1 ratio to receive nab-paclitaxel with either atezolizumab or placebo until disease progression or an unacceptable toxicity occurred (Schmid 2018). Patients were stratified by 1) prior exposure or non-exposure to taxane chemotherapy in the neoadjuvant or adjuvant setting, 2) the presence or absence of liver metastases, and 3) programmed death ligand 1 (PD-L1) expression (positive vs. negative) on archival tumor tissue from original diagnosis of breast cancer or biopsy of the metastatic disease. PD-L1 positive status was defined as ≥ 1% staining of tumor-infiltrating immune cells as a percentage of tumor area using the SP142 Ventana assay. Co-primary end

points were progression-free survival (PFS) in the intention-to-treat (ITT) population and the PD-L1–positive subgroup, and overall survival (OS). The type I error (0.05) was controlled and split between the analyses of PFS (0.01) and OS (0.04), with hierarchical testing for OS first in the ITT population and then in the PD-L1-positive subgroup. The sample size would provide 95% power for the primary analysis of PFS among the ITT population and 88% power for analysis of OS.

Each group included 451 patients, and with a median follow-up of 12.9 months, median PFS in the overall ITT population of 902 patients was improved to 7.2 months with atezolizumab plus nab-paclitaxel, as compared with 5.5 months with placebo plus nab-paclitaxel (HR - 0.80; 95% CI, 0.69 - 0.92; P = 0.002). Among the subset of 369 patients with PD-L1-positive tumors, median PFS was 7.5 and 5.0 months, respectively (HR - 0.62; 95% CI, 0.49 - 0.78; P<0.001). However, among the subset of 533 patients with PD-L1-negative tumors, the HR for PFS was 0.95 (95% CI, 0.79-1.15). In the OS analysis of the overall ITT population, median OS was 21.3 months with atezolizumab plus nab-paclitaxel and 17.6 months with placebo plus nab-paclitaxel (HR - 0.84; 95% CI, 0.69 - 1.02; P = 0.08), which did not meet pre-specified criteria for significance, so a formal analysis of OS in the PD-L1 positive subset could not be performed. However among these patients, the median overall survival was 25.0 months with atezolizumab plus nab-paclitaxel vs. 15.5 months with placebo plus nab-paclitaxel (HR - 0.62; 95% CI, 0.45 to 0.86) (Schmid 2018). At the second interim analysis (data cutoff Jan 2, 2019), median follow-up was 18.5 months in the atezolizumab group and 17.5 months (8.4-22.4) in the placebo group. Median overall survival in the ITT population was 21.0 months (95% CI 19.0-22.6) with atezolizumab and 18.7 months (16.9-20.3) with placebo (stratified hazard ratio [HR] 0.86, 95% CI 0.72-1.02, p=0.078) (Schmid 2020).

The primary efficacy results of IMpassion130 demonstrate clinically relevant activity in patients with metastatic TNBC, and coupled with the identification of no new safety signals and an acceptable safety profile, provide important additional justification for the evaluation of the efficacy and safety of incorporating atezolizumab into standard neoadjuvant systemic therapy for localized triple negative breast cancer (Schmid 2018). Since analyses of PFS and OS by PD-L1 status in IMpassion130 demonstrated differential activity in PD-L1-positive and negative cohorts, NSABP B-59/GBG 96-GeparDouze will be amended to incorporate PD-L1 testing as a stratification variable (positive vs. negative or indeterminate) using the criteria and assay employed in IMpassion130.

2.4 Carboplatin as component of neoadjuvant chemotherapy in triple-negative breast cancer

Triple negative breast cancers are genomically unstable and may be more sensitive to DNA damaging drugs such as anthracyclines and carboplatin. The ABC trials demonstrated improved outcomes in patients with operable TNBC treated with adjuvant anthracyclines in combination with cyclophosphamide and a taxane relative to those treated with cyclophosphamide and a taxane without an anthracycline (Blum 2017). More recent efforts to evaluate the potential incremental efficacy and toxicity of incorporating carboplatin into standard anthracycline-based regimens in patients with Stage II and III TNBC have focused on the neoadjuvant setting since neoadjuvant administration of chemotherapy has become a preferred approach for these patients.

2.4.1 *German Breast Group GeparSixto*

The GeparSixto study evaluated the addition of neoadjuvant carboplatin to an anthracycline/taxane-containing regimen and targeted therapy in 595 patients with TNBC or HER2-positive breast cancer (von Minckwitz 2014). Patients received weekly neoadjuvant chemotherapy with paclitaxel (80 mg/m^2) and non-pegylated liposomal doxorubicin (20 mg/m^2) for 18 weeks (6 cycles). Patients were randomly assigned to

receive carboplatin AUC of 2 once every week for 18 weeks. The dose was reduced to AUC of 1.5 after an interim safety analysis (329 patients had been accrued). Patients with HER2-positive breast cancer also received trastuzumab 8 mg/kg initial dose with subsequent 6 mg/kg doses every 3 weeks and oral lapatinib 750 mg daily. Bevacizumab 15 mg/kg every 3 weeks was given simultaneously with all cycles to patients with TNBC. The primary endpoint of the study was to compare pCR rates (ypT0ypN0) between the two chemotherapy regimens.

The pCR rate was 36.9% (95% CI, 31.3-42.4) without carboplatin and 43.7% (95% CI, 38.1-49.4) with the addition of carboplatin (odds ratio 1.33; 95% CI, 0.96-1.85; p=0.107). The pCR rate among patients with TNBC was increased from 36.9% (95% CI, 29.4-44.5) in patients not receiving carboplatin to 53.2% (95% CI, 54.4-60.9) in patients receiving carboplatin (p=0.005). The increase in pCR was not seen for patients with HER2-positive breast cancer (36.8%, 28.7-44.9 without carboplatin vs. 32.8%, 25-40.7 with carboplatin; p=0.581). The test for interaction for carboplatin effect in patients with TNBC versus HER2-positive breast cancer was significant (p=0.015) (von Minckwitz 2014). In addition, the GeparSixto study found that germline *BRCA1/2* mutations and RAD mutations as well as family history of breast and/or ovarian cancer could not identify patients most likely to benefit from carboplatin (Hahnen 2017).

After a median follow-up of 3 years, patients with TNBC who received carboplatin had a significantly longer DFS 85.8% vs 76.1% than for those without (HR 0.5; 95%CI [0.33-0.96]; p=0.035 (Hahnen 2017). After a median follow-up of 47.3 months, patients with TNBC who received carboplatin had a significantly longer DFS 86.1% vs 75.8% than for those without (HR 0.56; 95%CI [0.34-0.93]; p=0.0244 (Loibl Ann Oncol 2018). However, since the control regimen employed in this trial did not contain cyclophosphamide, concerns have been raised that the improved outcomes could partly reflect the importance of inclusion of an alkylator in the treatment of TNBC rather than a specific additive benefit of carboplatin.

2.4.2 ***CALGB 40603***

The CALGB/Alliance 40603 study evaluated adding carboplatin to a more standard neoadjuvant chemotherapy regimen of weekly paclitaxel followed by dose-dense AC in TNBC. However, a 2x2 design was employed to also evaluate bevacizumab in this trial. A total of 454 patients with stage II or III TNBC received weekly paclitaxel (80 mg/m^2) for 12 doses followed by dose-dense AC. Randomization was to 1) receive paclitaxel followed by AC alone, 2) co-administration of bevacizumab 10 mg/kg every 2 weeks for nine cycles beginning with the first dose of paclitaxel, 3) administration of carboplatin AUC of 6 every 3 weeks for four cycles beginning with the first dose of paclitaxel, or 4) administration of both bevacizumab and carboplatin. The addition of carboplatin to this standard neoadjuvant chemotherapy regimen significantly increased pCR in the breast and axilla from 41% to 54% (OR: 1.71; p=0.0029).

Grade 3 and 4 neutropenia and thrombocytopenia were increased with the addition of carboplatin (Grade 3 or 4 neutropenia: 22% vs. 56% and thrombocytopenia 4% vs. 20%). Chemotherapy was discontinued early due to toxicity in 6% of patients receiving carboplatin (Sikov 2014, Sikov 2015). Of the patients receiving paclitaxel with carboplatin, 62% were able to receive 11-12 doses of weekly paclitaxel, but 23% received 9-10 doses, 4% received 7-8 doses, and 12% received less than or equal to six doses (Sikov 2014). The protocol specified that paclitaxel therapy should be

administered weekly for 12 weeks, and skipped doses were not made up. Thus, the incremental toxicity of carboplatin appeared to impact on the delivery of paclitaxel.

With follow-up, there was a trend toward improvement in 3-year EFS with the addition of carboplatin (HR: 0.84 [95% CI 0.58-1.22] p=0.36) with an EFS of 76% with carboplatin and 71% without carboplatin. However, the study was underpowered for demonstration of a statistical improvement in EFS, so the result does not exclude potential benefit from carboplatin.

2.4.3 *BrighTNess*

BrighTNess was a global, Phase III, randomized, double-blinded, placebo-controlled trial in patients with stage II-IIIA TNBC, evaluating the activity and safety of incorporating the PARP inhibitor, veliparib, with carboplatin into a standard regimen of weekly paclitaxel followed by AC (Loibl Lancet Oncol 2018). The study was designed to confirm the results of one of the I-SPY2 trials, which had demonstrated a high probability for a positive Phase III trial based on demonstration of a substantial improvement in pCR breast and nodes with addition of veliparib and carboplatin to paclitaxel prior to administration of dose-dense AC in patients with early stage TNBC (Nanda 2017). The I-SPY2 study had been designed to add the two agents to paclitaxel since preclinical and clinical studies had suggested veliparib was most effective in combination with carboplatin. BrighTNess was subsequently designed to also demonstrate the incremental benefits and toxicities of adding veliparib to carboplatin and paclitaxel by incorporating an additional control arm of carboplatin with paclitaxel followed by AC. In order to ensure optimal exposure to co-administration of veliparib and carboplatin on BrighTNess, a decision was made to allow the duration of paclitaxel administration to extend to 16 weeks.

Following stratification by *gBRCA* status (deleterious mutation vs no deleterious mutation vs unknown), clinical nodal stage (N0 vs. N1-2), and planned schedule of AC administration (q2wk vs q3wk per investigator discretion), 634 patients with stage II-III TNBC were randomized in a 2:1:1 ratio to one of three treatment arms:

Arm A (V+Cb+P): veliparib (50 mg orally BID daily) + carboplatin (AUC 6 mg/mL/min q3wk x 4) + paclitaxel (80 mg/m^2 weekly) x 12 doses→AC x 4

Arm B (Cb+P): veliparib placebo (BID daily) + carboplatin (AUC 6 mg/mL/min q3wk x 4) + paclitaxel (80 mg/m^2 weekly) x 12 doses→AC x 4

Arm C (P): veliparib placebo (BID daily) + carboplatin placebo (q3wk x 4) + paclitaxel (80 mg/m^2 weekly) x 12 doses→AC x 4

The primary aim was to assess the incidence of pCR in breast and ipsilateral axillary lymph nodes (ypT0/Tis, pN0) following neoadjuvant treatment with veliparib and carboplatin with paclitaxel (V+Cb+P) followed by AC (Arm A) and compare with each of two control regimens, Arm B (Cb+P→AC) and Arm C (P→AC) employing the appropriate placebo controls. Statistical significance was to be determined by using a two-sided p value of ≤ 0.05. For primary endpoint of pCR breast and nodes, the study had a > 80% power to detect significant treatment effects for both pair-wise comparisons (Arm A vs Arm B, and Arm A vs Arm C).

Secondary objectives were to compare Arm A vs Arm B, and Arm A vs Arm C for event free survival (EFS), overall survival (OS), safety, and the rate of eligibility for breast

conservation after therapy among subjects who were initially deemed ineligible at screening.

Results of the primary efficacy and safety analyses were initially reported at ASCO 2017, and have been published in Lancet Oncology (Loibl Lancet Oncol 2018). A significant improvement in pCR with the addition of carboplatin and veliparib to paclitaxel (Arm A) relative to paclitaxel alone (Arm C) (53.2% vs 31.0%, p<0.001) was demonstrated. However, the addition of veliparib to carboplatin and paclitaxel (Arm A) did demonstrate improvement in pCR compared to carboplatin and paclitaxel (Arm B) (53.2% vs 57.5%, p=0.36), indicating the improvement in pCR in Arm A was due to carboplatin, without contribution from veliparib at the dose and schedule chosen for the study. Pre-specified analyses by stratification factors demonstrated no differences in pCR by germline *BRCA* status or schedule of anthracycline administration. While the protocol did not pre-specify a formal comparison of Arm B with Arm C, the percentage of patients with pCR in the breast and nodes was 57.5% vs 31% with the addition of the 4 cycles of carboplatin to weekly paclitaxel. Secondary endpoints of EFS or OS have not yet been analyzed.

The modifications in the BrighTNess trial designed to ensure optimal exposure to veliparib and carboplatin by allowing up to 16 weeks to administer the 12 doses of weekly paclitaxel, appears to have effectively addressed the reduction in paclitaxel delivery reported in CALGB 40603. All 12 planned doses of paclitaxel were delivered in 88% of patients in Arm B (Cb+P), comparable to the 83.4% of patients in Arm C (P alone). Slightly more Arm B patients (38%) required at least 1 dose level reduction compared to Arm C (31%). The time to complete the paclitaxel-based portion of the treatment program in Arm B was also slightly prolonged relative to Arm C: 12-14 weeks (86.0% vs 98.8%), and 15-16 weeks (14.0% vs 1.2%).

Allowance of extension of the paclitaxel administration period to 16 weeks was also successful in achieving administration of 4 doses of carboplatin, as 92.4% of patients in Arm B received 4 doses comparable to 4 doses of the IV placebo in 87.9% of the Arm C patients. However, a majority of the Arm B patients (56.3%) required at least one dose reduction for carboplatin with 13.3% requiring two dose reductions. Importantly, incorporation of carboplatin did not substantively impact delivery of subsequent AC, as 94.9% of patients in Arm B received 4 cycles of AC comparable to 92.9% in Arm C following paclitaxel, though dose reductions of AC were required in 43.6% of patients in Arm B vs 34% in Arm C.

As expected, addition of carboplatin resulted in increased toxicities across both sequences of therapy, with 84.8% of patients in Arm B developing at least one Grade 3/4 toxicity compared to 45.2% in Arm C. Serious adverse events were increased from 14% to 26.6%, but there were no fatal AEs in Arm B or Arm C. Paclitaxel was discontinued early in 7% in Arm B and 2.5% in Arm C. Overall, the addition of carboplatin increased hematologic and GI toxicities, but only 1 patient developed febrile neutropenia during the carboplatin/paclitaxel in Arm B. However, febrile neutropenia was increased during AC in Arm B relative to Arm C (16.7% vs 5.0%). Of interest, reports of residual neuropathy during AC administration were less in Arm B than Arm C (6.4% vs 14.9%), suggesting the extended administration schedule may have had a salutary effect on reducing the incidence of the most problematic toxicity associated with weekly paclitaxel.

In summary, the results demonstrated a substantial increase in the percentage of patients with pCR breast and nodes with the addition of every-3-week carboplatin to weekly paclitaxel (31% vs 57.5%) in this population recognized to be at high risk for disease

recurrence in the absence of pCR. The modification to allow prolongation of the time to administer the paclitaxel improved the number of paclitaxel doses administered and allowed for administration of the majority of the planned doses of both carboplatin and AC, though at a cost of a slight reduction in dose intensity. The increase in overall Grade 3/4 toxicities was expected for the known toxicities of carboplatin, but the toxicities were manageable and did not result in any fatalities or substantially increase early discontinuations or dose reductions of the standard agents.

2.5 Rationale for the chemotherapy regimen

TNBC is associated with worse outcomes compared to other subtypes of breast cancer, as the risk of distant recurrence is substantially higher in this population (Dent 2007). Adjuvant chemotherapy regimens employing combinations of anthracyclines, cyclophosphamide, and taxanes have improved DFS and OS, but over one third of patients with Stages II and III breast cancer still recur and die with these regimens. Patients with Stage II and III disease who receive these regimens as neoadjuvant therapy have a low risk of recurrence if they are documented with a pCR in the breast and nodes following neoadjuvant therapy, but only one third of patients have a pCR after these therapies. Patients with residual TNBC have a substantially higher risk of recurrence than women with other subtypes of breast cancer who do not have a pCR (Cortazar 2014, Liedtke 2008, Carey 2007, Loibl 2017). Thus an unmet need remains to identify additional agents which can be combined with the standard three drug regimens which can substantially increase the percentage of patients with pCR, and thereby potentially improve long term outcomes.

Anthracyclines in combination with cyclophosphamide followed by monotherapy with a taxane is a common and prevalent approach to adjuvant and neoadjuvant therapy of TNBC. The sequence of administration of the segments can be reversed without impacting efficacy, and a regimen of weekly paclitaxel is generally well-tolerated from the hematologic and gastrointestinal toxicities. The most problematic potential toxicity is peripheral neuropathy, which is the most common toxicity causing early discontinuation and may persist following completion of therapy. However, the overall safety profile has provided an opportunity to evaluate the potential for incremental benefit from the addition of additional agents.

Both GeparSixto and CALGB 40603 were both designed to determine if the addition of carboplatin to anthracycline/taxane based regimens would increase pCR in patients with TNBC, and both studies demonstrated a statistically significant increase in pCR. Results of GeparSixto was tempered by the lack of an alkylator in the control group, and CALGB 40603 was complicated by the 2x2 design and reductions in the doses of paclitaxel administration due to design issues which kept the duration of therapy similar between the control arm and the investigational arm but at a cost of reduce doses delivered.

While BrighTNess was designed to isolate the incremental impact of veliparib added to carboplatin and paclitaxel if the 3 drug combination was shown to be superior to paclitaxel alone and ensure optimal exposure to veliparib and carboplatin, the results again demonstrated a substantial increase in pCR with the addition of carboplatin. Importantly, the results were achieved with an investigational program which added carboplatin to a standard neoadjuvant regimen that did not substantively compromise administration of the standard agents. Based on the strong association with pCR and long term outcomes in TNBC, the consistent improvement in pCR with the addition of carboplatin, the improved dose delivery and safety profile from the modification in BrighTNess, and the high risk population eligible for this trial, we are incorporating carboplatin into the chemotherapy regimen used for this trial. However, based on

the evidence that 56% of patients required at least 1 dose level reduction in the starting carboplatin dose, we will employ a starting dose of an AUC of 5 in this trial.

2.6 Rationale for atezolizumab dose and schedule

Atezolizumab/placebo will be administered at a fixed dose of 1200 mg Q3W by IV infusion with paclitaxel and carboplatin for 4-5 doses depending on the time required to complete the paclitaxel and carboplatin. Atezolizumab/placebo will then be administered at a fixed dose of 1200 mg Q3W by IV infusion for 3 to 4 doses aligned with the AC/EC schedule chosen by the investigator. Atezolizumab/placebo will be held during surgery and will be resumed following recovery as a fixed dose of 1200 mg Q3W to complete 1 year of therapy; administration of atezolizumab/placebo treatment will not be continued beyond 52 weeks after the first dose.

Anti-tumor activity has been observed across doses ranging from 1 mg/kg to 20 mg/kg Q3W. In Study PCD4989g, the maximum tolerated dose of atezolizumab was not reached and no dose-limiting toxicities were observed at any dose. The fixed dose of 1200 mg Q3W (equivalent to an average body weight−based dose of 15 mg/kg Q3W) was selected on the basis of both nonclinical studies (Deng 2016) and available clinical pharmacokinetic, efficacy, and safety data (refer to the Atezolizumab Investigator's Brochure for details).

2.7 Overview of proposed trial

In this randomized, double-blinded clinical trial of neoadjuvant and adjuvant administration of atezolizumab/placebo in patients with high risk TNBC, we will evaluate the potential incremental efficacy and safety of neoadjuvant administration of atezolizumab/placebo with a sequential regimen of weekly paclitaxel with every-3-week carboplatin followed immediately by neoadjuvant administration of atezolizumab/placebo with AC/EC. Patients will then undergo surgery. Following recovery from surgery, patients will initiate approximately 6 months of adjuvant therapy with atezolizumab/placebo and receive the same investigational agent they received pre-operatively. Administration of radiation therapy will be based on local standards at the discretion of patients and investigators, but if administered, atezolizumab/placebo will be administered concurrently. Adjuvant atezolizumab/placebo may be delayed until after completion of radiation therapy per investigator discretion.

Results of the I-SPY2 trial demonstrate addition of PD-1/PD-L1 interaction inhibitors co-administered with chemotherapy can increase pCR over chemotherapy alone. The phase 1b study of atezolizumab and nab-paclitaxel in patients with metastatic TNBC also demonstrated a high response rate. This study will employ a double-blinded/placebo controlled design to evaluate the efficacy and safety of incorporation of atezolizumab throughout the full administration of a sequential chemotherapy regimen of paclitaxel/carbo→AC/EC, which is an appropriate chemotherapy regimen for high risk, TNBC.

Following surgery, determination of pCR status and recovery from surgery, patients who did not discontinue atezolizumab/placebo due to toxicity during the neoadjuvant therapy will resume the original randomized investigational therapy assignment and continue the therapy as adjuvant treatment until 1 year after they received the initial dose of atezolizumab/placebo. Since activity of radiation therapy may also be augmented by inhibition of PD-1/PD-L1, radiation therapy, if indicated, should be co-administered with atezolizumab/placebo. This will allow for collection of safety data related to co-administration of atezolizumab with radiation therapy on a blinded, placebo-controlled trial.

The risk of potential overlapping toxicities between atezolizumab and carboplatin, paclitaxel, doxorubicin, and cyclophosphamide is thought to be minimal based on the mechanism of action

of each product and their respective toxicity profiles. In order to mitigate any potential risks associated with this combination regimen, patients with cardiopulmonary dysfunction will be excluded from the study, and an independent Data Safety Monitoring Board (DSMB) will regularly review unblinded safety data. Cardiac function will also be closely monitored, including regular measurements of left ventricular ejection fraction (LVEF) throughout the study.

Atezolizumab has been generally well tolerated. Immune-mediated adverse events are consistent with the role of the PD-L1/PD-1 pathway in regulating peripheral tolerance. Given the mechanism of action of atezolizumab, events associated with inflammation and/or immune-mediated adverse events are closely monitored across the atezolizumab clinical development program. Immune-mediated adverse events associated with atezolizumab include dermatologic, hepatic, endocrine, gastrointestinal, respiratory, and neurological events. To date, the majority of these events have been manageable, often without requiring treatment discontinuation.

This study will enroll patients with cT2−cT3 TNBC tumors who will receive neoadjuvant therapy prior to surgery. Given the relatively poor prognosis for these patients, the limited availability of targeted agents, and the ability to assess the benefit−risk profile in the neoadjuvant setting, this population and setting are considered appropriate for trials of novel therapies. The benefit-risk ratio for atezolizumab in this trial is expected to be acceptable in this setting for this patient population. The cardiac safety lead-in component of the study in the first 60 patients who begin AC/EC will incorporate assessment of a 12-lead ECG and serum troponin-T collected between completion of the administration of the 1st and 3rd dose of AC/EC and administration of the corresponding doses of atezolizumab/placebo administered on those days of treatment. Patients will also have a baseline troponin-T obtained just prior to administration of the first dose of doxorubicin or epirubicin. If troponin-T is found to be elevated, the patient will undergo evaluation by a cardiologist. All patients on this protocol will have LVEF assessments at baseline, after surgery, and 18 months from randomization. As a component of the extra monitoring in the first 60 patients in the cardiac safety lead-in, an additional assessment of LVEF with echocardiogram or MUGA scan will also be obtained prior to the 3rd dose of doxorubicin or epirubicin. Results of the troponin assessments, all available LVEF assessments, and cardiac safety data will be evaluated by the Data Safety Monitoring Board (DSMB) when the last of the initial 20 patients who initiate AC/EC undergo their scheduled post-surgery LVEF assessment. In addition, when the last of the first 60 patients to initiate AC/EC undergo their scheduled post-surgery LVEF assessment, results of the troponin assessments, LVEF assessments, and cardiac safety data from all 60 patients will be evaluated by the DSMB.

The cardiac monitoring plan will be re-assessed after each of the reviews of the cardiac safety data from the lead-in cohorts by the DSMB. If safety concerns are not identified by the DSMB, the additional monitoring comprising the cardiac safety lead-in will end after the first 60 patients and monitoring for the rest of the patient population will proceed as planned.

2.8 Allowance of capecitabine in patients with non-pCR or olaparib in patients with *gBRCA1* or *gBRCA2* mutations with non-pCR

In June 2017, Toi and colleagues in Japan and South Korea published results of the CREATE-X trial, which demonstrated improvement in both disease-free survival (HR - 0.58; 95% CI, 0.39 to 0.87), and OS (HR - 0.52; 95% CI, 0.30 to 0.90) with administration of 6-8 cycles of capecitabine in the subgroup of patients with residual invasive TNBC following neoadjuvant therapy with a sequential anthracycline-taxane regimen. This corresponds to an improvement in 5-year DFS from 56.1% to 69.8% and OS from 70.3 % to 78.8% with administration of the adjuvant capecitabine (Masuda 2017). Based on these results, the NCCN, ESMO, and St. Gallen guidelines have recommended that patients with TNBC and residual invasive cancer following

neoadjuvant therapy to be considered for treatment with adjuvant capecitabine (NCCN 2019, Cardoso 2019, Burstein 2019).

At the SABCS 2019, the German Breast Group presented results of a meta-analysis of more than 15,000 patients from 12 trials evaluating adjuvant capecitabine in early breast cancer. Seven of the trials evaluated capecitabine as an additional agent to standard therapies and five evaluated capecitabine as a substitute for another agent. In the trials evaluating the addition of capecitabine, the HR for DFS in TNBC patients was 0.82 (95% CI, 0.71 to 0.94) and 0.78 for OS (95% CI, 0.66 to 0.92) (van Mackelenbergh SABCS 2019). The findings support the demonstration of benefit from use of capecitabine as an additional agent in adjuvant therapy, but the magnitude of benefit was substantially less than reported in CREATE-X. Based on these data, this protocol is being amended to provide patients with residual invasive cancer at the time of surgery the option of receiving capecitabine concurrently with continuation of atezolizumab/placebo in the adjuvant setting. Since the use of capecitabine in non-pCR patients varies globally and is not consistently implemented into local guidelines or accepted by patients, capecitabine usage will be monitored and a protocol amendment to increase sample size will be considered prior to any analysis of the primary endpoints to compensate for potential reduction in events due to use of capecitabine.

[The following paragraph was added in Amendment #5.]

In June 2021, results of the global, randomized, phase III, placebo controlled OlympiA trial, which evaluated the efficacy and safety of olaparib versus placebo for one year following completion of systemic chemotherapy, surgery, and radiation therapy for HER2-negative high risk early breast cancer, were published with a median follow-up of 2.5 years. The 3-year invasive disease–free survival was 85.9% in the olaparib group and 77.1% in the placebo group (HR for invasive disease or death, 0.58; 99.5% CI, 0.41 to 0.82; P<0.001). The 3-year distant disease–free survival was 87.5% in the olaparib group and 80.4% in the placebo group (HR for distant disease or death, 0.57; 99.5% CI, 0.39 to 0.83; P<0.001). Olaparib was associated with fewer deaths than placebo (59 and 86, respectively) (HR, 0.68; 99% CI, 0.44 to 1.05; P = 0.02), but this difference was not significant at the pre-specified interim-analysis boundary of P < 0.01. Global quality of life was not different between olaparib and placebo, and the toxicity profile of olaparib was consistent with findings in metastatic disease. Based on these results, patients with non-pCR and *gBRCA* mutations may receive olaparib as alternative to capecitabine but will need to stop atezolizumab/placebo if receiving olaparib (Tutt 2021).

2.9 Study delivery model

This is a collaborative study being conducted by NSABP Foundation, Inc. in partnership with the German Breast Group (GBG), and supported by funding by [redacted] Two versions of a single protocol have been written to cover this collaborative neoadjuvant breast cancer study. One protocol version covers all patients recruited by GBG and the other protocol version covers patients recruited by NSABP Foundation, Inc. The two versions of the protocol are identical in terms of study objectives and scientific content and only differ in logistical content appropriate for the country(ies) they cover.

The trial will use one randomization list and be conducted as a single study. The initial collection and verification of the patients' data will be done using two databases (one for NSABP patients and one for GBG patients). The two databases will be established using the same standards and identical eCRFs to ensure consistency in the data collection. The data from both databases will be combined into a single consolidated database at the International Drug Development Institute (IDDI), at regular, pre-specified intervals. All study endpoints as well as reports for periodic review by the DSMB will be analyzed and reported from the single combined reporting database held by IDDI on behalf of NSABP Foundation, Inc.

3.0 STUDY AIMS AND ENDPOINTS

3.1 Primary aim and endpoint

3.1.1 *Event-free survival (EFS)*

Aim: To determine whether the addition of atezolizumab to chemotherapy (weekly paclitaxel plus carboplatin followed by AC *or* EC) followed by adjuvant atezolizumab improves event-free survival (EFS) in patients with triple-negative breast cancer.

Endpoint: EFS is defined as time from randomization until event: EFS events are progression on protocol therapy resulting in administration of non-protocol cancer therapy or inoperability, local invasive recurrence following mastectomy, local invasive recurrence in the ipsilateral breast following lumpectomy, regional recurrence, distant recurrence, contralateral invasive breast cancer, second non-breast primary cancer (excluding squamous or basal cell carcinoma of the skin), or death from any cause prior to recurrence or second primary cancer.

3.2 Secondary aims and endpoints

3.2.1 *Overall survival (OS)*

Aim: To determine whether the addition of atezolizumab to chemotherapy (weekly paclitaxel plus carboplatin followed by AC *or* EC) followed by adjuvant atezolizumab improves overall survival (OS) in patients with triple-negative breast cancer.

Endpoint: Defined as time from randomization until death from any cause.

3.2.2 *Pathologic complete response in the breast and lymph nodes (ypT0/Tis ypN0)*

Aim: To determine whether the addition of atezolizumab to chemotherapy (weekly paclitaxel plus carboplatin followed by AC *or* EC) improves pathologic complete response in the breast and post-therapy lymph nodes evaluated histologically (pCR breast and nodes) in patients with triple-negative breast cancer.

Endpoint: Defined as the absence of any invasive component in the resected breast specimen and all resected lymph nodes following completion of neoadjuvant therapy (ypT0/Tis ypN0).

3.2.3 *Distant disease-free survival (DDFS)*

Aim: To determine whether the addition of atezolizumab to chemotherapy (weekly paclitaxel plus carboplatin followed by AC *or* EC) followed by adjuvant atezolizumab improves distant disease-free survival (DDFS) in patients with triple-negative breast cancer.

Endpoint: Defined as time from randomization until distant recurrence, death from breast cancer, death from other causes, and second primary invasive cancer (non-breast).

3.2.4 *Disease-free survival (DFS)*

Aim: To determine whether the addition of atezolizumab to chemotherapy (weekly paclitaxel plus carboplatin followed by AC or EC) followed by adjuvant atezolizumab improves disease-free survival (DFS) in patients with triple-negative breast cancer.

Endpoint: Defined as time from first breast surgical procedure (operation) to the first occurrence of disease recurrence or death from any cause. Events defining DFS are ipsilateral invasive breast tumor recurrence, ipsilateral local-regional invasive breast

cancer recurrence, distant recurrence, contralateral invasive breast cancer, ipsilateral or contralateral DCIS, second primary non-breast invasive cancer (with the exception of non-melanoma skin cancers and non-breast in situ carcinoma of any site) and death attributable to any cause including breast cancer, non-breast cancer, or unknown cause.

3.2.5 ***Toxicity***

Aim: To evaluate toxicity associated with study therapy added to chemotherapy and radiation therapy.

Aim: To evaluate immune-adverse events of special interest in Section 10.4.3.

Endpoint: Frequency and severity of adverse events graded according to the NCI Common Terminology Criteria for Adverse Events version 4.0 (CTCAE v4.0).

3.2.6 ***Cardiac safety lead-in study***

Aim: To assess for potential augmentation of anthracycline-related cardiac toxicity with co-administration of study therapy.

Endpoints: Troponin-T levels 1) prior to initial dose of AC/EC following completion of carboplatin/paclitaxel co-administered with atezolizumab/placebo and after administration of the 1st and 3rd cycles of AC/EC prior to administration of atezolizumab/placebo.

LVEF levels at 1) baseline prior to initiation of carboplatin/paclitaxel and atezolizumab/placebo, 2) before 3rd cycle of AC/EC, and 3) after surgery.

3.3 **Exploratory aims and endpoints**

3.3.1 ***Pathologic complete response in the breast (ypT0/Tis)***

Aim: To determine whether the addition of atezolizumab to chemotherapy (weekly paclitaxel plus carboplatin followed by AC *or* EC) improves pathologic complete response in the breast (pCR breast) in patients with triple-negative breast cancer.

Endpoint: Defined as the absence of any invasive component in the resected breast specimen (nodal material not considered).

3.3.2 ***Pathologic complete response in the breast and lymph nodes (ypT0 ypN0)***

Aim: To determine whether the addition of atezolizumab to chemotherapy (weekly paclitaxel plus carboplatin followed by AC *or* EC) improves pathologic complete response in the breast and post-therapy lymph nodes evaluated histologically (pCR breast and nodes) in patients with triple-negative breast cancer.

Endpoint: Defined as the absence of any invasive component or DCIS in the resected breast specimen and all resected lymph nodes following completion of neoadjuvant therapy.

3.3.3 ***Positive nodal status conversion rate***

Aim: To determine whether the addition of atezolizumab to chemotherapy (weekly paclitaxel plus carboplatin followed by AC or EC) improves conversion rate from clinically node-positive to pathologically node-negative.

Endpoint: Percentage of patients clinically node-positive that convert to pathologically node-negative following completion of neoadjuvant chemotherapy.

3.3.4 ***Recurrence-free interval (RFI)***

Aim: To determine whether the addition of atezolizumab to chemotherapy (weekly paclitaxel plus carboplatin followed by AC *or* EC) followed by adjuvant atezolizumab improves recurrence-free interval (RFI) in patients with triple-negative breast cancer.

Endpoint: Defined as time from randomization until invasive local, regional, or distant recurrence, or death from breast cancer (censored for death from other causes).

3.3.5 ***Brain metastases free survival***

Aim: To determine whether the addition of atezolizumab to chemotherapy (weekly paclitaxel plus carboplatin followed by AC *or* EC) followed by adjuvant atezolizumab improves brain metastases free survival in patients with triple-negative breast cancer.

Endpoint: Defined as time from randomization until documentation of first event brain metastasis or death from any cause. Patients who develop locoregional or distant recurrence outside of the central nervous system as first event will continue to be followed for subsequent development of brain metastases.

3.3.6 ***Pathologic complete response in the breast and lymph nodes (ypT0/Tis ypN0) in patients with deleterious germline BRCA mutation status***

Aim: To determine whether the addition of atezolizumab to chemotherapy (weekly paclitaxel plus carboplatin followed by AC *or* EC) improves pathologic complete response in the breast and post-therapy lymph nodes evaluated histologically (pCR breast and nodes [ypT0/Tis ypN0]) in patients with deleterious germline *BRCA* mutation status.

3.3.7 ***Event-free survival (EFS) in patients with deleterious germline BRCA mutation status***

Aim: To determine whether the addition of atezolizumab to chemotherapy (weekly paclitaxel plus carboplatin followed by AC *or* EC) followed by adjuvant atezolizumab improves event-free survival (EFS) in patients with deleterious germline *BRCA* mutation status.

3.4 **Correlative science aims and endpoints**

Aim: To evaluate baseline expression of PD-L1 and expression of PD-L1 following initial dose of atezolizumab with paclitaxel plus carboplatin as predictor for pCR of breast and nodes (ypT0/Tis ypN0) following neoadjuvant administration of atezolizumab with chemotherapy

Endpoint: pCR of breast and nodes (ypT0/Tis ypN0) as defined in Section 3.2.2.

Aim: To evaluate baseline expression of PD-L1 and expression of PD-L1 following initial dose of atezolizumab with paclitaxel plus carboplatin as predictor for EFS following neoadjuvant administration of atezolizumab with chemotherapy followed by adjuvant administration of atezolizumab

Endpoint: EFS as defined in Section 3.1.1.

Aim: To evaluate baseline percentages of TILs and percentage of TILs following initial dose of atezolizumab with paclitaxel plus carboplatin as predictor for pCR of breast and nodes following neoadjuvant administration of atezolizumab with chemotherapy

Endpoint: pCR of breast and nodes (ypT0/Tis ypN0) as defined in Section 3.2.2.

Aim: To evaluate baseline percentages of TILs and percentage of TILs following initial dose of atezolizumab with paclitaxel plus carboplatin as predictor for EFS following neoadjuvant

administration of atezolizumab with chemotherapy followed by adjuvant administration of atezolizumab

Endpoint: EFS as defined in Section 3.1.1.

Aim: To evaluate percentages of TILs in patients with residual breast cancer at time of surgery following neoadjuvant administration of atezolizumab with chemotherapy followed by adjuvant administration of atezolizumab as predictor for EFS.

Endpoint: EFS as defined in Section 3.1.1.

Aim: To use baseline and on therapy specimens, such as plasma ctDNA, to explore potential new biomarkers of responses and resistance to neoadjuvant administration of atezolizumab with chemotherapy followed by adjuvant administration of atezolizumab.

4.0 PATIENT ELIGIBILITY AND INELIGIBILITY

Investigators should consider each of these factors when selecting patients for this trial. Investigators should also consider all other relevant factors (medical and non-medical), as well as the risks and benefits of the study therapy, when deciding if a patient is an appropriate candidate for this trial.

- Study therapy should begin within 2 weeks after randomization and will continue for 1 year total of study therapy (inclusive of neoadjuvant and adjuvant therapy). Discussion should include patient availability and the timing of study therapy.
- ***Submission of samples from the breast surgery if there is residual tumor measuring ≥ 1 cm is required for all patients*** (see Section 6.1). The local country and pathology department policies regarding release of blocks must be considered when screening patients.
- *BRCA* mutation testing ***is not required*** for entry in the NSABP B-59/GBG 96-GeparDouze study; however, *BRCA* mutation status (positive; negative, unknown) will be collected at entry. If *BRCA* mutation testing has not been performed, record the *BRCA* mutation status as "Unknown." If *BRCA* mutation status becomes available subsequent to entry, the *BRCA* mutation status should be updated.

4.1 Conditions for patient eligibility

A patient cannot be considered eligible for this study unless all of the following conditions are met:

4.1.1 The patient must have consented to participate and, prior to beginning specific study procedures, must have signed and dated an appropriate IRB-approved consent form that conforms to federal and institutional guidelines for study treatment and for submission of tumor samples from a research biopsy as required by NSABP B-59/GBG 96-GeparDouze for baseline correlative science studies (see Section 6.0).

4.1.2 The diagnosis of invasive adenocarcinoma of the breast must have been made by core needle biopsy.

4.1.3 Local testing on the diagnostic core must have determined the tumor to be ER-negative, PgR-negative, and HER2-negative by current ASCO/CAP guidelines. (If local testing has determined a tumor to be HER2 equivocal or to have a borderline ER/PgR status (% IHC staining < 10% for both) and other eligibility criteria are met, material may be submitted for central testing to determine eligibility.)

4.1.4 Central testing for ER, PgR, and HER2 will be performed, and the tumor must be determined to be ER-negative, PgR-negative, and HER2-negative by current ASCO/CAP Guidelines Recommendations. Material from either the diagnostic core biopsy or the research biopsy can be used for central testing depending on local preferences and standards.

4.1.5 The tumor specimen used for central ER, PgR, and HER2 testing must also be used for central testing of PD-L1 status using the Ventana PD-L1 (SP142) assay kit. Patients will be eligible irrespective of PD-L1 testing result including PD-L1 indeterminate. Patients will be classified as positive, negative, or indeterminate for stratification purposes.

4.1.6 Patients must be ≥ 18 years old.

4.1.7 Patients may be female or male.

4.1.8 The ECOG performance status must be 0-1 (see Appendix A).

4.1.9 The primary tumor can be clinical stage T2 or T3, if clinically node negative according to AJCC 7th Edition. If the regional lymph nodes are cN1 and cytologically or histologically positive or cN2–N3 with or without a biopsy, the primary breast tumor can be clinically T1c, T2, or T3.

4.1.10 Ipsilateral axillary lymph nodes must be evaluated by imaging (ultrasound or MRI) within 42 days prior to study entry. If suspicious or abnormal, FNA or core biopsy is recommended. Findings of these evaluations will be used to define the nodal status prior to study entry according to the following criteria:

- Nodal status – negative
  - Imaging of the axilla is negative;
  - Imaging is suspicious or abnormal but the FNA or core biopsy of the questionable node(s) on imaging is negative;
- Nodal status – positive
  - FNA or core biopsy of the node(s) is cytologically or histologically suspicious or positive.
  - Imaging is suspicious or abnormal but FNA or core biopsy was not performed.

4.1.11 Patients with synchronous bilateral or multicentric HER2-negative breast cancer are eligible as long as the highest risk tumor is ER-negative and PgR-negative and meets stage eligibility criteria. All of the other invasive tumors must also be HER2-negative by ASCO/CAP Guidelines based on local testing. Central testing to confirm TNBC status is only required for the highest risk tumor.

4.1.12 Blood counts performed within 28 days prior to randomization must meet the following criteria:

- ANC must be ≥ 1500/mm^3;
- platelet count must be ≥ 100,000/mm^3; and
- hemoglobin must be ≥ 10 g/dL.

4.1.13 The following criteria for evidence of adequate hepatic function performed within 28 days prior to randomization must be met:

- total bilirubin must be ≤ ULN for the lab unless the patient has a bilirubin elevation > ULN to 1.5 x ULN due to Gilbert's disease or similar syndrome involving slow conjugation of bilirubin; *and*
- alkaline phosphatase must be ≤ 2.5 x ULN for the lab; *and*
- AST and ALT must be ≤ 1.5 x ULN for the lab.

4.1.14 Patients with AST or ALT or alkaline phosphatase > ULN are eligible for inclusion in the study if liver imaging (CT, MRI, abdominal ultrasound, PET-CT, or PET scan) performed within 28 days prior to randomization does not demonstrate metastatic disease and the requirements in criterion 4.1.13 are met.

4.1.15 Patients with alkaline phosphatase that is > ULN but ≤ 2.5 x ULN or with unexplained bone pain are eligible for inclusion in the study if bone imaging (bone scan, PET-CT scan, or PET scan) supported by additional studies when indicated (CT, x-ray, MRI) performed within 42 days prior to randomization does not demonstrate metastatic disease.

4.1.16 Creatinine clearance ≥ 50 mL/min (see Section 7.2.1 for instructions regarding calculation of creatinine clearance) performed within 28 days prior to randomization.

4.1.17 PT/INR ≤ ULN within 28 days prior to randomization. For laboratories that do not report an ULN for the INR assay, use ≤ 1.2 as the value for the ULN. Patients receiving therapeutic anti-coagulants are not eligible.

4.1.18 A serum TSH and AM (morning) cortisol performed within 28 days prior to randomization to obtain a baseline level. Patients with abnormal TSH or AM cortisol baseline levels should be further evaluated and managed per institutional standards. Asymptomatic patients who require initiation or adjustment of medication or are followed without initiating treatment based on endocrinologist's recommendations are eligible.

4.1.19 LVEF assessment must be performed within 42 days prior to randomization. (LVEF assessment performed by echocardiogram is preferred; however, MUGA scan may be substituted based on institutional preferences.) The LVEF must be ≥ 55% regardless of the cardiac imaging facility's lower limit of normal.

4.1.20 For women of childbearing potential: agreement to remain abstinent (refrain from heterosexual intercourse) or use contraceptive methods that result in a failure rate of < 1% per year during the treatment period and for at least 5 months after the last dose of atezolizumab/placebo or 12 months after the last dose of chemotherapy.

A woman is considered to be of childbearing potential if she is not postmenopausal, has not reached a postmenopausal state (≥ 12 continuous months of amenorrhea with no identified cause other than menopause), and has not undergone surgical sterilization (removal of ovaries and/or uterus).

Examples of contraceptive methods with a failure rate of < 1% per year include: bilateral tubal ligation; male partner sterilization; intrauterine devices.

The reliability of sexual abstinence should be evaluated in relation to the duration of the clinical study and the preferred and usual lifestyle of the patient. Periodic abstinence (e.g., calendar, ovulation, symptothermal, or postovulation methods) and withdrawal are not acceptable methods of contraception.

4.1.21 Patient must be willing and able to comply with scheduled visits, treatment plans, laboratory tests, and other study procedures.

4.1.22 Patients with N2 or N3 nodal disease or T3 primary disease must undergo liver imaging within 28 days prior to randomization and bone imaging (as described in 4.1.14 and 4.1.15) within 42 days prior to randomization, irrespective of baseline lab results, and studies must not demonstrate metastatic disease. Chest imaging with chest x-ray PA and Lateral, CT of the chest, or PET-CT must also be performed.

4.2 Conditions for patient ineligibility

Any patient with one or more of the following conditions will be ineligible for this study:

4.2.1 Excisional biopsy or lumpectomy performed prior to study entry.

4.2.2 FNA alone to diagnose the breast cancer.

4.2.3 Surgical axillary staging procedure prior to randomization. Exception: FNA or core biopsy of an axillary node is permitted for any patient. A pre-neoadjuvant therapy sentinel lymph node biopsy for patients with clinically negative axillary nodes is prohibited.

4.2.4 Definitive clinical or radiologic evidence of metastatic disease.

4.2.5 Previous history of contralateral invasive breast cancer. (Patients with synchronous and/or previous contralateral DCIS or LCIS are eligible.)

4.2.6 Previous history of ipsilateral invasive breast cancer or ipsilateral DCIS. (Patients with synchronous or previous ipsilateral LCIS are eligible.)

4.2.7 History of non-breast malignancies (except for in situ cancers treated only by local excision and basal cell and squamous cell carcinomas of the skin) within 5 years prior to study entry.

4.2.8 Treatment including radiation therapy, chemotherapy, or targeted therapy, for the currently diagnosed breast cancer prior to randomization.

4.2.9 Previous therapy with anthracyclines or taxanes for any malignancy.

4.2.10 Cardiac disease (history of and/or active disease) that would preclude the use of the drugs included in the treatment regimens. This includes but is not confined to:

Active cardiac disease:

- angina pectoris that requires the use of anti-anginal medication;
- ventricular arrhythmias except for benign premature ventricular contractions;
- supraventricular and nodal arrhythmias requiring a pacemaker or not controlled with medication;
- conduction abnormality requiring a pacemaker;
- valvular disease with documented compromise in cardiac function; or
- symptomatic pericarditis.

History of cardiac disease:

- myocardial infarction documented by elevated cardiac enzymes or persistent regional wall abnormalities on assessment of left ventricular function within 6 months prior to randomization;
- history of documented CHF; or
- documented cardiomyopathy.

4.2.11 Uncontrolled hypertension defined as sustained systolic BP $>$ 150 mmHg or diastolic BP $>$ 90 mmHg. (Patients with initial BP elevations are eligible if initiation or adjustment of BP medication lowers pressure to meet entry criteria.) Patients requiring $\geq$ 3 BP medications are not eligible.

4.2.12 History of severe allergic, anaphylactic, or other hypersensitivity reactions to chimeric or humanized antibodies or fusion proteins.

4.2.13 Known hypersensitivity to biopharmaceuticals produced in Chinese hamster ovary cells.

4.2.14 Known allergy or hypersensitivity to the components of the atezolizumab formulation.

4.2.15 Known allergy or hypersensitivity to the components of the doxorubicin, epirubicin, cyclophosphamide, carboplatin, or paclitaxel formulations.

4.2.16 Known allergy or hypersensitivity to liposomal or pegylated G-CSF formulations.

4.2.17 Active or history of autoimmune disease or immune deficiency, including but not limited to myasthenia gravis, myositis, autoimmune hepatitis, systemic lupus erythematosus, rheumatoid arthritis, inflammatory bowel disease, antiphospholipid syndrome, Wegener granulomatosis, Sjögren syndrome, Guillain-Barré syndrome, or multiple sclerosis (see

Appendix B) for a more comprehensive list of autoimmune diseases and immune deficiencies) with the following exceptions:

- Patients with a history of autoimmune-related hypothyroidism on a stable dose of thyroid replacement hormone may be eligible for this study.
- Patients with controlled Type 1 diabetes mellitus on a stable dose of insulin regimen may be eligible for this study.
- Patients with eczema, psoriasis, lichen simplex chronicus, or vitiligo with dermatologic manifestations only (e.g., patients with psoriatic arthritis are excluded) are permitted provided all of following conditions are met:
  - Rash must cover < 10% of body surface area.
  - Disease is well controlled at baseline and requires only low-potency topical corticosteroids.
  - No occurrence of acute exacerbations of the underlying condition requiring psoralen plus ultraviolet A radiation, methotrexate, retinoids, biologic agents, oral calcineurin inhibitors, or high-potency or oral corticosteroids within the previous 12 months.

4.2.18 History of idiopathic pulmonary fibrosis, organizing pneumonia (e.g., bronchiolitis obliterans), drug-induced pneumonitis, idiopathic pneumonitis, or evidence of active pneumonitis on screening chest CT scan.

4.2.19 Patients known to be HIV positive.

4.2.20 Active hepatitis B virus (HBV) infection, defined as having a positive hepatitis B surface antigen (HBsAg) test at screening.

Patients with a past or resolved HBV infection, defined as having a negative HBsAg test and a positive total hepatitis B core antibody (HBcAb) test at screening, are eligible for the study if active HBV infection is ruled out on the basis of HBV DNA viral load per local guidelines.

4.2.21 Active hepatitis C virus (HCV) infection, defined as having a positive HCV antibody test at screening confirmed by a polymerase chain reaction (PCR) positive for HCV RNA.

4.2.22 Patients with clinically active tuberculosis.

4.2.23 Severe infection within 28 days prior to randomization, including but not limited to hospitalization for complications of infection, bacteremia, or severe pneumonia.

4.2.24 Prior allogeneic stem cell or solid organ transplantation.

4.2.25 Administration of a live, attenuated vaccine within 28 days prior to randomization or anticipation that such vaccine will be required during the study.

Patients must agree not to receive live, attenuated influenza vaccine (e.g., FluMist®) within 28 days prior to randomization, during treatment or within 5 months following the last dose of atezolizumab/placebo.

4.2.26 Any other disease, metabolic dysfunction, physical examination finding, or clinical laboratory finding giving reasonable suspicion of a disease or condition that contraindicates the use of an investigational drug or that may affect the interpretation of the results or render the patient at high risk from treatment complications.

4.2.27 Prior treatment with CD137 agonists or immune checkpoint-blockade therapies, including anti-CD40, anti-CTLA-4, anti-PD-1, and anti-PD-L1 therapeutic antibodies.

4.2.28 Treatment with systemic immunosuppressive medications (including but not limited to interferons, IL-2) within 28 days or 5 half-lives of the drug, whichever is longer, prior to randomization.

4.2.29 Treatment with systemic immunosuppressive medications (including but not limited to prednisone, cyclophosphamide, azathioprine, methotrexate, thalidomide, and anti-tumor necrosis [anti-TNF] factor agents) within 14 days prior to randomization or anticipation of need for systemic immunosuppressive medications during the study.

4.2.30 Nervous system disorder (paresthesias, peripheral motor neuropathy, or peripheral sensory neuropathy) ≥ Grade 2, per the CTCAE v4.0.

4.2.31 Symptomatic peripheral ischemia.

4.2.32 Pregnancy or lactation at the time of randomization or intention to become pregnant during the study. (Note: Negative serum pregnancy test must be obtained within 14 days prior to randomization).

4.2.33 Use of any investigational agent within 28 days prior to randomization.

5.0 REQUIREMENTS FOR STUDY ENTRY AND DURING TREATMENT AND FOLLOW-UP

Tests and exams required before randomization are listed on Table 1. Requirements following randomization are listed on Table 2. Additional requirements for initial 60 patients who begin AC/EC in the Cardiac Safety Lead-in study are listed on Table 3.

Table 1. Tests, exams, and other requirements prior to randomization

Requirements	Prior to Randomization[a]	
Consent form signed by the patient	X	
Primary tumor tissue from research biopsy (see Section 6.0)		
Central determination of HER2, hormone receptor status, and PD-L1 status on diagnostic biopsy or research biopsy (see Section 4.1.4 and Section 4.1.5)[b]		
Determination of nodal status for stratification (see Sections 4.1.10 and 4.2.3)		
Tumor assessment and measurement[c]		
Menopausal status		
History & physical exam[d]	X	Within 28 days
Assessment of concomitant medications[e]	X	
Performance status (see Appendix A)	X	
Height & weight	X	
CBC/differential/platelet count	X	
Total bilirubin/AST/ALT/alkaline phosphatase	X	
Serum chemistry: creatinine, sodium, potassium, chloride, carbon dioxide, calcium, glucose, total protein, albumin	X	
Creatinine clearance	X	
TSH, AM (morning) Cortisol[f]	X	
PT/INR	X	
Hepatitis B Surface Antigen, Hepatitis B Core Antibody, Hepatitis C Antibody[g]	X	
Serum pregnancy test[h]	X	Within 14 days
Echocardiogram (or MUGA scan)[i]	X	Within 42 days
ECG	X	Within 28 days
Chest imaging[j]	X	
Liver imaging[k]	X	
Nuclear bone imaging[l]	X	Within 42 days
Ipsilateral breast (mammogram or MRI)[m] and axillary lymph nodes imaging (ultrasound or MRI)[n]	X	Within 42 days
Contralateral breast imaging (mammogram or MRI)[m]	X	Within 180 days
Marking of primary tumor site[o]	X	**Before or after randomization** (before therapy begins)

Table continued on next page.

Table 1. Tests, exams, and other requirements prior to randomization *(continued)*

**Requirements**	**Prior to Randomization**[a]	
Mandatory collection of plasma sample for ctDNA[p]	X	**Before or after randomization** (before therapy begins)

a Informed consent must be obtained prior to the performance of any screening assessments; however, results of screening tests or examinations performed as standard of care prior to obtaining informed consent but within the timeframes outlined in Table 1 may be used rather than repeating required tests.

b Material from either the diagnostic core biopsy or the research biopsy can be used for central testing depending on local preferences and standards. The tumor tissue must be submitted ***before study entry*** for central confirmation of TNBC status. See Section 6.0 and the ***NSABP B-59/ GBG 96-GeparDouze Pathology and Correlative Science Instructions (North American sites) or country-specific study manual***.

c Includes assessment of the primary breast tumor and palpable regional lymph nodes.

d Baseline H&P (by physician or other healthcare professional) appropriate for assessment.

e Concomitant medications include any prescription medications or over-the-counter medications.

f Patients with abnormal TSH or AM cortisol baseline levels should be further evaluated and managed per institutional standards.

g Hepatitis B virus DNA must be collected prior to randomization in patients who have negative serology for Hepatitis B surface antigen and positive serology for antibody to hepatitis B core antigen.

h For women of childbearing potential.

i ***Echocardiogram is the preferred method for assessment of LVEF.*** However, LVEF assessment by MUGA scan is permitted. All subsequent LVEF assessments should be performed by the same method (echocardiogram or MUGA scan) that was performed at baseline.

j Chest imaging is required for N2 or N3 nodal disease or T3 primary tumors. Acceptable methods for chest imaging include CXR PA and Lateral, CT scan of chest, or PET CT.

k Liver imaging is required for N2 or N3 nodal disease or T3 primary tumors ***or*** if alkaline phosphatase, AST, or ALT is > ULN. Acceptable methods of liver imaging include CT, MRI, abdominal ultrasound, PET-CT, or PET scans supported by additional studies when indicated (CT, x-ray, MRI) to rule out metastatic disease.

l Nuclear bone imaging (supported by additional studies when indicated [CT, x-ray, MRI]) is required for N2 or N3 nodal disease or T3 primary tumors ***or*** if alkaline phosphatase is > ULN or if the patient has unexplained bone pain. PET scan or PET-CT scan is permitted as a substitute for a bone scan.

m MRI is permitted before entry as a substitute for a mammogram (ultrasound is not) at baseline and before surgery; all other assessments must be performed by mammogram.

n If suspicious or abnormal nodes are detected on imaging, FNA or core biopsy is recommended prior to randomization.

o Marking of the primary tumor site before therapy begins is recommended (see Appendix C).

p See Section 6.0 and the ***NSABP B-59/GBG 96-GeparDouze Pathology and Correlative Science Instructions (North American sites) or country-specific study manual***.

Table 2. Tests, exams, and other requirements during therapy and through end of study for Arm 1 and Arm 2

**Required assessments** *(see footnotes **a** and **b**)*	**Within 3 days during weekly paclitaxel and every 3 week carboplatin (Weeks 1, 4, 7, 10, 13[c])**	**Within 3 days before each cycle of AC/EC[d]**	**Prior to surgery (2-4 weeks after last dose of study therapy)**	**3 to 6 weeks after surgery**	**During adjuvant atezolizumab/placebo**	**30 days (+/- 3 days) after the last dose of study therapy**	**Follow-up during Years 2 through 5 from randomization[b]**	**Follow-up during Years 6 through end of study from randomization**
History & physical exam[e]	X	X	X	X	X (every 6 weeks)	X	X (every 6 mos)	
Adverse event assessment[f]	X	X	X	X	X	X		
Assessment for initial EFS events, late immune-related events, survival, and post-recurrence immune therapies, if applicable							X (every 6 mos)	X (every 12 mos)
Weight	X	X						
Assessment of concomitant medications	X	X			X (every 6 weeks)	X		
Tumor assessment[g]	X (recommended)	X (recommended before each cycle; **required prior to first dose of AC/EC**)	X[h,i]					
CBC/differential/platelets	X (may be obtained weekly per institutional standards)	X	X		X (every 6 weeks)	X	X (every 6 mos in Year 2)	

Table continued on next page.

Table 2. Tests, exams, and other requirements during therapy and through end of study for Arm 1 and Arm 2 *(continued)*

**Required assessments** *(see footnotes **a** and **b**)*	**Within 3 days during weekly paclitaxel and every 3 week carboplatin (Weeks 1, 4, 7, 10, 13[c])**	**Within 3 days before each cycle of AC/EC[d]**	**Prior to surgery (2-4 weeks after last dose of study therapy)**	**3 to 6 weeks after surgery**	**During adjuvant atezolizumab/placebo**	**30 days (+/- 3 days) after the last dose of study therapy**	**Follow-up during Years 2 through 5 from randomization[b]**	**Follow-up during Years 6 through end of study from randomization**
Total bilirubin/AST/ALT/ alkaline phosphatase, total protein, albumin	X	X	X		X (every 6 weeks)	X	X (every 6 mos in Year 2)	
Serum chemistry: creatinine, sodium, potassium, chloride, carbon dioxide, calcium, glucose	X[j]	X	X		X (every 6 weeks)	X	X (every 6 mos in Year 2)	
TSH, AM (morning) Cortisol[k]		X (prior to first dose of AC/EC)	X		X (every 12 weeks)		X (every 6 mos in Year 2)	
Pregnancy test	As clinically indicated, per institutional standards.							
Research biopsy of primary tumor (first 500 patients who agree to optional research biopsy)	X (required 1-4 days prior to week 4 [Cycle 2])[l]							
Echocardiogram (or MUGA scan)[m]				X (see Section 8.23)			X (18 mos from randomization)	
Bilateral breast imaging[n]			X (only ipsilateral required)				X (minimum of every 12 mos from previous imaging)	

Table continued on next page.

Table 2. Tests, exams, and other requirements during therapy and through end of study for Arm 1 and Arm 2 *(continued)*

**Required assessments** *(see footnotes **a** and **b**)*	**Within 3 days during weekly paclitaxel and every 3 week carboplatin (Weeks 1, 4, 7, 10, 13[c])**	**Within 3 days before each cycle of AC/EC[d]**	**Prior to surgery (2-4 weeks after last dose of study therapy)**	**3 to 6 weeks after surgery**	**During adjuvant atezolizumab/placebo**	**30 days (+/- 3 days) after the last dose of study therapy**	**Follow-up during Years 2 through 5 from randomization[b]**	**Follow-up during Years 6 through end of study from randomization**
Submission of tumor sample(s)[o]				X				
Collection of plasma sample for ctDNA<br>*(A sample must also be collected at time of tumor recurrence.)*			X	X			X<br>(12 and 24 months only [+/- 2 months])	
Refer to Table 3 for additional tests and exams for patients assigned to the Cardiac Safety Lead-in study.								

a H&P, bloodwork, x-rays, scans, and other testing may be performed more frequently at the discretion of the investigator.

b Patients who discontinue all protocol therapy for reasons other than disease recurrence must follow the exams, assessments, and bloodwork outlined in the **Follow-up during Years 2 through 5 from randomization** column on Table 2. Tissue sample submission should continue as outlined on Table 2. ***NOTE****: While tests, exams, and assessments,* ***are not required*** *following a documented invasive breast cancer recurrence, invasive contralateral breast cancer, or second non-breast primary cancer excluding squamous or basal cell skin cancers or new in situ carcinomas of any site, follow-up for subsequent cancer events and immune-mediated events, post-recurrence immune therapies, and for survival continues to be required every 6 months through Year 5 and annually for Years 6 through end of study. (See Section 10.0 for adverse event reporting requirements.)*

c H&P, bloodwork, and assessments to be performed at Week 13 if required due to delays in treatment.

d Blood work and assessments follow the chemotherapy administration schedule during AC/EC. Blood work should be done if clinically indicated prior to the atezolizumab/placebo dose administered without AC/EC during the DD AC/EC schedule.

e Updated H&P with exams (by physician or other healthcare professional) appropriate for therapy-related assessments and follow-up evaluations.

f AE assessment is ongoing. Final AE assessment for ***preoperative therapy*** should be performed 2–4 weeks after completion of preoperative chemotherapy (before surgery). Following the AE assessment 30 days after the last dose of study therapy, routine reporting is no longer required (see Sections 10.7 and 10.8). Patients who stop study therapy due to breast cancer progression or recurrence, second breast primary cancer, or begin systemic treatment for another cancer must complete the 30-day post-study therapy assessments. These patients should continue to be followed for other protocol events as noted in footnote b.

Table continued on next page.

Table 2. Tests, exams, and other requirements during therapy and through end of study for Arm 1 and Arm 2 *(continued)*

g Includes assessment of the primary breast tumor and palpable regional lymph nodes.
h Tumor assessment by physical exam to ensure there has been no disease progression.
i Surgery should be performed as soon as possible after recovery from neoadjuvant therapy, final tumor assessment, and breast imaging.
j A current serum creatinine value is required before each carboplatin dose for calculation of the creatinine clearance, and subsequently the carboplatin dosing (see Section 7.2.1).
k Patients with abnormal TSH or AM cortisol baseline levels should be further evaluated and managed per institutional standards.
l A research biopsy is only required for the first 500 patients who agree to an optional research biopsy (see Section 6.3.2).
m ***Echocardiogram is the preferred method for assessment of LVEF.*** However, LVEF assessment by MUGA scan is permitted. All LVEF assessments should be performed by the same method (echocardiogram or MUGA scan) that was performed at baseline and at the same facility.
n MRI is permitted as a substitute for mammogram (ultrasound is not) at baseline and before surgery; all other assessments must be performed by mammogram. Postoperative assessment may be unilateral for patients who have had a mastectomy with or without reconstruction.
o If gross residual disease remains, submission of tumor samples is required within 4-6 weeks following definitive breast surgery (see Table 4 in Section 6.1) even if study therapy has been discontinued and alternative therapy has been given.

Table 3. Additional studies required in cardiac safety lead-in study (first 60 patients to begin AC/EC)

**Required assessments**	**Within 3 days prior to Cycle 1 of AC/EC**	**After administration of Cycle 1 of AC/EC prior to administration of atezolizumab/placebo**	**Prior to Cycle 3 of AC/EC**	**After administration of Cycle 3 of AC/EC prior to administration of atezolizumab/placebo**
ECG*	X	X		X
Serum troponin-T*	X	X		X
Echocardiogram (or MUGA scan)**			X	

***** If ECG is unchanged from baseline, therapy may be administered without waiting for results of the troponin-T (refer to Section 8.6.2 for evaluation and management of asymptomatic toxicity).
****** ***Echocardiogram is the preferred method for assessment of LVEF.*** However, LVEF assessment by MUGA scan is permitted. All LVEF assessments should be performed by the same method (echocardiogram or MUGA scan) that was performed at baseline and at the same facility.

6.0 PATHOLOGY AND CORRELATIVE SCIENCE STUDIES

6.1 Overview of requirements

Collection and submission of all patient tumor and blood samples listed below are requirements for all patients participating in the NSABP B-59/GBG 96-GeparDouze study. ***By signing the NSABP B-59/GBG 96-GeparDouze consent form, the patient has agreed to all the required sample collections and submissions*** as outlined in Table 4. The research biopsy 1-4 days prior to the second dose of atezolizumab/placebo is optional and will be performed on the first 500 patients who agree to an optional research biopsy.

Table 4. Summary of tumor and blood sample submission requirements

**Study requirements for ALL patients**	**Before randomization**	**1-4 days prior to 2nd dose of atezolizumab/ placebo**	**Prior to surgery (2-4 weeks after last dose of study therapy)**	**At the time of definitive surgery**	**3 to 6 weeks after surgery**	**12 and 24 months after randomization**
Material from either a diagnostic core biopsy or research biopsy must be submitted for central HER2, ER, and PgR testing and PD-L1 status	X					
Two cores from research biopsy for PD-L1 and TIL testing and future research. ***(Note: If 2 extra core biopsies were collected at the time of the diagnostic biopsy are available, they may be submitted as an alternative to a research biopsy at baseline.)***[a]	X	X (first 500 patients who agree to optional biopsy)				
Formalin-fixed paraffin-embedded (FFPE) cores from residual tumor at time of surgery for TIL testing and future research				X[b,c]		
Plasma collection for ctDNA (2 tubes) *(A blood sample must also be collected at the time of tumor recurrence.)*	X (Before or after randomization but before therapy begins)		X		X	X

Table continued on next page.

Table 4. Summary of tumor and blood sample submission requirements *(continued)*

**Study requirements for ALL patients**	**Before randomization**	**1-4 days prior to 2nd dose of atezolizumab/ placebo**	**Prior to surgery (2-4 weeks after last dose of study therapy)**	**At the time of definitive surgery**	**3 to 6 weeks after surgery**	**12 and 24 months after randomization**
**a** For core biopsy specimen procurement, follow instructions in the NSABP B-59/GBG 96-GeparDouze Pathology and Correlative Science Instructions (North American sites) or country-specific study manual.						
**b** If gross residual disease is < 1.0 cm, tissue should be submitted, if possible. If gross residual disease is ≥ 1.0 cm, a representative block of residual tumor and the node with the largest focus of metastasis must be submitted.						
**c** If the pathology department refuses to provide a block for research purposes, refer to the NSABP B-59/GBG 96-GeparDouze Pathology and Correlative Science Instructions for alternative sample submission instructions.						
Refer to the NSABP B-59/GBG 96-GeparDouze Pathology and Correlative Science Instructions (North American sites) or country-specific study manual for tumor and blood sample collection and submission instructions.						

6.2 Use of specimens

The tumor, plasma, and serum samples collected in this study will be used for research studies as described in the sections below and also for other studies which are not currently described in the protocol document. Results of the correlative science studies, including any sequencing data, will not be reported directly to the patient or the physician and will not have any bearing on patient treatment.

6.3 Tumor and plasma specimen submissions

6.3.1 *Core biopsy of breast primary for central testing*

Material from either the diagnostic core biopsy or the research biopsy can be used for central testing for ER, PgR, HER2, and PD-L1.

6.3.2 *Research core biopsies*

Research core biopsies of breast primary at baseline (all patients) and 1-4 days prior to the second dose of atezolizumab/placebo (first 500 patients who agree to an optional research biopsy) must be collected and submitted. Material will not be returned. If two extra core biopsies collected at the time of the diagnostic biopsy are submitted as the alternative to a research biopsy at baseline, the material will not be depleted and could be returned if requested.

6.3.3 *Residual tumor*

One to three representative blocks of residual primary tumor containing the maximum amount of tumor <u>**and**</u> node with the largest focus of metastasis is required from the definitive breast surgery if gross residual disease is ≥ 1.0 cm. ***If gross residual disease is < 1.0 cm,*** tissue should be submitted, if possible.

6.4 Central testing for PD-L1 status

PD-L1 testing will be performed with the Ventana PD-L1 (SP142) assay kit which uses the OptiView DAB IHC detection kit and OptiView Amplification kit on a Ventana BenchMark Ultra Instrument along with the specified antibody. PD-L1 expression within biopsied tumor will be assessed. PD-L1 positive status is defined as ≥ 1% staining of tumor-infiltrating immune cells as a percentage of tumor area using the SP142 Ventana assay. Results will not be provided to the investigator or the patient.

6.5 Rationale for correlative science studies

Denkert and colleagues demonstrated that the percentage of baseline TILs in the tumor stroma predicted for increasing rates of pCR with addition of carboplatin on GeparSixto (Denkert 2015). Baseline TILs have also been shown to be predictive of long-term outcomes in the adjuvant setting, especially in TNBC patients (Loi 2013, Adams 2015). PD-L1 staining intensity in baseline tumors has also been shown to predict for a greater probability for response of a number of solid tumors to inhibition of the PD-1/PD-L1 interaction. Therefore, research core biopsies of the primary tumor will be collected prior to starting protocol therapy to determine baseline stromal TIL infiltration and PD-L1 staining intensity to determine if either of these parameters will predict for increased activity of atezolizumab.

Neoadjuvant chemotherapy administration has also been shown to alter TIL infiltration (Demaria 2001), and the presence of TILs in residual cancer following neoadjuvant chemotherapy has associated with improved long-term outcomes (Dieci 2014). Expression of PD-L1 is also a dynamic process and relatively little is known about the impact of chemotherapy alone and chemotherapy in combination with atezolizumab on PD-L1 and other immune parameters (Vilain 2017). Early changes in TIL infiltration and the expression of PD-L1 and other immune biomarkers could potentially predict for increased probability for pCR and improved long-term outcomes. Alternatively, a failure of an apparent change from baseline could predict for lack of efficacy of atezolizumab. A second biopsy for research will be performed 1-4 days prior to administration of the second dose of atezolizumab/placebo and assessed for TIL infiltration and PD-L1 expression amongst other immune markers.

Circulating tumor DNA (ctDNA) may provide a means to predict relapse after primary breast cancer systemic therapy and characterize the genetic composition of the resistant clones (Bidard 2013; Dawson 2013; De Mattos-Arruda 2013). Specimens collected for ctDNA will be analyzed as an early predictor of relapse.

The tumor and plasma samples collected in this study will also be used for NSABP B-59/GBG 96-GeparDouze studies for analyses to be conducted in the future related to the purposes of the NSABP B-59/GBG 96-GeparDouze study but not currently described in the protocol document. The specimens procured will be used for future studies involving genes and proteins conferring susceptibility to cancer or other diseases. When hereditary genetic studies (germline research, i.e., involving traits that are passed on or inherited) are conducted, an anonymization process will be used. Results of the correlative science studies will not be reported to the patient or the patient's physician and will not have any bearing on the patient's treatment.

The results of correlative studies will be communicated through publication, in peer reviewed scientific literature and/or through presentations at scientific meetings. Research data (as deemed appropriate by NSABP and GBG), including genome sequencing data, may be submitted to public research databases for data sharing with controlled access to scientific researchers outside of NSABP and GBG. Submitted data will be anonymized or de-identified as deemed appropriate by NSABP and GBG.

7.0 STUDY TREATMENT

This is a double-blind trial; neither the patient nor the investigator/healthcare providers will know the treatment assigned.

- Study therapy should begin within 14 days following randomization.
- ***Results of laboratory safety assessments are to be reviewed prior to administration of components of study therapy pertinent to that parameter.***
- ***Central venous access is strongly recommended.***
- Duration of infusion times and order of administration of paclitaxel, carboplatin, and AC/EC may be altered per institutional standards; however, the atezolizumab/placebo must be administered after the chemotherapy.

7.1 Study regimen

Table 5. Arm 1 and Arm 2 treatment regimen

Drug	Dose and Administration	Dosing Interval	Planned Duration
Paclitaxel (WP)	[redacted]	[redacted]	[redacted]
Carboplatin	[redacted]	[redacted]	[redacted]
Atezolizumab/Placebo	[redacted]<br>[redacted]	[redacted]	[redacted]
Initiate ≥ 3 weeks after carboplatin or ≥ 1 week after paclitaxel, whichever is later, and toxicities of paclitaxel and carboplatin have resolved as outlined in Sections 8.5 and 8.8.			
Doxorubicin (A)	[redacted]	[redacted]	[redacted]
Cyclophosphamide (C)	[redacted]		
Atezolizumab/Placebo	[redacted]	[redacted]	[redacted]<br>[redacted]
Liposomal or pegylated G-CSF	[redacted]	[redacted]	[redacted]

Table continued on next page.

Table 5. Arm 1 and Arm 2 treatment regimen *(continued)*

**OR**			
**Drug**	**Dose and Administration**	**Dosing Interval**	**Planned Duration**
Epirubicin (E)	[redacted]	[redacted]	[redacted]
Cyclophosphamide (C)	[redacted]		
Atezolizumab/Placebo	[redacted]	[redacted]	[redacted]<br>[redacted]
Liposomal or pegylated G-CSF	[redacted]	[redacted]	[redacted]
**Surgery** (See Appendix C for surgery-related instructions.)			
Atezolizumab/Placebo[f,g,h]	[redacted]	[redacted]	[redacted] the first dose

- **a** Patients should receive premedications as follows before each paclitaxel dose:
  - Dexamethasone 4-12 mg IV, completed 30 minutes before each paclitaxel administration.
  - H-1 blocker IV or PO (diphenhydramine hydrochloride, dimetinden, or clemastine) and an H-2 blocker IV or PO (cimetidine, ranitidine, or famotidine) before each paclitaxel administration.
  - Per institutional standards, dexamethasone and diphenhydramine hydrochloride doses may be tapered during the paclitaxel cycles.
  - Doses and routes of premedication administration may be modified per institutional standards.
- **b** Carboplatin and paclitaxel therapy must not continue beyond 16 weeks after initiation of therapy.
- **c** If an infusion reaction occurs, the subsequent infusion must be given over 60 minutes (+/- 15 minutes). Premedication with antihistamines may be administered per institutional standards. See Section 8.14. For management of cytokine-release syndrome, see Section 8.14.
- **d** Patients should receive premedications before each AC/EC dose per institutional standards.
- **e** *Primary* prophylaxis *with G-CSF is required during DD AC/EC cycles* (see Section 7.3.1 for instructions); and per institutional standards for the every-3-week AC/EC schedule.
- **f** Post-operative administration of atezolizumab/placebo should resume 3-6 weeks after surgery when adequate healing has occurred. Administration of atezolizumab/placebo during radiation therapy is recommended, if radiation is administered. If decision is made to delay resumption of atezolizumab/placebo until after completion of radiation therapy, the atezolizumab/placebo must be resumed within 12 weeks of surgery (see Section 8.7).
- **g** If decision is made per local guidelines to administer adjuvant capecitabine to patients with a non-pCR status following neoadjuvant therapy and surgery, it should be given concurrently with atezolizumab/placebo with recording of doses administered in eCRF. If radiation therapy is also administered, capecitabine should be initiated following completion of radiation therapy.
- **h** Patients must initiate adjuvant atezolizumab/placebo within 12 weeks of surgery. If patient does not initiate adjuvant atezolizumab/placebo within the 12 week period, the study product should be permanently discontinued.

7.2 Dose determinations

7.2.1 *Carboplatin dose calculations*

- Carboplatin dose is calculated as total mg (not mg/m²) using a modified Calvert Formula (creatinine clearance is substituted for GFR) as follows:

  [redacted]

- The maximum dose that can be administered is based on a creatinine clearance that is capped at 125 mL/min for patients with normal renal function.

  [redacted]

7.2.2 *Recalculation of BSA and/or drug doses*

[redacted]

7.2.3 *Rounding doses*

Rounding of drug doses is optional. If done, perform according to institutional standards. (This also applies to dose modifications.)

7.3 Supportive therapy

7.3.1 *G-CSF*

- G-CSF may be used as secondary prophylaxis and to treat neutropenia or neutropenia associated infections during administration of carboplatin/paclitaxel per institutional standards.
- ***Primary prophylaxis with G-CSF is required during dose-dense AC/EC.*** It should be administered according to the package insert for the agent used.
- Loratadine (10 mg) or similar agents are recommended when liposomal or pegylated G-CSF is used.
- Do not administer G-CSF within 24 hours of chemotherapy.
- Liposomal or pegylated G-CSF is recommended for primary prophylaxis; however, if required by institutional standards, GM-CSF or G-CSF may be administered as an alternative per institutional standards.

7.3.2 *Magnesium supplementation*

Magnesium levels should be monitored in patients receiving carboplatin therapy and magnesium supplementation initiated per institutional standards.

7.3.3 *Bone-directed therapy*

Bisphosphonate and denosumab are allowed.

7.4 Concomitant therapy precautions

Concomitant therapy includes any medication (e.g., prescription drugs, over-the-counter drugs, vaccines, herbal or homeopathic remedies, nutritional supplements) used by a patient in addition to protocol-mandated study treatment from 14 days prior to initiation of randomization to the treatment discontinuation visit. Concomitant use of herbal therapies is not recommended because their pharmacokinetics, safety profiles, and potential drug-drug interactions are generally unknown.

Patients who experience infusion-associated symptoms may be treated symptomatically with acetaminophen, ibuprofen, H1-receptor antagonists (diphenhydramine, dimentinden, or clemastine) and/or H2-receptor antagonists (e.g., famotidine, cimetidine), or equivalent medications per local standard practice. Serious infusion-associated events manifested by dyspnea, hypotension, wheezing, bronchospasm, tachycardia, reduced oxygen saturation, or respiratory distress should be managed with supportive therapies as clinically indicated (e.g., supplemental oxygen and β2-adrenergic agonists).

Systemic corticosteroids are recommended, at the discretion of the investigator, for the treatment of specific immune-mediated adverse events when associated with atezolizumab or placebo therapy (refer to the Atezolizumab Investigator's Brochure for details).

7.5 Surgery

Investigators will follow country-specific surgery guidelines in Appendix C or according to the country-specific study manual.

7.6 Postoperative radiation therapy

Patients should have adjuvant radiotherapy according to local policy and/or international guidelines. When indicated, it is recommended that adjuvant atezolizumab/placebo be administered concurrently with radiation therapy, but the adjuvant atezolizumab/placebo may be delayed until after completion of radiation therapy per investigator discretion.

7.7 Prohibited therapies

Use of the following concomitant therapies is prohibited as described below:

- Neoadjuvant chemotherapy other than the neoadjuvant chemotherapy specified in this protocol.
- Immunotherapy other than the immunotherapy specified in this protocol.
- Radiotherapy other than the radiotherapy specified in this protocol, whether health authority−approved or experimental, are prohibited during study treatment and post-study therapy follow-up until disease recurrence.
- **Adjuvant therapy other than atezolizumab/placebo with the following exceptions:**
    - Endocrine therapies administered in patients with synchronous bilateral or multicentric HER2-negative cancers, ER-positive or PgR-positive nondominant tumors, or patients with residual breast cancer following neoadjuvant therapy which is ER-positive or PgR-positive on retesting.
    - Adjuvant capecitabine in patients with non-pCR status following neoadjuvant therapy per investigator discretion and local guidelines.
    - Adjuvant olaparib may be used as an alternative to capecitabine in patients with germline *BRCA1* or *BRCA2* mutations and non-pCR status following neoadjuvant therapy per investigator discretion and country-specific guidelines. If olaparib is administered, adjuvant atezolizumab/placebo should be discontinued.
- Additional investigational therapy is prohibited during the study.
- Live, attenuated vaccines (e.g., FluMist®) are prohibited within 28 days prior to randomization, during study treatment, and for 5 months after the last dose of atezolizumab/placebo.
- Systemic immunostimulatory agents (including, but not limited to, interferons and IL-2) are prohibited within 28 days or 5 half-lives of the drug, whichever is longer, prior to randomization and during study treatment because these agents could potentially increase the risk for autoimmune conditions when given in combination with atezolizumab.
- Systemic immunosuppressive medications (including, but not limited to, azathioprine, methotrexate, and thalidomide) are prohibited during study treatment because these agents could potentially alter the efficacy and safety of atezolizumab.

7.8 Participation in other clinical trials

Patients are not permitted to participate in other therapeutic trials. Contact the Clinical Coordinating Department for NSABP North American patients, the GBG Data Manager for European patients at GBG sites, or country-specific study monitors to discuss potential participation in supportive therapy trials, Phase III locoregional or radiation therapy trials, by a patient enrolled in NSABP B-59/GBG 96-GeparDouze (see Information Resources).

8.0 TREATMENT MANAGEMENT

8.1 General instructions

- The CTCAE v4.0 must be used to grade the severity of AEs. Refer to http://ctep.cancer.gov/protocolDevelopment/electronic_applications/ctc.htm.
- Dose reductions for paclitaxel, carboplatin, and AC/EC must be based on the AE requiring the greatest modification.
- Paclitaxel, carboplatin, and AC/EC doses that have been reduced may not be escalated.
- Atezolizumab/placebo will be administered every 3 weeks during paclitaxel and carboplatin and every 3 weeks during co-administration with AC/EC, regardless of AC/EC schedule (see Section 8.8).
- There are no dose reductions for atezolizumab/placebo.
- If necessary, the timing of a treatment may be adjusted to 2 days earlier or 2 days later than the scheduled date of treatment, though paclitaxel doses should not be administered within 5 days of each other.
- Atezolizumab/placebo must not be administered beyond 52 weeks after the first dose (Day 1) of the first treatment cycle regardless of any missed doses or treatment delays.

8.2 Management of anemia

Chemotherapy should not proceed with ≥ Grade 3 anemia. Transfusion is acceptable for improving the hemoglobin value to allow therapy to continue without delay. The patient should be assessed to rule out other causes of anemia. *Use of erythropoiesis-stimulating agents is prohibited.*

8.3 Management of protocol therapy with documented COVID-19 infection

Atezolizumab/placebo should be held with documentation of COVID-19, and patients with cough, tachypnea, dyspnea or hypoxemia, particularly if accompanied by fever, should undergo chest imaging to assess for pneumonia or pneumonitis. Refer patient to infectious disease specialists for further evaluation and therapy. If required for medical management, patient should be unblinded.

Patients with Grade 1-4 pneumonitis should be unblinded, and ID consultants should be notified if patients had been receiving atezolizumab.

Patients with documented COVID-19 infection should have atezolizumab/placebo held until all disease-related symptoms have been absent for at least 7 consecutive days and repeat testing demonstrates clearance of the virus. Resumption of atezolizumab/placebo for these patients will be at investigator discretion, unless medication has been held for 12 or more weeks which requires permanent discontinuation.

COVID-19 should be reported as an adverse event or severe adverse event if criteria are met, as Infections and infestations – Other, specify as COVID-19.

Chemotherapy should be held during any Grade 2, 3 or 4 events related to COVID-19 infections. Resumption or continuation of chemotherapy with only Grade 1 manifestations of COVID-19 will be at investigator discretion per local guidelines.

8.4 Treatment management for carboplatin and paclitaxel

- Paclitaxel will be administered weekly for 12 doses with delays as required by dose modifications in Section 8.5.
- Carboplatin will be administered every 3 weeks for 4 doses with delays as required by dose modifications in Section 8.5 and will begin with the first dose of weekly paclitaxel.
- Dose delays of either carboplatin or paclitaxel will be evaluated weekly to resume therapy.
- Carboplatin and paclitaxel must not be continued beyond 16 weeks after initiation of paclitaxel and carboplatin. Any of the 12 paclitaxel doses or 4 carboplatin doses remaining after 16 weeks following the first paclitaxel and carboplatin doses should not be administered. The patient should proceed to AC/EC when criteria for administration are met.
- Do NOT co-administer carboplatin with the 12th dose of paclitaxel if carboplatin delays in absence of paclitaxel delays have occurred previously.
- Unless otherwise specified, carboplatin or paclitaxel that is held due to toxicity will not be resumed until the toxicity has resolved to ≤ Grade 1. Carboplatin may be held independently of paclitaxel.
- If carboplatin is discontinued due to toxicity, the remaining paclitaxel doses should be administered.
- If paclitaxel is discontinued due to toxicity, the remaining carboplatin doses must also be discontinued.
- If carboplatin and paclitaxel are both discontinued prior to completion of planned doses, proceed with initiation of AC/EC with atezolizumab/placebo when criteria for administration are met.
- If a hypersensitivity and/or infusion reaction occurs during paclitaxel administration, weekly nab-paclitaxel may be substituted per investigator and local guidelines for subsequent paclitaxel doses with starting dose and dose modifications if necessary per investigator discretion. Patients continue on study and continue to follow protocol requirements outlined on Table 2.
- All dose modifications for carboplatin and paclitaxel are based on the dose level changes outlined in Table 6.

Table 6. Dose levels for paclitaxel and carboplatin (Arm 1 and Arm 2)

	**Dose Level 0**<br>***Starting Dose***	**Dose Level -1**	**Dose Level -2**	**Dose Level -3**
**Carboplatin (AUC)**<br>*(see Section 7.2.1)*	[redacted]	[redacted]	[redacted]	[redacted]
**Paclitaxel (mg/m²)**	[redacted]	[redacted]	[redacted]	[redacted]

8.5 Dose modifications for carboplatin and paclitaxel

[redacted]

[redacted]

[redacted]

[redacted]

[redacted]

[redacted]

[redacted]

[redacted]

[redacted]

[redacted]

[redacted]

[redacted]

[redacted]

[redacted]

[redacted]

[redacted]

[redacted]

[redacted]

[redacted]

[redacted]

[redacted]

[redacted]

[redacted]

[redacted]

[redacted]

[redacted]

[redacted]

[redacted]

[redacted]

[redacted]

[redacted]

[redacted]

[redacted]

[redacted]

[redacted]

[redacted]

[redacted] [redacted]

[redacted]

[redacted]

[redacted]

[redacted]

[redacted]

[redacted]

[redacted]

[redacted]

[redacted]

[redacted]

[redacted]

[redacted]

[redacted]

[redacted]

[redacted]

[redacted]

[redacted]

[redacted]

[redacted]

	[redacted]	[redacted]	[redacted]	[redacted]
[redacted]	[redacted]	[redacted]	[redacted]	[redacted]
[redacted]	[redacted]	[redacted]	[redacted]	[redacted]
[redacted]				
[redacted]	[redacted]	[redacted]	[redacted]	[redacted]
[redacted]	[redacted]	[redacted]	[redacted]	[redacted]

[redacted]

[redacted]

[redacted]

[redacted]

[redacted]

[redacted]

[redacted]

[redacted]

[redacted]

[redacted]

[redacted]

[redacted]

[redacted]

[redacted]

[redacted]

[redacted]

[redacted]

[redacted]

[redacted]

[redacted]

8.7 Management of atezolizumab/placebo specific adverse events and potential unblinding

Management of certain AEs of concern, including immune-mediated pneumonitis, hepatitis, colitis, endocrinopathies, pancreatitis, neuropathies, meningoencephalitis, and potential ocular toxicities are presented in the Atezolizumab Investigator's Brochure.

Atezolizumab has been associated with risks such as the following: infusion-related reactions (IRRs) and immune-mediated hepatitis, pneumonitis, colitis, pancreatitis, diabetes mellitus, hypothyroidism, hyperthyroidism, adrenal insufficiency, hypophysitis, Guillain-Barré syndrome, myasthenic syndrome or myasthenia gravis, meningoencephalitis, myocarditis, nephritis, myositis, and severe cutaneous adverse reactions. Immune-mediated reactions may involve any organ system and may lead to hemophagocytic lymphohistiocytosis and macrophage activation syndrome.

Toxicities associated or possibly associated with atezolizumab/placebo treatment should be managed according to standard medical practice. Investigators are encouraged to review and refer to Clinical Practice Guidelines for management of immune-mediated adverse events in patients treated with immune checkpoint inhibitors published by the American Society for Clinical Oncology (Brahmer 2018, Schneider 2021) and the European Society for Medical Oncology (Haanen 2017), along with the dose modification guidelines provided in this protocol for evaluation and management of potential immune adverse events. Additional tests, such as autoimmune serology or biopsies, should be used to evaluate for a possible immunogenic etiology.

Although most immune-mediated adverse events observed with immunomodulatory agents have been mild and self-limiting, such events should be recognized early and treated promptly to avoid potential major complications. Discontinuation of atezolizumab/placebo may not have an immediate therapeutic effect, and more severe immune-mediated toxicities may require acute management with topical corticosteroids, systemic corticosteroids, or other immunosuppressive agents.

The following are general recommendations for management of any adverse events that may occur and are not specifically listed in the following subsections.

- Patients and family caregivers should receive timely and up-to-date information about immunotherapies, their mechanism of action, and the clinical profile of possible immune-related adverse events prior to initiating therapy and throughout treatment and survival follow-up. There should be a high level of suspicion that new symptoms are treatment related.
- In general, atezolizumab therapy should be continued with close monitoring for Grade 1 toxicities, except for some neurologic toxicities.
- Consider holding atezolizumab for most Grade 2 toxicities and resume when symptoms and/or laboratory values resolve to Grade 1 or better. Corticosteroids (initial dose of 0.5-1 mg/kg/day of prednisone or equivalent) may be administered.
- For Grade 2 recurrent or persistent (lasting for more than 5 days) events, treat as a Grade 3 event.
- Hold atezolizumab for Grade 3 toxicities and initiate treatment with high-dose corticosteroids (1-2 mg/kg/day prednisone or equivalent). Corticosteroids should be tapered over 1 month to 10 mg/day oral prednisone or equivalent, before atezolizumab can be resumed. If symptoms do not improve within 48 to 72 hours of high-dose corticosteroid use, other immunosuppressants may be offered for some toxicities.
- Grade 4 toxicities warrant permanent discontinuation of atezolizumab treatment.

In general, for Grade 3 or 4 toxicities which have the potential for being immune-mediated, unblinding should be requested to determine if a patient had been receiving atezolizumab or placebo. **Management of patients with Grade 3 or 4 toxicities will require aggressive immunosuppressive therapies if a patient has been receiving atezolizumab.** If a patient had been receiving placebo, alternative therapies and additional diagnostic testing may be indicated. Patients with Grade 2 toxicities may have atezolizumab/placebo held and observed without initiation of immunosuppressive therapy, with consideration of rechallenge without unblinding if medically appropriate. However, if a decision is made to initiate immunosuppressive therapy, unblinding should be considered to determine if the patient was receiving atezolizumab or placebo to determine if the immunosuppressive therapy is appropriate. **If immunosuppressive**

therapy is initiated for management of immune-mediated adverse events, the specific agents utilized, along with dose and duration of therapy must be recorded. Patients should be followed for resolution of symptoms and discontinuation of immunosuppressive therapy without evidence of relapse.

When the unblinding is requested, the date and reason for unblinding must be fully documented in source documents and recorded in the case report forms (see Section 9.9). If unblinding occurs, the site staff should make every effort to ensure that the treatment arm in which the unblinded patient was assigned is not communicated to any sponsor personnel or designee involved in the study. ***If unblinding occurs, the atezolizumab/placebo must be permanently discontinued.***

8.8 Treatment management for atezolizumab/placebo

- Atezolizumab/placebo schedule should proceed independently of paclitaxel and carboplatin administration.
- The first dose of AC/EC should be co-administered with atezolizumab/placebo. The atezolizumab/placebo may be held for 2 weeks if needed to allow for co-administration.
- Atezolizumab/placebo must be discontinued if non-protocol chemotherapy is initiated.
- If atezolizumab/placebo is discontinued prior to surgery in the ***absence of progression***, the remaining chemotherapy doses should be administered if the patient is benefitting from the therapy.
- If atezolizumab/placebo must be held for > 12 weeks for toxicities related to atezolizumab/placebo, atezolizumab/placebo must be discontinued.
- If adjuvant atezolizumab/placebo is held due to administration of radiation therapy or adjuvant capecitabine, atezolizumab/placebo must be resumed within 12 weeks after surgery. If more than 12 weeks have elapsed since surgery, atezolizumab/placebo must be permanently discontinued.

8.9 Pulmonary events

Pulmonary events may present as new or worsening cough, chest pain, fever, dyspnea, fatigue, hypoxia, pneumonitis, and pulmonary infiltrates have been associated with the administration of atezolizumab. Patients should be assessed for pulmonary signs and symptoms throughout the study.

During the COVID-19 pandemic, patients presenting with cough, tachypnea, dyspnea or hypoxemia, particularly if accompanied by fever, should be tested for COVID-19 and undergo chest imaging to assess for pneumonia or pneumonitis.

Refer patient to infectious disease specialists for further evaluation and therapy. If required for medical management, patient should be unblinded.

All pulmonary events should be thoroughly evaluated for other commonly reported etiologies such as pneumonia or other infection, lymphangitic carcinomatosis, pulmonary embolism, heart failure, chronic obstructive pulmonary disease, or pulmonary hypertension. Patients with documented pneumonitis should be monitored with serial chest CTs until resolution or stable. Management guidelines for pulmonary events are provided in Table 8.

Table 8. Management guidelines for pulmonary events, including pneumonitis, related to atezolizumab/placebo

Event	Management
Pulmonary event, Grade 1	• Continue or withhold atezolizumab/placebo at investigator discretion and monitor closely.<br>• Consider diagnostic imaging. If pneumonitis documented, monitor on serial imaging until resolution or stable.<br>• Consider patient referral to pulmonary specialist.
Pulmonary event, Grade 2	• Hold atezolizumab/placebo.<br>• Refer patient to pulmonary and infectious disease specialists and consider bronchoscopy or bronchoscopic alveolar lavage with or without transbronchial biopsy.<br>• If evaluation fails to define non-immune etiology OR defines an immune etiology, discontinue atezolizumab/placebo and request unblinding if required for medical management.<br>- If receiving atezolizumab, initiate treatment with 1–2 mg/kg/day oral prednisone or equivalent.<br>- If no improvement after 48-72 hours of corticosteroids, treat as a Grade 3 or 4 event.<br>- When event resolves to Grade 1 or better, taper corticosteroids over ≥ 1 month.
Pulmonary event, Grade 3 or 4	• Refer patient to pulmonary and infectious disease specialists for bronchoscopy or bronchoscopic alveolar lavage with or without transbronchial biopsy.<br>• Oral or IV broad-spectrum antibiotics should be administered in parallel to the immunosuppressive treatment.<br>• Discontinue atezolizumab/placebo and request unblinding.<br>- If receiving atezolizumab, initiate treatment with 1–2 mg/kg/day IV methylprednisolone or equivalent.<br>- If event does not improve within 48 hours after initiating corticosteroids, consider adding an immunosuppressive agent.<br>- When event resolves to Grade 1 or better, taper corticosteroids over ≥ 1 month.

8.10 Hepatic events

Immune-mediated hepatitis has been associated with the administration of atezolizumab. Eligible patients must have adequate liver function, as manifested by measurements of total bilirubin and hepatic transaminases, and liver function will be monitored throughout study treatment. Management guidelines for hepatic events are provided in Table 9.

Patients with right upper-quadrant abdominal pain and/or unexplained nausea or vomiting should have liver function tests (LFTs) performed immediately and reviewed before administration of the next dose of study drug.

For patients with elevated LFTs, concurrent medication, viral hepatitis, and toxic or neoplastic etiologies should be considered and addressed, as appropriate.

Table 9. Management guidelines for hepatic events related to atezolizumab/placebo

Event	Management<br>*(Refer to Sections 8.5.3 and 8.6.7 for management of chemotherapy)*
Hepatic event, Grade 1	• Continue atezolizumab/placebo.<br>• If LFT values are above normal limits, monitor LFTs weekly until resolved to within normal limits or to baseline values.
Hepatic event, Grade 2	• Hold atezolizumab/placebo and recheck LFTs in 3-7 days.<br>- If LFTs are improving or have increased by < 25 IU and remain Grade 2, monitor LFTs weekly until LFTs return to ≤ Grade 1.<br>- If LFTs increase further by a minimum of 25 IU but remain Grade 2, discontinue atezolizumab/placebo and request unblinding if required for medical management.<br>• If patient was unblinded and was receiving atezolizumab, initiate treatment with 1–2 mg/kg/day oral prednisone or equivalent.<br>• When event resolves to Grade 1 or better, taper corticosteroids over ≥ 1 month.
Hepatic event, Grade 3 or 4	• Refer to hepatic specialist for evaluation and liver biopsy to establish etiology of hepatic injury.<br>• Discontinue atezolizumab/placebo and request unblinding.<br>- If receiving atezolizumab, initiate treatment with 1–2 mg/kg/day oral prednisone or equivalent.<br>- If event does not improve within 48 hours after initiating corticosteroids, consider adding an immunosuppressive agent.<br>- When event resolves to Grade 1 or better, taper corticosteroids over ≥ 1 month.

8.11 Gastrointestinal events

Immune-mediated colitis has been associated with the administration of atezolizumab. Management guidelines for diarrhea or colitis are provided in Table 10.

All events of diarrhea or colitis should be thoroughly evaluated for other more common etiologies. For events of significant duration or magnitude or associated with signs of systemic inflammation or acute-phase reactants (e.g., increased C-reactive protein, platelet count, or bandemia): Perform sigmoidoscopy (or colonoscopy, if appropriate) with colonic biopsy, with three to five specimens for standard paraffin block to check for inflammation and lymphocytic infiltrates to confirm immune colitis diagnosis.

Table 10. Management guidelines for gastrointestinal events (diarrhea or colitis) related to atezolizumab/placebo

Event	Management<br>*(Refer to Section 8.6.8 for management of chemotherapy)*
Diarrhea or colitis, Grade 1	• Continue atezolizumab/placebo.<br>• Initiate symptomatic treatment.<br>• Endoscopy is recommended if symptoms persist for > 7 days.<br>• Monitor closely.
Diarrhea or colitis, Grade 2	• Hold atezolizumab/placebo.<br>• Initiate symptomatic treatment.<br>• If strong clinical suspicion for immune-mediated colitis, start empiric IV steroids while waiting for definitive diagnosis.<br>• Refer to GI specialist for evaluation and sigmoidoscopy.<br>• If immune colitis documented or if events persist > 5 days, discontinue atezolizumab/placebo and request unblinding if required for medical management.<br>• If receiving atezolizumab, initiate treatment with 1–2 mg/kg/day oral prednisone or equivalent. If the event does not improve within 48 hours after initiating corticosteroids, consider adding an immunosuppressive agent.<br>• When event resolves to Grade 1 or better, taper corticosteroids over ≥ 1 month.
Diarrhea or colitis, Grade 3 or 4	• Refer patient to gastrointestinal specialist for evaluation and sigmoidoscopy with possible biopsy.<br>• Discontinue atezolizumab/placebo and request unblinding if required for medical management.<br>- Initiate treatment with 1–2 mg/kg/day intravenous methylprednisolone or equivalent and convert to 1–2 mg/kg/day oral prednisone or equivalent upon improvement.<br>- If event does not improve within 48 hours after initiating corticosteroids, consider adding an immunosuppressive agent.<br>- When event resolves to Grade 1 or better, taper corticosteroids over ≥ 1 month.

8.12 Endocrine events

Thyroid disorders, adrenal insufficiency, diabetes mellitus, and pituitary disorders have been associated with the administration of atezolizumab. Management guidelines for endocrine events are provided in Table 11.

Patients experiencing one or more unexplained AEs possibly indicative of endocrine dysfunction (including headache, fatigue, myalgias, impotence, mental status changes, and constipation) should be investigated for the presence of thyroid, pituitary, or adrenal endocrinopathies. The patient should be referred to an endocrinologist if an endocrinopathy is suspected. Thyroid-stimulating hormone (TSH) and T3 and T4 levels should be measured to determine whether thyroid abnormalities are present. Pituitary hormone levels and function tests (TSH, growth hormone, luteinizing hormone, follicle-stimulating hormone, testosterone, prolactin, adrenocorticotropic hormone [ACTH] levels, and ACTH stimulation test) and MRI of the brain (with detailed pituitary sections) may help to differentiate primary pituitary insufficiency from

primary adrenal insufficiency. Table 11 describes dose management guidelines for hyperthyroidism, hypothyroidism, symptomatic adrenal insufficiency, and hyperglycemia.

Table 11. Management guidelines for endocrine events related to atezolizumab/placebo

Event	Management
Asymptomatic hypothyroidism (Grade 1)	• Continue atezolizumab/placebo.<br>• Initiate treatment with thyroid replacement hormone.<br>• Monitor TSH closely.
Symptomatic hypothyroidism (Grade 2)	• Hold atezolizumab/placebo.<br>• Initiate treatment with thyroid replacement hormone.<br>• Monitor TSH closely.<br>• Consider patient referral to endocrinologist.<br>• Resume atezolizumab/placebo when symptoms are controlled and thyroid function is improving.
Symptomatic hypothyroidism (Grade 3)	• Hold atezolizumab.<br>• Initiate treatment with thyroid replacement hormone.<br>• Monitor TSH closely.<br>• Refer to an endocrinologist.<br>• Admit patient to the hospital for symptoms of myxedema (bradycardia, hypothermia and altered mental status).<br>• Resume atezolizumab/placebo when symptoms are controlled and thyroid function is improving.
Symptomatic hypothyroidism (Grade 4)	• Admit patient to the hospital.<br>• Discontinue atezolizumab/placebo and request unblinding.<br>– If receiving atezolizumab, initiate treatment with 1-2 mg/kg/day intravenous methylprednisolone or equivalent and convert to 1-2 mg/kg/day oral prednisone or equivalent upon improvement.<br>– When event resolves to Grade 1 or better, taper corticosteroids over ≥ 1 month.<br>– Initiate treatment with thyroid replacement hormone.<br>– Refer to an endocrinologist.
Asymptomatic hyperthyroidism (Grade 1)	**TSH ≥ 0.1 mU/L and < 0.5 mU/L:**<br>• Continue atezolizumab/placebo.<br>• Monitor TSH every 3-4 weeks.<br>• Consider patient referral to endocrinologist.<br>**TSH < 0.1 mU/L:**<br>• Follow guidelines for symptomatic hyperthyroidism (Grade 2).<br>• Consider patient referral to endocrinologist.
Symptomatic hyperthyroidism (Grade 2)	• Hold atezolizumab/placebo.<br>• Initiate treatment with anti-thyroid drug such as methimazole or carbimazole as needed.<br>• Refer to endocrinologist.<br>• Resume atezolizumab/placebo when symptoms are controlled and thyroid function is improving.

Table continued on next page.

Table 11. Management guidelines for endocrine events related to atezolizumab/placebo *(continued)*

Event	Management
Symptomatic hyperthyroidism (Grade 3, 4)	• Admit patient to the hospital.<br>• Discontinue atezolizumab/placebo and request unblinding.<br>- If receiving atezolizumab, initiate treatment with 1-2 mg/kg/day intravenous methylprednisolone or equivalent and convert to 1-2 mg/kg/day oral prednisone or equivalent upon improvement.<br>- When event resolves to Grade 1 or better, taper corticosteroids over ≥ 1 month.<br>• Initiate treatment with anti-thyroid drugs such as methimazole or carbimazole as needed.<br>• Refer to endocrinologist.
Symptomatic adrenal insufficiency, Grade 2-4	• Refer patient to endocrinologist.<br>• Perform appropriate imaging.<br>• Discontinue atezolizumab/placebo and request unblinding if required for medical management.<br>- If receiving atezolizumab initiate treatment with 1-2 mg/kg/day intravenous methylprednisolone or equivalent and convert to 1-2 mg/kg/day oral prednisone or equivalent upon improvement.<br>- When event resolves to Grade 1 or better, taper corticosteroids over ≥ 1 month.
Hypophysitis, (panhypopituitarism) Grades 2, 3, 4<br><br>*(Report as Endocrine disorders – Other, specify)*	• Perform brain MRI (pituitary protocol).<br>• Refer patient to endocrinologist.<br>• Initiate hormone replacement if clinically indicated.<br>• Discontinue atezolizumab/placebo and request unblinding if required for medical management.<br>- If receiving atezolizumab initiate treatment with 1-2 mg/kg/day intravenous methylprednisolone or equivalent and convert to 1-2 mg/kg/day oral prednisone or equivalent upon improvement.<br>- When event resolves to Grade 1 or better, taper corticosteroids over ≥ 1 month.
Hyperglycemia, Grades 1, 2,	• Continue atezolizumab/placebo.<br>• Investigate for diabetes. If patient develops Type 1 diabetes, treat as a Grade 4 event. If patient has not developed Type 1 diabetes, treat as per institutional guidelines.<br>• Monitor for glucose control.
Hyperglycemia Grade 3	• Withhold atezolizumab/placebo.<br>• Initiate treatment with insulin, if appropriate.<br>• Evaluate for diabetic ketoacidosis and manage as per institutional guidelines.<br>• If patient has developed Type 1 diabetes, treat as a Grade 4 event. If patient has not developed Type 1 diabetes, treat as per institutional guidelines.<br>• Monitor for glucose control.<br>• Resume atezolizumab/placebo when glucose levels are stable.

Table continued on next page.

Table 11. Management guidelines for endocrine events related to atezolizumab/placebo *(continued)*

Event	Management
Hyperglycemia, Grade 4	• Discontinue atezolizumab/placebo and request unblinding if required for medical management.<br>• Initiate treatment with insulin.<br>• Evaluate for diabetic ketoacidosis and manage as per institutional guidelines.<br>• Monitor for glucose control.

8.13 Ocular events

An ophthalmologist should evaluate visual complaints (e.g., uveitis, retinal events). Management guidelines for ocular events are provided in Table 12.

Table 12. Management guidelines for ocular events related to atezolizumab/placebo

Event	Management
Ocular event, Grade 1	• Continue atezolizumab/placebo.<br>• Patient referral to ophthalmologist is strongly recommended.<br>• Initiate treatment with topical corticosteroid eye drops and topical immunosuppressive therapy.<br>• If symptoms persist, manage as a Grade 2 event.
Ocular event, Grade 2	• Hold atezolizumab/placebo.<br>• Refer to ophthalmologist.<br>• If immune-mediated toxicity is suspected, permanently discontinue atezolizumab/placebo and request unblinding if required for medical management.<br>- If receiving atezolizumab, initiate treatment with topical corticosteroid eye drops and topical immunosuppressive therapy.
Ocular event, Grade 3 or 4	• Refer patient to ophthalmologist.<br>• Permanently discontinue atezolizumab/placebo and request unblinding if required for medical management.<br>- If receiving atezolizumab, initiate treatment with 1-2 mg/kg/day oral prednisone or equivalent.<br>- If event resolves to Grade 1 or better, taper corticosteroids over ≥ 1 month.

8.14 Infusion-related reaction and cytokine-release syndrome

No premedication is indicated for the administration of Cycle 1 of atezolizumab/placebo. However, patients who experience an infusion-related reaction (IRR) or cytokine-release syndrome (CRS) with Cycle 1 of atezolizumab/placebo may receive premedication with antihistamines, antipyretics and/or analgesics (e.g., acetaminophen) for subsequent infusions. Metamizole (dipyrone) is prohibited in treating atezolizumab-associated IRRs because of its potential for causing agranulocytosis.

IRRs are known to occur with the administration of monoclonal antibodies and have been reported with atezolizumab. These reactions, which are thought to be due to release of cytokines and/or other chemical mediators, occur within 24 hours of atezolizumab administration and are generally mild to moderate in severity.

CRS is defined as a supraphysiologic response following administration of any immune therapy that results in activation or engagement of endogenous or infused T cells and/or other immune effector cells. Symptoms can be progressive, always include fever at the onset, and may include hypotension, capillary leak (hypoxia), and end-organ dysfunction (Lee 2019). CRS has been well documented with chimeric antigen receptor T-cell therapies and bispecific T-cell engager antibody therapies but has also been reported with immunotherapies that target PD-1 or PD-L1 (Rotz 2017, Adashek 2019) including atezolizumab.

There may be significant overlap in signs and symptoms of IRRs and CRS, and in recognition of the challenges in clinically distinguishing between the two, consolidated guidelines for medical management of IRRs and CRS are provided in Table 13.

Severe COVID-19 appears to be associated with a cytokine-release syndrome (CRS) involving the inflammatory cytokines interleukin (IL)-6, IL-10, IL-2, and interferon- γ (Merad and Martin 2020). If a patient develops suspected CRS during the study, a differential diagnosis should include COVID-19, which should be confirmed or refuted through assessment of exposure history, appropriate laboratory testing, and clinical or radiologic evaluations per investigator judgment. If a diagnosis of COVID-19 is confirmed, the disease should be managed as per local or institutional guidelines.

Table 13. Management guidelines for infusion-related reactions and cytokine-release syndrome related to atezolizumab/placebo

Event	Management<br>*(Refer to Section 8.5.7 for management of chemotherapy)*
Grade 1[a]<br>Fever[b] with or without constitutional symptoms	• Immediately interrupt atezolizumab/placebo infusion and administer symptomatic treatment per institutional practice.<br>• Upon symptom resolution, wait for 30 minutes and then restart infusion at half the rate being given at the time of event onset.<br>• If the infusion is tolerated at the reduced rate for 30 minutes, the infusion rate may be increased to the original rate.<br>• If symptoms recur, discontinue atezolizumab/placebo.<br>• For subsequent infusions, consider administration of oral premedication with antihistamines, anti-pyretics, and/or analgesics, and monitor closely for IRRs and/or CRS.<br>• Manage with antipyretics and antihistamine until resolution.<br>• Monitor closely for additional symptoms.
Grade 2[a]<br>Fever[b] with hypotension not requiring vasopressors responding to fluids **and/or** Hypoxia requiring low-flow oxygen[d] by nasal cannula or blow-by	• Immediately interrupt atezolizumab/placebo infusion if infusion not completed.<br>• Upon symptom resolution, wait for 30 minutes and then restart infusion at half the rate being given at the time of event onset. If symptoms do not resolve within 30 minutes, discontinue atezolizumab/placebo and unblind.<br>• If symptoms recur after restart of the infusion discontinue atezolizumab/placebo.<br>• Administer symptomatic treatment per institutional practice.[c]<br>• For hypotension, administer IV fluid bolus as needed.<br>• If symptoms do not promptly resolve consider admission to monitor cardiopulmonary and other organ function closely (in the ICU, if appropriate). If hospitalization required, discontinue atezolizumab/placebo. Administer IV fluids as clinically indicated and manage constitutional symptoms and organ toxicities as per institutional practice.<br>○ Rule out other inflammatory conditions that can mimic CRS (e.g., sepsis). If no improvement within 24 hours, initiate workup and assess for signs and symptoms of HLH or MAS.<br>○ Consider IV corticosteroids (e.g., methylprednisolone 2 mg/kg/day or dexamethasone 10 mg every 6 hours).<br>○ Consider anti-cytokine therapy.<br>○ Continue hospitalization until complete resolution of symptoms. If no improvement within 24 hours, manage as per Grade 3.<br>• If evaluation fails to define etiology, consider unblinding if required for medical management.

Table continued on next page.

Table 13. Management guidelines for infusion-related reactions and cytokine-release syndrome related to atezolizumab/placebo *(continued)*

Grade 3[a]<br>Fever[b] with hypotension requiring a vasopressor (with or without vasopressin)<br>**and/or**<br>Hypoxia requiring high-flow oxygen[d] by nasal cannula, face mask, non-rebreather mask, or venturi mask	• Immediately stop atezolizumab/placebo infusion.<br>• Request unblinding to guide further management.<br>• Administer aggressive symptomatic treatment as per institutional.[c]<br>• Admit to the hospital.<br>• For hypotension, administer IV fluid bolus and vasopressor as needed.<br>• Monitor cardiopulmonary and other organ function closely; monitoring in the ICU is recommended. Administer IV fluids as clinically indicated and manage constitutional symptoms and organ toxicities as per institutional practice.<br>• Rule out other inflammatory conditions that can mimic CRS (*e.g.*, sepsis). If no improvement within 24 hours, initiate workup and assess for signs and symptoms of HLH or MAS.<br>• Administer IV corticosteroids (*e.g.*, methylprednisolone 2 mg/kg/day or dexamethasone 10 mg every 6 hours) if receiving atezolizumab.<br>• Consider anti-cytokine therapy.<br>• Hospitalize patient until complete resolution of symptoms. If no improvement within 24 hours, manage as per Grade 4, that is, admit patient to ICU and initiate hemodynamic monitoring, mechanical ventilation, and/or IV fluids and vasopressors as needed; for patients who are refractory to anti-cytokine therapy, experimental treatments[e] may be considered at the discretion of the investigator.<br>• Permanently discontinue atezolizumab/placebo.
Grade 4[a]<br>Fever[b] with hypotension requiring multiple vasopressors (excluding vasopressin)<br>**and/or**<br>Hypoxia requiring oxygen by positive pressure (e.g., CPAP, BiPAP, intubation and mechanical ventilation)	• Permanently stop atezolizumab/placebo.<br>• Administer symptomatic treatment.[c]<br>• Request unblinding to guide further management.<br>• Admit patient to ICU and initiate hemodynamic monitoring, mechanical ventilation, and/or IV fluids and vasopressors as needed. Monitor other organ function closely. Manage constitutional symptoms and organ toxicities as per institutional practice.<br>• Rule out other inflammatory conditions that can mimic CRS (e.g., sepsis). If no improvement to initial therapies, assess for signs and symptoms of HLH or MAS.<br>• Administer IV corticosteroids if receiving atezolizumab (e.g., methylprednisolone 2 mg/kg/day or dexamethasone 10 mg every 6 hours).<br>• Consider anti-cytokine therapy. For patients who are refractory to anti-cytokine therapy, experimental treatments[e] may be considered at the discretion of the investigator.<br>• Hospitalize patient until complete resolution of symptoms.

ASTCT = American Society for Transplantation and Cellular Therapy; BiPAP = bi-level positive airway pressure; CAR = chimeric antigen receptor; CPAP = continuous positive airway pressure; CRS = cytokine-release syndrome; HLH = hemophagocytic lymphohistiocytosis; IRR = infusion-related reaction; MAS = macrophage activation syndrome.

Note: The management guidelines have been adapted from NCCN guidelines for management of CAR T-cell-related toxicities (Version 2.2019).

a Grading system for management guidelines is based on ASTCT consensus grading for CRS. NCI CTCAE (version as specified in the protocol) should be used when reporting severity of IRRs,

CRS, or organ toxicities associated with CRS on the Adverse Event eCRF. Organ toxicities associated with CRS should not influence overall CRS grading.

b Fever is defined as temperature ≥ 38°C not attributable to any other cause. In patients who develop CRS and then receive anti-pyretic, anti-cytokine, or corticosteroid therapy, fever is no longer required when subsequently determining event severity (grade). In this case, the grade is driven by the presence of hypotension and/or hypoxia.

c Symptomatic treatment may include oral or IV antihistamines, anti-pyretics, analgesics, bronchodilators, and/or oxygen. For bronchospasm, urticaria, or dyspnea, additional treatment may be administered as per institutional practice.

d Low flow is defined as oxygen delivered at ≤ 6 L/min, and high flow is defined as oxygen delivered at > 6 L/min.

e Refer to Riegler 2019.

8.15 Management of pancreatic events

Symptoms of abdominal pain associated with elevations of amylase and lipase, suggestive of pancreatitis, have been associated with the administration of atezolizumab. The differential diagnosis of acute abdominal pain should include pancreatitis. Appropriate work-up should include an evaluation for ductal obstruction, as well as serum amylase and lipase tests. Management guidelines for pancreatic events, including pancreatitis, are provided in Table 14.

Table 14. Management guidelines for pancreatic events, including pancreatitis, related to atezolizumab/placebo

Event	Management
Amylase and/or lipase elevation, Grade 1	• Continue atezolizumab/placebo.<br>• Monitor amylase and lipase prior to dosing.
Amylase and/or lipase elevation, Grade 2	**Amylase and/or lipase > 1.5-2.0 x ULN:**<br>• Continue atezolizumab/placebo.<br>• Monitor amylase and lipase weekly.<br>• For prolonged elevation (e.g., > 3 weeks), hold atezolizumab/placebo.<br>• If non-immune etiology not identified, discontinue atezolizumab/placebo and request unblinding if required for medical management.<br>- If receiving atezolizumab, begin treatment with 1-2 mg/kg/day oral prednisone or equivalent.<br>- If event resolves to Grade 1 or better, taper corticosteroids over ≥ 1 month.<br>**Asymptomatic with amylase and/or lipase > 2.0-5.0 x ULN:**<br>• Treat as a Grade 3 or 4 event.

Table continued on next page.

Table 14. Management guidelines for pancreatic events, including pancreatitis, related to atezolizumab/placebo *(continued)*

Event	Management
Amylase and/or lipase elevation, Grade 3 or 4	• Hold atezolizumab/placebo.<br>• Refer patient to gastrointestinal specialist.<br>• Monitor amylase and lipase every 2 to 3 days until improved.<br>• May resume atezolizumab/placebo with next cycle if amylase and lipase are ≤ Grade 1.<br>• For prolonged elevation (e.g., > 3 weeks), continue to hold atezolizumab/placebo.<br>• If non-immune etiology not identified, discontinue atezolizumab/placebo and request unblinding.<br>- If receiving atezolizumab, begin treatment with 1-2 mg/kg/day oral prednisone or equivalent.<br>- If event resolves to Grade 1 or better, taper corticosteroids over ≥ 1 month.
Immune-mediated pancreatitis, Grade 2, 3, or 4	• Refer patient to gastrointestinal specialist.<br>• Discontinue atezolizumab/placebo and request unblinding.<br>- If receiving atezolizumab, initiate treatment with 1-2 mg/kg/day intravenous methylprednisolone or equivalent and convert to 1-2 mg/kg/day oral prednisone or equivalent upon improvement.<br>- If event does not improve within 48 hours after initiating corticosteroids, consider adding an immunosuppressive agent.<br>- If event resolves to Grade 1 or better, taper corticosteroids over ≥ 1 month.

8.16 Dermatologic events

Treatment-emergent rash has been associated with atezolizumab. The majority of cases of rash were mild in severity and self-limiting, with or without pruritus. A dermatologist should evaluate persistent and/or severe rash or pruritus. Although uncommon, cases of severe cutaneous adverse reactions such as Stevens-Johnson syndrome and toxic epidermal necrolysis have been reported with atezolizumab. A biopsy should be considered unless contraindicated. Management guidelines for dermatologic events are provided in Table 15.

Table 15. Management for dermatologic events related to atezolizumab/placebo

Event	Management
Dermatologic event, Grade 1	• Continue atezolizumab/placebo.<br>• Consider treatment with topical corticosteroids and/or other symptomatic therapy (e.g., antihistamines).

Table continued on next page.

Table 15. Management for dermatologic events related to atezolizumab/placebo *(continued)*

Event	Management
Dermatologic event, Grade 2	• Continue atezolizumab/placebo.<br>• Refer to dermatologist and consider skin biopsy.<br>• Initiate treatment with topical corticosteroids. If unresponsive to topical corticosteroids, consider oral prednisone 0.5 mg/kg/day.<br>• If rash does not improve, discontinue atezolizumab/placebo and consider unblinding if required for medical management.<br>- If receiving atezolizumab, initiate treatment with 10 mg/day oral prednisone or equivalent, increasing dose to 1-2 mg/kg/day if event does not improve within 48-72 hours.<br>- If event resolves to Grade 1 or better, taper corticosteroids over ≥ 1 month.
Bullous dermatitis, Grade 2	• Treat as Grade 3 or 4 Dermatologic event.
Dermatologic event, Grade 3 or 4	• Refer patient to dermatologist and consider skin biopsy.<br>• Discontinue atezolizumab/placebo and request unblinding.<br>- If receiving atezolizumab, initiate treatment with 10 mg/day oral prednisone or equivalent, increasing dose to 1-2 mg/kg/day if event does not improve within 48-72 hours.<br>- If event resolves to Grade 1 or better, taper corticosteroids over ≥ 1 month.
Stevens-Johnson syndrome or toxic epidermal necrolysis (any grade)	**Additional guidance for Stevens-Johnson syndrome or toxic epidermal necrolysis:**<br>• Withhold atezolizumab/placebo for suspected Stevens-Johnson syndrome or toxic epidermal necrolysis.<br>• Confirm diagnosis by referring patient to a specialist (dermatologist, ophthalmologist, or urologist as relevant) for evaluation and, if indicated, biopsy.<br>• Follow the applicable treatment and management guidelines as above for dermatologic event Grade 3 or 4.<br>• If Stevens-Johnson syndrome or toxic epidermal necrolysis is confirmed, permanently discontinue atezolizumab/placebo and request unblinding.

8.17 Neurological disorders

Myasthenia gravis and Guillain-Barré syndrome have been observed with single-agent atezolizumab. Patients may present with signs and symptoms of sensory and/or motor neuropathy. Diagnostic work-up is essential for an accurate characterization to differentiate between alternative etiologies. Management guidelines for neurologic disorders are provided in Table 16.

Table 16. Treatment management guidelines for neurological disorders related to atezolizumab/placebo

<table>
<tr><th>Event</th><th colspan="2">Management</th></tr>
<tr><td>Immune-mediated neuropathy, Grade 1</td><td colspan="2">• Continue atezolizumab/placebo.
• Investigate etiology.</td></tr>
<tr><td>Immune-mediated sensory neuropathy, Grade 2</td><td>Grade 2 while receiving paclitaxel*:
• Withhold atezolizumab/placebo.
• Oral steroids should not be initiated for first or second episodes.
• Resume atezolizumab/placebo if event resolves to Grade 1 or better within 3 weeks.
• If event does not resolve to Grade 1 or better within 3 weeks:
- Refer patient to neurologist
- Consider unblinding if required for medical management.
- If receiving atezolizumab, begin treatment with 1-2 mg/kg/day oral prednisone or equivalent.
- If event resolves to Grade 1 or better, taper corticosteroids over ≥ 1 month.</td><td>Grade 2 if not receiving paclitaxel*:
• Refer patient to neurologist.
• Discontinue atezolizumab/placebo.
• Request unblinding if required for medical management.
- If receiving atezolizumab, begin treatment with 1-2 mg/kg/day oral prednisone or equivalent.
- If event resolves to Grade 1 or better, taper corticosteroids over ≥ 1 month.</td></tr>
<tr><td>Immune-mediated motor neuropathy Grade 2</td><td colspan="2">• Refer patient to neurologist.
• Discontinue atezolizumab/placebo and consider unblinding.
- If receiving atezolizumab, begin treatment with 1-2 mg/kg/day oral prednisone or equivalent.
- If event resolves to Grade 1 or better, taper corticosteroids over ≥ 1 month.</td></tr>
<tr><td>Immune-mediated neuropathy, Grade 3 or 4</td><td colspan="2">• Refer patient to neurologist.
• Discontinue atezolizumab/placebo and request unblinding if required for medical management.
- If receiving atezolizumab, begin treatment with 1-2 mg/kg/day oral prednisone or equivalent.
- If event resolves to Grade 1 or better, taper corticosteroids over ≥ 1 month.</td></tr>
<tr><td>Immune-mediated facial paresis or cranial nerve disorder (any grade)</td><td colspan="2">• Refer patient to neurologist.
• Discontinue atezolizumab/placebo and request unblinding if required for medical management.
– If receiving atezolizumab, begin treatment with 1-2 mg/kg/day oral prednisone or equivalent.
– If event resolves to Grade 1 or better, taper corticosteroids over ≥ 1 month.</td></tr>
</table>

Table continued on next page.

Table 16. Treatment management guidelines for neurological disorders related to atezolizumab/placebo *(continued)*

Myasthenia gravis, Guillain-Barré syndrome, and immune-mediated myelitis (any grade)	• Refer patient to neurologist and initiate treatment as per institutional guidelines.<br>• Discontinue atezolizumab/placebo and request unblinding.<br>- If receiving atezolizumab, begin treatment with 1-2 mg/kg/day oral or intravenous prednisone or equivalent.<br>- If event resolves to Grade 1 or better, taper corticosteroids over ≥ 1 month.
* Refer to Section 8.5.4 for concurrent dose modifications for paclitaxel.	

8.18 Immune-mediated meningoencephalitis

Immune-mediated meningoencephalitis is an identified risk associated with the administration of atezolizumab. Immune-mediated meningoencephalitis should be suspected in any patient presenting with signs or symptoms suggestive of meningitis or encephalitis, including, but not limited to, headache, neck pain, confusion, seizure, motor or sensory dysfunction, and altered or depressed level of consciousness. Encephalopathy from metabolic or electrolyte imbalances needs to be distinguished from potential meningoencephalitis resulting from infection (bacterial, viral, or fungal) or progression of malignancy, or secondary to a paraneoplastic process.

All patients being considered for meningoencephalitis should be urgently evaluated with a CT scan and/or MRI scan of the brain to evaluate for metastasis, inflammation, or edema. If deemed safe by the treating physician, a lumbar puncture should be performed and a neurologist should be consulted.

Patients with signs and symptoms of meningoencephalitis, in the absence of an identified alternate etiology, should be treated according to the guidelines in Table 17.

Table 17. Management guidelines for immune-mediated meningoencephalitis related to atezolizumab/placebo

Event	Management
Immune-mediated meningoencephalitis, all grades	• Refer patient to neurologist.<br>• Permanently discontinue atezolizumab/placebo and request unblinding.<br>- If receiving atezolizumab, initiate treatment with 1-2 mg/kg/day IV methylprednisolone or equivalent and convert to 1-2 mg/kg/day oral prednisone or equivalent upon improvement.<br>- If event does not improve within 48 hours after initiating corticosteroids, consider adding an immunosuppressive agent.<br>- If event resolves to Grade 1 or better, taper corticosteroids over ≥ 1 month.

8.19 Management and Evaluation of Immune-Mediated Renal Events

Immune-mediated nephritis has been associated with the administration of atezolizumab. Eligible patients must have adequate renal function. Renal function, including serum creatinine, should be monitored throughout study treatment. Patients with abnormal renal function should be evaluated and treated for other more common etiologies (including prerenal and postrenal causes, and concomitant medications such as non-steroidal anti-inflammatory drugs). Patients with signs and symptoms of nephritis, in the absence of an identified alternate etiology, should be treated according to the guidelines in Table 18.

Refer to a renal specialist if clinically indicated. A renal biopsy may be required to enable a definitive diagnosis and appropriate treatment.

Table 18. Management and evaluation of immune-mediated renal events (potential immune-mediated nephritis)

Event	**Management**<br>*(Refer to Section 8.5.5 for management of chemotherapy.)*
Renal event,<br>Grade 1	• Continue atezolizumab/placebo.<br>• If creatinine value is above normal limits or protein is found in the urine, monitor weekly until value resolves to within normal limits or to baseline value.
Renal event,<br>Grade 2	• Hold atezolizumab/placebo and recheck creatinine in 3-7 days.<br>- If creatinine is improving but remains Grade 2, continue to hold atezolizumab/placebo and monitoring creatinine weekly until ≤ Grade 1.<br>- If repeat creatinine value does not improve but remains Grade 2, refer to nephrologist for evaluation and continue to hold atezolizumab/placebo.<br>- If evaluation fails to define non-immune etiology OR defines an immune etiology, discontinue atezolizumab/placebo and request unblinding for medical management.<br>• If patient was unblinded and was receiving atezolizumab, initiate treatment with 1–2 mg/kg/day oral prednisone or equivalent. When event resolves to Grade 1 or better, taper corticosteroids over ≥ 1 month.
Renal event,<br>Grade 3 or 4	• Refer to nephrologist for evaluation and consideration of renal biopsy to establish etiology of renal injury.<br>• Discontinue atezolizumab/placebo and request unblinding.<br>- If receiving atezolizumab, initiate treatment with 1–2 mg/kg/day oral prednisone or equivalent.<br>- If event does not improve within 48 hours after initiating corticosteroids, consider adding an immunosuppressive agent.<br>- When event resolves to Grade 1 or better, taper corticosteroids over ≥ 1 month.

8.20 Immune-mediated myositis

Immune-mediated myositis has been associated with the administration of atezolizumab. Myositis or inflammatory myopathies are a group of disorders sharing the common feature of inflammatory muscle injury; dermatomyositis and polymyositis are amongst the most common disorders. Initial diagnosis is based on clinical (muscle weakness, muscle pain, skin rash in dermatomyositis), biochemical (serum creatine-kinase increase), and imaging (electromyography/MRI) features and is confirmed with a muscle-biopsy. Patients with possible myositis should be referred to a rheumatologist or neurologist. Patients with possible myositis should be monitored for signs of myocarditis.

Patients with signs and symptoms myositis, in the absence of an identified alternate etiology, should be treated according to the guidelines in Table 19.

Table 19. Management and evaluation of immune-mediated myositis

Event	**Management**<br>*(Refer to Sections 8.5.5 and 8.6.9 for management of chemotherapy.)*
Myositis,<br>Grade 1 (mild pain with elevated CK)	• Hold atezolizumab/placebo.<br>• Refer patient to rheumatologist or neurologist.<br>• If evaluation fails to define non-immune etiology OR defines an immune etiology, discontinue atezolizumab/placebo and request unblinding if required for medical management.<br>- If receiving atezolizumab, initiate treatment with 1-2 mg/kg/day IV methylprednisolone or equivalent and convert to 1-2 mg/kg/day oral prednisone or equivalent upon improvement.<br>- When CK elevation resolves and pain improved, taper corticosteroids over ≥ 1 month.<br>• If non-immune etiology identified, may resume or discontinue atezolizumab/placebo per investigator discretion.
Myositis,<br>Grade 2 (moderate pain with weakness)	• Hold atezolizumab/placebo.<br>• Refer patient to rheumatologist or neurologist.<br>• If evaluation fails to define non-immune etiology OR defines an immune etiology, discontinue atezolizumab/placebo and request unblinding if required for medical management.<br>- If receiving atezolizumab, initiate treatment with 1-2 mg/kg/day IV methylprednisolone or equivalent and convert to 1-2 mg/kg/day oral prednisone or equivalent upon improvement.<br>- If event does not improve within 48 hours after initiating corticosteroids, consider adding an immunosuppressive agent.<br>- If event resolves to Grade 1 or better, taper corticosteroids over ≥ 1 month.<br>• If non-immune etiology identified, may resume or discontinue atezolizumab/placebo per investigator discretion.
Myositis,<br>Grade 3 (pain with severe weakness)	• Hold atezolizumab/placebo.<br>• Refer patient to neurologist or rheumatologist.<br>• Evaluate for associated myocarditis and consider consulting cardiologist.<br>• Respiratory support may be required in more severe cases.<br>• Discontinue atezolizumab/placebo and request unblinding.<br>- If receiving atezolizumab, initiate treatment with 1-2 mg/kg/day IV methylprednisolone or equivalent or higher-dose bolus if patient is severely compromised (e.g., cardiac or respiratory symptoms, dysphagia, or weakness that severely limits mobility) and convert to 1-2 mg/kg/day oral prednisone or equivalent upon improvement.<br>- If event does not improve within 48 hours after initiating corticosteroids, consider adding an immunosuppressive agent.<br>- If event resolves to Grade 1 or better, taper corticosteroids over ≥ 1 month.

8.21 Hemophagocytic lymphohistiocytosis and macrophage activation syndrome

Immune-mediated reactions may involve any organ system and may lead to hemophagocytic lymphohistiocytosis (HLH) and macrophage activation syndrome (MAS).

Clinical and laboratory features of severe CRS overlap with HLH, and HLH should be considered when CRS presentation is atypical or prolonged.

Patients with suspected HLH should be diagnosed according to published criteria by McClain and Eckstein (2014). A patient should be classified as having HLH if five of the following eight criteria are met:

- Fever ≥ 38.5°C
- Splenomegaly
- Peripheral blood cytopenia consisting of at least two of the following:
  - Hemoglobin < 90 g/L (9 g/dL) (< 100 g/L [10 g/dL] for infants < 4 weeks old)
  - Platelet count < 100 × 10^9/L (100,000/μL)
  - ANC < 1.0 × 10^9/L (1000/μL)
- Fasting triglycerides > 2.992 mmol/L (265 mg/dL) and/or fibrinogen < 1.5 g/L (150 mg/dL)
- Hemophagocytosis in bone marrow, spleen, lymph node, or liver
- Low or absent natural killer cell activity
- Ferritin > 500 mg/L (500 ng/mL)
- Soluble interleukin 2 (IL-2) receptor (soluble CD25) elevated ≥ 2 standard deviations above age-adjusted laboratory-specific norms

Patients with suspected MAS should be diagnosed according to published criteria for systemic juvenile idiopathic arthritis by Ravelli et al. (2016). A febrile patient should be classified as having MAS if the following criteria are met:

- Ferritin > 684 mg/L (684 ng/mL)
- At least two of the following:
  - Platelet count ≤ 181 × 10^9/L (181,000/μL)
  - AST ≥ 48 U/L
  - Triglycerides >1.761 mmol/L (156 mg/dL)
  - Fibrinogen ≤ 3.6 g/L (360 mg/dL)

Patients with suspected HLH or MAS should be treated according to the guidelines in Table 20.

Table 20. Management and evaluation of hemophagocytic lymphohistiocytosis and macrophage activation syndrome

Event	Management
Suspected HLH or MAS	• Permanently discontinue atezolizumab/placebo and contact Protocol Officer.<br>• Consider patient referral to hematologist.<br>• Initiate supportive care, including intensive care monitoring if indicated per institutional guidelines.<br>• Initiate IV corticosteroids and/or an immunosuppressive agent.<br>• If event does not respond to treatment within 24 hours, contact Protocol Officer and initiate treatment as appropriate according to published guidelines (La Rosée 2015; Schram and Berliner 2015; La Rosée 2019).<br>• If event does not improve within 48 hours after initiating corticosteroids, consider adding an immunosuppressive agent.<br>• If event resolves to Grade 1 or better, taper corticosteroids over ≥1 month.

HLH=hemophagocytic lymphohistiocytosis; MAS=macrophage activation syndrome.

8.22 Immune-mediated cardiac toxicity

8.22.1 *Evaluation and management of cardiac toxicity*

- Cardiac toxicity is a potential toxicity with the use of atezolizumab/placebo. Patients should be monitored for signs and symptoms of left ventricular dysfunction and cardiac arrhythmias. Consider echocardiogram and ECG.
- For Grade 2 symptomatic cardiac toxicities refer to cardiologist for a cardiac evaluation. Consider endomyocardial biopsy and initiation of oral steroids.
- For Grade 3 or Grade 4 cardiac toxicities, hospitalize, obtain a cardiology consult and obtain endomyocardial biopsy unless contraindicated. Treat with intravenous steroids followed by high dose oral steroids.

8.22.2 *Management of atezolizumab/placebo during episodes of symptomatic cardiac toxicity*

- If paclitaxel and carboplatin or AC/EC are discontinued for **non-immune** cardiac toxicity, atezolizumab/placebo should also be discontinued.
- If atezolizumab/placebo must be discontinued due to immune-mediated cardiac toxicity, paclitaxel and carboplatin may be resumed per investigator discretion when steroid taper is completed and cardiac toxicity has resolved to ≤ Grade 1. AC/EC must be discontinued.
- See Table 21 for management of symptomatic cardiac toxicities related to atezolizumab/placebo.

8.22.3 *Immune-mediated myocarditis*

Immune-mediated myocarditis has been associated with the administration of atezolizumab. Immune-mediated myocarditis should be suspected in any patient presenting with signs or symptoms suggestive of myocarditis, including, but not limited to, laboratory (e.g., B-type natriuretic peptide) or cardiac imaging abnormalities, dyspnea, chest pain, palpitations, fatigue, decreased exercise tolerance, or syncope. Myocarditis

may also be a clinical manifestation of myositis or associated with pericarditis (see Section 8.22.4) and should be managed accordingly. Immune-mediated myocarditis needs to be distinguished from myocarditis resulting from infection (commonly viral, e.g., in a patient who reports a recent history of gastrointestinal illness), ischemic events, underlying arrhythmias, exacerbation of preexisting cardiac conditions, or progression of malignancy.

All patients with possible myocarditis should be urgently evaluated by a cardiologist and assessed with serum troponin, ECG, chest imaging, echocardiogram and considered for cardiac MRI as appropriate per institutional guidelines. An endomyocardial biopsy may be considered to enable a definitive diagnosis and appropriate treatment, if clinically indicated.

Patients with signs and symptoms of myocarditis, in the absence of an identified alternate etiology, should be treated according to the guidelines in Table 21.

8.22.4 ***Immune-mediated pericardial disorders***

Immune-mediated pericarditis should be suspected in any patient presenting with chest pain and may be associated with immune-mediated myocarditis (see Section 8.22.3).

Immune-mediated pericardial effusion and cardiac tamponade should be suspected in any patient presenting with chest pain associated with dyspnea or hemodynamic instability.

Patients should be evaluated for other causes of pericardial disorders such as infection (commonly viral), cancer related (metastatic disease or chest radiotherapy), cardiac injury related (post myocardial infarction or iatrogenic), and autoimmune disorders, and should be managed accordingly.

All patients with suspected pericardial disorders should be urgently evaluated by performing an ECG, chest X-ray, transthoracic echocardiogram, and cardiac MRI as appropriate per institutional guidelines. A cardiologist should be consulted. Pericardiocentesis should be considered for diagnostic or therapeutic purposes, if clinically indicated.

Patients with signs and symptoms of pericarditis, pericardial effusion, or cardiac tamponade, in the absence of an identified alternate etiology, should be treated according to the guidelines in Table 21. Discontinue treatment with atezolizumab for documented ≥ Grade 1 pericarditis and conduct a detailed cardiac evaluation to determine the etiology and manage accordingly.

Table 21. Treatment modifications and instructions for **symptomatic** cardiac toxicities including myocarditis related to atezolizumab/placebo

<table>
<tr><th>Event</th><th>Grade</th><th>Management
(Refer to Section 8.6.1 for management of chemotherapy)</th></tr>
<tr><td rowspan="2">Myocarditis, Pericarditis, or Heart Failure</td><td>1,2</td><td>• Consult cardiologist to conduct cardiac evaluation including LVEF assessment, troponin, and ECG.
• Initiate treatment as per institutional guidelines and consider antiarrhythmic drugs and other interventions as appropriate.
• Discontinue atezolizumab/placebo.
• Request unblinding if required for medical management.
• If receiving atezolizumab, initiate treatment with 1-2 mg/kg/day oral prednisone or equivalent.
• If Grade 2 myocarditis does not improve within 48 hours after initiating corticosteroids, consider adding an immunosuppressive agent.
• When event resolves to Grade 1 or better, taper corticosteroids over ≥ 1 month.
• If event does not resolve to grade 1 or better, contact Protocol Officer.</td></tr>
<tr><td>3,4</td><td>• Admit to the hospital.
• Consult cardiologist to conduct cardiac evaluation including LVEF assessment, troponin, and ECG.
• Initiate treatment as per institutional guidelines and consider antiarrhythmic drugs, temporary pacemaker, ECMO, VAD, or pericardiocentesis as appropriate.
• Discontinue atezolizumab/placebo and request unblinding.
- If receiving atezolizumab, initiate treatment with 1-2 mg/kg/day oral prednisone or equivalent.
- If event does not improve within 48 hours after initiating corticosteroids, consider adding an immunosuppressive agent.
- When event resolves to Grade 1 or better, taper corticosteroids over ≥ 1 month.</td></tr>
<tr><td>Left Ventricular Dysfunction</td><td>3,4</td><td>• Admit to the hospital.
• Consult cardiologist to conduct cardiac evaluation including LVEF assessment, troponin, and ECG.
• Endomyocardial biopsy should be considered unless medical condition precludes the biopsy.
• Discontinue atezolizumab/placebo and request unblinding if required for medical management.
- If receiving atezolizumab, initiate treatment with 1-2 mg/kg/day oral prednisone or equivalent.
- If event does not improve within 48 hours after initiating corticosteroids, consider adding an immunosuppressive agent.
- When event resolves to Grade 1 or better, taper corticosteroids over ≥ 1 month.</td></tr>
</table>

Table continued on next page.

Table 21. Treatment modifications and instructions for **symptomatic** cardiac toxicities including myocarditis related to atezolizumab/placebo *(continued)*

Event	Grade	Management<br>*(Refer to Section 8.6.1 for management of chemotherapy)*
Other Cardiac Toxicities (not listed in Table 22)	1	• Hold atezolizumab/placebo.<br>• Consult cardiologist to conduct cardiac evaluation including LVEF assessment, troponin, and ECG.<br>• If immune mediated etiology identified, request unblinding if required for medical management.<br>- If receiving atezolizumab, initiate treatment with 1-2 mg/kg/day oral prednisone or equivalent.<br>- When event resolves to Grade 1 or better, taper corticosteroids over ≥ 1 month.
	Grade 2 requiring discontinuation of atezolizumab/ placebo (see Table 22)	• Consult cardiologist to conduct cardiac evaluation including LVEF assessment, troponin, and ECG.<br>• Consider endomyocardial biopsy.<br>• Discontinue atezolizumab/placebo and request unblinding if required for medical management.<br>- If receiving atezolizumab, initiate treatment with 1-2 mg/kg/day oral prednisone or equivalent.<br>- When event resolves to Grade 1 or better, taper corticosteroids over ≥ 1 month.
	Grade 2 NOT requiring discontinuation of atezolizumab/ placebo unless immune etiology identified	• Hold atezolizumab/placebo.<br>• Consult cardiologist to conduct cardiac evaluation including LVEF assessment, troponin, and ECG.<br>• If evaluation fails to define non-immune etiology, consider endomyocardial biopsy.<br>• If immune mediated etiology identified, request unblinding if required for medical management.<br>- If receiving atezolizumab, initiate treatment with 1-2 mg/kg/day oral prednisone or equivalent.<br>- When event resolves to Grade 1 or better, taper corticosteroids over ≥ 1 month.
	3,4	• Admit to the hospital.<br>• Consult cardiologist to conduct cardiac evaluation including LVEF assessment, troponin, and ECG.<br>• Endomyocardial biopsy should be performed unless medical condition precludes the biopsy.<br>• Discontinue atezolizumab/placebo and request unblinding.<br>- If receiving atezolizumab, initiate treatment with 1-2 mg/kg/day oral prednisone or equivalent.<br>- If event does not improve within 48 hours after initiating corticosteroids, consider adding an immunosuppressive agent.<br>- When event resolves to Grade 1 or better, taper corticosteroids over ≥ 1 month.

Table 22. CTCAE grade 2 other cardiac disorders

**CTCAE Grade 2 Other Cardiac Disorders AEs That Prohibit Further Atezolizumab/Placebo Therapy**	
CARDIAC DISORDERS – CTCAE v4.0	
Adverse Event	Grade 2 Criteria
Acute coronary syndrome	Symptomatic, progressive angina; cardiac enzymes normal; hemodynamically stable
Atrioventricular block complete	Non-urgent intervention indicated
Mobitz (type) II atrioventricular block	Symptomatic; medical intervention indicated
Mobitz type I	Symptomatic; medical intervention indicated
Pericarditis	Symptomatic pericarditis (e.g., chest pain)
Right ventricular dysfunction	Symptoms with mild to moderate activity or exertion
Ventricular tachycardia	Non-urgent medical intervention indicated

8.23 Management of atezolizumab/placebo during an asymptomatic decrease in LVEF

The results of the LVEF assessments after surgery will be used to determine if atezolizumab/placebo can be continued postoperatively (refer to Table 23).

- Echocardiogram is the preferred method for assessment of LVEF. However, LVEF assessment by MUGA scan is permitted.
- ***All LVEF assessments should be performed by the same method*** (either echocardiogram or MUGA scan) that was performed at baseline.
- Investigators are strongly urged to schedule the LVEF assessment at the same cardiac imaging facility that performed the patient's baseline LVEF assessment.

Table 23. Resumption of atezolizumab/placebo following surgery based on LVEF assessment after surgery

The following are instructions for patients who have an **asymptomatic decrease in LVEF** determined by ECHO/MUGA performed after surgery prior to resuming atezolizumab/placebo.	
**LVEF**	**Asymptomatic decrease in LVEF after surgery prior to resuming atezolizumab/placebo**
**≥ 50%**	Resume atezolizumab/placebo.
**45-49%**	Hold atezolizumab/placebo and repeat ECHO/MUGA in 6 weeks.<br>***Treatment rules based on repeat LVEF results:***<br>• If LVEF result is ≥ 50%, resume atezolizumab/placebo.<br>• If LVEF result is 45-49%, hold atezolizumab/placebo and recheck in 3 weeks.<br>– If < 50%, discontinue atezolizumab/placebo and manage per Table 21 for Grade 2 heart failure.<br>– If LVEF result is > 50%, resume atezolizumab/placebo.<br>• If LVEF result is < 45%, discontinue atezolizumab/placebo and manage per Table 21 for Grade 2 heart failure.
**< 45%**	Discontinue atezolizumab/placebo and manage per Table 21 for Grade 2 heart failure.

8.24 Liver dysfunction (Hy's Law)

Hy's Law is based on the observation that pure hepatocellular injury sufficient to cause hyperbilirubinemia is an ominous indicator of the potential for a drug to cause serious liver injury. A diagnosis of potential drug-induced liver injury caused by a study drug can only be determined/inferred by excluding other potential causes of liver injury (e.g., other drugs or viral hepatitis) and by ruling out an obstructive cause for the elevated bilirubin (e.g., alkaline phosphatase should not be substantially elevated) (FDA-Guidance 2009, Temple 2006).

8.24.1 *Definition of cases potentially meeting Hy's Law criteria*

Patients who present with the following laboratory abnormalities should be evaluated further to definitively determine the etiology of the abnormal laboratory values:

- *Patients with AST or ALT baseline values within the normal range* who subsequently present with AST or ALT > 3 times the ULN concurrent with a total bilirubin > 2 times the ULN with no evidence of hemolysis and an alkaline phosphatase < 2 times the ULN or not available.
- *Patients with pre-existing AST or ALT baseline values above the normal range* who subsequently present with AST or ALT > 2 times the baseline values and > 3 times the ULN, or ≥ 8 times the ULN (whichever is smaller) concurrent with a total bilirubin of > 2 times the ULN and increased by one ULN over baseline or > 3 times the ULN (whichever is smaller) with no evidence of hemolysis and an alkaline phosphatase < 2 times the ULN or not available.

8.24.2 *Evaluation of potential Hy's Law cases*

The patient should return to the investigational site to be evaluated as soon as possible, preferably within 48 hours from awareness of the abnormal results, and **managed as Grade 3 or 4 hepatotoxicity per Table 9**. This evaluation should include laboratory tests, detailed history, and physical assessment. In addition to repeating AST and ALT, laboratory tests should include albumin, creatine kinase, total bilirubin, direct and indirect bilirubin, gamma-glutamyl transferase (GGT), international normalized ratio (INR) and alkaline phosphatase. A detailed history, including relevant information, such as review of ethanol, recreational drug and supplement consumption, family history, sexual history, travel history, history of contact with a jaundiced patient, surgery, blood transfusion, history of liver or allergic disease, and work exposure, should be collected. Further testing for acute hepatitis A, B, or C infection and liver imaging (e.g., biliary tract) may be warranted. The possibility of recurrent disease should be considered.

Potential Hy's Law cases should be reported as serious adverse events (see Sections 10.4.2 and 10.7.2).

9.0 DRUG INFORMATION

9.1 Chemotherapy

The investigational agent, atezolizumab/placebo, required for completion of this study will be centrally supplied and distributed by [redacted] Depending on local legislation, cyclophosphamide, carboplatin, doxorubicin, epirubicin, or paclitaxel may also be considered an investigational agent in this study. Where permitted by regulatory requirements, sites will obtain and utilize commercially available cyclophosphamide, carboplatin, doxorubicin, epirubicin, and paclitaxel.

NSABP sites:

[redacted]

[redacted]

[redacted]

[redacted]

GBG sites

[redacted]

9.2 Atezolizumab/placebo description and supply

[redacted]

[redacted]

[redacted] [redacted]

[redacted]

[redacted]

[redacted]

[redacted]

9.4 Preparation and administration

Administration of atezolizumab or placebo will be performed in a setting with emergency medical facilities and staff who are trained to monitor for and respond to medical emergencies. Patients who experience an infusion-related reaction (IRR) with the first infusion may receive premedication with antihistamines or antipyretics or analgesics (e.g., acetaminophen) for subsequent infusions. The initial dose of atezolizumab or placebo will be delivered over 60 (± 15) minutes. Subsequent infusions will be delivered over 30 (± 10) minutes if the previous infusion was tolerated without infusion-associated adverse events or 60 (± 15) minutes if the patient experienced an infusion-associated adverse event with the previous infusion. Vital signs are to be measured before, as well as during, and after infusions, if clinically indicated.

Guidelines for medical management of infusion-related reactions and cytokine-release syndrome are provided in the Atezolizumab Investigator's Brochure and Sections 8.14.

Atezolizumab/placebo must be prepared under appropriate aseptic conditions as it does not contain antimicrobial preservatives. The prepared solution for infusion should be used immediately to limit microbial growth in case of potential accidental contamination. If not used immediately, in-use storage time and conditions prior to use are the responsibility of the user. For flat or fixed dosing, (e.g., 800 mg, 840 mg, 1200 mg, or 1680 mg) in IV infusion bags, the dose solution may be stored at 2°C-8°C (36°F-46°F) for 24 hours or at ambient temperature ≤ 25C° (77°F) for 8 hours. This time includes storage and time for administration for infusion. If the dose solution is stored at 2°C–8°C (36°F–46°F), it should be removed from refrigeration and allowed to reach room temperature prior to administration. Do not shake or freeze infusion bags containing the dose solution.

9.5 Procurement of atezolizumab/placebo

Atezolizumab/placebo will be shipped directly to the investigator whose sites are participating in NSABP B-59/GBG 96-GeparDouze.

9.5.1 *Shipping*

Vials of atezolizumab/placebo will be shipped at refrigerated temperature by overnight express delivery ***Monday through Thursday excluding holidays.***

9.5.2 *Storage/stability*

The vials containing atezolizumab/placebo must be refrigerated at 2°C-8°C (36°F-46°F) upon receipt until use. Vials should not be used beyond the expiration date provided by the manufacturer. No preservative is used in the atezolizumab drug product or placebo; therefore, each vial is intended for single use only. Discard any unused portion of drug left in a vial. Vial contents should not be frozen or shaken and should be protected from light.

9.6 **Transfer of atezolizumab/placebo**

Atezolizumab/placebo may not be used outside the scope of NSABP B-59/GBG 96-GeparDouze, nor transferred or licensed to any party not participating in this clinical trial.

9.7 **Destruction of atezolizumab/placebo**

- At the end of an infusion for an individual patient, any remaining or unused atezolizumab/placebo should be destroyed at the site according to the institution's policy for drug destruction. At the completion of the study, all unused atezolizumab/placebo will also be destroyed at the site as per institutional policy for drug destruction after the monitoring review is completed.
- Written documentation of destruction must contain the following:
  - identity (Patient ID number) of study medication destroyed;
  - quantity of study medication destroyed;
  - date of destruction (date discarded in designated hazardous container for destruction); and
  - name and signature of the person who discarded the atezolizumab/placebo in a hazardous container for destruction.

9.8 **Drug accountability**

The investigator, or a responsible party designated by the investigator, must maintain an accurate record of the receipt, return, and destruction of all atezolizumab/placebo vials received through the NSABP B-59/GBG 96-GeparDouze study using an investigational agent accountability record form.

9.9 **Procedures for unblinding**

Unblinding of the study therapy assignment of a patient should be carried out by the investigator only to 1) serve emergency purposes 2) guide management of potential immune-mediated toxicities as defined in the protocol or 3) when needed to guide therapy of disease recurrence. Requests for unblinding of patients found to have residual invasive breast cancer at surgery is strongly discouraged. Capecitabine and olaparib can be administered without unblinding of study medication if required. Investigators are encouraged to proceed with radiation therapy as soon as possible in all patients following surgery. Once unblinding has occurred, study medication must be discontinued.

When a decision is made to request unblinding, investigators must request unblinding of a patient's study therapy assignment and the reason for the unblinding request. After the confirmation of the request and the indication for unblinding is obtained, the investigator will be notified immediately of the patient's treatment assignment. A computer record will be created to identify the patient as having been unblinded. The institution is responsible for providing continued follow-up (for patients whose treatment assignment has been unblinded) on the same schedule as indicated in the study protocol (see Table 2) for patients who have not been unblinded, unless otherwise specified.

10.0 ADVERSE EVENT REPORTING

The investigator is responsible for the detection and documentation of events meeting the criteria and definition of an adverse event (AE) or a serious adverse event (SAE), as provided in this protocol. NSABP North American sites must submit expedited adverse event report forms and their supporting documentation to ProPharma and GBG sites must submit to [redacted] according to the instructions in Sections 10.7 and 10.8.

10.1 Double-blind study drug

This is a double-blind study and includes an investigational agent with placebo. When an AE occurs that is expected for the investigational agent, the AE should be considered expected for the blinded study therapy. Conversely, when an adverse event occurs that is not listed for the investigational agent, the AE should be considered to be unexpected for the blinded therapy, unless it is expected for the chemotherapeutic agents being administered concurrently with the investigational agent.

10.2 Definition of an AE

An AE can be any unfavorable and unintended sign (including an abnormal laboratory finding), symptom, or disease temporally associated with the use of a medicinal (investigational) product, whether or not considered related to the medicinal (investigational) product occurring in a patient participating in NSABP B-59/GBG 96-GeparDouze. The event does not need to be causally related to study therapy or other requirements of the NSABP B-59/GBG 96-GeparDouze trial to be considered an AE.

- Examples of an AE include, but are not limited to, the following:
  - Any toxicity related to study therapy.
  - Any clinically significant worsening of a pre-existing condition.
  - An AE occurring from an overdose of any study therapy, whether accidental or intentional. Overdose is a dose greater than that specified in the protocol.
  - An AE occurring from abuse (e.g., use for nonclinical reasons) of any of the agents included in the study therapy.
  - An AE that has been associated with the discontinuation of the use of any of the agents included in the study therapy.
  - An AE occurring during a clinical study that is not related to the study therapy, but is considered by the investigator or sponsor to be related to the study requirements, for example, an AE may be an untoward event related to a medical procedure required by the protocol.
- A laboratory test result should be reported as an AE if it meets any of the following criteria:
  - Accompanied by clinical symptoms.
  - Results in a change in study treatment (e.g., dosage modification, treatment interruption, or treatment discontinuation).
  - Results in medical intervention (e.g., potassium supplementation for hypokalemia) or treatment discontinuation.
  - Clinically significant per the investigator.
- Examples of clinical events that should **not** be considered AEs:
  - Medical or surgical procedure (e.g., endoscopy, appendectomy). Note, the condition that leads to the procedure may be an AE, but the procedure itself is not.
  - Anticipated day-to-day fluctuations of pre-existing disease(s) or condition(s) present or detected at the start of the study that do not worsen.

10.3 Definition of an SAE

An SAE is any untoward medical occurrence that at any dose:

- Results in death,
- Is life-threatening,

  NOTE: The term "life-threatening" in the definition of "serious" refers to an event in which the patient was at risk of death at the time of the event. It does not refer to an event, which might have hypothetically caused death, if it were more severe.

- Requires inpatient hospitalization or prolongation of existing hospitalization

  Hospitalization is any inpatient admission to a health care facility even if for less than 24 hours. Hospitalization or prolongation of a hospitalization constitutes a criterion for an AE to be serious; however, it is not in itself considered an SAE. In the absence of an AE, a hospitalization or prolongation of a hospitalization should not be reported as an SAE. For example, the following hospitalizations would not require expedited reporting for an SAE:

  – a hospitalization or prolongation of hospitalization needed for a procedure required by the protocol or as part of another routine procedure; or
  – a hospitalization for a pre-existing condition that has not worsened.

- Results in persistent or significant incapacity or substantial disruption of the ability to conduct normal life functions

  This is not intended to include experiences of relatively minor medical significance such as uncomplicated headache, nausea, vomiting, diarrhea, influenza, and accidental trauma (e.g., sprained ankle) which may interfere or prevent everyday life functions but do not constitute a substantial disruption.

- Is a congenital anomaly/birth defect
- Also, appropriate medical judgment should be exercised in deciding whether SAE reporting is required in other situations, such as important medical events that may not result in death, be life-threatening, or require hospitalization, but may jeopardize the patient and may require medical or surgical intervention to prevent one of the other outcomes listed in the definition of an SAE (Section 10.3). Examples of such events are intensive treatment in an emergency room or at home for allergic bronchospasm, blood dyscrasias or convulsions that do not result in inpatient hospitalization, or development of drug dependency or drug abuse.

10.4 Events requiring expedited reporting

All events listed in Section 10.4 must be reported in an expedited manner according to the instructions in Section 10.7.

10.4.1 *SAEs*

All events meeting the definition of an SAE (Section 10.3) require expedited reporting.

10.4.2 *Other events requiring expedited reporting*

Other events that must be recorded, reported, and followed up as indicated for an SAE (see Sections 10.3 and 10.7 for reporting procedures). This includes the following events:

- Pregnancy exposure to study therapy (If a pregnancy is confirmed, use of study therapy must be discontinued immediately. See Section 10.5.)

- Lactation exposure to study therapy, with or without an AE
- Inadvertent or accidental exposure to study therapy, with or without an AE
- Medication errors involving study therapy with an AE, including product confusion and potential product confusion. (A medication error is any preventable event that may cause or lead to inappropriate use or harm while the study therapy is in control of the healthcare professional or patient. Examples of reportable medication error include administration of unassigned treatment and administration of expired study therapy, when associated with an AE/SAE.)
- Death, excluding death due to progression of breast cancer
- Potential Hy's Law cases (see Sections 10.4.4 and 10.7.3)

10.4.3 *Adverse events of special interest*

Adverse events of special interest (AESI) listed below are required to be reported by expedited reporting by the investigator (i.e., no more than 24 hours after learning of the event; see Section 10.7 for reporting instructions). Some of the events listed below are not considered AESIs unless they meet the severity grade criteria. When the event does not meet the severity grade criteria, the event will be reported routinely as an AE on the eCRFs unless the event meets the definition of an SAE.

Patients remain at risk for AESIs for an extended period following discontinuation of atezolizumab/placebo, so monitoring and reporting for AESIs should continue in the follow-up period. Refer to Section 10.7.2 for reporting requirements.

Adverse events of special interest for this study include the following:

- Cases of potential drug-induced liver injury that include an elevated ALT or AST in combination with either an elevated bilirubin or clinical jaundice, as defined by Hy's Law (see Section 8.20) and based on the following observations:
  - Treatment-emergent ALT or AST > 3 x baseline value in combination with total bilirubin > 2 x ULN (of which ≥ 35% is direct bilirubin)
  - Treatment-emergent ALT or AST > 3 x baseline value in combination with clinical jaundice
- Suspected transmission of an infectious agent by the study treatment (STIAMP), defined as – Any organism, virus, or infectious particle (e.g. prion protein transmitting transmissible spongiform encephalopathy), pathogenic or non-pathogenic, is considered an infectious agent. A transmission of an infectious agent may be suspected from clinical symptoms or laboratory findings that indicate an infection in a patient exposed to a medicinal product. This term applies only when a contamination of study treatment is suspected.
- Pneumonitis ≥ Grade 2
- Colitis - Grade 2 requiring steroids or Grade 3 or Grade 4 colitis
- Hyperglycemia ≥ Grade 3
- Pancreatitis - Grade 2 requiring steroids or Grade 3 or 4 pancreatitis
- Adrenal insufficiency, hyperthyroidism, and hypophysitis ≥ Grade 2
- AST, ALT, total bilirubin increase (Hepatitis) - Grade 2 requiring steroids or ≥ Grade 3 AST, ALT, total bilirubin increase
- Systemic lupus erythematosus

- Neurological disorders: Guillain-Barré syndrome, myasthenic syndrome or myasthenia gravis, and meningoencephalitis, myelitis, facial paresis
- Hypersensitivity/infusion-related reactions ≥ Grade 3, cytokine release syndrome ≥ Grade 3
- Influenza-like illness ≥ Grade 3
- Systemic inflammatory response syndrome
- Systemic immune activation
- Nephritis - ≥ Grade 2
- Uveitis
- Retinitis
- Optic neuritis
- Myositis ≥ Grade 2
- Myopathies, including rhabdomyolysis ≥ Grade 2
- Severe cutaneous reactions (e.g., Stevens-Johnson syndrome, bullous dermatitis, toxic epidermal necrolysis)
- Vasculitis
- Autoimmune hemolytic anemia
- Atrioventricular block complete ≥ Grade 2
- Mobitz (type) II atrioventricular block ≥ Grade 2
- Right ventricular dysfunction ≥ Grade 2
- Acute coronary syndrome ≥ Grade 2
- Heart failure ≥ Grade 2
- Left ventricular dysfunction ≥ Grade 3
- Myocardial dysfunction ≥ Grade 3
- Myocarditis or pericarditis ≥ Grade 2

10.4.4 *Clinical laboratory abnormalities*

- Abnormal laboratory findings (e.g., clinical chemistry and hematology) or other abnormal assessments (e.g., x-rays and scans) will be recorded as AEs or SAEs if they meet the definition of an AE or SAE, as defined in Section 10.2 and Section 10.3, respectively, and if the abnormality meets reporting requirements as described in Section 10.2.

- Special reporting requirements related to Hy's Law: All cases confirmed on repeat testing as meeting one of the criteria described in Section 8.24.2 with no other cause for LFT abnormalities identified at the time should be considered potential Hy's Law cases regardless of availability of all the results of the investigations performed to determine etiology of the abnormal LFTs. Such potential Hy's Law cases should be reported as serious adverse events (see Section 10.7.3).

10.4.5 *Disease-related events and/or disease-related outcomes not qualifying as SAEs*

Any event that is part of the natural course of breast cancer does not need to be reported as an SAE. Recurrence of breast cancer, second breast primary cancer, and second primary cancers unrelated to study therapy will be reported on the appropriate page of the eCRF.

Note: Any occurrence of a secondary malignancy (leukemia secondary to oncology chemotherapy [AML]; myelodysplastic syndrome [MDS]; and/or treatment related *secondary* malignancy is to be reported as an SAE.

10.5 Pregnancy

- If a patient becomes pregnant while receiving study therapy (discontinue study therapy) or within 5 months after the last dose of atezolizumab/placebo, NSABP North American sites must report the pregnancy to ProPharma and GBG sites must submit to MedCODES. ***The investigator will record pregnancy information on the NSABP B-59/GBG 96-GeparDouze Pregnancy eCRF and complete within 2 weeks of learning of a patient's pregnancy.***
- Information about the use of study therapy in pregnancy encompasses the entire course of pregnancy and delivery, and perinatal and neonatal outcomes even if there were no abnormal findings. Information on the status of the mother and child will be provided to ProPharma by NSABP North American sites and to GBG Projectmanagement by GBG sites. Generally, follow-up will be no longer than 6 to 8 weeks following the estimated delivery date. Any premature termination of the pregnancy will also be reported.
- Should a female partner become pregnant by a male patient participating in the study, the investigator must be notified immediately. (See separate consent for release of pregnancy outcome information.) If the female partner signs consent, the outcome of the pregnancy will be reported.
- Any pregnancy complication or elective termination of a pregnancy for medical reasons will be recorded as an AE or SAE. A spontaneous abortion is always considered to be an SAE and will be reported as such.
- Any SAE occurring in association with pregnancy brought to the investigator's attention after the patient has completed the study and considered by the investigator as possibly related to study therapy, must be reported on the eCRF.

10.6 Grading the severity of the AE

The NCI CTCAE v4.0 must be used to determine the grade of the AE. The CTCAE provides descriptive terminology and a grading scale for each AE listed. Information regarding the CTCAE can be found on the CTEP Web site at http://ctep.cancer.gov. If you need further assistance, contact CCD (see Information Resources).

10.7 Expedited reporting instructions

10.7.1 *Time period for reporting SAEs requiring expedited reporting*

- All SAEs as noted in Section 10.3 regardless of relationship to study therapy begins with the first study treatment and will be reported in an expedited manner as described in Section 10.7.3. Reporting SAEs (and other applicable events) regardless of relationship to study therapy begins with the first study treatment and continues until 30 days after the last dose of study therapy.
- Any SAE assessed as related to study participation (e.g., protocol-mandated procedures) will be recorded from the time of the first study treatment up to and including any follow-up contact.
- Following the AE assessment 30 days after the last dose of study therapy, only SAEs determined to be related to study therapy or participation will be reported in an expedited manner.
- The investigator must follow-up on all SAEs until the events have subsided, until values have returned to baseline, or until the condition has stabilized.

10.7.2 *Time period for reporting adverse events of special interest requiring expedited reporting*

Adverse events of special interest require expedited reporting regardless of relatedness to study drug from the time of the first study treatment, up to and including any follow-up contact, until study closure.

10.7.3 *Reporting instructions*

- All SAEs and other events requiring expedited reporting must be reported using a NSABP B-59/GBG 96-GeparDouze eCRF and completed within 24 hours of the study site personnel's initial notification of the event to [redacted]
- When reporting potential Hy's Law cases, the eCRF should include the following:
  - Seriousness Criteria = Important Medical Event
  - Narrative: Include the term "Potential Hy's Law case" in the narrative; the text should also detail what additional study results are available at the time of reporting and what other studies are planned or results pending to further investigate alternative causes of the abnormal ALT/AST or bilirubin that triggered the report. The timing of planned patient follow-up should also be noted.
- NSABP will forward expedited report forms and their supporting documentation for SAEs that meet reporting requirements to the FDA [redacted].
- Investigators are responsible for reporting AEs that meet criteria specific to their local IRBs.

10.8 Time period and frequency for routine reporting of AEs

- Patients will be monitored for the occurrence of AEs at each scheduled assessment and during any contact with the patient during the study.
- All AEs, including SAEs and other AEs that have been reported on NSABP B-59/GBG 96-GeparDouze on the SAE eCRF, regardless of relationship to study therapy, will be recorded on the AE eCRF with the first study treatment until 30 days after the last dose of study therapy.
- For routine reporting, **all non-laboratory ≥ Grade 1 AEs** will be reported on the AE eCRF. Note: Only clinically significant laboratory AEs must be reported on the AE eCRF (see Section 10.2).
- The investigator must follow up on all AEs until the events have subsided, until values have returned to baseline, or until the condition has stabilized.
- Following the AE assessment 30 days after the last dose of study therapy, routine reporting is no longer required. (See Section 10.7.1 for expedited reporting requirements.)

11.0 ASSESSMENT OF EFFECT

11.1 Evaluation of pCR

Evaluation of the surgical specimens following completion of neoadjuvant chemotherapy will be conducted and recorded on a pathology report by the local pathologist. Specimens will be processed uniformly as outlined in Appendix D. The local pathology reports will be submitted for a central review to determine if protocol criteria for pCR breast and nodes (primary endpoint) and other secondary endpoints related to pathologic response were met.

11.2 Disease progression on neoadjuvant therapy

During the neoadjuvant therapy, diagnosis of disease progression should be supported by clinical, laboratory, radiological, and/or histological findings. As a guide for determining progression, progression may be defined as an increase of breast tumor or axillary nodes in maximum diameter by 25%, if it is not considered to be a pseudoprogression. Progression during carboplatin and paclitaxel with atezolizumab/placebo resulting in the decision to end that segment and initiate AC or EC with atezolizumab/placebo will not be considered as progression meeting protocol criteria for an EFS event. Only progression resulting in administration of non-protocol cancer therapy or inoperability will meet protocol criteria for an EFS event.

12.0 DIAGNOSIS OF BREAST CANCER RECURRENCE

12.1 General instructions

- Documentation of a breast cancer recurrence requires meeting at least one of the criteria defined below. Suspicious findings do not provide adequate documentation of a breast cancer recurrence and should not be an indication to alter protocol therapy.
- Tumor marker evaluations alone do not document breast cancer recurrence.
- Treatment of a breast cancer recurrence or second primary cancer will be at the discretion of the investigator.

12.2 Local recurrence

Note: If the first local recurrence is non-invasive breast cancer, the first invasive breast cancer must also be reported.

Recurrent local tumor is defined as evidence of invasive breast cancer or DCIS in the ipsilateral breast or invasive breast cancer in the skin of the ipsilateral breast. Patients who develop clinical evidence of local recurrence in the ipsilateral breast must have a biopsy confirmation of recurrence. However, if a patient also meets criteria for regional or distant metastatic disease, results of clinical exams alone will be sufficient to document local recurrences.

12.2.1 *Ipsilateral breast tumor recurrence (IBTR)*

An IBTR event is defined as recurrent invasive breast cancer or DCIS in the ipsilateral breast parenchyma or invasive breast cancer in the skin of the breast occurring after lumpectomy.

Acceptable documentation includes core, incisional or excisional biopsy. Cytology alone will not be adequate to establish IBTR.

12.2.2 *Other local recurrence*

Defined as recurrence in the skin of the chest wall (exclusive of the breast) or chest wall.

Acceptable documentation includes core, incisional or excisional biopsy, as well as cytology.

12.3 Regional recurrence

Defined as the development of tumor in the ipsilateral internal mammary, ipsilateral supraclavicular, ipsilateral infraclavicular and/or ipsilateral axillary nodes, as well as the soft tissue of the ipsilateral axilla, following surgery. Recurrence must be confirmed by biopsy or cytology. However, if a patient meets criteria for distant metastatic disease, results of clinical exams alone will be sufficient to document regional recurrences.

12.4 Distant recurrence

Defined as evidence of tumor in all areas, with the exception of those described in Sections 12.2 and 12.3. **The first distant recurrence <u>and</u> the first central nervous system recurrence will be reported.** Further treatment for distant metastasis, with or without evidence of local-regional recurrence, will be at the discretion of the investigator.

12.4.1 ***Skin, subcutaneous tissue, and lymph nodes (other than local or regional)***

Acceptable documentation includes positive cytology, aspirate or biopsy, or radiologic evidence of metastatic disease.

12.4.2 ***Bone marrow***

Acceptable documentation includes positive cytology, aspirate, biopsy, or MRI scan.

12.4.3 ***Lung***

Acceptable documentation includes: (i) positive cytology, aspirate, or biopsy, or (ii) radiologic evidence of multiple pulmonary nodules that are judged to be consistent with pulmonary metastases.

Note: If a solitary lung lesion is found and no other lesions are present on lung tomograms, CT scan, or MRI scan, further investigations, such as biopsy, needle aspiration, PET-CT scan, or PET scan should be performed. Proof of neoplastic pleural effusion must be established by cytology or pleural biopsy.

12.4.4 ***Skeletal***

Acceptable documentation includes: (i) x-ray, CT, or MRI evidence of lytic or blastic lesions consistent with bone metastasis, (ii) biopsy proof of bone metastases, or (iii) bone scan, PET-CT scan, or PET scan clearly positive for bone metastases.

Note: If the diagnosis is equivocal by bone scan or radiologic evaluation, a biopsy is strongly recommended. A bone scan with uptake limited to joints or in a recent area of trauma (surgical or otherwise) cannot be used as a criterion for breast cancer recurrence.

12.4.5 ***Liver***

Acceptable documentation includes: (i) abdominal CT scan, liver scan, ultrasound, MRI, PET-CT scan, or PET scan consistent with liver metastases, or (ii) liver biopsy confirmation of the metastatic disease.

Note: If the radiologic findings are not definitive (especially with solitary liver nodules), a liver biopsy is recommended; however, if a biopsy is not performed, serial scans must be obtained to document stability or progression.

12.4.6 ***Central nervous system***

Acceptable documentation includes: (i) positive CT scan, PET-CT scan, PET scan, or MRI scan, usually in a patient with neurological symptoms, or (ii) biopsy or cytology (for a diagnosis of leptomeningeal involvement).

12.5 Contralateral breast cancer

Contralateral breast cancer is defined as evidence of invasive breast cancer or DCIS in the contralateral breast or chest wall. The diagnosis of a contralateral breast cancer must be confirmed by core, incisional, or excisional biopsy. Cytology alone will not be adequate to document a contralateral breast cancer.

12.6 Second primary cancer

Second primary cancer is defined as any ***invasive*** non-breast cancer other than squamous or basal cell carcinoma of the skin. The diagnosis of a second primary cancer must be confirmed histologically whenever possible.

13.0 PATIENT ENTRY PROCEDURES

13.1 Patient consent form

Before study entry, the consent form including any addenda must be signed and dated by the patient and the person obtaining informed consent. After written informed consent has been obtained, register the patient into the [redacted] The study site will obtain a unique patient number which will stay the same throughout the entire study covering all study periods. At this time point, the patient is enrolled into the study.

13.2 Study entry

As part of the NSABP B-59/GBG 96-GeparDouze consent form, patients have agreed to allow the submission of material to be submitted for central testing to determine ER, PgR, HER2, and PD-L1 status for final eligibility assessment and to a baseline research biopsy. Material from either the diagnostic core biopsy or the research biopsy can be used for central testing. If local testing has determined a tumor to be HER2 equivocal or to have a borderline ER/PgR status (% IHC staining < 10% for both) and other eligibility criteria are met, material may be submitted for central testing to determine eligibility. Patients have also agreed to submission of material from residual tumor if present at the time of surgery. Patients may agree to have a research biopsy procedure to procure tumor tissue prior to receiving the second cycle of atezolizumab/placebo (will be performed on the first 500 patients who agree to the optional biopsy).

13.2.1 *Pretreatment core biopsy specimen collection for research*

Two core biopsy specimens from the breast primary collected for research before initiation of study therapy is required.

Refer to the NSABP B-59/GBG 96-GeparDouze Pathology and Correlative Science Instructions for detailed information regarding the NSABP B-59/GBG 96-GeparDouze pathology requirements and shipment.

13.2.2 *Randomization*

Once eligibility has been established, the patient may be randomized, and the site will obtain the patient's treatment assignment (Arm A or Arm B). Treatment assignments will be blinded. Patients enrolled but not randomized for any reason have to be registered as a Screening Failure in the [redacted]

13.3 Treatment assignment

After all the entry eCRFs have been completed, the institution will receive the patient's randomization information via [redacted]

13.4 Regulatory requirements for Canadian institutions

Any Canadian institution that will utilize PET scans in the conduct of this study *must* indicate the intent to do so to the NSABP Department of Regulatory Affairs *prior to initiating patient enrollment at the institution (and any satellites)*. Use of PET scans as part of this study may require submission by the NSABP of specific documentation to Health Canada before activation of the trial at the institution.

13.5 Requirements for discontinuation of study therapy

In addition to the specific toxicities and toxicity grades outlined in the dose modification sections of the protocol, study therapy must be discontinued if one or more of the following occur:

- the patient develops intolerable toxicity related to study medications that cannot be controlled with other medications;
- any medical condition that may jeopardize the patient's safety if study drug administration continues;
- pregnancy;
- disease recurrence or progression resulting in a decision to administer non-protocol cancer therapy;
- the patient is unable to meet the study requirements; or
- new information about the study drugs or other treatments for breast cancer becomes available.

If study therapy is stopped, study data and other materials should be submitted according to the study schedule unless the patient withdraws from the study.

13.6 Patient-initiated discontinuation of study therapy

Even after a patient agrees to take part in this study, the patient may stop study therapy at any time. If study therapy is stopped but the patient still allows the study doctor to submit information, study data, and other materials according to the study schedule, such agreement should be documented in the patient's medical record.

13.7 Patient-initiated withdrawal from the study

If a patient chooses to have no further interaction regarding the study, the investigator must provide written documentation of the patient’s decision to fully withdraw from the study. Any data collected up to the time of withdrawal from the study will continue to be used.

14.0 DATA HANDLING AND RECORDKEEPING

14.1 Instruction for completion of NSABP B-59/GBG 96-GeparDouze forms and materials

Refer to the "NSABP B-59/GBG 96-GeparDouze eCRF Completion Guidelines" for detailed instructions regarding data collection, AE reporting, and electronic case report form completion.

The EDC system databases will be maintained by IDDI for the NSABP sites and by GBG for the GBG sites. Periodically throughout the duration of the trial and at the completion of the trial, the IDDI and GBG databases will be merged to facilitate analysis of the complete trial dataset.

15.0 STATISTICAL CONSIDERATIONS

15.1 Study design

NSABP B-59/GBG 96-GeparDouze is a prospective, randomized, double-blind, phase III clinical trial. Patients are randomized 1:1 to placebo or the experimental agent, atezolizumab.

15.2 Primary aim and endpoint

Aim: To determine event-free survival (EFS)

Endpoint: EFS is defined as time from randomization until event: EFS events are progression on protocol therapy resulting in administration of non-protocol cancer therapy or inoperability, local invasive recurrence following mastectomy, local invasive recurrence in the ipsilateral breast following lumpectomy, regional recurrence, distant recurrence, contralateral invasive breast cancer, second non-breast primary cancer (excluding squamous or basal cell carcinoma of the skin), or death from any cause prior to recurrence or second primary cancer. On July 26, 2021, FDA approved the use of pembrolizumab for high-risk early stage TNBC. Some US patients who are still on the neoadjuvant period of the study may opt to stop the protocol therapy without disease progression and take pembrolizumab instead. These patients will NOT be considered to have experienced EFS events.

15.3 Secondary endpoints

The secondary endpoints of the study are:

- Overall survival (OS), defined as time from randomization until death from any cause.
- Pathologic complete response in the breast and post-therapy lymph nodes evaluated histologically (pCR breast and nodes [ypT0/Tis ypN0]) defined as the absence of any invasive component in the resected breast specimen and all resected lymph nodes following completion of neoadjuvant therapy.
- Distant disease-free survival (DDFS), defined as time from randomization until distant recurrence, death from breast cancer, death from other causes, and second primary invasive cancer (non-breast).
- Disease-free survival (DFS), defined as time from the first breast surgical procedure (operation) to the first occurrence of disease recurrence or death from any cause. Events defining DFS are ipsilateral invasive breast tumor recurrence, ipsilateral local-regional invasive breast cancer recurrence, distant recurrence, contralateral invasive breast cancer, ipsilateral or contralateral DCIS, second primary non-breast invasive cancer (with the exception of non-melanoma skin cancers and non-breast in situ carcinoma of any site) and death attributable to any cause including breast cancer, non-breast cancer, or unknown cause.
- Frequencies of adverse events graded according to the NCI Common Terminology Criteria for Adverse Events version 4.0 (CTCAE v4.0).
- Frequency of elevated levels of troponin-T and changes in LVEF levels among the cardiac safety lead-in study patients.

15.4 Exploratory endpoints

- Pathologic complete response in the breast (ypT0/Tis), defined as the absence of any invasive component in the resected breast specimen (nodal material not considered).

- Pathologic complete response in the breast and lymph nodes (ypT0 ypN0), defined as the absence of any invasive component or DCIS in the resected breast specimen and all resected lymph nodes following completion of neoadjuvant therapy.
- Positive nodal status conversion rate, defined as the percentage of patients clinically node-positive that convert to pathologically node-negative following completion of neoadjuvant chemotherapy.
- Recurrence-free interval (RFI), defined as time from randomization until invasive local, regional, or distant recurrence, or death from breast cancer (censored for death from other causes).
- Brain metastases free survival, defined as time from randomization until documentation of first event brain metastasis or death from any cause. Patients who develop locoregional or distant recurrence outside of the central nervous system as first event will continue to be followed for subsequent development of brain metastases.
- Event-free survival (EFS) in patients with deleterious germline *BRCA* mutation status.
- Correlative science

15.5 Stratification and randomization

Eligible triple-negative breast cancer patients will be randomized to receive either paclitaxel/carboplatin/placebo followed by doxorubicin/cyclophosphamide/placebo or epirubicin/cyclophosphamide/placebo or paclitaxel/carboplatin/atezolizumab followed by doxorubicin/cyclophosphamide/atezolizumab or epirubicin/cyclophosphamide/atezolizumab. Randomization will be accomplished using stratified permuted blocks. The stratification factors include: 1) Group (NSABP, Foundation, Inc.; GBG); 2) nodal status as determined by protocol-specified criteria (negative; positive); 3) AC/EC (every 2 weeks; every 3 weeks); 4) clinical size of the primary tumor (1.1-3.0 cm; > 3.0 cm); and 5) PD-L1 status (positive; negative, indeterminate, or not available). It is noted that the PD-L1 status of patients was not available at randomization for patients enrolled prior to the amendments in July 2019.

15.6 Patient cohorts used for analysis

15.6.1 *Intent-to-treat cohort*

The intent-to-treat (ITT) population (defined as all randomized patients) will be used for analysis of the primary and secondary endpoints except toxicity. Patients will be analyzed according to their randomly assigned treatment regardless of treatment actually received.

15.6.2 *Safety cohort*

The safety population includes all patients who receive at least one dose of study therapy and will be used for the toxicity endpoint. Patients will be analyzed according to treatment actually received, thus any patient receiving at least one dose of atezolizumab/placebo will comprise the experimental group.

15.7 Sample size determination and protocol duration for the primary endpoint of EFS

The sample size for this trial is based on the requirements for testing of the EFS endpoint. The hazard rates used for determination were taken from our experience in NSABP B-40 trial. The docetaxel followed by AC arm of NSABP B-40 was similar in efficacy to AC followed by weekly paclitaxel in ECOG 1199 (Sparano 2015). In NSABP B-40, the annual hazard rates were not constant and varied substantially over the first 4 to 5 years. Thus, instead of using a constant

hazard rate across time, yearly-specific hazards rates were used. These rates were then reduced by 20% to account for an anticipated effect of adding carboplatin to the treatment regimen based on CALGB 40603 (Sikov SABCS 2015). To achieve monthly precision in estimates of accrual and study duration, the calculation of sample size was based on monthly hazard rates converted from each of the yearly hazard rates described above. The resulting monthly hazard rates are 0.00460 during year 1, 0.01020 during year 2, 0.00440 during year 3, 0.00186 during year 4 and 0.00200 for year 5 and all subsequent years.

The original sample size was based on having sufficient statistical power to detect a hazard ratio of 0.70 at α=0.045 assuming a lost-to-follow-up rate of 0.00083 per month. A sample size of 760 patients per arm for a total sample size of 1,520 patients followed to obtain 269 EFS events would provide a test of hypothesis with 80% statistical power at an overall alpha level of 0.045. After allowing for a ramp-up of accrual in the first eighteen months to 40 patients per month and a further ramp-up to 90 patients per month by accrual month 28, it is anticipated that the accrual rate would peak at 105 patients per month by accrual month 32. With this accrual pattern, it would take about 34 months to accrue 1,520 patients. Follow-up of an additional 30 months after completion of accrual (total study time of 64 months) would be sufficient to obtain 269 EFS events.

Accrual to NSABP B-59/GBG 96-GeparDouze began in December 2017 and was completed in May 2021 with a total of 1550 patients randomized. Based on actual accrual and the decision to eliminate pCR as a co-primary endpoint, we recalculated the power to detect a hazard ratio of 0.70 attributed to the addition of atezolizumab, assuming a lost-to-follow-up rate of 0.00083 per month, using the actual accrual pattern for the power calculation. With 1550 patients accrued in 42 months, an additional 22 months follow up will allow us to obtain 252 events under the assumptions stated above, which will provide 80% power to detect a HR of 0.7 between the atezolizumab and the placebo arm at an overall 2-sided alpha level of 0.05.

Consensus guidelines (NCCN, ESMO, and St. Gallen) now include administration of adjuvant capecitabine as a standard of care option for patients with residual invasive cancer in the breast or axillary lymph nodes following administration of the neoadjuvant therapy on this protocol. The use of adjuvant capecitabine therapy across both treatment arms will be monitored blinded to treatment arm. Because the use of capecitabine may reduce the observed rate of EFS events as well as slightly increase the HR, an amendment to formally delay the final analysis in order to obtain enough EFS events will be considered based on the overall use of adjuvant capecitabine therapy observed on this study. The amendment would be submitted prior to any analysis of the primary endpoint.

15.8 Statistical analysis plans

15.8.1 *Primary analysis of EFS*

The comparison of EFS between the two treatment arms will be assessed using the stratified log-rank test incorporating the stratification factors identified in Section 15.5. Due to the potential risk of over-stratification (Schoenfeld 1987; Akazawa 1997), if at least one stratum has fewer than four EFS events at the interim analysis or the primary analysis, the stratification factor that contains the level with the smallest number of patients will be removed from the stratified analyses. If there still exists a stratum with fewer than four EFS events, a second stratification factor that contains the level with the second smallest number of patients will also be removed from the stratified analyses. If necessary, at most two stratification factors will be considered for removal to avoid losing statistical power while reducing over-stratification (Akazawa 1997). The same stratification factors will be applied to the main analyses of all endpoints where stratified

analyses are planned. The definitive analysis will be performed when 252 EFS events are recorded. It is estimated that this would occur about 64 months after accrual is initiated. There will be one interim analysis for EFS efficacy performed when we obtain 190 events, i.e., at 75.4% information time. It is estimated that this would occur about 55 months after accrual is initiated. To maintain the experiment-wise alpha level at 0.05, O'Brien Fleming spending will be used accounting for the proportion of the total number of expected EFS events observed at the interim analysis. At 75.4% information time (190 events), the interim analysis would be performed at an alpha level of 0.0197, and the final, definitive analysis would be performed at an alpha level of 0.0441.

The Kaplan-Meier estimates will also be calculated separately for patients under the different treatment regimens. Stratified Cox proportional hazards models will be used to assess differences in EFS when adjusting for potential confounders such as age at study entry, PD-L1 status, nodal status, *BRCA* mutation status, tumor size, and tumor grade. Potential interactions between treatment and clinical factors will also be assessed using Cox models. In the analysis of EFS and all time-to-event analysis, the follow-up time and event status of patients will be based on the follow-up time and event status obtained at the date of the last clinic visit/medical testing formally evaluating and documenting the patient's event status. This same rule would be used for those who have withdrawn from study participation or who have missed scheduled clinic visits/testing.

15.8.2 *Subgroup analysis of EFS*

To assess the generalizability of the observed treatment effect on EFS, subgroup analysis based on stratification factor as listed in Section 15.5 will be performed. Potential interaction between treatment and each factor will be explored by Cox proportional hazard models. HRs estimated from Cox proportional models and their corresponding 95% CIs will be calculated and presented by forest plots. Kaplan-Meier estimates of median survival time (if reached) will also be calculated.

15.8.3 *Sensitivity analysis of EFS in patients who entered the study before the end of July 2020*

US patients who entered the study within one year prior to the approval of pembrolizumab in the US for high-risk, early stage TNBC could potentially have the access to pembrolizumab while they are still on the study treatment. Some of them may opt to stop the study therapy and take pembrolizumab instead. A total of 149 patients belong to this group, which is less than 10% of the total accrual of the study. Nevertheless, to assess the effect of the approval of pembrolizumab in the US in July of 2021, we will perform a sensitivity analysis excluding the 149 US patients who entered the study since July 2020. Analysis described in Section 15.8.1 for EFS will be repeated in this subset of study population.

15.8.4 *Analysis of secondary efficacy endpoints*

Analysis of overall survival (OS)

If EFS reached a significant result at either interim analysis or final analysis, we will recycle the type I error and formally compare the OS between the two treatment arms at α=0.05. Stratified log-rank test will be used as the primary analysis to compare the OS between the two study arms in the similar manner as described in Section 15.8.1 for the analysis of EFS, and a 2-sided alpha level at 0.05 will be used for claiming statistical

significance. To control the overall type I error at 0.05, EFS and OS will be tested following a hierarchical fixed sequence testing procedure: if the test of EFS is not statistically significant, OS will not be formally tested, and we will not claim statistical significance for OS.

The final analysis of OS will be performed by November of 2027 or when 300 deaths have been observed, whichever happens first. Three hundred (300) deaths will provide 70% power to detect a HR of 0.75 (2-sided α=0.05). We estimated the annual hazard rates for the placebo arm of NSABP B-40, which were further reduced by 30% to account for the anticipated efficacy of adding carboplatin to the treatment (ClinicalTrials.gov 2000). The monthly hazard rate is estimated to be 0.0017 for year 1, 0.0035 for year 2, 0.0054 for year 3, 0.0033 for year 4 and 0.0021 for year 5 and all subsequent years. Using the actual accrual pattern and the above assumptions, we estimated that we will reach 300 deaths by November 2027, 10 years after the initiation of the trial. One planned interim analysis for OS will be performed when the EFS endpoint crosses the boundary (either at interim or final analysis of EFS). To maintain the experiment-wise alpha level at 0.05, O'Brien Fleming spending will be used accounting for the proportion of the total number of expected deaths observed at the interim analysis.

Analysis of pCR breast and nodes (ypT0/Tis ypN0)

The difference between treatment groups in the proportion of patients with pCR will be assessed using the Cochran-Mantel-Haenszel test. In the analysis of pCR, patients who do not have surgery, have withdrawn from study participation before reaching surgery or have a missing value for tumor response for any reason will be considered as a failure (non-pCR).

Analysis of distant disease-free survival (DDFS) and disease-free survival (DFS)

DDFS and DFS will be compared between the two study arms in the same manner as described for the analysis of EFS for the ITT population in Section 15.8.1.

15.8.5 ***Toxicity***

Analysis of CTCAEs will be performed as part of the safety analyses. The distribution by treatment group of the highest grade of each CTCAEs experienced by each patient categorized will be tabulated and reviewed on a monthly basis by a medical review committee. The tabulations will also be reviewed on a semi-annual basis by the Data Safety Monitoring Board (DSMB). These tabulations will include summaries by system organ class and summaries by term under each system organ class. While the tabulated distributions will include all grades, the primary focus will be the comparison of Grade 3, 4, and 5 events, particularly noting those events which are unexpected from the treatments being provided.

15.8.6 ***Cardiac Safety Lead-in Study***

The first 60 patients randomized will be included in the cardiac safety lead-in study involving the collection of troponin-T levels and one extra LVEF assessment. Data from this population will be included in an assessment of these parameters. The proportion of patients experiencing elevated troponin-T levels measured at baseline and after each of the 1st and 3rd cycles of AC/EC will be determined and assessed to evaluate differences by treatment group. For LVEF assessment, change from baseline to before the initiation

of the 3rd cycle of AC/EC and after surgery will also be determined and assessed for differences by treatment group. Two reports of findings on these parameters will be prepared and provided for review by the DSMB for their assessment of any unexpected cardiac toxicity signal. One report will be prepared when the troponin-T and LVEF data have been received for the first 20 patients in the cardiac safety lead-in study, and a final report prepared when the data for all 60 patients have been received. Potential emerging safety signals or issues identified during the cardiac safety lead-in and subsequent safety reviews will be evaluated by the DSMB to determine any impact to the conduct of the clinical trial (e.g., enrollment hold, modification to safety monitoring, or implementation of risk minimization measures). The DSMB will also be provided a summary of all cardiac toxicities reported for these patients using CTCAE v4.0.

15.8.7 *Analysis of exploratory endpoints*

Binary endpoints will be carried out in the same manner as the analysis of ypT0/Tis ypN0 in Section 15.8.4. Time to event endpoints will be analyzed in the same manner as described in Section 15.8.1 for EFS.

15.9 Data Safety Monitoring Board (DSMB)

This study will use a DSMB whose members include therapeutic area experts and statisticians who are not employed by [redacted] NSABP, or GBG and who have no material conflict of interest. Members of the DSMB will be jointly selected by [redacted], NSABP, and GBG, and the DSMB will report their recommendations to the Executive Committee.

The DSMB will meet approximately every 6 months and will review ongoing study progress including safety and, when appropriate, efficacy data. DSMB monitoring will be unblinded. The DSMB will also review the data presented for the two reports of findings from the cardiac safety study and the findings from the protocol-defined interim analysis. The DSMB will have the ability to recommend suspension of enrollment if safety concerns are identified at any of the 6 monthly assessments. At the interim analysis, both efficacy and safety will be reviewed and trial recommendations will be based on the totality of the data. The DSMB can recommend changes to the trial, including stopping of the trial, if they have concerns over the benefit-risk profile for atezolizumab. If no cardiac safety concerns are identified by the DSMB among the cardiac safety lead-in population, the additional monitoring performed for the cardiac safety lead-in population will end and monitoring for the rest of the patient population will proceed as planned. If the DSMB review identifies a cardiac safety concern, additional cardiac safety monitoring may be instituted for all patients in the study as part of a protocol amendment.

Reports for the DSMB will be prepared by an independent statistician of IDDI not affiliated with NSABP B-59/GBG 96-GeparDouze and will be presented to the DSMB in closed session. Following review of data, the DSMB will make a recommendation regarding the future conduct of the trial to the Executive Committee. The DSMB recommendation will not contain any unblinded information or any information related to treatment outcomes of the trial unless the recommendation is to stop the trial early.

A separate DSMB charter will be developed defining the responsibilities and procedures for the DSMB.

16.0 PREMATURE TERMINATION OF THE STUDY

NSABP/GBG (the study sponsor) has the right to close this study which may be due but not limited to the following reasons:

- If risk-benefit ratio becomes unacceptable owing to, for example:
  - safety findings from this study (e.g., SAEs);
  - results of any interim analysis; or
  - results of parallel clinical studies.
- If the study conduct (e.g., recruitment rate; drop-out rate; data quality; protocol compliance) does not suggest a proper completion of the trial within a reasonable time frame.

The investigator has the right to close his/her center at any time.

For any of the above closures, the following applies:

- Closures should occur only after consultation between involved parties. Final decision on the closure must be in writing.
- All affected institutions (e.g., IRB(s); competent authority(ies); study center; head of study center) must be informed as applicable according to local law.
- The investigator will retain all other documents until notification is given for destruction.
- In case of a partial study closure, ongoing patients, including those in post-study follow-up, must be taken care of in an ethical manner.

Details for individual patient's withdrawal can be found in Section 13.0.

17.0 ETHICAL AND LEGAL CONDUCT OF THE STUDY

17.1 Study conduct

The study will be performed in accordance with ethical principles that have their origin in the Belmont Report and are consistent with the International Conference on Harmonization of Technical Requirements for Registration of Pharmaceuticals for Human Use (ICH)/Good Clinical Practice (GCP) as adopted by the United States Federal Food and Drug Administration ("FDA"), and applicable regulatory requirements. The study will also be carried out in keeping with applicable local law(s) and regulation(s).

For NSABP sites outside North America and GBG sites, this study will be conducted in full conformance with the ICH E6 guideline for Good Clinical Practice and the principles of the Declaration of Helsinki, or the laws and regulations of the country in which the research is conducted, whichever affords the greater protection to the individual. The study will comply with the requirements of the ICH E2A guideline (Clinical Safety Data Management: Definitions and Standards for Expedited Reporting). Studies conducted in the EU/EEA will comply with the EU Clinical Trial Directive (2001/20/EC).

Documented approval from appropriate IRB/Ethic Committee(s) will be obtained for each participating center before participation in the study, according to GCP, local laws, and regulations. When necessary, an extension, amendment, or renewal of the IRB/Ethic Committee(s) approval must be obtained and also forwarded to NSABP.

Strict adherence to all specifications in this protocol is required for all aspects of study con duct; the investigator may not modify or alter the procedures described in this protocol.

Details on discontinuation of the entire study or parts thereof can be found in Section 15.0.

17.2 Patient information and consent

A sample informed consent form (ICF) is provided as a document separate to this protocol. The investigator or designee will explain all relevant aspects of the study to each patient prior to his/ her entry into the study (i.e., before any study-related procedures are performed). Each patient/legal representative (not applicable for Europe) will have ample time and opportunity to ask questions and will be informed about the right to withdraw from the study at any time without any disadvantage and without having to provide reasons for this decision.

Only if the patient/legal representative voluntarily agrees to sign the ICF and has done so, may the patient enter the study. Additionally, the person who conducted the informed consent discussion will personally sign and date the form. The patient/legal representative or proxy consenter will receive a copy of the signed and dated form.

The signed ICF is to remain in the investigator site file or, if locally required, in the patient's note/file of the medical institution. In the event that informed consent is obtained on the date that baseline study procedures are performed, the study record or patient's clinical record must clearly show that informed consent was obtained prior to these procedures.

The ICF and any other written information provided to patients will be revised whenever important new information becomes available that may be relevant to the patient's consent, or there is an amendment to the protocol that necessitates a change to the content of the patient information and/or the written ICF. The investigator will inform the patient/legal representative of changes in a timely manner and will ask the patient to confirm his/her participation in the study by signing the revised ICF. Any revised written informed consent form and written information must receive the IRB/REB's approval/favorable opinion in advance of use.

17.3 **Record retention**

Copies of all essential documents as defined in ICH GCP Section 8 will be retained by the investigator according to the requirements of the regulatory authority.

17.4 **Confidential follow-up**

The investigator will be responsible for retaining sufficient information about each patient (e.g., name, address, phone number, identity number, and identity in the study) so that regulatory agencies, NSABP, GBG, and [redacted] may access this information should the need to do so arise. These records should be retained in a confidential manner for as long as legally mandated according to local requirements.

17.5 **Study agreements**

Each participating center will enter into an agreement obligating the Principal Investigator at each site to comply with all applicable terms, conditions, and obligations of the Clinical Site Study Agreement (CSSA), or equivalent, for this study. In the event of any inconsistency between this Clinical Study Protocol and the CSSA, the terms of Clinical Study Protocol shall prevail with respect to the conduct of the study and the treatment of patients and in all other respects, not relating to study conduct or treatment of patients, the terms of the CSSA shall prevail. Agreements should be in place before any study-related procedures can take place, or patients are enrolled.

18.0 STUDY MANAGEMENT

18.1 Pre-study activities

Qualification assessments are performed by NSABP or its authorized representatives. NSABP allocates the approved protocol to its Research Sites selected to participate which are in good standing. Those sites which are confident that they have significant investigator interest and available patients proceed by seeking local (or central) Institutional Review Board or Ethics Board approval. Upon receipt of the approval, a database of site IRB/REB approvals is maintained through NSABP. Patient entry cannot proceed unless an up-to-date IRB/REB approval is in this database and an activation notice has been received. Some obligations of NSABP may be assigned to a third-party organization.

18.2 Training of study site personnel

The Principal Investigator will ensure that all persons assisting with the conduct of the trial are adequately informed about the protocol, the investigational product, and their protocol-related duties and functions. In addition, any new information relevant to the performance of this study will be forwarded to the staff involved. The Principal Investigator will maintain a record of all individuals involved in the study (medical, nursing, and other staff) and their related training.

18.3 Monitoring of the study

During the course of the study, a third party will conduct remote and on-site monitoring visits according to the final approved monitoring plan in compliance with GCP requirements.

18.4 Auditing and inspection

To ensure compliance with GCP and governing regulatory agency requirements, arrangements may be made to conduct an audit or inspection to assess the performance of the study at the study site and to assess the study documents originating at the study site. These audits/inspections will be scheduled and performed according to the agreed upon audit plan.

18.5 Source data

Refer to the Clinical Site Study Agreement (CSSA) for location of source data.

18.6 Archiving of study documents

The Investigator will follow the principles outlined in the CSSA.

18.7 Data management

Data management will be performed by NSABP and by GBG. NSABP/GBG will provide the investigator site with a web base electronic data capture (EDC) system that is fully validated and conforms to 21 CFR Part 11 requirements. Investigator site staff will not be given access to EDC system until they have been trained on the EDC system.

Adverse events and medical history will be classified according to the terminology of CTCAE v4.0. Concomitant medications used to manage AESIs entered into the database will be coded using the World Health Organization (WHO) Drug Dictionary.

Data queries will be raised for inconsistent, implausible or missing data which will allow for modification or verification of the entered data by the investigator staff. All entries to the study database will be available in an audit trail. The data will be validated as defined in the Data

Management Plan. Quality control procedures will be applied to each stage of data handling to ensure that all data are reliable and have been processed correctly.

When all data have been declared to be cleaned, validated and coded, the database will be locked and the treatment codes will be unblinded to be made available for data analysis.

19.0 PUBLICATION INFORMATION

The publication or citation of study results will be made in accordance with the publication rules established by NSABP and the GBG that are in effect at the time the information is to be made publicly available.

20.0 REFERENCES

Adams S, Goldstein LJ, Sparano JA, et al. Tumor infiltrating lymphocytes (TILs) improve prognosis in patients with triple negative breast cancer (TNBC). Oncoimmunology 2015; 4(9): e985930. eCollection 2015 Sep.

Adashek ML and Feldman M. Cytokine release syndrome resulting from anti-programmed death-1 antibody: raising awareness among community oncologists. J Oncol Practice 2019; 15(19):502-504.

Akazawa K, Nakamura T, Palesch Y. Power of logrank test and Cox regression model in clinical trials with heterogeneous samples. Stat Med 1997; 16(5):583-597.

[redacted]

Bear H, Anderson S, Brown A, et al. The effect of tumor response of adding sequential preoperative docetaxel to preoperative doxorubicin and cyclophosphamide: Preliminary results from National Surgical Adjuvant Breast and Bowel Project protocol B-27. J Clin Oncol 2003; 21:4165-4174.

Bear H, Anderson S, Smith R, et al. Sequential preoperative or postoperative docetaxel added to preoperative doxorubicin plus cyclophosphamide for operable breast cancer: National Surgical Adjuvant Breast and Bowel Project protocol B-27. J Clin Oncol 2006; 24:2019-2027.

Bear HD, Tang G, Rastogi P, et al. Bevacizumab added to neoadjuvant chemotherapy for breast cancer. N Engl J Med 2012; 366(4):310-320.

Bear HD, Tang G, Rastogi P, et al. Neoadjuvant plus adjuvant bevacizumab in early breast cancer (NSABP B-40 [NRG Oncology]): secondary outcomes of a phase 3, randomised controlled trial. Lancet Oncol 2015; 16(9):1037-1048.

Bidard FC, Weigelt B, Reis-Filho JS. Going with the flow: From circulating tumor cells to DNA. Sci Transl Med 2013; 5(207):207ps214.

Blum JL, Flynn PJ, Yothers G, et al. Anthracyclines in early breast cancer: The ABC Trials-USOR 06-090, NSABP B-46-I/USOR 07132, and NSABP B-49 (NRG Oncology). J Clin Oncol 2017; 35(23):2647-2655.

Bonadonna G, Veronesi U, Brambilla C, et al. Primary chemotherapy to avoid mastectomy in tumors with diameters of three centimeters or more. J Natl Cancer Inst 1990; 82:1539-1545.

Bonadonna G, Valagussa P, Brambilla C, et al. Primary chemotherapy in operable breast cancer: Eight-year experience at the Milan Cancer Institute. J Clin Oncol 1998; 16:93-100.

Brahmer JR, Lacchetti C, Schneider BJ, et al. Management of immune-related adverse events in patients treated with immune checkpoint inhibitor therapy: American Society of Clinical Oncology Clinical Practice Guideline. J Clin Oncol 2018; 36(17):1714-1768.

Burstein HJ, Curigliano G, Loibl S, et al. Estimating the benefits of therapy for early-stage breast cancer: the St. Gallen International Consensus Guidelines for the primary therapy of early breast cancer 2019. Ann Oncol 2019; 30(10):1541-1557.

Cardoso F, Kyriakides S, Ohno S, et al. Early breast cancer: ESMO clinical practice guidelines for diagnosis, treatment and follow-up. Ann Oncol 2019; 30(10):1194-1220

Carey LA, Dees EC, Sawyer L, et al. The triple negative paradox: primary tumor chemosensitivity of breast cancer subtypes. Clin Cancer Res 2007; 13(8):2329-2334.

ClinicalTrials.gov [Internet]. Bethesda (MD): U.S. National Library of Medicine. 2000. Available from: https://clinicaltrials.gov/ct2/show/study/NCT00861705 ClinicalTrials.gov Identifier: NCT00861705.

Cortazar P, Zhang L, Untch M, Mehta K, et al. Pathological complete response and long-term clinical benefit in breast cancer: the CTNeoBC pooled analysis. Lancet 2014; 384(9938):164-172.

Dawson SJ, Tsui DW, Murtaza M et al. Analysis of circulating tumor DNA to monitor metastatic breast cancer. N Engl J Med 2013; 368:1199-1209.

De Mattos-Arruda L, Cortes J, Santarpia L, et al. Circulating tumour cells and cell-free DNA as tools for managing breast cancer. Nat Rev Clin Oncol 2013; 10(7):377-389.

Demaria S, Volm MD, Shapiro RL, et al. Development of tumor-infiltrating lymphocytes in breast cancer after neoadjuvant paclitaxel chemotherapy. Clin Cancer Res 2001; 7(10):3025-3030.

Denduluri N, Chavez-MacGregor M, Telli ML, et al. Selection of optimal adjuvant chemotherapy and targeted therapy for early breast cancer: ASCO Clinical Practice Guideline Focused Update. J Clin Oncol 2018; 36(23):2433-2443.

Deng R, Bumbaca D, Pastuskovas CV, et al. Preclinical pharmacokinetics, pharmacodynamics, tissue distribution, and tumor penetration of anti-PD-L1 monoclonal antibody, an immune checkpoint inhibitor. MAbs 2016; 8:593-603.

Denkert C, von Minckwitz G, Brase JC, et al. Tumor-infiltrating lymphocytes and response to neoadjuvant chemotherapy with or without carboplatin in human epidermal growth factor receptor 2-positive and triple-negative primary breast cancers. J Clin Oncol 2015; 33(9):983-991.

Dent R, Trudeau M, Pritchard KI, et al. Triple-negative breast cancer: clinical features and patterns of recurrence. Clin Cancer Res 2007; 13(15 Pt 1):4429-4434.

Dieci MV, Criscitiello C, Goubar A, et al. Prognostic value of tumor-infiltrating lymphocytes on residual disease after primary chemotherapy for triple-negative breast cancer: a retrospective multicenter study. Ann Oncol 2014; 25(3):611-618.

Emens LA, Braiteh FS, Cassier P, et al. Inhibition of PD-L1 by MPDL3280A leads to clinical activity in patients with metastatic triple-negative breast cancer (TNBC). Cancer Res 2015; 75(15 Suppl):Abstr 2859.

FDA-Guidance. Drug-Induced Liver Injury: Premarketing Clinical Evaluation; July 2009. (http:www.fda/gov/downloads/Drugs/GuidanceComplianceRegulatoryInformation/Guidances/UCM174090.pdf12009).

Fehrenbacher L, Spira A, Ballinger M, et al. Atezolizumab versus docetaxel for patients with previously treated non-small-cell lung cancer (POPLAR): a multicentre, open-label, phase 2 randomised controlled trial. Lancet 2016; 387:1837-1846.

Fisher B, Brown A, Mamounas E, et al. Effect of preoperative chemotherapy on local-regional disease in women with operable breast cancer: Findings from National Surgical Adjuvant Breast and Bowel Project B-18. J Clin Oncol 1997; 15:2483-2493.

Fisher B, Bryant J, Wolmark N, et al. Effect of preoperative chemotherapy on the outcome of women with operable breast cancer. J Clin Oncol 1998; 16:2672-2685.

[redacted] Personal communication, 2018.

Haanen JBAG, Carbonnel F, Robert C, et al. Management of toxicities from immunotherapy: ESMO Clinical Practice Guidelines for diagnosis, treatment and follow-up. Ann Oncol 2017; 28(suppl_4):iv119-iv142.

Hahnen E et al. Manuscript accepted for publication. JAMA Oncol 2017.

Hortobagyi GN, Blumenschein GR, Spanos W, et al. Multimodal treatment of locoregionally advanced breast cancer. Cancer 1983; 51:763-768.

Hortobagyi GN, Ames FC, Buzdar AU, et al. Management of stage III primary breast cancer with primary chemotherapy, surgery, and radiation therapy. Cancer 1988; 62:2507-2516.

La Rosée P. Treatment of hemophagocytic lymphohistiocytosis in adults. Hematology Am Soc Hematol Educ Protram 2015; 1:190-196.

La Rosée P, Horne A, Hines M, et al. Recommendations for the management of hemophagocytic lymphohistiocytosis in adults. Blood 2019; 133(23):2465-2477.

Lee DW, Santomasso BD, Locke FL, et al. ASTCT Consensus Grading for Cytokine Release Syndrome and Neurologic Toxicity Associated with Immune Effector Cells. Biol Blood Marrow Transplant 2019; 25(4):625-638.

Liedtke C, Mazouni C, Hess KR, et al. Response to neoadjuvant therapy and long-term survival in patients with triple-negative breast cancer. J Clin Oncol 2008; 26(8):1275-1281.

Loi S, Sirtaine N, Piette F, et al. Prognostic and predictive value of tumor-infiltrating lymphocytes in a phase III randomized adjuvant breast cancer trial in node-positive breast cancer comparing the addition of docetaxel to doxorubicin with doxorubicin-based chemotherapy: BIG 02-98. J Clin Oncol 2013; 31(7):860-867.

Loibl S, Jackisch C, Schneeweiss A, et al. Dual HER2-blockade with pertuzumab and trastuzumab in HER2-positive early breast cancer: a subanalysis of data from the randomized phase III GeparSepto trial. Ann Oncol 2017; 28(3):497-504.

Loibl S, O'Shaughnessy J, Untch M, et al. Addition of the PARP inhibitor veliparib plus carboplatin or carboplatin alone to standard neoadjuvant chemotherapy in triple-negative breast cancer (BrighTNess): a randomized, phase 3 trial. Lancet Oncol 2018; 19(4):497-509.

Loibl S, Weber KE, Timms KM, et al. Survival analysis of carboplatin added to an anthracycline/taxane-based neoadjuvant chemotherapy and HRD score as predictor of response-final results from GeparSixto. Ann Oncol 2018; 29(12):2341-2347.

Masuda N, Lee S-J, Ohtani S, et al. Adjuvant capecitabine for breast cancer after preoperative chemotherapy. N Engl J Med 2017; 376 (22):2147-2159.

McClain, K.L and O. Eckstein. Clinical features and diagnosis of hemaphagocytic lymphohistiocytosis. Up to Date [resource on the Internet]. 2014 [updated 29 October 2018; cited: 17 May 2019]. Available from: https://www.uptodate.com/contents/clinical-features-and-diagnosis-of-hemophagocytic-lymphohistiocytosis.

Merad M, Martin JC. Pathological inflammation in patients with COVID-19: a key role for monocytes and macrophages. Nat Rev Immunol 2020; 20(6):355-362.

Mittendorf EA, Zhang H, Barrios CH, et al. Neoadjuvant atezolizumab in combination with sequential nab-paclitaxel and anthracycline-based chemotherapy versus placebo and chemotherapy in patients with early-stage triple-negative breast cancer (IMpassion031): a randomised, double-blind, phase 3 trial. Lancet 2020; 396(10257):1090-1100.

Nanda R, Liu MC, Yee D, et al. Pembrolizumab plus standard neoadjuvant therapy for high-risk breast cancer: Results from I-SPY 2 Trial. Presented at the American Society of Clinical Oncologists, Chicago, June 2017.

National Comprehensive Cancer Network: Breast Cancer. NCCN Clinical Practice Guidelines in Oncology 2019 (Version 3.2019). http://www.nccn.org.

NSABP Biostatistical Center. Personal communication, 2014.

Rastogi P, Anderson S, Bear H, et al. Preoperative chemotherapy: Updates of National Surgical Adjuvant Breast and Bowel Project Protocols B-18 and B-27. J Clin Oncol 2008; 26:778-785.

Ravelli A, Minoia F, Davì S, et al. Classification criteria for macrophage activation syndrome complicating systemic juvenile idiopathic arthritis: A European League Against Rheumatism/American College of Rheumatology/Paediatric Rheumatology International Trials Organisation Collaborative Initiative. Arthritis Rheumatol 2016; 68(3):566-576.

Riegler LL, Jones GP, and Lee DW. Current approaches in the grading and management of cytokine release syndrome after chimeric antigen receptor T-cell therapy. Ther Clin Risk Manag 2019; 15:323-335.

Rosenberg JE, Hoffman-Censits J, Powles T, et al. Atezolizumab in patients with locally advanced and metastatic urothelial carcinoma who have progressed following treatment with platinum-based chemotherapy: a single-arm, multicentre, phase 2 trial. Lancet 2016; 387:1909-1920.

Rotz SJ, Leino D, Szabo S, et al. Severe cytokine release syndrome in a patient receiving PD-1-directed therapy. Pediatr Blood Cancer 2017; 64:e26642.

Schmid P, Cruz C, Braitch FS, et al. Atezolizumab in metastatic TNBC (mTNBC): Long-term clinical outcomes and biomarker analyses. American Association for Cancer Research Annual Meeting, Wash DC 2017; 58:Abstr. 2986.

Schmid P, Adams S, Rugo HS, et al. Atezolizumab and nab-paclitaxel in advanced triple-negative breast cancer. N Engl J Med 2018; 379(22):2108-2121.

Schmid P, Rugo HS, Adams S, et al. Atezolizumab plus nab-paclitaxel as first-line treatment for unresectable, locally advanced or metastatic triple-negative breast cancer (IMpassion130): updated efficacy results from a randomised, double-blind, placebo-controlled, phase 3 trial. Lancet Oncol 2020; 21(1):44-59.

Schneider BJ, Naidoo J, Santomasso BD, et al. Management of immune-related adverse events in patients treated with immune checkpoint inhibitor therapy: ASCO Guideline Update. J Clin Oncol 2021; 39(36):4073-4126.

Schoenfeld D and Tsiatis A. A modified log rank test for highly stratified data. Biometrika 1987; 74(1):167-175.

Schram AM, Berliner N. How I treat hemophagocytic lymphohistiocytosis in the adult patient. Blood 2015; 125(19):2908-2914.

Sikov WM, MD. Personal communication, 2014.

Sikov WM, Berry DA, Perou CM, et al. Impact of the addition of carboplatin and/or bevacizumab to neoadjuvant once-per-week paclitaxel followed by dose-dense doxorubicin and cyclophosphamide on pathologic complete response rates in stage II to III triple-negative breast cancer: CALGB 40603 (Alliance). J Clin Oncol 2015; 33(1):13-21.

Sikov WM, Berry DA, Perou CM, et al. Event-free and overall survival following neoadjuvant weekly paclitaxel and dose-dense AC +/- carboplatin and/or bevacizumab in triple-negative

breast cancer: Outcomes from CALGB 40603 (Alliance); San Antonio Breast Cancer Symposium 2015. Abstr.S2-05. Available from: https://cancerres.aacrjournals.org/content/76/4_Supplement/S2-05**.**

Sparano JA, Zhao F, Martino S, et al. Long-term follow-up of the E1199 phase III trial evaluating the role of taxane and schedule in operable breast cancer. J Clin Oncol 2015; 33(21):2353-2360.

Temple R. Hy's law: predicting serious hepatotoxicity. Pharmacoepidemiol Drug Safety 2006; 15(4):241-243.

Tutt ANJ, Garber JE, Kaufman B, et al. Adjuvant olaparib for patients with *BRCA1*- or *BRCA2*-mutated breast cancer. N Engl J Med 2021; 384(25):2394-2405.

van Mackelenbergh M, Seither F, Moebus V, et al. Effects of capecitabine as part of neo-/adjuvant chemotherapy: A meta-analysis of individual patient data from 12 randomized trials including 15,457 patients. Presented at San Antonio Breast Cancer Symposium December 2019.

Vilain RE, Menzies AM, Wilmott JS, et al. Dynamic changes in PD-L1 expression and immune infiltrates early during treatment predict response to PD-1 blockade in melanoma. Clin Cancer Res 2017; May 16. pii: clincanres.0698.2016. doi: 10.1158/1078-0432.CCR-16-0698. [Epub ahead of print]

von Minckwitz G, Schneeweiss A, Loibl S, et al. Neoadjuvant carboplatin in patients with triple-negative and HER2-positive early breast cancer (GeparSixto; GBG 66): a randomized phase 2 trial. Lancet Oncol 2014; 15(7):747-756.

Wolmark N, Wang J, Mamounas E, et al. Preoperative chemotherapy in patients with operable breast cancer: Nine-year results from National Surgical Adjuvant Breast and Bowel Project B-18. J Natl Cancer Inst Monogr 2001; 30:96-102.

APPENDIX A
ASSESSMENT OF PERFORMANCE STATUS AND ACTIVITIES OF DAILY LIVING

1.0 DETERMINATION OF PERFORMANCE STATUS

ECOG or Zubrod Scale		Karnofsky Score
0	Fully active; able to carry on all pre-disease performance without restriction	90–100%
1	Restricted in physically strenuous activity but ambulatory	70–80%
2	Ambulatory and capable of self-care, but unable to carry out any work activities	50–60%
3	Capable of only limited self-care; confined to bed or chair more than 50% of waking hours	30–40%
4	Completely disabled	10–20%

2.0 NCI DEFINITION FOR ACTIVITIES OF DAILY LIVING

The following definitions for activities of daily living (ADL) should be used when the CTCAE v4.0 grading criteria are based on ADL:

- *Instrumental ADL* refer to preparing meals, shopping for groceries or clothes, using the telephone, managing money, etc.
- *Self-care ADL* refer to bathing, dressing and undressing, feeding self, using the toilet, taking medications, and not bedridden.

APPENDIX B
COMPREHENSIVE LIST OF AUTOIMMUNE DISEASES AND IMMUNE DEFICIENCIES

Patients should be carefully questioned regarding their history of acquired or congenital immune deficiencies or autoimmune disease. Patients with any history of immune deficiencies or autoimmune disease listed in the table below are excluded from participating in the study. Possible exceptions to this exclusion could be patients with a medical history of such entities as atopic disease or childhood arthralgias where the clinical suspicion of autoimmune disease is low. Patients with a history of autoimmune-related hypothyroidism on a stable dose of thyroid replacement hormone may be eligible for this study. In addition, transient autoimmune manifestations of an acute infectious disease that resolved upon treatment of the infectious agent are not excluded (e.g., acute Lyme arthritis).

Autoimmune Diseases and Immune Deficiencies

• Acute disseminated encephalomyelitis<br>• Addison disease<br>• Ankylosing spondylitis<br>• Antiphospholipid antibody syndrome<br>• Aplastic anemia<br>• Autoimmune hemolytic anemia<br>• Autoimmune hepatitis<br>• Autoimmune hypoparathyroidism<br>• Autoimmune hypophysitis<br>• Autoimmune myocarditis<br>• Autoimmune oophoritis<br>• Autoimmune orchitis<br>• Autoimmune thrombocytopenic purpura<br>• Behçet disease<br>• Bullous pemphigoid<br>• Chronic fatigue syndrome<br>• Chronic inflammatory demyelinating polyneuropathy<br>• Churg-Strauss syndrome<br>• Crohn disease	• Dermatomyositis<br>• Diabetes mellitus type 1<br>• Dysautonomia<br>• Epidermolysis bullosa acquisita<br>• Gestational pemphigoid<br>• Giant cell arteritis<br>• Goodpasture syndrome<br>• Graves disease<br>• Guillain-Barré syndrome<br>• Hashimoto disease<br>• IgA nephropathy<br>• Inflammatory bowel disease<br>• Interstitial cystitis<br>• Kawasaki disease<br>• Lambert-Eaton myasthenia syndrome<br>• Lupus erythematosus<br>• Lyme disease, chronic<br>• Meniere syndrome<br>• Mooren ulcer<br>• Morphea<br>• Multiple sclerosis<br>• Myasthenia gravis	• Neuromyotonia<br>• Opsoclonus myoclonus syndrome<br>• Optic neuritis<br>• Ord thyroiditis<br>• Pemphigus<br>• Pernicious anemia<br>• Polyarteritis nodosa<br>• Polyarthritis<br>• Polyglandular autoimmune syndrome<br>• Primary biliary cirrhosis<br>• Psoriasis<br>• Reiter syndrome<br>• Rheumatoid arthritis<br>• Sarcoidosis<br>• Scleroderma<br>• Sjögren syndrome<br>• Stiff-Person syndrome<br>• Takayasu arteritis<br>• Ulcerative colitis<br>• Vitiligo<br>• Vogt-Koyanagi-Harada disease<br>• Wegener granulomatosis

APPENDIX C
COUNTRY-SPECIFIC SURGERY GUIDELINES

NSABP INSTITUTIONS

1.0 Marking the primary tumor site prior to initiation of therapy

Patients who are considered potential candidates for breast conservation should have the primary tumor site marked prior to initiating chemotherapy or, at least, prior to disappearance of the tumor clinically. This can be achieved with insertion of a radiopaque marker, clip, or similar marking technique as long as the technique provides assurance that the primary tumor site can be located and excised. If a clip is used, a specimen radiograph should be performed to confirm its removal.

2.0 Breast surgery

- As soon as possible following recovery from neoadjuvant therapy and after the final clinical tumor assessment, the patient should undergo a lumpectomy or mastectomy.
- Breast conservation may be selected according to patient and surgeon preferences.
  - Patients who are not considered candidates for breast conservation or who do not desire breast conservation will undergo a total mastectomy with or without immediate breast reconstruction.
  - Patients who are deemed to be good candidates for breast conservation will undergo segmental excision of the primary tumor bed. If the residual tumor is non-palpable, methods to insure adequate excision of the primary tumor site should be used to guide the excision. If tumor location was marked with clips, a specimen radiograph should be obtained intraoperatively to document that the lesion has been removed including the clips. Hemoclips should be inserted at the base of the segmental mastectomy operative site to facilitate radiotherapy treatment planning of the actual tumor bed.
  - The margins of the resected specimen of patients treated with breast conservation must be histologically free of invasive tumor and DCIS. In patients for whom pathologic examination demonstrates tumor at the margin, additional operative procedures should be performed to obtain clear margins.

3.0 Nodal staging following neoadjuvant therapy

- Post-neoadjuvant therapy axillary staging is required for all patients.
- Use of sentinel node biopsy procedure following completion of neoadjuvant therapy is at the discretion of the investigator. If sentinel node biopsy was not performed, surgical evaluation of the axilla is required.
  - If the post-neoadjuvant therapy sentinel node biopsy is positive, additional surgical evaluation of the axilla is recommended.
  - If the post-neoadjuvant therapy sentinel node biopsy is negative, further surgical nodal staging procedure is not required. NOTE: For patients who had documented node-positive disease prior to neoadjuvant therapy, it is recommended that a minimum of 2 sentinel lymph nodes be removed. The removal of at least 3 sentinel lymph nodes and the use of dual tracer for lymphatic mapping are strongly recommended. If the only sentinel node identified by isotope scan is in the internal mammary nodal chain, the axilla should be explored for a blue or suspicious node.

- If axillary nodal staging procedures were performed before randomization (see Protocol Sections 4.1.10 and 4.2.3), follow these guidelines.
  - *Even if FNA or core biopsy of an axillary node(s) was performed **before** randomization*, surgical evaluation of the axilla (either sentinel node biopsy and/or axillary dissection as described in the bullet above) must be performed following completion of neoadjuvant therapy, **regardless of whether FNA or core biopsy was positive or negative**.
  - Note: Performance of sentinel node biopsy prior to randomization is prohibited.

4.0 Tissue processing and collection

See Appendix D for suggested procedures for evaluation of post-neoadjuvant therapy surgical specimens.

GBG EUROPEAN INSTITUTIONS

[redacted]

In a neoadjuvant trial all patients should be treated surgically after completion of chemotherapy, because otherwise neither the primary objective (pCR) nor the secondary objective (rate of breast conservations) can be evaluated properly.

This surgical protocol is in line with the recommendations for standardized pathological characterization of residual disease for neoadjuvant clinical trials of breast cancer by the BIG-NABCG collaboration.

1.0 Timing of surgery

Definitive surgery should be performed 1-14 days after completion (after day 21) of the last chemotherapy cycle. In any case full hematologic recovery to normal limit is recommended. This is the case after having completed all (8 or 12) planned chemotherapy cycles or if no further chemotherapy can be given for reasons related to tumor progression and / or intolerable toxicity according to the protocol. In some trials the primary endpoint might be assessed after one part of the sequential therapy. Another algorithm might be followed then as describe in the respective protocol.

2.0 Localizing tumor in the breast

Prior to initiating chemotherapy, the breast surgeon should localize the breast tumor exactly. Clip placement into the tumor bed is strongly recommended as a routine procedure in most patients especially in women with a good response to chemotherapy after the first few cycles. The clip can be applied either at the time of diagnosis or within the first cycles of chemotherapy.

Markings on the skin can be performed in addition. With the patient standing or lying down and stretching the arm in 90° to the side, this can be done by measuring the distance from the jugulum to the nipple and taking a rectangle to determine the distance from the jugulum-nipple line to the middle of the palpable mass (see Figure 4).

After performing the markings, photographs of both breasts should be taken. The patient should be seen by the breast surgeon at least after every two chemotherapy cycles. Thus the excisional area can be best identified, even if the tumor has become non-palpable.

Prior to surgery stereotactic wire localization of the clip should be performed in cases of a clinically complete response without a palpable lesion.

If a clinically node-positive lymph node is histologically confirmed to be positive by FNA or core needle biopsy, a clip should be placed into the involved lymph node.

3.0 Extent of the excision, margins

Surgical resection volume is based on preoperative imaging (after completion of chemotherapy). All detectable residual disease should be removed with clear margins. In case of complete radiologic response, the center of the tumor bed should be removed, including any radiologic clips. Specimen radiography is mandatory in patients with non palpable lesions and a clip placement. Orientation of the specimen by the surgeon is imperative. Marking of the excisional cavity to allow targeted boost irradiation is recommended.

4.0 **Breast conservation**

Breast surgery after primary chemotherapy should be performed according to the guidelines for breast surgery without prior systemic therapy. Breast conservation techniques include lumpectomy, wide excision (segmental resection) and quadrantectomy. For medium and enlarged breasts dermoglandular flaps can be used to fill the excision site.

5.0 **Mastectomy and breast reconstruction**

If mastectomy is indicated primary or secondary reconstruction can be performed according to current guidelines (https://www.ago-online.de/). Due to the majority of these women being high risk patients, and therefore radiotherapy might be indicated, this needs to be discussed in the multidisciplinary team.

6.0 **Surgery of the axilla**

It is not recommended to perform SLNB prior to chemotherapy in clinically node negative patients, since assessment of nodal response in the axilla is important. The clinical endpoint of the study cannot be assessed, when a SLNB has been performed before chemotherapy and yielded a positive result. Therefore postchemotherapy SLNB is recommended for patients with initially unsuspicious nodes. In case a SNB has still been done and reveals an involved lymph node prior to surgery the patient has to be treated with axillary dissection at the time of definite surgery. Patients who present with suspicious nodes prior to chemotherapy (palpation and/or ultrasound) should undergo core needle biopsy of the node. If the result is positive the patient should undergo axillary dissection after chemotherapy.

Figure 4: Proposal for identifying a palpable mass after complete clinical response. However clip insertion is recommended in addition.

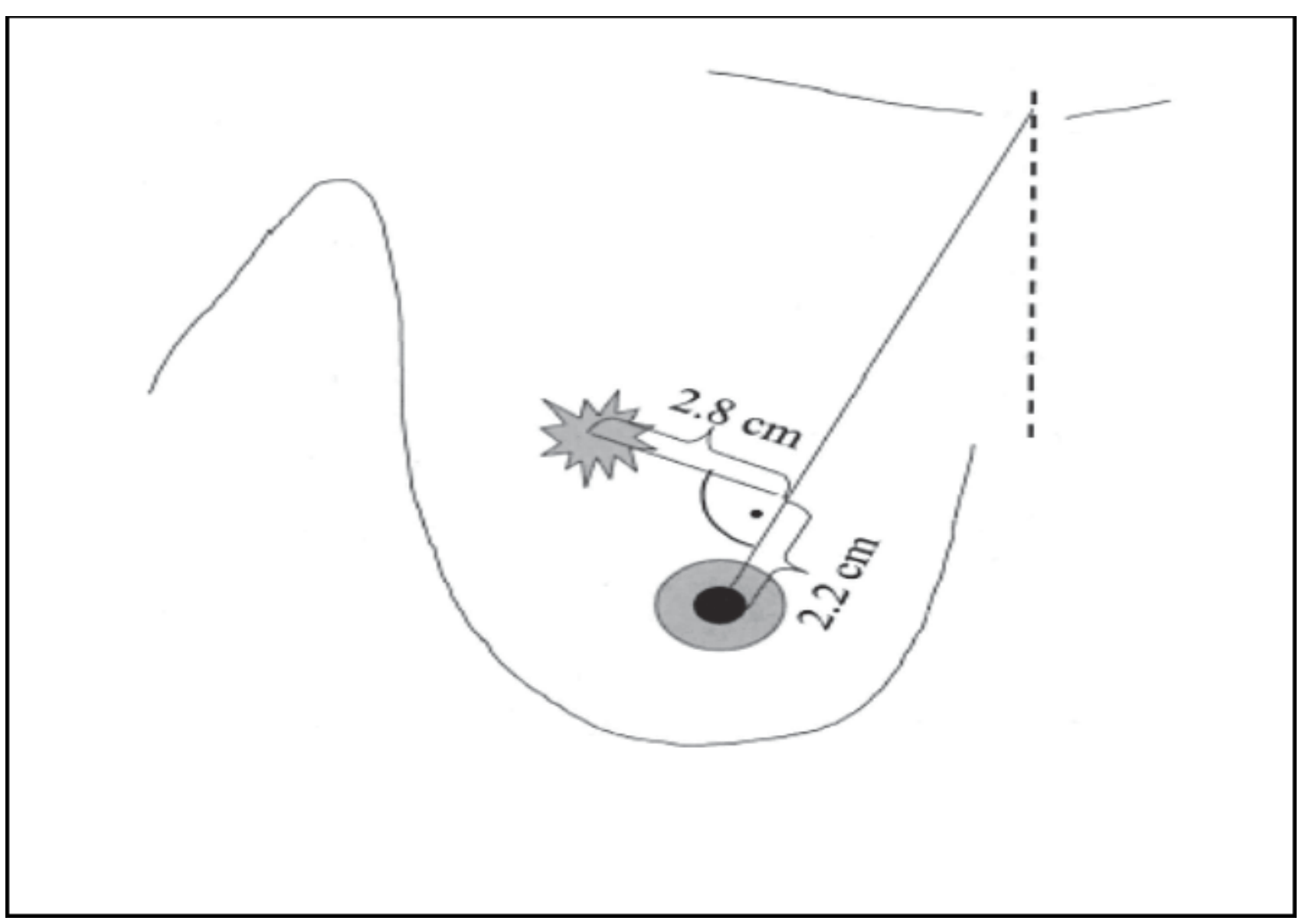

REFERENCES

Bossuyt V, Provenzano E, Symmans WF, et al. Breast International Group-North American Breast Cancer Group (BIG-NABCG) collaboration. Recommendations for standardized pathological

characterization of residual disease for neoadjuvant clinical trials of breast cancer by the BIG-NABCG collaboration. Ann Oncol 2015; Jul;26(7):1280-91.

Kaufmann M, von Minckwitz G, Smith R, et al. International expert panel on the use of primary (preoperative) systemic treatment of operable breast cancer: review and recommendations. J Clin Oncol 2003; 21(13):2600-2608.

Senofsky GM, Gierson ED, Craig PH, et al. Local excision, lumpectomy, and quadrantectomy. Surgical considerations. In: Spear SL (ed.): Surgery of the Breast, Principle and Art. Lippincott- Raven, Philadelphia, PA (USA), 1998.

APPENDIX D
COUNTRY-SPECIFIC SUGGESTED PROCEDURE FOR EVALUATION OF SURGICAL SPECIMENS

NSABP INSTITUTIONS

The following procedures used for processing breast specimens following neoadjuvant chemotherapy at MD Anderson Cancer Center are provided as guidelines for investigators. They are provided as suggestions and may be modified as needed to make them functional in participating institutions.

Specimens should be oriented with sutures by the surgeon following removal. The surgeon and breast pathologist should confer to ensure optimal evaluation of the primary tumor site for possible pCR.

In cases showing significant clinical response in the breast:

– The breast resection specimen is radiographed to identify metallic markers which were placed during or prior to chemotherapy.

– Each specimen is inked using multiple colors to identify each face of the specimen, and then sectioned into 3-5 mm slices.

– The sliced specimen is radiographed and a radiologist reviews the films to determine the presence and extent of residual tumor.

– The pathologist examines the sliced specimen grossly to identify suspicious areas and notes their proximity to margins.

– The radiographic and pathologic evaluation is discussed with the surgeon who decides whether additional margins should be obtained.

– Permanent paraffin sections of the suspicious areas and margins are obtained. The number of sections taken is based on the gross inspection, radiologic features, and size of the resection specimen.

– The entire radiographic abnormality as well as firm and suspicious appearing breast tissue is submitted for histologic evaluation.

– In general, for non-palpable (clinical complete response) cases at least 10-15 blocks are examined to assess the presence of residual microscopic disease.

In cases with residual palpable mass (partial clinical response or no response in the breast):

– The resection specimen is inked and sectioned into 3-5 mm slices.

– The pathologist examines the slices and determines the tumor size on gross evaluation and confirms the tumor size by microscopic evaluation.

Evaluation of axillary lymph nodes regardless of response:

All axillary lymph nodes are also carefully evaluated by serial gross sectioning.

– One or two representative histologic sections are evaluated for lymph nodes that contain grossly identifiable metastatic carcinoma.

– The lymph nodes that do not show grossly identifiable tumor are submitted for histologic evaluation in their entirety. One representative histologic section is evaluated per paraffin block. Immunohistochemical staining for cytokeratin is not routinely performed on negative nodes.

GBG EUROPEAN INSTITUTIONS

[redacted]

The core needle biopsies and resected breast tissues are evaluated histopathologically in accordance with the current diagnostic standards for breast pathology, for example as summarized in the National S3 Diagnostic Guideline, Therapy and Aftercare for Breast Cancer of the German Cancer Association, the AGO Organkommission Guideline 'Mamma' or in the relevant international guidelines. [1, 2, 3]

For an update on guidelines for evaluation of specimen after neoadjuvant chemotherapy, we recommend the international recommendations published in 2015:

- Provenzano E, Bossuyt V, Viale G, Cameron D, Badve S, Denkert C, MacGrogan G, Penault-Llorca F, Boughey J, Curigliano G, Dixon JM, Esserman L, Fastner G, Kuehn T, Peintinger F, von Minckwitz G, White J, Yang W, Symmans WF. Standardization of pathologic evaluation and reporting of postneoadjuvant specimens in clinical trials of breast cancer: recommendations from an international working group. Mod Pathol. 2015 Sep;28(9):1185-201. doi: 10.1038/modpathol.2015.74. Epub 2015 Jul 24. PubMed PM1D: 26205180.

- Bossuyt V, Provenzano E, Symmans WF, Boughey JC, Coles C, Curigliano G, Dixon JM, Esserman LJ, Fastner G, Kuehn T, Peintinger F, von Minckwitz G, White J, Yang W, Badve S, Denkert C, MacGrogan G, Penault-Llorca F, Viale G, Cameron D; Breast International Group-North American Breast Cancer Group (BIG-NABCG) collaboration. Recommendations for standardized pathological characterization of residual disease for neoadjuvant clinical trials of breast cancer by the BIG-NABCG collaboration. Ann Oncol. 2015 Jul;26(7):1280-91. doi: 10.1093/annonc/mdv161. Epub 2015 May 27. Review. PubMed PM1D: 26019189.

It is the task of pathohistological diagnostics to determine the extent and regression grade of the tumor after chemotherapy. Pathomorphologically determined complete remission (pCR) is one of the most important prognosis factors for relapse-free survival after neoadjuvant chemotherapy. Therefore, the pathological results should be collected in a standardized way.
Special features present in the main where there is no or only very little tumor tissue in evidence after therapy. The following section serves as an only very brief presentation of the generally known diagnostic standards. This is followed by a more detailed review of the special features of histological diagnostics in neoadjuvant chemotherapy.

1. Standardized diagnostics

1.1 Core needle biopsy prior to treatment

Core needle biopsies fixed in buffered formalin and then evaluated histopathologically after paraffin embedding. A frozen section should not be used in this diagnostic procedure in order to avoid methodological problems in grading and in immunohistological testing.
The results produced by the core needle biopsy should include:
a histopathological tumor classification according to the WHO (2012).[4]
the Elston-Ellis histological grading system [5]; the mitosis rate should be determined based on the individual field view size.
hormone receptor expression (given as %), HER-2 status (HER-2 score by means of standardized immunohistochemistry and/or in-situ hybridization).
The following material should be sent to the central pathology laboratory of GBG:

- the paraffin block of the diagnostic core needle biopsy prior to treatment
- a copy of the histology report containing the ER, PR and HER2 status (pseudonymized, with study name, patient number and site number)

1.2 Resected tumor tissue

After completion of neoadjuvant chemotherapy, the tumor is resected, with either breast conservation or mastectomy according to the clinical assessment. The following clinical information is necessary for histological diagnostics:

- confirmation that neoadjuvant chemotherapy has been carried out;
- tumor localization within the specimen;
- tumor size as determined clinically prior to treatment.
- Clinical response
- Information about a possible pretherapeutic sentinel node procedure

The current diagnostic standards should be applied to the histopathological preparation. Special features are necessary when determining the extent of the existing residual tumor and tumor regression. We strongly recommend to include the Residual Cancer Burden (RCB) in each pathology report for improvement of international standardization. This requires simply to add the tumor cellularity, the size of the tumor bed as well as the size of the largest lymph node metastasis to the histopathology report. From these parameters the RCB can be calculated at any time.

The RCB is further explained on these websites:

http://www3.mdanderson.org/app/medcalc/index.cfm?pagename=j sconvert3

http://www.pathology.at/pathologinnen/fachlicheinfo/233-rcb-score

The standardized results pertaining to the resected tumor tissue after completion of neoadjuvant chemotherapy should include:

- a histopathological tumor classification according to the WHO (2012).
- Elston-Ellis [5] histological grading marked 'Condition after chemotherapy'. The mitosis rate should be determined taking due account of the individual field view size.
- Tumor size in two dimensions, where applicable details of multifocality or multicentricity with the size of the residual tumor foci.
- Tumor cellularity, using the standardized evaluation schema developed at the MD Anderson Cancer Center, which is available here: http://www3.mdanderson.org/app/medcalc/index.cfm?pagename=j sconvert3
- Intraductal tumor components: Amount, extent and grading pursuant to WHO (2012)
- R-status with distance from the resection margins in mm, where applicable, data to be given separately for the invasive and intraductal tumor components.
- Hormone receptor status (given as %).
- HER-2 status (HER-2 score).
- Regression grade (see below). [6]
- Lymph vessel invasion, vein invasion.
- Lymph node status, including the size of the largest lymph node metastasis
- yTNM classification.

After conclusion of histological diagnostics, the following material should be sent to the central tumor bank:

- a representative paraffin block of the tumor (If histological examination shows no evidence of residual tumor, a representative block from the former tumor bed with DCIS or inflammatory changes should be sent.)
- a representative paraffin block of one lymph node (if possible with tumor infiltrate, but a negative node will be fine also, if the tumor is nodal negative)
- a copy of the histological report of tumor resection as well as lymph nodes which contains the macroscopic assessement as well as the pathological response (patient name and date of birth should be crossed out)

Two paraffin blocks will be stored in the central tumor bank of the GBG for translational research project (based on the informed consent of the patient.

2. Special features of the histopathological examination

2.1 Special features applying to section and the macroscopic assessment

Delimitable tumor focus

If a delimitable tumor focus is found, section generally poses no difficulties. At least three tumor blocks are embedded — with larger tumors at least one tumor cross section at the point of maximum diameter. Additionally, tissue from all resection margins and, where applicable, also fibrosed areas adjacent to the tumor are examined.

Weakly delimitated tumor bed

If macroscopically the tumor focus cannot be sharply delimitated, the former tumor bed is examined in order to document the response to preoperative chemotherapy. In addition, the clinical partners should indicate or mark the extent and site / quadrant of the tumor. With this information, the tumor bed is then generally detectable as an irregular fibrosed area. the number of tissue blocks should be based on the pretherapeutic tumor size. Here, too, examination is based on the maximum tumor diameter (as a guide, at least one block per cm of tumor — pre-treatment size) and the distance to all resection margins is documented. Additionally, fibrosed areas adjacent to the tumor bed and in the region of the resection margins suspected of being tumorous should be examined.

No clearly detectable tumor bed

If both macroscopically and on the basis of the clinical information, no obvious tumor bed is detectable, smaller diagnostic excisions should be embedded completely at the discretion of the pathologist.

– Where larger amounts of tissue are resected, the first step is exploratory evlauation in the fibrosed areas. Here, too, the extent of the section should be governed by the size of the tumor prior to treatment. As a guide, roughly one block per cm tumor diameter should be. If there is macroscopic evidence of tumor infiltrates, DCIS or inflammatory / regressive changes, a follow up examination should then be made specifically of tissue from this area by extending the section. The histological results should show the total number of blocks examined.

2.2 Regression grade

An overview of typical histological and cytological changes after chemotherapy can be found, for example, in Langer et al. [8] The regression grade is modified using Sinn's method. [6]

- Regression grade 0: no effect
- Regression grade 1: tumor sclerosis with resorptive inflammation and/or cytopathic effects.
- Regression grade 2: extensive tumor sclerosis with only focal / multifocal minimally invasive residual tumor (< 0.5 cm).
- Regression grade 3 +: only non-invasive residual tumor in the breast, lymph nodes unaffected
- Regression grade 3 -: only non-invasive residual tumor in the breast, although lymph nodes are affected.
- Regression grade 4: no residual (non-invasive or invasive) tumor in the breast, but affected nodes
- Regression grade 5: no residual (non-invasive or invasive) tumor in the breast and lymph nodes.

In addition we recommend to include the Residual Cancer Burden (RCB), as explained above.

2.3 Special features of regression grades 3 and 4

In this case, histological examination reveals no residual presence of the carcinoma or only evidence of DCIS. If regression grades 3 or 4 are suspected, further tissue should be examined where applicable depending on the macroscopic results so that an adequate histological assessment of the whole former tumor bed can be made. This is because in some cases only very small multifocal residual infiltrates measuring a few millimeters are found after therapy. Also, if the infiltrate is severely inflamed, it can be difficult to

distinguish between regressively changed tumor cells and histiocytes. Here, immunohistological examinations should be used to detect or rule out regressively changed residual tumor cells in the region of the sclerotic areas that have changed because of inflammation.

2.4 Assessment of tumor extent / Pseudo-multifocality

Determination of tumor size takes microscopic and macroscopic findings into account. If histological examination reveals several tumor cell islands interspersed with fibrotic zones in a macroscopically clearly identifiable tumor bed, the total macroscopic extent of the tumor and not the largest individual focus should be used for tumor size determination (therapy-induced pseudo-multifocality). [8] Where applicable, the attempt can be made to find immunohistological evidence of individual degeneratively changed tumor cells in the fibrotic areas.

A multifocal tumor should only be diagnosed if there are different tumor foci which, taking due account of the macroscopic findings as well, are not located in a common fibrosed area corresponding to the tumor bed prior to treatment.

2.5 Assessment of the resection margin

If the tumor is only weakly delimitated or discontinuous after treatment, particular caution should be exercised when assessing the resection margins. Where applicable here, discontinuous fibrotic foci should be examined with reference to the resection margin.

2.6 Lymph node examination

When examining lymph nodes, it may be necessary to grade regression separately. Here, too, immunohistological examinations should be carried out, where applicable, to find evidence of small regressively changed portions of residual tumor.

REFERENCES

'Deutsche Krebsgesellschaft. Informationszentrum far Standards in der Onkologie (ISTO): Interdiziplinare Leitlinie der Deutschen Krebsgesellschaft und der beteiligten medizinisch-wissenschaftlichen Fachgesellschaften. Diagnostik, Therapie und Nachsorge des Mammakarzinoms der Frau — eine nationale S3 Leitlinie. http://www.krebsgesellschaft.de - Leitlinien Mammakarzinom

[2]The Association of Directors of Anatomic and Surgical Pathology: ADASP Checklists for Surgical Pathology Reports of malignant neoplasms. http://www.adasp.org

[3]Guski H. Institut fur Pathologic der Charite — Mammadiagnostik. http://www.charite.de/ch/patho

talchani 5, Ellis JO, et al, (eds.) WHO Classification of Tumors of the breast. IARC Press, Lyon (F), 2012 [5]Elston CW & Ellis JO. Pathological prognostic factors in breast cancer. I. The value of histological grade in breast cancer: Experience from a large study with long-term follow-up. Histopathology 19:403-10, 1991

[6]Sinn HP, Schmid H, Junkermann H, et al. Histologische Regression des Mammakarzinoms nach primarer (neoadjuvanter) Chemotherapie. Geburtshilfe Frauenheilkd 54:552-8, 1994

'Wittekind C, Meyer HJ, Bootz F. TNM Klassifikation maligner Tumoren. 6. Auflage, Springer Verlag, Berlin Heidelberg New York, 2003

[8]Langer F, Lfick HJ, Kreipe HH. Therapieinduzierte Tumorregression beim Mammakarzinom. Pathologe 25:45560, 2004

Supplemental Information 3: Summary of clinically important protocol amendments

Protocol Title: A Randomized, Double-Blind, Phase III Clinical Trial of Neoadjuvant Chemotherapy with Atezolizumab or Placebo in Patients with Triple-Negative Breast Cancer Followed by Adjuvant Continuation of Atezolizumab or Placebo

The study protocol was amended 6 times during the conduct of the study (versions v4.0–v10.0). Key changes are summarized below.

**Amendment/ Protocol version**	**Changes**
1/v4.0	- Updated safety according to Atezolizumab IB Version 14 (October 2018).<br>- Updated efficacy data on atezolizumab with nab-paclitaxel in metastatic triple-negative breast cancer: analysis of PFS and OS by PD-L1 status in IMpassion130 demonstrated differential activity in PD-L1-positive and negative cohorts. Central PD-L1 testing was implemented as a stratification variable (positive vs. negative or indeterminate) using the criteria and assay employed in IMpassion130.
2/v5.0	- Updated Safety according to Atezolizumab IB Version 15 (July 2019) and IB Addendum to IB 15 (Dec 2019).<br>- Addition of disease-free survival (DFS) as secondary aim and endpoint.<br>- Adjustment inclusion and exclusion criteria.<br>- Inclusion of capecitabine as optional post-neoadjuvant treatment in non-pCR patients following neoadjuvant therapy per investigator discretion and local guidelines.<br>- Marking of primary tumor site added as requirement for study entry.<br>- Updated duration of Atezolizumab/Placebo to 3-4 doses with EC q2weeks.
3/v6.0	- Updated Safety according to Atezolizumab IB Version 18 (Jul 2021).<br>- Inclusion of olaparib for patients with germline *BRCA1* and *BRCA2* mutations with residual invasive cancer at the time of surgery.<br>- The endpoint of pCR changed to a secondary endpoint.<br>- EFS sole primary endpoint for assessment of efficacy to fully preserve the alpha for the EFS assessment.<br>- Several of the secondary endpoints moved to exploratory endpoints with the exception of OS, DFS, DDFS, Toxicity, Cardiac safety lead-in study.<br>- Sample size determination and protocol duration for the primary endpoint of EFS, Enrollment period, and Follow-up sections updated with the final study enrollment period, accrual information, and the time to obtain the updated number of EFS events.
4/v7.0	- Updated Safety according to Atezolizumab IB Version 19 (Aug 2022).<br>- Strategy for pooling strata added to the analysis plan for EFS at both the interim analysis and the definitive analysis if there exist strata with fewer than 4 events in order to address the risk of over-stratification.
5/v8.0	Non-substantial amendment. No key changes (EudraCT No. replaced by EU-CT-No.).
6/v10.0*	As the primary analysis for EFS did not meet criteria for superiority, trial was closed without additional follow-up for overall survival.

*protocol version v9.0 was never made effective.

Statistical Analysis Plan

SPONSOR:	NSABP
PROTOCOL TITLE:	A Randomized, Double-Blind, Phase III Clinical Trial of Neoadjuvant Chemotherapy with Atezolizumab or Placebo in Patients with Triple-Negative Breast Cancer Followed by Adjuvant Continuation of Atezolizumab or Placebo
PROTOCOL VERSION:	Version 8.0 Europe (Amendment #5), dated 05 January 2024<br>Final USA (Amendment #7), dated 20 December 2022
STUDY CODE:	NSABP B-59/GBG 96

[redacted]

[redacted]

Version History

Version	Change implemented
2.0	Section 4.6 Rules for Missing or Partial Dates<br>Section 9 Efficacy Evaluation including collapse of stratification factors, subgroup analysis and pCR endpoint analysis<br>Section 11.1 Extent of Exposure including relative total dose intensity<br>Section 11.2 Adverse Events
3.0	Section 9.1.1 Event-Free Survival (EFS) including power calculation and an update to number of EFS events for the definitive analysis to be performed

[redacted]

Table of Contents

Contents

1. LIST OF ABBREVIATIONS AND DEFINITION OF TERMS 4
2. INTRODUCTION 6
3. STUDY DESIGN AND OBJECTIVES 6
3.1 Study Objectives 6
3.1.1 Primary Objective 6
3.1.2 Secondary Objectives 6
3.1.3 Exploratory Objectives 6
3.1.4 Correlative Science Objectives 7
3.2 Study Design 7
3.3 Sample Size Justification 10
4. GENERAL ANALYSIS DEFINITIONS 11
4.1 Study Period Definitions 11
4.1.1 Treatment Periods 11
4.2 Planned Analyses 13
4.2.1 Interim EFS Analysis 13
4.2.2 Definitive EFS Analysis 13
4.3 Definition of Populations 14
4.3.1 Intention-To-Treat (ITT) Population 14
4.3.2 Safety (SAF) Population 14
4.3.3 Cardiac Safety Lead-in (CSAF) Population 14
4.3.4 Atezolizumab/Placebo Only (APO) Population 14
4.3.5 Atezolizumab/Placebo Plus Capecitabine (APC) Population 14
4.4 Treatment Assignment and Treatment Arms 14
4.5 Calculated Variables 15
4.6 Rules for Missing or Partial Dates 16
4.7 Methods to Be Used for Handling Missing Data 17
4.8 Changes to Analysis Specified in Protocol 17
5. STUDY SUBJECTS 17
5.1 Disposition of Subjects 17
5.2 Protocol Deviations 17
5.3 Inclusion and Exclusion Criteria 17
6. DEMOGRAPHIC AND OTHER BASELINE CHARACTERISTICS 17
7. MEDICAL HISTORY 18
8. CONCOMITANT MEDICATIONS 18
9. EFFICACY EVALUATION 18
9.1 Primary Efficacy Analyses 19
9.1.1 Event-Free Survival (EFS) 19
9.1.1.1 Descriptive Analyses 20
9.1.1.2 Subgroup Analysis 20
9.1.1.3 Sensitivity Analysis 1 20
9.1.1.4 Sensitivity Analysis 2 21
9.1.1.5 Sensitivity Analysis 3 21
9.1.1.6 Exploratory Analysis 21

[redacted]

9.2 Secondary Efficacy Analyses 21
9.2.1 Overall Survival (OS) 22
9.2.2 pCR in Breast and Lymph Nodes (ypT0/Tis ypN0) 22
9.2.2.1 Subgroup Analysis 23
9.2.2.2 Exploratory Analysis 23
9.2.3 Distant Disease-Free Survival (DDFS) 23
9.2.3.1 Sensitivity Analysis 24
9.2.4 Disease-Free Survival (DFS) 24
9.3 Exploratory Efficacy Analyses 25
9.3.1 pCR in Breast (ypT0/Tis) 25
9.3.2 pCR in Breast and Lymph Nodes (ypT0/ypN0) 25
9.3.3 Positive Nodal Status Conversion Rate 25
9.3.4 Recurrence-Free Interval (RFI) 26
9.3.4.1 Sensitivity Analysis 26
9.3.5 Brain-Metastases-Free Survival (BMFS) 26
9.4 Correlative Science Analyses 27

10. SURGERY 27

11. SAFETY EVALUATION 28

11.1 Extent of Exposure 28
11.2 Adverse Events 31
11.2.1 Treatment-Emergent Adverse Events (TEAEs) 31
11.2.2 Fatal AEs and Serious Adverse Events 34
11.3 Clinical Laboratory Determination 34
11.4 Vital Signs, Physical Findings and Other Observations Related to Safety 35
11.4.1 LVEF 36
11.4.2 12-Lead ECG Testing 36
11.5 Cardiac Safety Lead-in Study Endpoints 36
11.5.1 Troponin-T 36
11.5.2 LVEF 37

[redacted]

1. List of Abbreviations and Definition of Terms

Abbreviation	Term
AC	Doxorubicin/Cyclophosphamide
AE	Adverse Event
ALT	Alanine Aminotransferase
ANC	Absolute Neutrophil Count
AST	Aspartate Aminotransferase
BMFS	Brain-Metastases-Free Survival
CSAF	Cardiac Safety Population
CNS	Central Nervous System
CR	Complete Response
CRF	Case Report Form
CTC	Common Toxicity Criteria
CTCAE	Common Terminology Criteria for Adverse Events
DDFS	Distant Disease-Free Survival
DFS	Disease-Free Survival
DMU	Deleterious Mutation
DSMB	Data Safety Monitoring Board
EC	Epirubicin/Cyclophosphamide
ECOG	Eastern Cooperative Oncology Group
ECG	Electrocardiogram
EFS	Event-Free Survival
FNA	Fine Needle Aspiration
INR	International Normalized Ratio
ICH	International Council for Harmonisation of Technical Requirements for Pharmaceuticals for Human Use
ITT	Intention-To-Treat
K-M	Kaplan-Meier
LLN	Lower Limit of Normal
LVEF	Left Ventricular Ejection Fraction
MedDRA	Medical Dictionary for Regulatory Activities
MRI	Magnetic Resonance Imaging
NCI	National Cancer Institute
OS	Overall Survival
pCR	Pathologic Complete Response
PD-L1	Programmed Death-ligand 1
PH	Proportional Hazards
PT	Prothrombin Time
RFI	Recurrence-Free Interval
SAE	Serious Adverse Event
SAF	Safety
SOC	System-Organ Class
PT	Preferred Term
TE	Treatment Emergent
TEAE	Treatment Emergent Adverse Event
TILs	Tumor-Infiltrating Lymphocytes
TNBC	Triple Negative Breast Cancer
TSH	Thyroid-Stimulating Hormone

[redacted]

WBC	White Blood Cells
WHO	World Health Organization
WP	Weekly Paclitaxel

[redacted]

2. Introduction

This Statistical Analysis Plan was written for the clinical trial NSABP B-59/GBG-96, a randomized, double-blind, Phase III clinical trial of neoadjuvant chemotherapy with Atezolizumab or placebo in patients with triple-negative breast cancer followed by adjuvant continuation of Atezolizumab or placebo. The ICH guidelines E3 "Structure and Content of Clinical Study Reports" and E9(R1) "Statistical Principles for Clinical Trials" were used as a guide to the writing of the plan. This SAP is based on NSABP B-59/GBG-96 protocols as follows:

- Version 7.0 Europe (Amendment #4), dated 13 December 2022
- Final USA (Amendment #7), dated 29 November 2022

3. Study Design and Objectives

3.1 Study Objectives

3.1.1 Primary Objective

There is one primary objective:

- Event-free-survival (EFS):
  To determine whether the addition of atezolizumab to chemotherapy (weekly paclitaxel plus carboplatin followed by AC *or* EC) followed by adjuvant atezolizumab improves event-free survival (EFS) in subjects with triple-negative breast cancer.

3.1.2 Secondary Objectives

The secondary objectives are:
To determine, in subjects with triple-negative breast cancer, whether the addition of atezolizumab to chemotherapy (weekly paclitaxel plus carboplatin followed by AC *or* EC) followed by adjuvant atezolizumab improves:

- Overall survival (OS)
- Pathologic complete response (pCR) in the breast and lymph nodes (ypT0/Tis ypN0)
- Disease-free survival (DFS)
- Distant-disease-free survival (DDFS).

Toxicity:

- To evaluate toxicity associated with study therapy added to chemotherapy and radiation therapy
- To evaluate immune-adverse events of special interest.

Cardiac safety lead-in study**:**

- To assess for potential augmentation of anthracycline-related cardiac toxicity with co-administration of study therapy.

3.1.3 Exploratory Objectives

Exploratory objectives are:
To determine, in subjects with triple-negative breast cancer, whether the addition of atezolizumab to chemotherapy (weekly paclitaxel plus carboplatin followed by AC or EC) followed by adjuvant atezolizumab improves:

[redacted]

- Pathologic complete response in the breast (ypT0/Tis)
- Pathologic complete response in the breast and lymph nodes (ypT0 ypN0)
- Positive nodal status conversion rate
- Recurrence-free interval (RFI)
- Brain metastases free survival (BMFS).

3.1.4 Correlative Science Objectives

The correlative science objectives are:

- To evaluate baseline expression of PD-L1 and expression of PD-L1 following initial dose of atezolizumab with paclitaxel plus carboplatin as predictor for pCR of breast and nodes (ypT0/Tis ypN0) following neoadjuvant administration of atezolizumab with chemotherapy.
- To evaluate baseline expression of PD-L1 and expression of PD-L1 following initial dose of atezolizumab with paclitaxel plus carboplatin as predictor for EFS following neoadjuvant administration of atezolizumab with chemotherapy followed by adjuvant administration of atezolizumab.
- To evaluate baseline percentages of tumor-infiltrating Lymphocytes (TILs) and percentage of TILs following initial dose of atezolizumab with paclitaxel plus carboplatin as predictor for pCR of breast and nodes (ypT0/Tis ypN0) following neoadjuvant administration of atezolizumab with chemotherapy.
- To evaluate baseline percentages of TILs and percentage of TILs following initial dose of atezolizumab with paclitaxel plus carboplatin as predictor for EFS following neoadjuvant administration of atezolizumab with chemotherapy followed by adjuvant administration of atezolizumab.
- To evaluate percentages of TILs in subjects with residual breast cancer at time of surgery following neoadjuvant administration of atezolizumab with chemotherapy followed by adjuvant administration of atezolizumab as predictor for EFS.
- To use baseline and on therapy specimens to explore potential new biomarkers of responses and resistance to neoadjuvant administration of atezolizumab with chemotherapy followed by adjuvant administration of atezolizumab.

3.2 Study Design

This is a randomized, double-blinded clinical trial of neoadjuvant and adjuvant administration of atezolizumab/placebo in subjects with high risk TNBC to evaluate the efficacy and safety of neoadjuvant administration of atezolizumab with a sequential regimen of weekly paclitaxel with every-3-week carboplatin followed immediately by neoadjuvant administration of atezolizumab/placebo with AC/EC. Subjects will then undergo surgery. Following recovery from surgery, subjects will initiate approximately 6 months of adjuvant therapy with atezolizumab/placebo and receive the same investigational agent they received preoperatively. Administration of radiation therapy will be based on local standards at the discretion of subjects and investigators, but if administered, atezolizumab/placebo will be administered concurrently.

Following surgery, determination of pCR status and recovery from surgery, subjects who did not discontinue atezolizumab/placebo due to toxicity during the neoadjuvant therapy will resume the original randomized investigational therapy assignment and continue the

[redacted]

therapy as adjuvant treatment until 1 year after they received the initial dose of atezolizumab/placebo. Since activity of radiation therapy may also be augmented by inhibition of PD-1/PD-L1, radiation therapy, if indicated, should be co-administered with atezolizumab/placebo. This will allow for collection of safety data related to coadministration of atezolizumab with radiation therapy on a blinded, placebo-controlled trial. Subjects with residual invasive cancer at the time of surgery may receive capecitabine with atezolizumab/placebo in the adjuvant setting per investigator discretion and local guidelines.

The cardiac safety lead-in component of the study in the first 60 subjects who begin AC/EC incorporates assessment of a 12-lead electrocardiogram (ECG) and serum troponin-T collected between completion of the administration of the 1st and 3rd dose of AC/EC and administration of the corresponding doses of atezolizumab/placebo administered on those days of treatment. Subjects also have a baseline troponin-T obtained just prior to administration of the first dose of doxorubicin or epirubicin. If troponin-T is found to be elevated, the subject will undergo evaluation by a cardiologist. All subjects on this protocol have LVEF assessments at baseline, after surgery, and 18 months from randomization. As a component of the extra monitoring in the first 60 subjects in the cardiac safety lead-in, an additional assessment of LVEF with echocardiogram or MUGA scan is also obtained prior to the 3rd dose of doxorubicin or epirubicin. Results of the troponin assessments, all available LVEF assessments, and cardiac safety data are evaluated by the Data Safety Monitoring Board (DSMB) when the last of the initial 20 subjects who initiate AC/EC undergo their scheduled post-surgery LVEF assessment. In addition, when the last of the first 60 subjects to initiate AC/EC undergo their scheduled post-surgery LVEF assessment, results of the troponin assessments, LVEF assessments, and cardiac safety data from all 60 subjects are evaluated by the DSMB.

The cardiac monitoring plan is re-assessed after each of the reviews of the cardiac safety data from the lead-in cohorts by the DSMB. If safety concerns are not identified by the DSMB, the additional monitoring comprising the cardiac safety lead-in ends after the first 60 subjects and monitoring for the rest of the subject population will proceed as planned.

The schedules of visits are listed in Tables 2-4 of the study protocol.

[redacted]

Figure 1 B-59/GBG 96-GeparDouze Schema

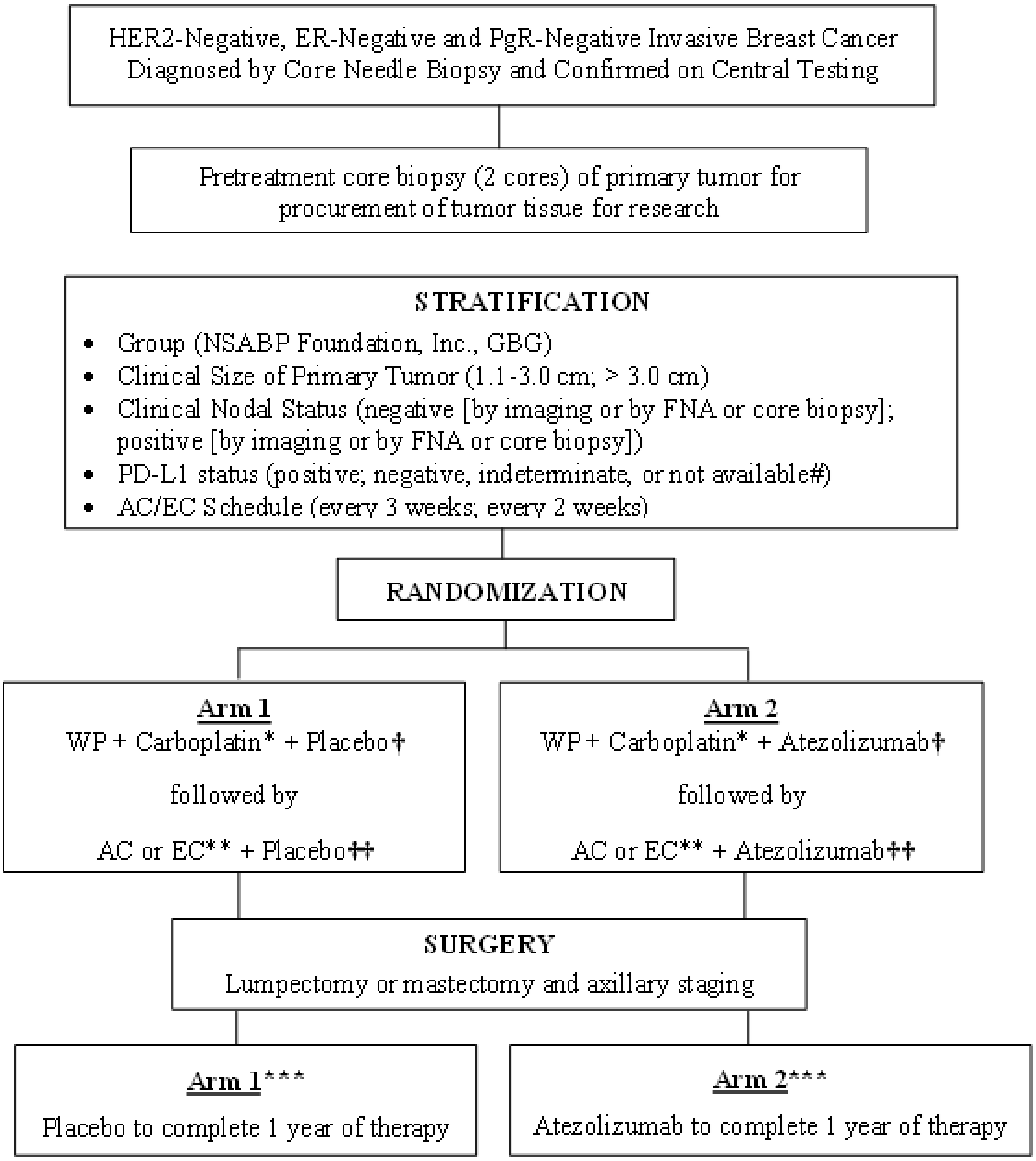

#	PD-L1 status of patients were not available at randomization for patients enrolled prior to amendments in July 2019.
*	Paclitaxel 80 mg/m2 IV weekly x 12 doses (WP) + Carboplatin AUC of 5 IV Day 1 every 3 weeks for 4 cycles.
**	Doxorubicin (A) 60 mg/m2 IV + cyclophosphamide (C) 600 mg/m2 IV Day 1 every 2 or 3 weeks for 4 cycles **OR** Epirubicin (E) 90 mg/m2 IV + cyclophosphamide (C) 600 mg/m2 IV Day 1 every 2 or 3 weeks for 4 cycles. Choice of anthracycline and schedule per investigator discretion.
†	Atezolizumab 1200 mg or placebo IV Day 1 every 3 weeks for 4 doses.
††	Atezolizumab 1200 mg or placebo IV Day 1 every 3 weeks for 3 to 4 doses depending on AC/EC schedule used.
***	Atezolizumab 1200 mg or placebo IV Day 1 every 3 weeks after surgery until 1 year after the first dose.

[redacted]

Study design AC/EC q3 weekly

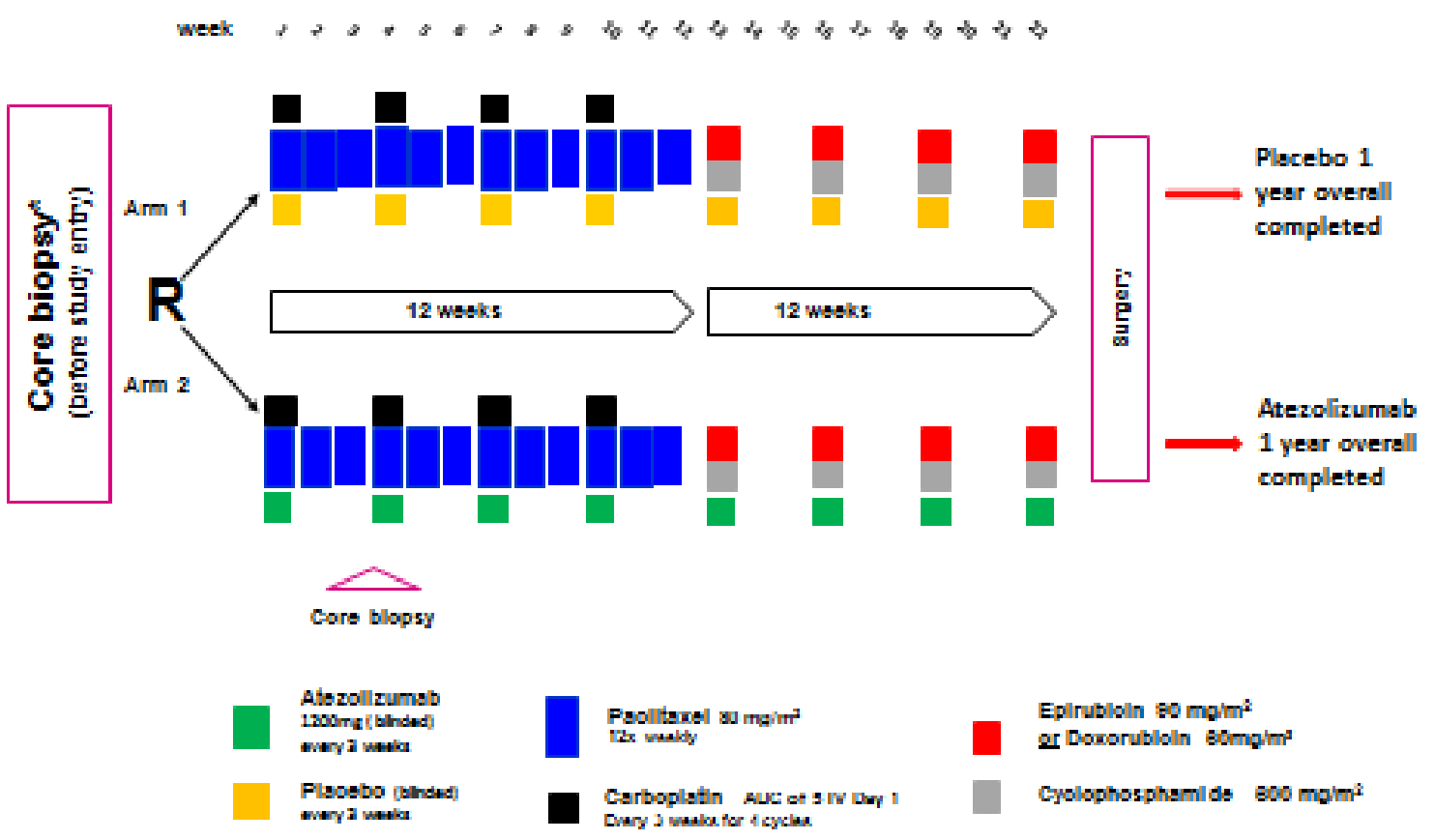

Study design AC/EC q2 weekly

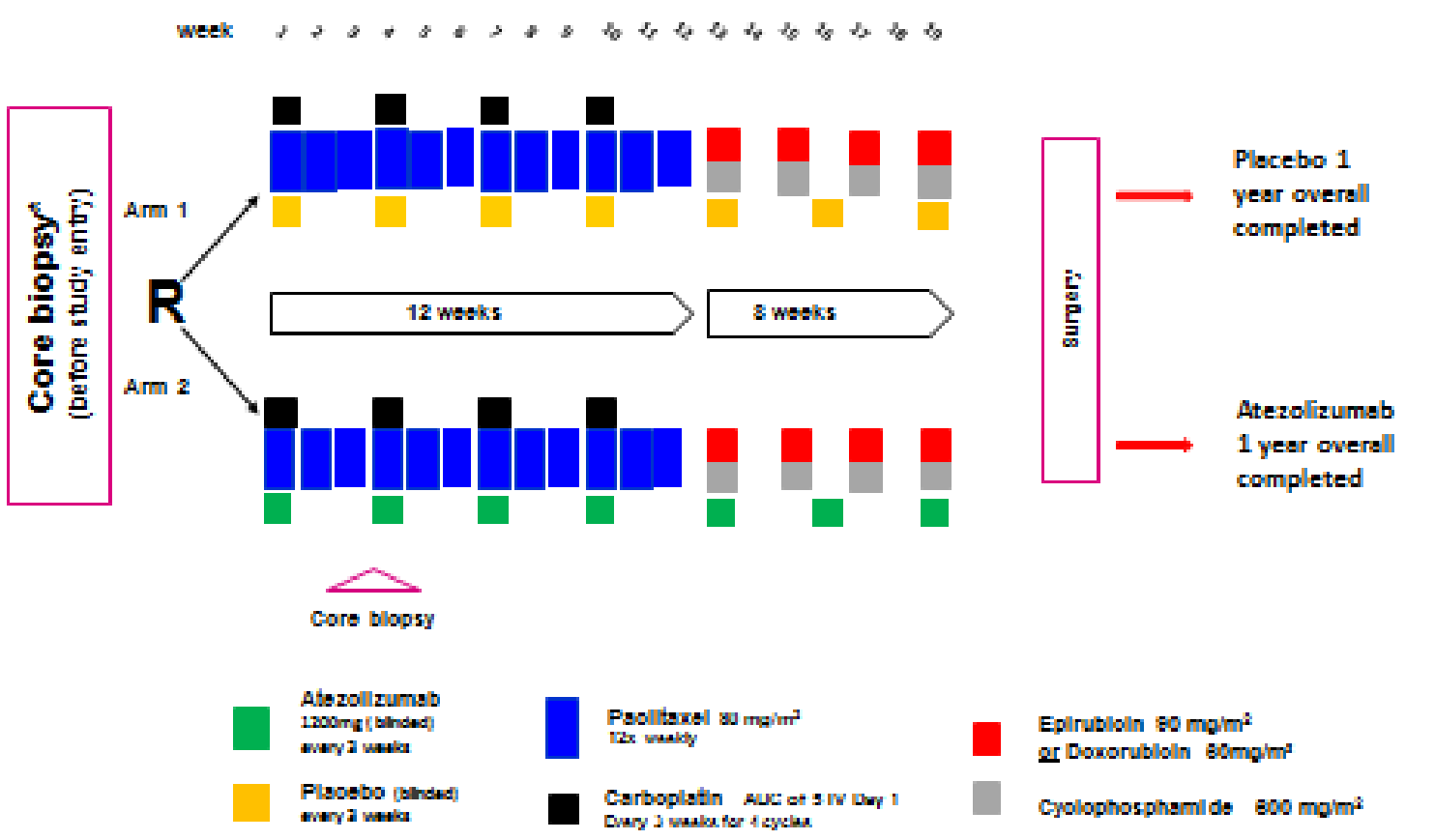

3.3 Sample Size Justification

The experiment-wise, two-sided alpha level for the primary endpoint EFS is 0.05.

The sample size for this trial is based on the requirements for testing of the EFS endpoint. The hazard rates used for determination were taken from the experience in NSABP B-40 trial. The regimen of docetaxel followed by AC arm employed in NSABP B-40 was similar in efficacy to AC followed by weekly paclitaxel in ECOG 1199 (Sparano 2015). In NSABP B-40, the annual hazard rates were not constant and varied substantially over the first 4 to 5 years. Thus, instead of using a constant hazard rate across time, yearly-specific hazards rates were used. These rates were then reduced by 20% to account for an anticipated further reduction in event rates with addition of carboplatin to the treatment regimen based on CALGB 40603 (Sikov SABCS 2015). To achieve monthly precision in estimates of accrual and study duration, the calculation of sample size was based on monthly hazard rates converted from each of the yearly hazard rates described above.

[redacted]

The resulting monthly hazard rates are 0.00460 during year 1, 0.01020 during year 2, 0.00440 during year 3, 0.00186 during year 4 and 0.00200 for year 5 and all subsequent years.

The original sample size was based on having sufficient statistical power to detect a hazard ratio of 0.70 at alpha=0.045 assuming a lost-to-follow-up rate of 0.00083 per month. A sample size of 760 subjects per arm for a total sample size of 1,520 subjects followed to obtain 269 EFS events would provide a test of hypothesis with 80% statistical power at an overall alpha level of 0.045. After allowing for a ramp-up of accrual in the first eighteen months to 40 subjects per month and a further ramp-up to 90 subjects per month by accrual month 28, it was anticipated that the accrual rate would peak at 105 subjects per month by accrual month 32. With this accrual pattern, it would take about 34 months to accrue 1,520 subjects. Follow-up of an additional 30 months after completion of accrual (total study time of 64 months) would be sufficient to obtain 269 EFS events required for the final EFS analysis.

Accrual to NSABP B-59/GBG 96-GeparDouze began in December 2017 and was completed in May 2021 with a total of 1550 subjects randomized. Based on actual accrual and the decision to eliminate pCR as a co-primary endpoint, the power to detect a hazard ratio of 0.70 attributed to the addition of atezolizumab was recalculated, assuming a lost-to-follow-up rate of 0.00083 per month, using the actual accrual pattern for the power calculation. With 1550 subjects accrued in 42 months, an additional 22 months follow up would be sufficient to obtain 252 EFS events under the assumptions stated above, which will provide 80% power to detect a HR of 0.7 between the atezolizumab and the placebo arm at an overall 2-sided alpha level of 0.05.

4. General Analysis Definitions

Data will be analyzed using SAS (Version 9.4 or higher).

Descriptive statistics will be tabulated as follows:

- Categorical data will be summarized in contingency tables presenting frequencies and percentages.
- Continuous data will be summarized using number of non-missing values (n), mean, standard deviation, median, minimum, and maximum values.

Baseline tables will be presented by treatment arm and overall. Tables containing post-randomization information will be created by treatment arm, unless otherwise specified.

In addition to the tables combining all data, the tables showing baseline characteristics and the results of the primary efficacy endpoint analyses will be presented by group: NSABP and GBG. This is for descriptive purposes only.

Subject listings containing subject-level data will be provided for all data presented in the tables, where noted. The listings will be presented by treatment group.

4.1 Study Period Definitions

4.1.1 Treatment Periods

The treatment periods include:

1. Neoadjuvant Treatment Period:

[redacted]

[redacted]

2. Adjuvant Treatment Period (only applicable to patients who received at least one dose of adjuvant Placebo/Atezolizumab):

[redacted]

3. Paclitaxel (WP) + Carboplatin + Placebo/Atezolizumab Treatment period

[redacted]

4. AC/EC + Placebo/Atezolizumab Treatment period:

[redacted]

Unscheduled/Unknown visits:

A study assessment record with "Unscheduled" visit label, or a record without any visit label, will be categorized to a treatment period, based on visit date if visit date is available, for summary analysis. If the visit date of a particular unscheduled visit for a subject is not available, then that record will be categorized to Unscheduled/Unknown visit for summary analysis.

Table 1 Treatment Periods

Period	Start date	End date
Period 1:<br>Neoadjuvant Treatment Period	Date of first dose of WP or Carboplatin or AC or EC treatment or preoperative Placebo/Atezolizumab, whichever occurs the earliest.	**If surgery is given**:<br>Date of surgery – 1 day<br>**Else**:<br>Date of last administered dose of any of WP or Carboplatin or AC/EC or preoperative Placebo/Atezolizumab +30 days, consent withdrawal, or date of death, in case it occurs earlier.
Period 2:<br>Adjuvant Treatment Period - Applicable only for patients who received Adjuvant treatment	Date of surgery	Date of last administered dose of postoperative Placebo/Atezolizumab + 30 days, consent withdrawal, or date of death, in case it occurs earlier.
Period 3:	Date of first dose of WP or Carboplatin or preoperative	**For subjects entering the AC/EC period:**

[redacted]

Paclitaxel (WP) + Carboplatin + Placebo/Atezolizumab treatment period	Placebo/Atezolizumab, whichever occurs the earliest.	Date of start of AC/EC period – 1 day.<br>**For the other subjects:**<br>If surgery is given:<br>Date of surgery – 1 day<br>Else:<br>Date of last administered dose of any of WP or Carboplatin or preoperative Placebo/Atezolizumab +30 days, consent withdrawal, or date of death, in case it occurs earlier.
Period 4:<br>AC/EC + Placebo/Atezolizumab treatment period	Date of first dose of AC or EC treatment	**If surgery is given**:<br>Date of surgery – 1 day<br>**Else**:<br>Date of last administered dose of AC/EC or preoperative Placebo/Atezolizumab + 30 days, consent withdrawal, or date of death, in case it occurs earlier.

For study assessments that assessment dates are not collected, such as adverse events, assessment periods will be determined by the visit label on CRFs. Otherwise, the date of assessment will be used to determine the treatment period.

4.2 Planned Analyses

4.2.1 Interim EFS Analysis

One interim analysis will be performed for EFS when 190 events are observed. To maintain the experiment-wise alpha level at 0.05, O'Brien Fleming spending will be used accounting for the proportion of the total number of expected EFS events observed at the interim analysis. At 75.4% information time (190 events), the interim analysis would be performed at an alpha level of 0.0197. If the superiority boundary for EFS is crossed at the interim analysis, we will perform an interim analysis of OS and other secondary endpoints. The details are described in section 9.2. If the trial is not stopped early for efficacy, EFS and the other time to event endpoints will be analyzed when the 252 EFS events are reached, as described in Section 4.2.2.

4.2.2 Definitive EFS Analysis

The definitive analysis of EFS will be performed when 252 EFS events are recorded (irrespectively of the results of the interim analysis of EFS). It is estimated that this would occur about 64 months after accrual is initiated. All remaining time-to event efficacy endpoints will be analyzed at this time.

Table 2 Projected Interim and Definitive EFS Analyses Characteristics

Analysis	No. of events	% Information	Event to Patient Ratio	Projected Cutoff Date [a]	Projected MDD[b]	Projected Boundary (p-value) [c]

[redacted]

Interim	~190	75%	12.3%	Month 55	HR ≤0.713	p ≤0.0197
Final	~252	100%	16.3%	Month 64	HR ≤0.776	p ≤0.0441

HR=hazard ratio; MDD=minimal detectable difference.

Note: Assumes lost-to-follow-up rate of 0.00083 per month for EFS analyses and assumes monthly hazard rates: 0.00460 during year 1, 0.01020 during year 2, 0.00440 during year 3, 0.00186 during year 4 and 0.00200 for year 5 and all subsequent years.

[a] Study month at which required number of events are projected to occur, where Study Month 1 is the month the first subject is enrolled. Analysis results will be available after data cleaning.

[b] The largest observed HR that is projected to be statistically significant.

[c] The projected boundary for statistical significance for the number of events shown (actual boundary to be calculated at time of analysis based on actual number of events).

4.3 Definition of Populations

4.3.1 Intention-To-Treat (ITT) Population

The Intention-to-Treat (ITT) Population is defined as all randomized subjects. It will be used for analyses of inclusion and exclusion criteria, demographics and baseline characteristics, medical history, prior and concomitant medications, surgery, protocol deviation, death summaries, and the primary, secondary, and exploratory endpoints (except for toxicity).
Subject data will be analyzed according to their randomly assigned treatment regardless of treatment actually received.

4.3.2 Safety (SAF) Population

The safety population (SAF) is defined as all subjects who receive at least one dose of study therapy. It will be used for the toxicity endpoints, extent to exposure, adverse events, clinical laboratory tests, and other safety data summaries.

Subjects will be analyzed according to treatment actually received. Thus, any subjects receiving at least one dose of atezolizumab will be comprised to the 'Chemo/Atezolizumab' group.

4.3.3 Cardiac Safety Lead-in (CSAF) Population

The Cardiac Safety Lead-in Population (CSAF) is a sub-set of Safety Population. It includes the first 60 subjects randomized who are included in the Cardiac Safety Lead-in Study involving the collection of troponin-T levels and one extra LVEF assessment.

4.3.4 Atezolizumab/Placebo Only (APO) Population

The APO Population is a sub-set of Safety Population. It includes subjects having received adjuvant atezolizumab/placebo but did not receive capecitabine.

4.3.5 Atezolizumab/Placebo Plus Capecitabine (APC) Population

The APC Population is a sub-set of Safety Population. It includes subjects having received adjuvant atezolizumab/placebo plus capecitabine.

4.4 Treatment Assignment and Treatment Arms

Eligible triple-negative breast cancer subjects are randomized to receive either

[redacted]

1) paclitaxel/carboplatin/placebo followed by doxorubicin/cyclophosphamide/ placebo or epirubicin/cyclophosphamide/placebo or

2) paclitaxel/carboplatin/atezolizumab followed by doxorubicin/cyclophosphamide/ atezolizumab or epirubicin/cyclophosphamide/atezolizumab.

Randomization is accomplished using stratified permuted blocks. The 5 stratification factors include:

1) Group (NSABP; GBG)

2) Nodal status as determined by protocol-specified criteria (negative; positive)

- Nodal status (positive/negative) as specified in the protocol will be defined using the following criteria (at baseline and throughout study):

  Ipsilateral axillary lymph nodes must be evaluated by imaging (ultrasound or MRI) within 42 days prior to study entry. If suspicious or abnormal, FNA or core biopsy is recommended. Findings of these evaluations will be used to define the nodal status prior to study entry according to the following criteria:

Nodal status – negative

- Imaging of the axilla is negative.
- Imaging is suspicious or abnormal but the FNA or core biopsy of the questionable node(s) on imaging is negative.

Nodal status – positive

- FNA or core biopsy of the node(s) is cytologically or histologically suspicious or positive.
- Imaging is suspicious or abnormal but FNA or core biopsy was not performed.

3) AC/EC (every 2 weeks; every 3 weeks)

4) Clinical size of the primary tumor (1.1-3.0 cm; > 3.0 cm)

- Clinical size of primary tumor (1.1-3.0cm, >3cm) as determined by protocol-specified criteria is defined as the first available measurement from the following methods (highest priority listed first):
  - MRI
  - Ultrasound
  - Mammography
  - Palpation

5) PD-L1 status (Positive, Negative/Indeterminate/Not Available combined [subject randomized before the protocol amendment of July 2019 will be included as the "Not Available" category]).

4.5 Calculated Variables

- Study Day-1 is defined as the day of randomization.
- Treatment initiation date is defined as: 1) the first day of any study treatment for patients who started any treatment; 2) missing for patients who never started any treatment.
- Calculation of Study Day of assessment:

  If Assessment Date < Randomization Date, then

[redacted]

Study Day = Assessment Date - Randomization Date;
If Assessment Date ≥ Randomization Date, then
Study Day = Assessment Date - Randomization Date + 1.

- The baseline is defined as the latest assessment done before or on randomization.
- Body Mass Index (BMI) = weight (kg) / [height (m)]^2
- BSA (m^2) = {[height (cm) × weight (kg)] / 3600}$^{0.5}$
- Months = days/30.4375
- Observed follow-up time (months): *(Date of death, or last visit if known to be alive) on study – Date of randomization + 1)/30.4375*

4.6 Rules for Missing or Partial Dates

In general, missing or partially missing dates will not be imputed for safety and medical history data. However, assumptions for missing or partially missing dates for important variables will be made to allow inclusion of appropriate data records in the analyses. In general, the assumptions about the missing or partially missing dates, when needed, will be made conservative to avoid overestimation of treatment effects and underestimation of adverse effects.

- For the assignment of adverse events of special interest (AESIs) to a study treatment period the following rules will be applied in case of missing/incomplete start date: If end date is before the date of first treatment administration, the AESI will not be assigned to a treatment period.
- If start date is missing and end date is after or on the date of first treatment administration or the end date is missing, the start date will be imputed by the date of first treatment administration.
- For partial dates:
- 1) AE onset day missing: a) If year and month of AE onset date are same as first study treatment date, then use first study treatment day to impute; b) otherwise use 1st day of the month to impute;
- 2) AE onset month and year missing: a) If AE onset year is same as first study treatment year, then use first study treatment month and day to impute; b) otherwise, use January 1st to impute;
- 3) AE end day missing: a) If year and month of AE end date is same as the last study treatment date, then use last study treatment day to impute; b) otherwise use last day of the month to impute;
- 4) AE end month and year missing: a) If AE end year are same as last study treatment year, then use last study treatment month and day to impute; b) otherwise use December 31st to impute.

The imputed dates above will only be used for assigning AESIs to a treatment period and imputing missing day in partial medication start date using Day 1 can be applied for COVID-19 vaccine, but these imputed rules will not be used in any other calculation. They will not be listed as such (only original partial date will be listed).

In addition, for partial efficacy event dates, we imputed partial day by Day 15 of the month if only event day is missing, but no earlier than randomization date. If other imputation is

[redacted]

necessary based on relevant data, NSABP/GBG has the discretion to choose proper imputation rules for analysis.

4.7 Methods to Be Used for Handling Missing Data

No imputations will be made in case of missing data. However, if subjects do not have an evaluable sample for pCR, they will be considered as non-responders (non-pCR) instead of missing. This applies to all pCR endpoints.

4.8 Changes to Analysis Specified in Protocol

There are no changes to analysis specified in protocol.

5. Study Subjects

5.1 Disposition of Subjects

The number of subjects in each population will be tabulated by treatment arm and overall. Number of screened and screen-failed will be tabulated. Reasons for screen failure will be presented.

The frequency of subjects treated, subjects discontinued the study treatment (on the safety population) and of subjects terminated the study (on the ITT population) will be presented. The primary reasons for discontinuation of the study treatment and terminating the study will be summarized by treatment arm.

Study termination is defined as any subject who has died, lost to follow-up, or withdrawal of consent.

The details of the subject's or investigator's decision will be included in a listing.

5.2 Protocol Deviations

Protocol deviations classified as major will be determined before database lock. The major protocol deviations will be categorized by type and summarized by treatment arm.

A listing of all major protocol deviations will be provided by treatment arm.

5.3 Inclusion and Exclusion Criteria

Enrolled subjects who violate inclusion/exclusion entry criteria will be summarized for the ITT Population.

A listing of all inclusion and exclusion criteria not met will be provided by treatment arm.

6. Demographic and Other Baseline Characteristics

Descriptive statistics with respect to subject characteristics at baseline will be displayed for the ITT population by group (NSABP and GBG) and overall by treatment arm and total. Data will also be displayed for the Safety Population by treatment arm. Stratification information will be tabulated also.

The variables to be summarized are:

[redacted]

The summary of demographic and baseline characteristics data will be present the following variables:

- Age at Screening (years)
- Race
- Ethnicity
- Sex
- Height (cm)
- Weight (kg)
- BMI (kg/m^2)
- Time since diagnosis of primary invasive breast cancer at randomization
- Histological tumor type (Invasive Ductal Carcinoma, Invasive lobular carcinoma or mixed lobular carcinoma, other)
- Tumor grade
- ER
- PgR
- HER2
- Germline Mutation Status (gBRCA1, gBRCA2)
- Tumor site (unilateral bilateral)
- Tumor type (unifocal, multifocal, multicentric)
- Tumor size (longest diameter in mm)
- cT
- Core- or fine needle biopsy of lymph node result
- Are there any palpable regional lymph nodes? (If yes, Axillary, internal mammary, intraclavicular, or supraclavicular)
- cN
- ECOG Performance Status
- LVEF method and result
- PD-L1 status
- Stromal TILs (continuous, categories <10%, >=10% and <30%, >=30%)

A subject listing for demographic and other baseline characteristics data will be provided for the ITT Population.

7. Medical History

Medical histories will be classified according to the terminology of CTCAE v4.0 and coded using MedDRA version 21.0. Medical histories will be tabulated by system organ class and preferred term for the ITT population by treatment arm and total.

8. Concomitant Medications

Medications at screening and at any time post-baseline will be tabulated by medication category for ITT Population by treatment arm and total.

Medication data will also be presented in a listing.

9. Efficacy Evaluation

The primary efficacy analyses will be performed on the ITT population.

[redacted]

9.1 Primary Efficacy Analyses

9.1.1 Event-Free Survival (EFS)

Endpoint: EFS is defined as time from randomization until event: EFS events are progression on protocol therapy resulting in administration of non-protocol cancer therapy or inoperability, local invasive recurrence following mastectomy, local invasive recurrence in the ipsilateral breast following lumpectomy, regional recurrence, distant recurrence, contralateral invasive breast cancer, second non-breast primary cancer (excluding squamous or basal cell carcinoma of the skin), or death from any cause prior to recurrence or second primary cancer. Some US patients who are still on the neoadjuvant period of the study may opt to stop the protocol therapy without disease progression and take pembrolizumab instead. These patients will NOT be considered to have experienced EFS events.

Subjects without an event will be censored on the date on which they are last known to be alive and event-free, on or before the clinical cut-off date for the analysis. Subjects with no post-baseline information will be censored at the date of randomization. Subjects who were determined ineligible post randomization due to metastatic disease or second non-breast primary cancer will be censored at the date of randomization.

EFS is calculated as follows:

EFS (months) = (Date of event/censor - Date of randomization + 1)/30.4375

The comparison of EFS between the two treatment arms will be assessed using the stratified log-rank test incorporating the stratification factors used in the randomization. The definitive analysis will be performed when 252 EFS events are recorded. Due to the potential risk of over-stratification, if at least one stratum has fewer than four EFS events at the interim analysis or the primary analysis, the stratification factor that contains the level with the smallest number of patients will be removed from the stratified analyses. If there still exists a stratum with fewer than four EFS events, a second stratification factor that contains the level with the second smallest number of patients will also be removed from the stratified analyses. If necessary, at most two stratification factors will be considered for removal, the collapse of at most two stratification factors, to avoid losing statistical power while reducing over-stratification. The same stratification factors will be applied to the main analyses of all endpoints where stratified analyses are planned. In addition, unstratified log-rank test will also be provided.

There is one interim analysis for EFS efficacy performed when approximately 190 EFS events have occurred. To maintain the experiment-wise alpha level at 0.05, the O'Brien Fleming spending will be used accounting for the proportion of the total number of expected EFS events observed at the interim analysis. The nominal significance level to be used at the interim and final analysis will be calculated using this function at the time of the interim analysis on the basis of the actual proportion of EFS events available at that time. The hazard ratio (HR) for EFS will be estimated using a stratified Cox proportional hazards model with the same stratification factors used for the stratified log-rank test. Two-sided 95% confidence intervals for the hazard ratio will be reported.

Due to clinical considerations (such as delayed occurrence of events preventing timely read-out of the study) and the dynamic nature of data closure and event confirmation, we may perform the definitive analysis when at least 242 EFS events are recorded. That gives

[redacted]

us at least 77% power to detect a hazard ratio of 0.7 between the atezolizumab and the placebo arm at an overall 2-sided alpha level of 0.05.

Example SAS code:

```
PROC LIFETEST DATA = data method=km plots=(survival) outtest=results
conftype=LOGLOG;
    TIME survtime*censor(1);
    STRATA group nodal ACsched size PDL1 / test=logrank group=trt01pn;
RUN;
```

where `group`, `nodal`, `ACsched`, `size`, and `PDL1` are the randomization strata.

9.1.1.1 *Descriptive Analyses*

The Kaplan-Meier (K-M) estimates for EFS will also be calculated separately for subjects in the different treatment arms. The K-M curves will be drawn and a summary of the events/censored will be presented by treatment arm in a table. The K-M estimates for 2-, 3- and 4-year EFS and their 95% CIs will be presented. The two-sided log-log type confidence intervals for these parameters will be used.

A listing of all EFS events will also be provided.

9.1.1.2 *Subgroup Analysis*

The generalizability of the results from the primary analysis will be explored by estimating the treatment effect in subgroups. The specific subgroups that will be investigated, based on their actual values, are:

- Group (NSABP Foundation, Inc., GBG)
- Clinical Size of Primary Tumor (1.1-3.0 cm; > 3.0 cm)
- Clinical Nodal Status (negative, positive)
- PD-L1 status
- AC/EC Schedule (every 3 weeks; every 2 weeks)
- TILs with cut-off 10% and 30%
- Gene mutation status: deleterious gBRCA mutation, no reported deleterious gBRCA mutation, and unknowns. Deleterious gBRCA mutation is defined as randomized subjects who have "deleterious mutation" identified during screening and at other visits in at least one of the following: BRCA1, BRCA2. Patients with "variant of unknown significance" reported for BRCA1 or BRCA2 will be included in those without reported deleterious gBRCA mutation.

Potential interaction between treatment and each factor will be explored by Cox proportional hazard (PH) models. Unstratified HRs estimated from Cox proportional models and their corresponding 95% CIs will be calculated and presented by forest plots. Kaplan-Meier estimates will also be produced for each level of the categorical variables.

9.1.1.3 *Sensitivity Analysis 1*

The primary analysis will be repeated but the levels of the actual levels of the stratification factors, except the retrospective PD-L1 status for patients randomized prior to PD-L1 being a stratification factor, are used rather than the levels provided at the time of randomization, whenever those differed.

[redacted]

9.1.1.4 ***Sensitivity Analysis 2***

The primary analysis will be repeated but the levels of the actual levels of the stratification factors, including the retrospective PD-L1 status for patients randomized prior to PD-L1 being a stratification factor, are used rather than the levels provided at the time of randomization, whenever those differed.

9.1.1.5 ***Sensitivity Analysis 3***

The primary analysis will be repeated in patients who entered the study before the end of July 2020 to assess the effect of the approval of pembrolizumab in the US in July of 2021.

9.1.1.6 ***Exploratory Analysis***

Stratified Cox proportional hazards (PH) models based on actual values of stratification factors will be used to compare EFS when adjusting for potential confounders such as age at study entry, race, and tumor grade. Potential interactions between treatment and clinical factors will also be assessed using Cox models. The Cox PH analyses are considered exploratory.

BRCA mutation status, another potential confounder, was not required for entry, though it was commonly obtained as part of routine care for the study patient population thus. It was collected when available but was not available in all subjects. As a sensitivity analysis, we will repeat the above analysis, with the addition of BRCA mutation status in the list of potential confounders in subjects with BRCA mutation status information.

Stromal TILs are associated with a patient's immune response and is a potential prognostic factor. In an exploratory analysis, we will study the association between baseline percentages of TILs (in categories: <10%, >=10% and <30%, >=30%) and EFS in stratified Cox PH models with potential confounders such as age, race and tumor grade adjusted.

All EFS analyses above will be done at interim and definitive EFS analyses.

9.2 Secondary Efficacy Analyses

To control the overall type I error rate at a two-sided significance level α=0.05, a hierarchical fixed sequence testing procedure will be used. The type I error will be recycled to formally compare the key secondary endpoint, OS, between the two treatment arms only if the primary EFS analysis reaches a significant result. If the test of EFS is not statistically significant, OS will not be formally tested, and we will not claim statistical significance for OS. A group-sequential design (Lan-DeMets with O'Brien-Fleming stopping boundaries) will be used to control the overall type I error rate (Lan and DeMets 1983) across the interim and final OS analyses.

One planned interim analysis for OS will be performed when the EFS endpoint crosses the boundary (either at interim or final analysis of EFS). The final analysis of OS will be performed by November 2027 or when 300 deaths are observed, whichever happens first.

The following supportive secondary efficacy analyses will be performed following the EFS/OS analyses:

- Pathologic complete response (pCR) in the breast and lymph nodes (ypT0/Tis ypN0)
- Disease-Free Survival (DFS)
- Distant Disease-Free Survival (DDFS)

[redacted]

9.2.1 Overall Survival (OS)

Endpoint: Defined as time from randomization until death from any cause.

The overall survival is defined as the time from randomization to death from any cause. The subjects who are still alive will be censored as follows:

- at the date of subject's first record of withdrawal of consent to study participation, and
- at the date of last confirmed contact in the other cases.

The overall survival will be analyzed using the Kaplan-Meier (K-M) method. The K-M curves will be drawn and a summary of the events/censored will be presented by treatment arm. K-M estimates of 2-, 3- and 4-year OS and their corresponding 95% CIs will be presented s. The stratified log-rank test p-value incorporating the stratification factors used in the primary analysis of the EFS endpoint will be calculated.

OS will also be analyzed using Cox PH models as described in 9.1.1.

9.2.2 pCR in Breast and Lymph Nodes (ypT0/Tis ypN0)

Endpoint: Defined as the absence of any invasive component in the resected breast specimen and all resected lymph nodes following completion of neoadjuvant therapy (ypT0/Tis ypN0).

A subject is considered as demonstrating a pathologic complete response in breast and nodes following neoadjuvant therapy if there is no histological evidence of invasive tumor cells found in the surgical breast specimen and none found in any of the resected lymph nodes. For patients with bilateral breast cancer, if both the indicator breast tumor and the contralateral breast tumor are triple-negative, the patients are considered as a pathologic complete response only if response occur in both breasts. For a bilateral breast cancer patient whose contralateral breast tumor is not triple-negative, her pCR status is the response status of the indicator breast tumor. A subject without an evaluable sample (including but not limited to subject who did not have surgery, has withdrawn from study participation before reaching surgery, or has a missing value for tumor response for any reason), or has ypT0/Tis and ypN0 but has lymphovascular or vascular invasion, will be considered as a non-responder (non-pCR).

The number of subjects demonstrating a pathologic complete response in breast and nodes will be displayed, and the pathologic complete response rate will be calculated along with the two-sided 95% confidence interval. Additionally, the difference in pCR rates between treatment arms and the two-sided 95% confidence interval will also be presented using the normal approximation to the binomial distribution.

The difference between treatment groups in the proportion of subjects with pCR will be assessed using the Cochran-Mantel-Haenszel test with a two-sided alpha level of 0.05. The test will be stratified for the stratification factors.

Example SAS code:

```
PROC FREQ DATA = data;
   TABLES strata*treatment*pCR / cmh;
RUN;
```


[redacted]

Where strata will be all levels/combinations of the selected stratification variables in the primary analysis of EFS, which may include up to 32combinations: Group (NSABP and GBG), Nodal Status (Positive and Negative), AC/EC Schedule (Every-2-week and Every-3-week), Size of Primary Tumor (1.1-3.0 cm and >3.0 cm), PD-L1 status (Before Amendment, Positive, and Negative/Indeterminate/Not Available)]; Treatment contains 2 level (Chemo/Atezolizumab and Chemo/ Placebo), and pCR contains 2 levels (responder and non-responder). The Mantel-Haenszel odds ratio and its 95% confidence interval will be reported.

pCR can be derived based on the Histopathology – [redacted] (ypT and ypN status and confirmation of negative responses for lymphovascular invasion, vascular invasion and perineural invasion).

9.2.2.1 **Subgroup Analysis**

Subgroup analyses will be performed to assess differences between treatments arms in the proportion of subjects with pCR for each subgroup, besides all other secondary efficacy endpoints. The specific subgroups based on their actual values that will be investigated are:

- Group (NSABP Foundation, Inc., GBG)
- Clinical Size of Primary Tumor (1.1-3.0 cm; > 3.0 cm)
- Clinical Nodal Status (negative, positive)
- PD-L1 status (negative, positive, indeterminate)
- AC/EC Schedule (every 3 weeks; every 2 weeks)
- TILs with cut-off 10% and 30%
- Gene mutation status: deleterious gBRCA mutation, no reported deleterious gBRCA mutation, and unknown.

Summaries of the difference between treatment arms in pCR rates will be produced separately for each level of the categorical variables and summaries will be presented in forest plots. Potential interaction between treatment and each factor will be explored by logistic regression models. Unstratified odds ratios (OR) estimated from regression models and their corresponding 95% CIs will be calculated and presented by forest plots. OR estimates will be produced for each level of the categorical variables.

9.2.2.2 ***Exploratory Analysis***

A multiple logistic regression model will be used to study treatment efficacy when adjusting for actual values of stratification factors and other clinical factors such as age at study entry, race, tumor grade. The associated ORs and their 95% confidence intervals will be presented.

The K-M estimates of EFS will be compared by pCR status within each treatment arm.

9.2.3 Distant Disease-Free Survival (DDFS)

Endpoint: Defined as time from randomization until distant recurrence, death from breast cancer, death from other causes, and second primary non-breast invasive cancer (with the exception of non-melanoma skin cancers and in situ carcinoma of any site).

The distant disease-free survival is defined as time from the randomization to the earliest of the following:

[redacted]

(i) date of relapse confirmation of distant recurrence

(ii) date of second primary non-breast invasive cancer (with the exception of non-melanoma skin cancers and in situ carcinoma of any site)

(iii) date of death from any cause

Subjects without an event will be censored to the latest date that is known to be alive during the study and event-free:

- at the date of subject withdrawal of consent, in case of consent withdrawal, and
- at the date of last confirmed contact during study.

The distribution of the distance disease-free survival will be analyzed using K-M estimation. The K-M curves will be drawn and a summary of the events/censored will be presented by treatment arm. K-M estimates of 2-, 3- and 4-year DDFS and the corresponding 95% CIs will be presented. The stratified log-rank test p-value incorporating the stratification factors used in the primary analysis of the EFS endpoint will be calculated.

DDFS will also be analyzed using Cox PH models as described in 9.1.1.

A listing of all distant cancer recurrences and new primary cancers will be provided.

9.2.3.1 **Sensitivity Analysis**

For this analysis, another definition of DDFS will be used. DDFS is defined as time from first breast surgical procedure (primary surgery) until distant recurrence or death from any cause, or second primary non-breast invasive cancer (with the exception of non-melanoma skin cancers and in situ carcinoma of any site) whichever occurs first. Patients with events prior to surgery and those without surgery will be censored on Day 1.

9.2.4 Disease-Free Survival (DFS)

Endpoint: Defined as the time from the first breast surgery to the first occurrence of disease recurrence or death from any cause.

The disease-free survival is defined as the number of months from the first breast surgery to the earliest of the following, whichever is available. Population for DFS analysis includes subjects who had surgery.

(i) date of ipsilateral invasive breast tumor recurrence
(ii) date of ipsilateral local-regional invasive breast cancer recurrence
(iii) date of distant recurrence
(iv) date of contralateral invasive breast cancer
(v) date of ipsilateral or contralateral DCIS
(vi) date of second primary non-breast invasive cancer (with the exception of non-melanoma skin cancers and in situ carcinoma of any site)
(vii) date of death attributable to any cause (or unknown cause)

Patients with distant metastasis or second primary cancer prior to surgery and those without surgery will be censored on Day 1. Subjects without an event will be censored to the latest date that subject is known to be alive during the study and event-free:

[redacted]

- at the date of subject withdrawal of consent, in case of consent withdrawal, and
- at the date of last confirmed contact during study.

The distribution of the DFS interval will be analyzed using K-M estimation. The K-M curves will be drawn and a summary of the events/censored will be presented by treatment arm. K-M estimates of 2-, 3- and 4-year EFS and the corresponding 95% CIs will be presented. The stratified log-rank test p-value incorporating the stratification factors used in the randomization will calculated.

DFS will analyzed using Cox PH models as described in 9.1.1.

9.3 Exploratory Efficacy Analyses

9.3.1 pCR in Breast (ypT0/Tis)

Endpoint: Defined as the absence of any invasive component in the resected breast specimen (nodal material not considered) following completion of neoadjuvant therapy.

The number of subjects demonstrating a pathologic complete response in breast will be displayed and the pathologic complete response rate (%) is calculated along with the two-sided 95% confidence interval. Additionally, the difference in rates between treatment arms, the associated Mantel-Haenszel odds ratio, and their two-sided 95% confidence intervals will also be calculated.

pCR in breast (ypT0/Tis) on subject level will also be displayed in a listing.

9.3.2 pCR in Breast and Lymph Nodes (ypT0/ypN0)

Endpoint: Defined as the absence of any invasive component or DCIS in the resected breast specimen and all resected lymph nodes following completion of neoadjuvant therapy.

Patients who underwent surgical axillary staging procedures prior to randomization would be deemed ineligible for pCR in breast and lymph nodes. They would be named non-responders for this endpoint.

The number of subjects demonstrating a pathologic complete response will be displayed and the pathologic complete response rate (%) will be calculated along with the two-sided 95% confidence interval. Additionally, the difference in rates between treatment arms, the associated Mantel-Haenszel odds ratio, and their two-sided 95% confidence intervals will be also calculated.

pCR in breast and lymph nodes (ypT0/ypN0) on subject level will also be displayed in a listing.

9.3.3 Positive Nodal Status Conversion Rate

Endpoint: Percentage of subjects clinically node-positive that convert to pathologically node-negative following completion of neoadjuvant chemotherapy.

The number of subjects demonstrating positive nodal status conversion will be displayed and the conversion rate (%) will be calculated along with the two-sided 95% confidence interval. Additionally, the difference in rates between treatment arms, the associated

[redacted]

Mantel-Haenszel odds ratio, and their two-sided 95% confidence intervals will also be calculated.

The positive nodal status conversion on subject level will also be displayed in a listing.

9.3.4 Recurrence-Free Interval (RFI)

Endpoint: Defined as time from randomization until invasive local, regional, or distant recurrence, or death from breast cancer (censored for death from other causes).

The recurrence-free interval is defined as the number of months from the randomization to the earliest of the following:

(i) date of relapse confirmation of first local-regional recurrence or first distant recurrence

(ii) date of death from breast cancer

Patients with distant metastasis prior to surgery and those without surgery will be censored on Day 1. Subjects who die of other cause than breast cancer will be censored at the time of death. Deaths due to other causes is not considered a competing risk in this setting since the rate of events of deaths due to other causes is generally low.

Subjects without an event will be censored to the latest date that is known to be alive during the study and event free:

- at the date of subject withdrawal of consent, in case of consent withdrawal, and
- at the date of last confirmed contact during study.

The distribution of the recurrence-free interval will be analyzed using K-M estimation. The K-M curves will be drawn and a summary of the events/censored subjects will be presented by treatment group. K-M estimates of proportions of recurrence-free at 2, 3, and 4 years and the corresponding 95% CIs and will be presented. The stratified log-rank test p-value incorporating the stratification factors used in the primary analysis of the EFS endpoint will be calculated.

RFI will also be analyzed using Cox-PH models with adjustment for the factor described in the exploratory analysis of the primary endpoint under 9.1.1.

A listing of all cancer recurrences will be provided.

9.3.4.1 Sensitivity Analysis

For this analysis, another start date for the calculation of RFI will be used. RFI is defined as time from the date of the primary breast surgery until invasive local, regional, or distant recurrence, or death from breast cancer (censored for death from other causes) whichever occurs first. Patients with distant metastasis prior to surgery and those without surgery will be censored on Day 1.

9.3.5 Brain-Metastases-Free Survival (BMFS)

Endpoint: Defined as time from randomization until documentation of first brain metastasis (coded "CNS metastases") or death from any cause. Subjects who develop locoregional

[redacted]

or distant recurrence outside of the central nervous system (CNS) as first event will be continuously to be followed for subsequent development of brain metastases.

The brain-metastases-free survival is defined as the number of months from randomization to the earliest of the following:

- brain metastasis
- death from any cause

Ineligible patients with brain metastasis confirmed post randomization will be censored on the randomization day. Subjects without an event will be censored to the latest date that subject is known to be alive during the study and event free:

- at the date of subject withdrawal of consent, in case of consent withdrawal, and
- at the date of last confirmed contact during study.

The distribution of the brain metastases free survival will be analyzed using K-M estimation. The K-M curves will be drawn and a summary of the events/censored will be presented by treatment arm. The stratified log-rank test p-value incorporating the stratification factors used in the primary analysis of the EFS endpoint will be calculated.

Brain-metastases-free survival will also be analyzed using Cox PH models as described in 9.1.1.

9.4 Correlative Science Analyses

Molecular and genetic correlates of response will be explored by NSABP and GBG out of the scope of this SAP.

We will study the association between the values of Tumor-infiltrating lymphocytes (TILs) at various occasions and clinical outcomes, including pCR of breast and nodes and EFS.

10. Surgery

The parameters related to the surgery will be tabulated for the ITT population:

- Was primary surgery performed?
- Was surgery performed within 6 weeks after last chemotherapy?
- Did the subject receive non-protocol anti-cancer therapy before surgery?
- Were additional surgeries performed? If Yes, Number of additional surgeries.
- Was a complete resection (R0) achieved?
- Most radical surgery type
- Lymphadenectomy after chemotherapy
- Prophylactic contralateral mastectomy

The histopathology of the surgery will also be tabulated:

- Bilateral Breast Cancer (No/Yes)
- Indicator Location (Left/Right)
- ypT (Neoadjuvant Pathologic T stage)
- ypN (Neoadjuvant Pathologic N stage)

[redacted]

- Lymph Nodes at Surgery (Positive/Negative)
- Was sentinel node biopsy performed? (Y/N) If yes, Number of positive sentinel nodes, Number of lymph nodes dissected for SNB
- Was axilla clearance performed? (Y/N) If yes, Number of positive nodes, Number of lymph nodes dissected
- Local R status
- Closest margin (mm)
- Was there residual disease? (Y/N)
- Invasive residual disease
- Non-invasive residual disease
- Post Therapeutic Grading (1, 2, 3)
- Lymphovascular invasion (only extra tumoral)
- Vascular invasion (Y/N)
- Perineural invasion (Y/N)
- HER2 status (Positive/Negative)
- Estrogen receptor status (Positive/Negative)
- Progesterone receptor status (Positive/Negative)

Additional surgical details, such as reasons, will be displayed in a listing:

- Reason surgery was not performed within 6 weeks after last chemotherapy
- Reason subject received non-protocol chemotherapy before surgery

11. Safety Evaluation

The safety analyses will be presented for the Safety Population.

11.1 Extent of Exposure

The exposure to each study drug and combinations will be summarized in terms of:

- total number of administered infusions,
- total duration of treatment (weeks),
- number of subjects with at least one dose delay, dose reduction (except for atezolizumab/Placebo), and dose interruption (zero total dose administered), and
- reasons for dose reduction (except for atezolizumab/Placebo), dose delay and dose interruption

for the following treatments:

- Paclitaxel/Nab-Paclitaxel,
- Carboplatin,
- Doxorubicin/Cyclophosphamide (AC),
- Epirubicin/Cyclophosphamide (EC),
- Atezolizumab/Placebo (by treatment period).

Total administrated doses (mg) or number of administrated doses and total duration of treatment (weeks) will be summarized for the following treatments:

- Olaparib (alternative to Capecitabine in subjects with germline BRCA1 or BRCA2 mutations), and

[redacted]

- Pembrolizumab.

For capecitabine, we will record whether a patient received capecitabine or not; if started, the starting dose level, the total number of cycles received and the starting cycle.

Total duration of treatment (weeks) will be calculated as follows:
(Date of last drug administration - date of first drug administration + number of days to the end of the cycle)/ 7, without taking into account any interruption.

The number of days to the end of each treatment cycle is listed as follows:

- paclitaxel = 7 days
- carboplatin = 21 days
- AC/EC = 14 days for q2w, 21 days for q3w
- atezolizumab/placebo = 21 days

In addition, the dose intensity of paclitaxel in mg/m²/week, carboplatin in AUC/cycle, AC/EC in mg/m²/week, and of atezolizumab (by treatment period Neoadjuvant and Adjuvant and total) in mg/week will be tabulated by treatment arm.

The dose intensities will be calculated as follows:

For paclitaxel, doxorubicin (A), epirubicin (E), cyclophosphamide (C):

- The cumulative dose per subject will be calculated by summing up all actual doses administered (mg/m²).
- The dose intensity (mg/m²/week) over the total duration the subject was treated will be calculated as follows:

  Cumulative dose (mg/m²) / [(date of last drug administration - date of first drug administration + number of days to the end of the cycle) / 7].
- The relative total dose intensity will be calculated as follows:

  *the dose intensity (mg/*m²/week) over the planned dose intensity (mg/m²/week)

For carboplatin:

- The cumulative dose (AUC) per subject will be calculated by summing up all scheduled doses (AUC) of this study drug administered.
- The dose intensity (AUC/cycle) over the total duration the subject was treated is defined as follows:
  the cumulative dose / ((date of last drug administration - date of first drug administration + 21) /21).
- The relative total dose intensity will be calculated as follows:
  *the dose intensity (*AUC/cycle) over the planned dose intensity (5 AUC/cycle)

For atezolizumab (by treatment period):

- The cumulative dose (mg) per subject will be calculated by adding all doses (mg) of this study drug administered to the subject by treatment period.
- The dose intensity (mg/week) over the total duration the subject was treated is defined as follows:

  the cumulative dose / ((date of last drug administration - date of first drug administration + 21) /7).

For olaprarib and pembrolizumab:

[redacted]

- The cumulative dose (mg) per subject will be calculated by adding all doses (mg) of this study drug administered.
- The dose intensity (mg/day for Olaparib and mg/week for pembrolizumab) over the total duration the subject was treated is defined as follows:

  the cumulative dose / ((date of last drug administration - date of first drug administration + 1)) for olaparib and *the cumulative dose/((date of last drug administration - date of first drug administration +21) /7)* for *pembrolizumab*.

To better explain the definition of relative total dose intensity (RTDI) and its calculation, some more details are provided below.

RTDI is the dose intensity achieved by a patient relative to intended dose intensity based on the planned schedule of chemotherapy. RTDI will be calculated according to the following step-by-step algorithm.

Planned dose (PnD) is the amount of a drug planned to be given in a cycle/week. PnD is calculated separately for each component of a therapy. Planned total dose (PTD) is the planned cumulative dose over the entire treatment duration; i.e. if a patient was planned to receive n cycles/weeks of drug, then sum the total amount of drug planned during those n cycles/weeks.

i.e. $$PTD(\text{mg}) = \sum_{i=1}^{n} PnD_i$$

Planned total dose intensity (PTDI) is the planned average dose intensity over the entire treatment duration.

i.e. $$PTDI(\text{mg/week}) = \frac{PTD}{planned\ \ duration\ \ of\ \ therapy\,(weeks)}$$

Actual dose (AD) is the total amount of drug that the patient has received over one cycle/week. Actual total dose (ATD) is the cumulative doses of the drug that have been given over the treatment duration of n cycles/weeks.

i.e. $$ATD\,(\text{mg}) = \sum_{i=1}^{n} AD_i$$

Actual total dose intensity (ATDI) is defined as the dose intensity of what has actually been administered over the n cycles/weeks, calculated as actual total dose divided by the treatment duration in weeks.

i.e. $$ATDI(\text{mg/week}) = \frac{ATD}{duration\ of\ \ therapy\,(weeks)}$$

For those patients who discontinued treatment due to death, progression, or relapse, the planned total dose intensity as well as planned total dose and actual total dose intensity will be calculated based on the actual duration of treatment in weeks.

[redacted]

For all other patients who discontinued chemotherapy prematurely, including those due to toxicity, the remaining cycles/weeks will be considered to be a zero dose without delay, and the PTD and denominator of ATDI will be as initially planned.

The last cycle/week (the regular one or the last one before the withdrawal) is always assumed to have the planned length, the treatment end date is the last day of the last cycle/week. If a patient has not received some drug at all, ATD and ATDI for this drug is considered to be 0.

Relative Total Dose Intensity (RTDI) is the ratio of ATDI and PTDI, expressed as a percentage.

i.e. $$RTDI(\%) = \frac{ATDI}{PTDI} \times 100$$

Note that RTDI expresses the effect of reductions, interruptions and delays as well as premature discontinuation in a treatment. Also note that planned and actual total dose intensity can be calculated by mg/m2/week for Paclitaxel, AUC/cycle for Carboplatin, mg/week for atezolizumab/placebo, and mg/m²/week for AC/EC.

11.2 Adverse Events

Safety tables for adjuvant toxicity will be produced by the following three sets:

- All subject in Safety Population
- Subjects having received only atezolizumab/placebo in the adjuvant setting
- Subjects having received concurrently atezolizumab/placebo and capecitabine in the adjuvant setting)

Adverse events (AEs) recorded on the AE form will be coded using the Medical Dictionary for Regulatory Activities (MedDRA Version 21.0) and will be graded according to the National Center Institute Common Terminology Criteria for AEs (NCI-CTCAE criteria [v4.0]). Uncoded terms will be presented under "SOC uncoded", with their reported term.

AEs will be summarized by Safety Population.

11.2.1 Treatment-Emergent Adverse Events (TEAEs)

Treatment-Emergent Adverse Events (TEAEs) are defined as any AEs start on or after the first study treatment. AE onset/end dates were not collected for in this study except SAEs collected [redacted]. As such all non-serious, non-AESI adverse events without an onset date will be assumed to be treatment-emergent adverse events (TEAEs).

An extension of the list of AESIs required for the label has been implemented after the initial study protocol. The list is generated by identification of all Grade 1 or greater preferred term AEs included in the AE data base and preparing a summary of the most severe grade of each term reported across all treatment cycles for each patient for review by [redacted]

[redacted]

For Grade 2 and higher AEs, reviewers will propose a single clinical diagnosis preferred term for each clinical AESI and designate which other identified AESI terms are part of the clinical diagnosis preferred term. For each proposed clinical diagnosis preferred term a new specific [redacted] has been designed to collect detailed information on date of onset, date of resolution, information on treatment with corticosteroids or non-corticosteroid immunosuppressive therapies and requirements for chronic thyroid. The site investigator will be provided opportunity to request an alternative clinical diagnosis preferred term be used on the new specific [redacted]

For patients with no Grade 2 or greater AESIs, but with certain Grade 1 AESIs that require additional data collection, as determined by a medical staff, the AESI details/summary forms will be generated for the sites to complete. Such cases that require additional details/summary forms are also referred as clinical diagnosis from medical review of AESIs.

For patients with no Grade 2 or greater AESIs, but with Grade 1 AESIs that resolve while therapy is continued, the AESI details/summary form will not be generated. The start date will be inferred to be on the first day of the treatment cycle in which the toxicities appeared and will be inferred to have resolved on the last day of the cycle in which the toxicities were reported.

Those AESIs that warrant additional data collection will be tabulated separately from the other AE tables, as they are collected independently.

11.2.1.1 Listing and tabulation of TEAEs

TEAEs will be analyzed in terms of their type, incidence, and severity.

The analyses of TEAEs will be presented by treatment arm and by treatment periods (when indicated below) based on the study period (see Section 4.4.1), as well as by overall study.

A summary table will be presented by treatment arm the number and percentage of subjects with at least one of AE in the following categories (separate tables for each of the 4 study periods described in section 4.4.1 and overall):

- AE
- Grade 3/4 AE
- Serious AE
- Cardiac AE
- AE leading to permanent discontinuation of study therapies
- AE leading to permanent discontinuation of atezolizumab/placebo
- AEs leading to death
- AESIs (as reported by the investigator)
- Immune-related AESIs (as reported by the investigator)
- AESIs leading to death
- Immune-related AESIs (as reported by the investigator) leading to death
- AESI related to study treatment
- AESIs leading to permanent discontinuation of any study therapies
- AESI leading to permanent discontinuation of atezolizumab/placebo
- Grade 2/3/4 AESIs

[redacted]

The following tabulations will be presented by SOC and PT for overall period and by neoadjuvant and adjuvant treatment periods unless specified otherwise:

- All AEs
- AEs leading to permanent discontinuation of any study therapies
- AESIs (as reported by the investigator)
- Immune-related AESIs (as reported by the investigator)
- AESIs leading to permanent discontinuation of any study therapies
- AESIs leading to permanent discontinuation of atezolizumab/placebo
- Grade 3, 4, and 5 AEs and AESIs (all, 4 study periods described in section 4.1.1)
- Grade 3, 4, and 5 AESIs as reported by the investigator (all, 4 study periods described in section 4.1.1)

In addition, tabulations will be presented by SOC, PT, and grade for all AEs and AESIs for the overall period and by neoadjuvant and adjuvant treatment periods. In case a subject experienced the same event more than once, the worst severity will be used for TEAEs by grade tabulations.

Radiation-related AEs and COVID-19 related AEs will be separately summarized by SOC and PT for overall period.

A listing of all adverse events (including AEs recorded at unscheduled visits) by treatment arm will be provided including the subject unique identifier, age, race, sex, SOC/preferred term/verbatim, severity, relationship to study treatment, and onset period (i.e., reported visit). Similarly, a listing of grade 3/4 AEs will be provided.

A listing of all AESIs by treatment arm will be provided including the subject identifier, age, race, sex, SOC/PT/verbatim, severity, relationship to study treatment, outcome, and onset period (i.e., reported visit).

11.2.1.2 Listing and tabulation of AESIs that require additional data collection

The following tabulations will be presented for the AESIs, as determined by medical review, that require additional details/summary form in the following categories (separate tables for the 4 study periods described in section 4.1.1 and overall):

- AESI
- Grade 2/3/4 AESI
- AESI related to atezolizumab/placebo
- AESI leading to death
- AESI leading to permanent discontinuation of atezolizumab/placebo
- AESI leading to treatment interruption of atezolizumab/placebo
- AESI leading to treatment with corticosteroids
- AESI leading to treatment with non-corticosteroid immunosuppressive medications
- AESI leading to ongoing treatment with chronic thyroid hormone replacement therapy
- AESI leading to ongoing treatment with chronic adrenal hormone replacement therapy
- AESI leading to ongoing treatment with chronic insulin therapy in without prior history of diabetes mellitus

[redacted]

The following tabulations will be presented by SOC and PT for the overall period, and by neoadjuvant and adjuvant periods unless specified otherwise:

- AESI
- Grade 3, 4, and 5 AESIs (all, 4 study periods described in section 4.1.1)
- AESI related to atezolizumab/placebo
- AESI leading to death
- AESI leading to permanent discontinuation of atezolizumab/placebo
- AESI leading to treatment interruption of atezolizumab/placebo
- AESI leading to treatment with corticosteroids
- AESI leading to ongoing treatment with chronic thyroid hormone replacement therapy
- AESI that is not resolved or recovered
- AESI leading to ongoing treatment with chronic adrenal hormone replacement therapy
- AESI leading to ongoing treatment with chronic insulin therapy in without prior history of diabetes mellitus

In addition, tabulations of the number of subjects who experienced AESIs that require additional data collection will be presented by system organ class (SOC), preferred term (PT) and grade. In case a subject experienced multiple AESIs within the same SOC, the worst severity will be used for AESIs by grade tabulations.

A listing of all AESIs that require additional details/summary form by treatment arm will be provided including the subject identifier, age, race, sex, SOC/PT/verbatim, severity, relationship to study treatment, outcome, and onset period (i.e., reported visit).

11.2.2 Fatal AEs and Serious Adverse Events

Serious adverse events (SAEs) and fatal AEs will be summarized by SOC and PT for TEAEs.

The following tabulations will be presented for the overall period, and by neoadjuvant and adjuvant periods unless specified otherwise:

- Serious AEs
- AEs leading to death for overall and 4 study periods described in section 4.4.1
- Serious AESIs (as reported by the investigators)
- AESIs (as reported by the investigators) leading to death for overall and 4 study periods described in section 4.4.1
- AESIs (from clinical diagnosis by medical review) leading to death for overall and 4 study periods described in section 4.4.1

Listings of SAEs and fatal AEs will be provided, similarly to the listing of all AEs.

The number of deaths will be tabulated together with the primary cause of death. The details of the 'other cause' will be included in the listing.

11.3 Clinical Laboratory Determination

Clinical safety laboratory assessments will include tests from the following parameters:

Hematological parameters:

[redacted]

- White blood cells (WBC)
- Absolute Neutrophil count (ANC)
- Hemoglobin
- Platelets

Chemistry parameters:

- AST
- ALT
- Alkaline phosphatase
- Total bilirubin
- Albumin
- Creatinine
- Calcium
- Glucose (Hypoglycemia)

Where applicable, laboratory values will be graded according to the NCI-CTCAE v4.0.

Shift tables of baseline CTCAE grade to worst post-baseline CTCAE grade will be presented separately for gradable Hematology Parameters and Chemistry Parameters by treatment arm and overall. For the purposes of tabulation, laboratory values which are not assigned NCI-CTCAE Grades 1, 2, 3, or 4 due to a toxicity will be assigned Grade 0 (within the normal range).

Potential Hy's law cases will be tabulated.

Lab listings will be provided for:

- All Hematology parameters, including but not limited to hemoglobin, neutrophils, neutrophils/leukocytes, platelets, prothrombin time, and WBC
- All Chemistry parameters, including but not limited to ALB, ALP, ALT, AST, bilirubin, calcium, cortisol, creatinine, creatinine clearance, glucose, and thyrotropin
- Coagulation parameters:
    - Prothrombin Time (PT)
    - INR
- Serology parameters:
    - Hepatitis B Surface Antigen
    - Hepatitis B Core Antibody
    - Hepatitis C Antibody
- Endocrinology parameters:
    - TSH
    - AM (morning) Cortisol
- Serum pregnancy (positive/negative)

Assessments at unscheduled visits or records without visit label will not be summarized in by visit tables but will be included in the shift tables and subject data listings.

11.4 Vital Signs, Physical Findings and Other Observations Related to Safety

[redacted]

Vital signs include systolic and diastolic blood pressure, pulse rate, weight, height, and body surface area. Summary tables will be presented by treatment arm for the Safety Population. Graphs of median (Q1-Q3) change from baseline values for each treatment over time will be generated.

The subject level values will be presented in a listing.

11.4.1 LVEF

The LVEF absolute values and the change from baseline will be summarized for the scheduled timepoints of assessment. Values after surgery will also be tabulated by category (>=50%, 45-49%, <45%).

Number and percentage of subjects with the following conditions will be summarized:

- an absolute decline >=15% from baseline, and
- an absolute decline >=10% from baseline and an absolute value below the lower limit of normal (LLN).

The absolute values and the change from baseline only count the data assessed by the same method as baseline. If several baseline assessments were done by different methods, the method most often used at post-baseline visits will be used for the analysis.

Subject level LVEF results will also be presented in a listing.

11.4.2 12-Lead ECG Testing

A summary table of findings that suggest potential cardiac disease (any and by type) at screening and at any post-baseline visit will be presented.

Subject level 12-lead ECG testing results will also be presented in a listing for those patients/visits with a finding that suggests potential cardiac disease.

11.5 Cardiac Safety Lead-in Study Endpoints

The cardiac safety lead-in study is to assess for potential augmentation of anthracycline-related cardiac toxicity with co-administration of study therapy. It is part of the secondary objectives.

Cardiac safety lead-in study endpoints will be analyzed descriptively by treatment arm for the Cardiac Safety Lead-in (CSAF) Population.

11.5.1 Troponin-T

Troponin-T elevated levels will be summarized for:

1) Immediately prior to initial dose of AC/EC following completion of carboplatin/paclitaxel co-administered with atezolizumab/placebo,
2) Immediately after administration of the 1st cycle of AC/EC prior to administration of atezolizumab/placebo,
3) Immediately after administration of the 3rd cycle of AC/EC prior to administration of atezolizumab/placebo, and
4) Maximum at post-Baseline

[redacted]

11.5.2 LVEF

LVEF results will be summarized for:

1) Baseline (prior to initiation of carboplatin/paclitaxel and atezolizumab/ placebo),
2) 3- 6 weeks after surgery,
3) Follow-up 18 months after randomization, and
4) Minimum post-baseline.

The first 60 subjects randomized are included in the cardiac safety lead-in study involving the collection of troponin-T levels and one extra LVEF assessment. Data from this population will be included in an assessment of these parameters. The proportion of subjects experiencing elevated troponin-T levels measured at baseline and after each of the 1st and 3rd cycles of AC/EC are determined and assessed to evaluate differences by treatment group. For LVEF assessment, change from baseline to before the initiation of the 3rd cycle of AC/EC and after surgery will also be determined and assessed for differences by treatment group.

The number and percentage of subjects experiencing elevated troponin-T levels measured at baseline (obtained just prior to administration of the first dose of doxorubicin or epirubicin) and after each of the 1st and 3rd cycles of AC/EC will be tabulated.

Additionally, values and change from baseline for the continuous measurement of troponin-T levels will be summarized.

Values and change from baseline for the continuous measurement of LVEF before the initiation of the 3rd cycle of AC/EC, and 3-6 weeks after surgery, follow-up 18 months after randomization, and minimum post-baseline will be summarized.

Additionally, the number and percentage of subjects whose LVEF findings at Baseline and post-baseline visits suggest potential cardiac disease will summarized by treatment arm for overall study duration.

[redacted]
