# Supplementary material for: Atezolizumab in early triple-negative breast cancer: the randomized phase 3 NSABP B-59/GeparDouze trial
Source: Nat Med. 2026 Sep 1;32(10):3801–11. doi: 10.1038/s41591-026-04565-6 (PMC13668648; doi:10.1038/s41591-026-04565-6)
Supplement: Supplementary file 2 [file 41591_2026_4565_MOESM2_ESM.pdf]

nature portfolio

Corresponding author(s): Sibylle Loibl

Last updated by author(s): Jun 30, 2026

Reporting Summary

Nature Portfolio wishes to improve the reproducibility of the work that we publish. This form provides structure for consistency and transparency in reporting. For further information on Nature Portfolio policies, see our Editorial Policies and the Editorial Policy Checklist.

Statistics

For all statistical analyses, confirm that the following items are present in the figure legend, table legend, main text, or Methods section.

n/a	Confirmed	
☐	☒	The exact sample size (*n*) for each experimental group/condition, given as a discrete number and unit of measurement
☐	☒	A statement on whether measurements were taken from distinct samples or whether the same sample was measured repeatedly
☐	☒	The statistical test(s) used AND whether they are one- or two-sided<br>*Only common tests should be described solely by name; describe more complex techniques in the Methods section.*
☐	☒	A description of all covariates tested
☐	☒	A description of any assumptions or corrections, such as tests of normality and adjustment for multiple comparisons
☐	☒	A full description of the statistical parameters including central tendency (e.g. means) or other basic estimates (e.g. regression coefficient) AND variation (e.g. standard deviation) or associated estimates of uncertainty (e.g. confidence intervals)
☐	☒	For null hypothesis testing, the test statistic (e.g. *F*, *t*, *r*) with confidence intervals, effect sizes, degrees of freedom and *P* value noted<br>*Give P values as exact values whenever suitable.*
☒	☐	For Bayesian analysis, information on the choice of priors and Markov chain Monte Carlo settings
☒	☐	For hierarchical and complex designs, identification of the appropriate level for tests and full reporting of outcomes
☐	☒	Estimates of effect sizes (e.g. Cohen's *d*, Pearson's *r*), indicating how they were calculated

Our web collection on statistics for biologists contains articles on many of the points above.

Software and code

Policy information about availability of computer code

Data collection	Data were collected in MEDCODES v6.0, and in MARVIN by Xclinical.
Data analysis	analyzes were carried out with SAS v.9.4 and R v4.5.1. Further software is specified in the method section in the manuscript:<br>TNBC6 subtyping: We developed a TNBC6 subtype classification algorithm using the gene signatures defined by Lehmann et al. (reference: see manuscript.)<br>TNBC4-IR subtyping: GSVA (v2.2.0) R package with default settings and gsankey package (v 0.0.99999). The workflow was implemented in R (v4.5.1).<br>Differential expression and Gene Set Enrichment Analysis: LIMMA (v3.50.3) in R<br>Gene Set Enrichment Analysis (GSEA): HTG gene sets and 50 hallmark (HM) gene sets of the Human Molecular Signatures Database (v2023.2) were used. The GSEA was carried out using Microsoft Windows-based software v.4.3.2.

For manuscripts utilizing custom algorithms or software that are central to the research but not yet described in published literature, software must be made available to editors and reviewers. We strongly encourage code deposition in a community repository (e.g. GitHub). See the Nature Portfolio guidelines for submitting code & software for further information.

Data

Policy information about availability of data

All manuscripts must include a data availability statement. This statement should provide the following information, where applicable:

- Accession codes, unique identifiers, or web links for publicly available datasets
- A description of any restrictions on data availability
- For clinical datasets or third party data, please ensure that the statement adheres to our policy

The data set contains multiple indirect identifiers and sensitive information, therefore the access to the data is controlled and will be shared upon request. Individual participant data that underlie the results reported in this article, after final analysis and publication of all secondary efficacy endpoints, will be shared with researchers who provide translational research proposals. Proposals should be approved by the NSABP B-59/GeparDouze Translational Committee. The study protocol and statistical report may be shared if necessary for the project. Proposal forms should be requested from trafo@gbg.de; once the application has been approved and a data transfer agreement has been signed, researchers will be given access to the data within 4 weeks. Data will be available beginning after final analysis and publication of all secondary efficacy endpoints; no end date.

Research involving human participants, their data, or biological material

Policy information about studies with human participants or human data. See also policy information about sex, gender (identity/presentation), and sexual orientation and race, ethnicity and racism.

Reporting on sex and gender	Patients were eligible for the study regardless of sex or gender, and sex was determined based on self-report at the time of enrollment. Since only one male patient was included in the study, no sex- or gender-based analyses were performed, and study results are reported for the overall cohort. Data were not disaggregated for privacy reasons.
Reporting on race, ethnicity, or other socially relevant groupings	Race and ethnicity were collected but not included in the manuscript. no race- or ethnicity-specific analyzes were performed.
Population characteristics	Baseline characteristics of the cohort (age, sex and tumorspecific characteristics) are described in Table 1 in the manuscript (p. 18).
Recruitment	Participants were recruited in 353 participating sites in USA, Germany, Canada, and Spain between December 2017 and May 2021. Eligible patients were identified consecutively by the principal investigators based on predefined inclusion and exclusion criteria. Eligible patients had clinical stage II-III early TNBC, defined as estrogen receptor (ER) and progesterone receptor (PgR) status below 1% and negative HER2 status. Central testing for HER2, ER, and PgR was required to confirm TNBC. For patients with clinical regional lymph node involvement – defined as cN1 with cytological or histological confirmation, or cN2–N3 with or without a biopsy – the primary breast tumor could be clinically staged as T1c, T2, or T3. For patients with clinically negative nodes, the primary tumor had to be T2 or T3. Bilateral or multicentric HER2-negative BC were permitted if the highest risk tumor was ER-negative, PgR-negative and met stage eligibility criteria. Patients were also required to have left ventricular ejection fraction (LVEF) >55% and no significant cardiac history. Patients were excluded if they had a history of ipsilateral invasive BC or ipsilateral ductal carcinoma in situ (DCIS), evidence of metastatic disease, prior treatment for the currently diagnosed BC before randomization, or if they had received immunotherapy previously. Patients participated voluntarily and provided written informed consent. The multicenter and multinational design of the study, involving sites across different healthcare systems and countries, migitates center-specific and regional biases on the overall study results.
Ethics oversight	The trial complied with Good Clinical Practice guidelines and Declaration of Helsinki. Study protocol and amendments were approved by the competent authorities and independent ethics committees, which are listed in Supplemental Information 1. An independent data monitoring committee oversaw the study. Patients provided written informed consent. There was no compensation for participants.

Note that full information on the approval of the study protocol must also be provided in the manuscript.

Field-specific reporting

Please select the one below that is the best fit for your research. If you are not sure, read the appropriate sections before making your selection.

☒ Life sciences ☐ Behavioural & social sciences ☐ Ecological, evolutionary & environmental sciences

For a reference copy of the document with all sections, see nature.com/documents/nr-reporting-summary-flat.pdf

Life sciences study design

All studies must disclose on these points even when the disclosure is negative.

Sample size	The sample size for this trial is based on the requirements for testing of the EFS endpoint. The hazard rates used for determination were taken from our experience in NSABP B-40 trial. The docetaxel followed by AC arm of NSABP B-40 was similar in efficacy to AC followed by weekly paclitaxel in ECOG 1199 (Sparano 2015). In NSABP B-40, the annual hazard rates were not constant and varied substantially over the first 4 to 5 years. Thus, instead of using a constant hazard rate across time, yearly-specific hazards rates were used. These rates were then reduced by

20% to account for an anticipated effect of adding carboplatin to the treatment regimen based on CALGB 40603 (Sikov SABCS 2015). To achieve monthly precision in estimates of accrual and study duration, the calculation of sample size was based on monthly hazard rates converted from each of the yearly hazard rates described above. The resulting monthly hazard rates are 0.00460 during year 1, 0.01020 during year 2, 0.00440 during year 3, 0.00186 during year 4 and 0.00200 for year 5 and all subsequent years.
The original sample size was based on having sufficient statistical power to detect a hazard ratio of 0.70 at α=0.045 assuming a lost-to-follow-up rate of 0.00083 per month. A sample size of 760 patients per arm for a total sample size of 1,520 patients followed to obtain 269 EFS events would provide a test of hypothesis with 80% statistical power at an overall alpha level of 0.045. After allowing for a ramp-up of accrual in the first eighteen months to 40 patients per month and a further ramp-up to 90 patients per month by accrual month 28, it is anticipated that the accrual rate would peak at 105 patients per month by accrual month 32. With this accrual pattern, it would take about 34 months to accrue 1,520 patients. Follow-up of an additional 30 months after completion of accrual (total study time of 64 months) would be sufficient to obtain 269 EFS events.

Data exclusions

no data were excluded.

Replication

In this clinical trial, a traditional replication of experiments is not applicable because the study is a prospective investigation in patients following a predefined treatment and follow-up protocol, rather than a laboratory or preclinical experiment that can be repeated under identical conditions. For ethical and practical reasons, the same interventions cannot simply be re-run in the same participants. Instead, the robustness of our findings is supported through the prospective design, predefined endpoints, standardized data collection procedures, and, where feasible, comparison or validation in independent patient cohorts.

Randomization

At the time of protocol activation, patients were stratified by region (North America vs. Europe), tumor size (1.1-3.0 cm vs. >3.0 cm), AC/EC schedule (q2w vs. q3w), and clinical nodal status (positive vs. negative) and then centrally randomized 1:1 using permuted block randomization to receive atezolizumab or placebo (1200 mg IV every 3 weeks).
PD-L1 status (positive, negative, indeterminate, or not available) was added as a 5th stratification factor by amendment #1 (16.07.2019) after 374 patients had been randomized. PD-L1 status assessment was done centrally using the antibody Ventana PD-L1 (SP142), the IC Score was reported in % according to the Ventana evaluation protocol. PD-L1 positive status was defined as ≥1% staining of tumor-infiltrating immune cells as a percentage of tumor area using the SP142 Ventana assay (Ventana Medical Systems, Tuscan AZ, USA).

Blinding

This is a double-blind trial; neither the patient nor the investigator/healthcare providers will know the treatment assigned.

Reporting for specific materials, systems and methods

We require information from authors about some types of materials, experimental systems and methods used in many studies. Here, indicate whether each material, system or method listed is relevant to your study. If you are not sure if a list item applies to your research, read the appropriate section before selecting a response.

Materials & experimental systems

n/a	Involved in the study	
☒	☐	Antibodies
☒	☐	Eukaryotic cell lines
☒	☐	Palaeontology and archaeology
☒	☐	Animals and other organisms
☐	☒	Clinical data
☒	☐	Dual use research of concern
☒	☐	Plants

Methods

n/a	Involved in the study	
☒	☐	ChIP-seq
☒	☐	Flow cytometry
☒	☐	MRI-based neuroimaging

Clinical data

Policy information about clinical studies
All manuscripts should comply with the ICMJE guidelines for publication of clinical research and a completed CONSORT checklist must be included with all submissions.

Clinical trial registration

NCT03281954

Study protocol

The study protocol was submitted with the manuscript.

Data collection

In this multicenter study, data were collected at oncology and breast cancer centers in Germany, the United States, Canada, and Spain. Participating sites included academic university hospitals and specialized cancer centers with established outpatient breast cancer clinics. All clinical assessments and study-related procedures were performed in routine outpatient settings according to a harmonized study protocol. Recruitment began in December 2017 and was completed in May 2021 with a total of 1550 patients randomized. Data collection occurred in parallel with recruitment and continued until completion of the scheduled follow-up assessments (22 months after completion of accrual). Data were collected in MEDCODES v6.0, and in MARVIN by Xclinical.

Outcomes

The primary endpoint was EFS, defined as time from randomization to the occurrence of any of the following events: disease progression on protocol therapy resulting in initiation of non-protocol cancer therapy or inoperability, local invasive recurrence following mastectomy, local invasive recurrence in the ipsilateral breast after lumpectomy, regional recurrence, distance recurrence, contralateral invasive breast cancer, a second non-breast primary malignancy, or death from any cause.
Secondary endpoints included pCR in the breast and lymph nodes evaluated after surgery (ypT0/Tis ypN0) defined as the absence of

any invasive residual tumor in the resected breast specimen and lymph nodes following completion of neoadjuvant therapy; overall survival (OS), defined as the time from randomization to death due to any reason; distant disease-free survival (DDFS), defined as time from randomization until distant recurrence, death from breast cancer, death from other causes, or occurrence of second primary invasive non-breast cancer; and disease-free survival (DFS), defined as time from first breast surgery to disease recurrence or death from any cause.
Safety by toxicity grades was defined by the National Cancer Institute Common Terminology Criteria for Adverse Events (NCI-CTCAE) version 4.0. Cardiac safety was evaluated in the first 60 patients randomized and who initiated AC/EC in the cardiac safety lead-in cohort. The evaluation was based on the frequency of elevated troponin-T levels and changes in LVEF, to identify and further assess potential cardiac toxicity associated with the combination of anthracycline and atezolizumab. Brain metastases-free survival was an exploratory endpoint.

Plants

Seed stocks

not applicable.

Novel plant genotypes

not applicable.

Authentication

not applicable.
